# Genomic evidence of bitter taste in snakes and phylogenetic analysis of bitter taste receptor genes in reptiles

**DOI:** 10.7717/peerj.3708

**Published:** 2017-08-18

**Authors:** Huaming Zhong, Shuai Shang, Xiaoyang Wu, Jun Chen, Wanchao Zhu, Jiakuo Yan, Haotian Li, Honghai Zhang

**Affiliations:** 1 College of Life Science, Qufu Normal University, Qufu, Shandong, China; 2 Ocean University of China, College of Marine Life Sciences, Qingdao, Shandong, China; 3 Shandong University, Marine college, Weihai, Shandong, China

**Keywords:** Bitter taste, Snake, Calhm1, Reptiles, *Tas2r*, Diet

## Abstract

As nontraditional model organisms with extreme physiological and morphological phenotypes, snakes are believed to possess an inferior taste system. However, the bitter taste sensation is essential to distinguish the nutritious and poisonous food resources and the genomic evidence of bitter taste in snakes is largely scarce. To explore the genetic basis of the bitter taste of snakes and characterize the evolution of bitter taste receptor genes (*Tas2r*s) in reptiles, we identified *Tas2r* genes in 19 genomes (species) corresponding to three orders of non-avian reptiles. Our results indicated contractions of *Tas2r* gene repertoires in snakes, however dramatic gene expansions have occurred in lizards. Phylogenetic analysis of the *Tas2r*s with NJ and BI methods revealed that *Tas2r* genes of snake species formed two clades, whereas in lizards the *Tas2r* genes clustered into two monophyletic clades and four large clades. Evolutionary changes (birth and death) of intact *Tas2r* genes in reptiles were determined by reconciliation analysis. Additionally, the taste signaling pathway calcium homeostasis modulator 1 (*Calhm1*) gene of snakes was putatively functional, suggesting that snakes still possess bitter taste sensation. Furthermore, Phylogenetically Independent Contrasts (PIC) analyses reviewed a significant correlation between the number of *Tas2r* genes and the amount of potential toxins in reptilian diets, suggesting that insectivores such as some lizards may require more *Tas2r*s genes than omnivorous and carnivorous reptiles.

## Introduction

Vertebrates possess five basic tastes: sweet, umami, bitter, salty, and sour (Bachmanov & Beauchamp, 2007; Lindemann, 1996). It is generally believed that bitter transduction evolved as a key defense mechanism against the ingestion of toxic substances. As bitter foods generally contain harmful or toxic compounds, the bitter taste sensation is essential for animals to evaluate the potential food resources and distinguish between nutritious and toxic food (Garcia & Hankins, 1975; Glendinning, 1994). Many bitter substances come from not only plants, but also from insects and meat tissues (Cott, 2010; Drewnowski & Gomezcarneros, 2000; Garcia & Hankins, 1975;Glendinning, 1994; Howse, 1975; Unkiewicz-Winiarczyk & Gromysz-Kałkowska, 2012). The bitter perception is mediated by a group of G-protein-coupled receptors taste receptor member 2 (*Tas2r*) which are encoded by the *Tas2r* gene family (Adler et al., 2000; Chandrashekar et al., 2000; Matsunami, Montmayeur & Buck, 2000). Different animal species have distinct diets and therefore different bitter compounds are detected by different *Tas2r*s (Meyerhof et al., 2010). Thus the *Tas2r* repertoires are expected to be species-specific. Indeed, the number of intact *Tas2r* genes varies among species, from 0 to 37 in mammals (Feng et al., 2014; Shi & Zhang, 2006), from 1 to 12 in birds (Wang & Zhao, 2015) and 0 to 74 in fish (Bachmanov et al., 2013; Syed & Korsching, 2014), due to the duplication or loss of *Tas2r* genes in what is called a “birth-and-death” process, characteristic of chemosensory genes (Nei, Gu & Sitnikova, 1997). Different *Tas2r* repertoires among species may reflect varying dietary pressures. Generally speaking, among animals potential foods, plant tissues may contain more toxic compounds than animal tissues (Glendinning, 1994; Wang, 2004). Insects and some amphibians like newts also secrete defensive poisonous chemicals to deter predators (Brodie & Tumbarello, 1978; Howse, 1975). Hence, more functional *Tas2r* genes exist in herbivorous, omnivorous and insectivorous animals than in carnivorous animals, implying that distinct diets may shape the variation of *Tas2r* repertoire among species (Li & Zhang, 2014; Wang & Zhao, 2015). Factors other than diets like foraging pattern may also affect *Tas2r* numbers. For example, cetaceans swallow their food whole, and 9 out of 10 amplified *Tas2r* genes from 12 whales were pseudogenized (Feng et al., 2014).

Reptiles (10,450 species) live in a wide range of terrestrial, marine and freshwater environments. Among them, snakes (3,619 species) and lizards (6,263 species) comprise of 87 families, according to The Reptile Database (http://reptile-database.org/, last accessed July 18, 2017). Studies of taste genetics in vertebrate species have been mainly focused on mammals and birds (Behrens, Korsching & Meyerhof, 2014; Behrens & Meyerhof, 2013; Davis et al., 2010; Dong, Jones & Zhang, 2009; Go, 2006; Hayakawa et al., 2012; Hong & Zhao, 2014; Jiang et al., 2012; Li & Zhang, 2014; Liu et al., 2016; Nei, Gu & Sitnikova, 1997; Feng et al., 2014; Shang et al., 2017; Shi & Zhang, 2006; Shi et al., 2003; Wang & Zhao, 2015; Wang, 2004; Wu, Chen & Rozengurt, 2005; Zhou, Dong & Zhao, 2009), however, similar studies of *Tas2r* genes of snakes have not been performed. The strictly carnivorous diet of snakes comprises few toxins, although it may contain defensive toxic secretions released by insects or highly toxic newts (Brodie, Ridenhour & Iii, 2002; Mehrtens, 1987; Parker & Gans, 1978). Besides, snakes cannot masticate and they have to swallow the preys whole without chewing. Therefore, we hypothesized that *Tas2r*s would still exist but would be smaller in number in snakes according to their unique dietary and foraging pattern, compared with the number of *Tas2r* genes in lizards, the other suborder in Squamates, many of which are primarily insectivorous and need to to avoid intake of poisonous secretions released by insect prey. Apart from taste receptors, the taste pathway gene calcium homeostasis modulator 1 (*Calhm1*) that is responsible for taste signalling is also indispensable for taste function (Taruno et al., 2013). Thus, *Calhm1* might be intact and functional if bitter taste is retained in snakes. To test our hypothesis, we identified *Tas2r* genes from eight snake species for the first time. Additionally, we identified the *Calhm1* gene to explore the function of the taste signaling pathway in Squamates. We found that snakes have a considerable contraction of the *Tas2r*s repertoire, which was in accordance with their foraging and dietary patterns. In contrast, the *Tas2r* family in the two lizard species (the green anole and the Japanese gecko) we examined expanded dramatically, which was consistent with their insectivorous diet. Furthermore, *Calhm1* genes were under strong purifying selection, implying the retention of bitter taste functionality in Squamates.

## Materials & Methods

### Genome data

The Squamates comprise two suborders: Serpentes (snake) and Lacertilia (lizard). A total of eight Serpentes genome sequences were downloaded from GenBank (http://www.ncbi.nlm.nih.gov/). They are corn snake (*Pantherophis guttatus*) and common garter snake (*Thamnophis sirtalis*) in Family Colubridae; speckled rattlesnake (*Crotaus mitchellii*), timber rattlesnake (*Crotalus horridus*), adder (*Vipera berus*) and brown spotted pit viper (*Protobothrops mucrosquamatus*) in Family Viperidae; king cobra (*Ophiophagus hannah*) in family Elapidae; the Burmese python (*Python bivittatus*) in family Pythonidae. In suborder Lacertilia*,* genome sequences of the green anole (*Anolis carolinensis*) and the Japanese gecko (*Gekko japonicas*) were retrieved. We also retrieved nine additional genome sequences of other reptiles to conduct further phylogenetic analysis. They were Spiny Softshell Turtle (*Apalone spinifera*), Green Sea Turtle (*Chelonia mydas*), Painted Turtle (*Chrysemys picta*), Diamondback Terrapin (*Malaclemys terrapin*) and Chinese Softshell Turtle (*Pelodiscus sinensis*) in Testudines; Saltwater Crocodile (*Crocodylus porosus*), American Alligator (*Alligator mississippiensis*), Chinese Alligator (*Alligator sinensis*) and Gharial (*Gavialis gangeticus*) in Crocodylia. Hence, a total of 19 genomes of reptiles were retrieved from GenBank (Table S1). The sequencing depths of the assemblies were all above 15 × except in green anole (7.1 ×) and corn snake (13 ×). Scaffold N50 sizes ranged from 2.4 to 437.3 Mb, suggesting high-coverage genomes.

### Gene identification

*Tas2r* genes are about 900 bp without introns. First, we collected previously determined intact *Tas2r* protein sequences from human, mouse, lizard, chicken, frog and coelacanth genomes as queries (Li & Zhang, 2014; Syed & Korsching, 2014). We used TBLASN to search against the genomes with the *E*-value of 1e–10. Second, non-overlapping sequences were extracted and the blast hits shorter than 100 bp were discarded. We extracted the remaining blast hits and extended them in both 5′ and 3′ directions. They were regarded as putative *Tas2r* regions. Third, the deduced sequences were divided into three categories. Sequences more than 270 amino acids with a presumed start and stop codon were considered intact genes. The TMHMM method was used to make sure the seven transmembrane domains existed in intact genes (Krogh et al., 2001). Sequences longer than 100 bp with premature stop codons or frame-shifts were considered pseudogenes. Sequences longer than 100 bp containing a start codon (or a stop codon) were regarded as partial genes. Partial *Tas2r*s may be intact genes, but they were not sequenced completely or assembled completely (Hayakawa et al., 2014). To ensure the reliability of the partial genes, we assessed whether the partial genes are from independent loci or not (Table S2). We also performed syntenic analysis to determine whether partial genes are unique (Table S3). Lastly, we used BLASTP to search against GenBank to verify all the candidate genes belonging to the *Tas2r* family. The nomenclature of the *Tas2r* genes followed ‘*Tas2r* X’, in which ‘X’ represents arabic numbers one by one. See Data S1 and  S2 for the nucleotide and amino acid sequences.

### Phylogenetic analyses

To clarify the evolutionary history and relationships of the *Tas2r* genes in reptiles, phylogenetic analysis was conducted. Non-avian reptiles contain four general groups: Squamata (snakes and lizards), Crocodylia (alligators and crocodiles), Testudines (turtles) and Sphenodontia (tuatara). In the present study, tuataras were not included because no genome data was available in this group. The protein sequences of intact *Tas2r* genes were aligned by MUSCLE (Thompson, Higgins & Gibson, 1994) implemented in MEGA5 (Tamura et al., 2011) with manual adjustments. The western clawed frog (*Xenopus tropicalis*) *V1R* gene was used as outgroup, because among GPCRs, *V1R* genes are relatively close to *Tas2r* genes. Partial *Tas2r* genes were not included because most of them were too short to make a good alignment. Pseudogene sequences were also removed from the phylogenetic analysis. The best-fitting substitution model Jones-Taylor-Thornton (JTT) +G +F was determined by ModelGenerator 0.85 (Keane et al., 2006). After gaps were removed from the alignment with the pairwise deletion option, the neighbor-joining method (Saitou & Nei, 1987) implemented in MEGA5 was used to construct a phylogeny. Statistical confidence was evaluated with 1,000 replicates by the bootstrap method (Felsenstein, 1985). The BI (Bayesian Inference) (Yang & Rannala, 1997) tree was rebuilt by MrBayes 3.2.6 (Ronquist & Huelsenbeck, 2003) under the same model of substitution with 10 million generations.

### Evolutionary analysis of *Tas2r*s repertoire

To recover evolutionary changes of *Tas2r* gene numbers in reptiles, we conducted a reconciliation analysis by NOTUNG 2.6 (Chen et al., 2004). This method is based on algorithms for tree rearrangement and for reconciliation with non-binary trees. It supports predicting gene gain and loss events with a parsimony-based optimization criterion. We used our BI tree as the gene tree (Fig. S1). The phylogeny and divergence times of these species were drawn from TimeTree (http://www.timetree.org/).

### Phylogenetically Independent Contrasts (PIC) analyses

To explore the potential impact of diets on the evolution of the *Tas2r*s repertoires of reptiles, we carried out a regression analysis of *Tas2r* gene numbers against the diet type, which we summarized as a diet code. As insect and plant tissues contain more potential bitter compounds (toxins) than non-insect animal tissues, we coded the species as 0 (carnivore) and 1 (insectivore and herbivore) according to the amount of plant materials, insect tissues or non-insect animal tissues in their diets (Table S1). The omnivorous species painted turtle was coded as 1 because its diet appeared to contain more plant materials than animal tissues. We categorized the *Tas2r* genes into two sets: The first comprised putatively functional genes (intact and partial genes), and the second consisted of all genes (intact, partial and pseudogenes) because not only functional genes, but also pseudogenes may reflect the physiological demands (Rouquier, Blancher & Giorgi, 2000). Then we conducted a phylogenetically independent contrast (PIC) analysis with the R package Analyses of Phylogenetics and Evolution (APE) (Paradis, Claude & Strimmer, 2004). The correlation was assessed with nonparametric Spearman’s rank correlation coefficient (ρ).

## Results

### Identification of Tas2r genes

**Figure 1:**
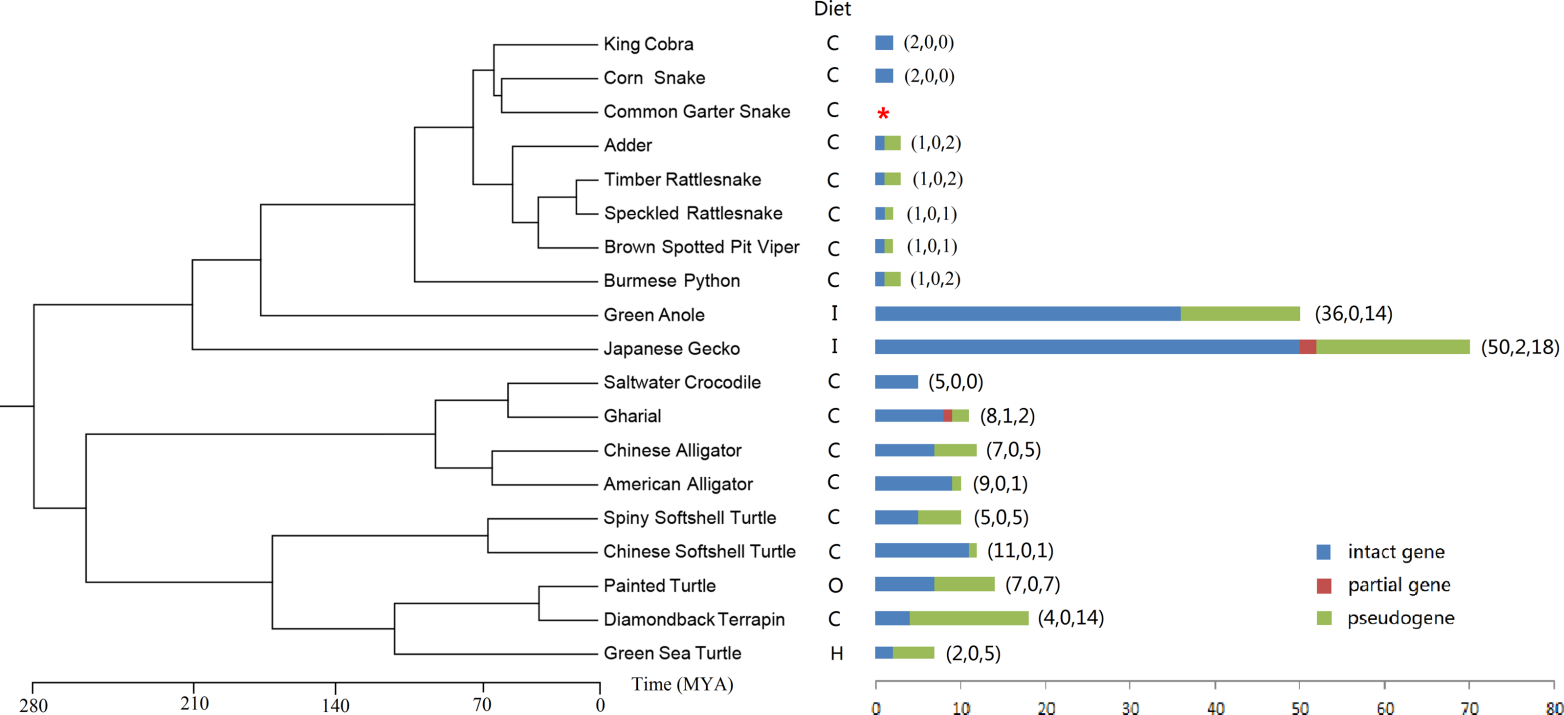
Numbers of intact, partial and pseudo *Tas2r* genes and dietary preferences (C: Carnivore; I: Insectivore; O: Omnivore; H: Herbivore) of the 19 reptile species. Species tree and divergence times were taken from the literatures and TimeTree (http://www.timetree.org/, see Table S4). “*”indicates no *Tas2r* gene was as detected in the common garter snake.

By TBLASN, we identified *Tas2r* genes from 19 genome sequences of reptiles. The result revealed that speckled rattlesnake and brown spotted pit viper followed the same pattern with one intact gene and one pseudogene. In the timber rattlesnake, adder and the Burmese python, the numbers were one intact gene and two pseudogenes. The corn snake and king cobra both possess two intact genes (Fig. 1). All the eight species showed absence of partial genes. Strikingly, no *Tas2r* gene was detected in common garter snake. The average number of intact genes of the seven snakes (except garter snake) was 1.3 and the average total number of *Tas2r*s was 2.4. Overall, the repertoires of *Tas2r*s in snake species were generally small than that in other vertebrates (Li & Zhang, 2014; Liu et al., 2016; Wang & Zhao, 2015). The nucleotide length of intact *Tas2r* genes of the 7 snake species was from 885 to 966 bps with the average of 904 bp and the amino acid sequence identities were ranging from 32.73% to 94.22%. By contrast, the two lizard species had a relatively larger *Tas2r*s repertoire. We identified 36 intact genes, 0 partial gene and 14 pseudogenes in the green anole (*Anolis carolinensis*). In the Japanese gecko (*Gekko japonicas*), the numbers were 50, 2 and 18 respectively. The average number of intact genes of the two lizards was 33 times of snakes’, with the exception of the common garter snake. The length of intact genes ranged from 870 to 1,077 with the average of 937 bp and the amino acid sequence identities were ranging from 17.24% to 95.42%. Another obvious difference between lizards and snakes was the number of pseudogenes: we discovered 14 in the green anole, and 18 in the Japanese gecko respectively (Fig. 1). In the five Testudines genomic assembles, we detected 2–11 intact *Tas2r* genes and 1–14 pseudogenes; the total number of *Tas2r* genes was 7–18 (Fig. 1). In order Crocodylia, the numbers of intact *Tas2r* genes, pseudogenes and total genes were 5–9, 0–5 and 5–12 respectively.

### Phylogenetic analysis

**Figure 2:**
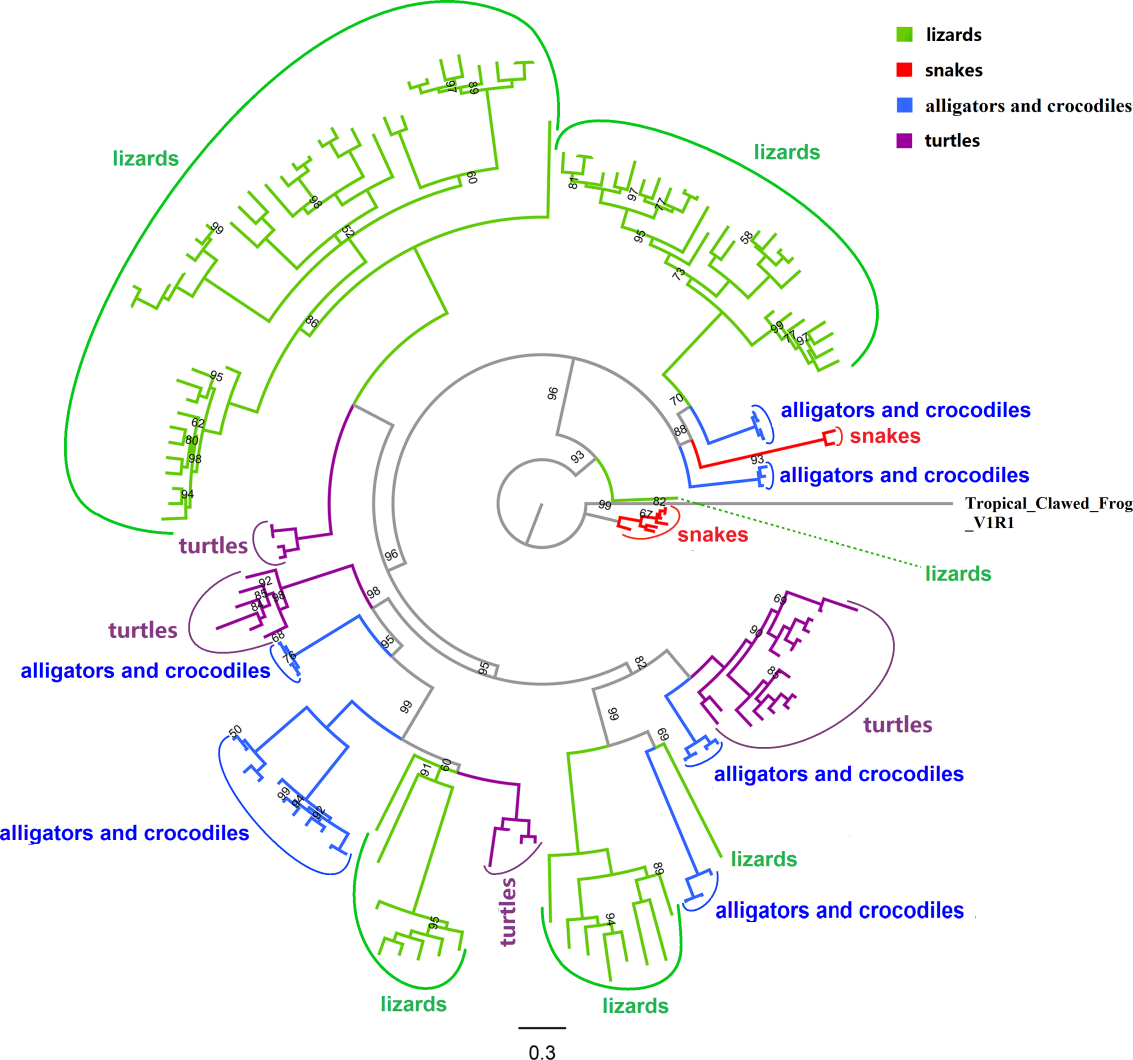
Phylogenetic relationship of all 154 intact *Tas2r* genes from 19 reptiles. The western clawed frog (*Xenopus tropicalis*) *V1R1* gene was used as an outgroup. The tree was reconstructed using the BI method with the best-fitting model of JTT+G+F. Branch lengths were drawn to the scale. Posterior probabilities from Bayesian inference analyses are presented in percent, in which the value of 100 was not shown. The detailed information about species, gene names and posterior probabilities was shown in Fig. S1. The NJ tree showing a similar topology to this tree was provided in Fig. S2.

To investigate the phylogenetic relationship of *Tas2r* genes within reptiles, we conducted a phylogenetic analysis and constructed phylogenies with both the NJ and BI methods. As we can see from the BI tree (Fig. 2), the *Tas2r* genes of seven snake species (common garter snake was excluded) formed two monophyletic clades. In comparison, the *Tas2r* genes of lizards mainly clustered into two single-gene clades and four large clades, two of which were consisted of large numbers of *Tas2r* genes. After testing gene conversion among paralogous genes with GENECONV (Sawyer, 1989), we detected no possible events of gene conversion, suggesting that gene conversion may not have played a role in the evolution of lizards’*Tas2r* repertoire. We infer that the large and separate *Tas2r* repertoire in lizards was due to lineage-specific gene duplications, as demonstrated by our evolutionary analysis(see results below). Turtles (Testudines) had four separate ancestral nodes, two clades of which mingled with lizards and the other two with alligators and crocodiles (Crocodylia). The alligators and crocodiles had six segregated clades (Fig. 2).

### Evolutionary analysis of *Tas2r*s repertoire

**Figure 3:**
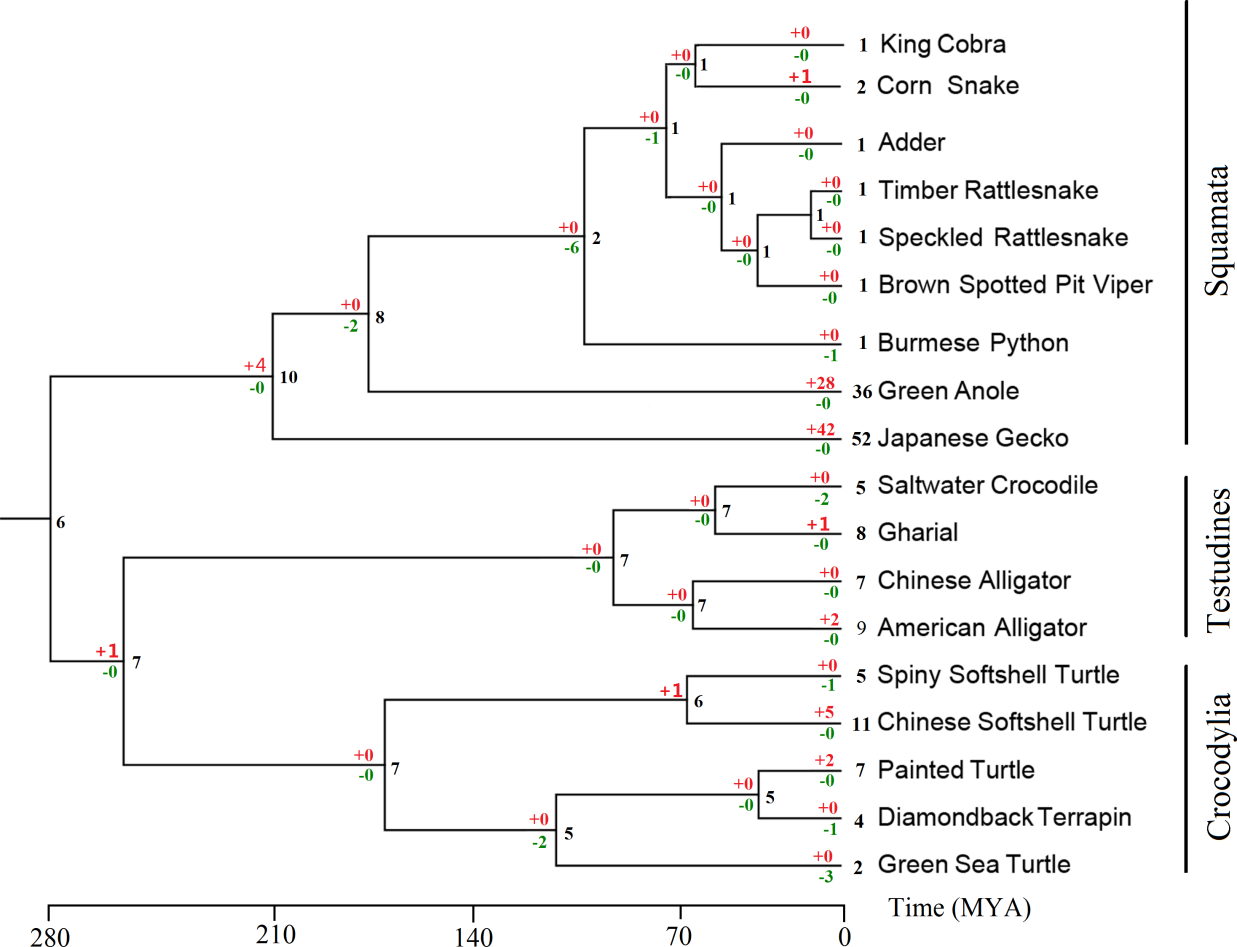
Evolutionary changes of intact *Tas2r* gene numbers in 19 reptiles. The estimated *Tas2r* gene numbers for ancestral lineages were indicated with black, whereas the numbers of gene gains and losses were shown with red and green, respectively. The common garter snake was not included because no *Tas2rs* gene was detected in the genome assembly.

To determine the evolution of *Tas2r* gene repertoires among non-avian reptile species, we predicted the intact *Tas2r*s numbers in the common ancestors of different clades. Then we performed a reconciliation analysis and inferred the birth and death of intact *Tas2r* genes. The BI tree was used (Fig. S1) to predict the changes of gene numbers, because the Bayesian posterior probability of each branch is above 50% and may better support the analysis than the NJ tree (Fig. S2). We found that the common ancestor of Squamata showed a gain (*n* = 4) when they were diverging with the common ancestor of Testudines and Crocodylia. In Squamata, the number of intact *Tas2r* genes in the common ancestor of snakes was only 2 (Fig. 3), indicating a reduction when snakes diverged with lizards. In contrast, green anole (36 intact genes) and the Japanese gecko (50 intact genes) both showed large numbers of species-specific duplication (28 and 40 respectively) (Figs. 1 and 2). Besides, a further gain (*n* = 1) happened in the common ancestor of Testudines and Crocodylia. In Testudines, gain and loss events did not happen in their common ancestor, but in each branch. There were two losses, one gain and two gains in saltwater crocodile, gharial and the American alligator, respectively. The evolutionary changes of the *Tas2r* gene number in the three species were consistent with the results from a recent study (Wang & Zhao, 2015). In Crocodylia, substantial gains (*n* = 5) were found in the Chinese softshell turtle.

### Correlation between dietary preferences and *Tas2r*s repertoire

**Figure 4:**
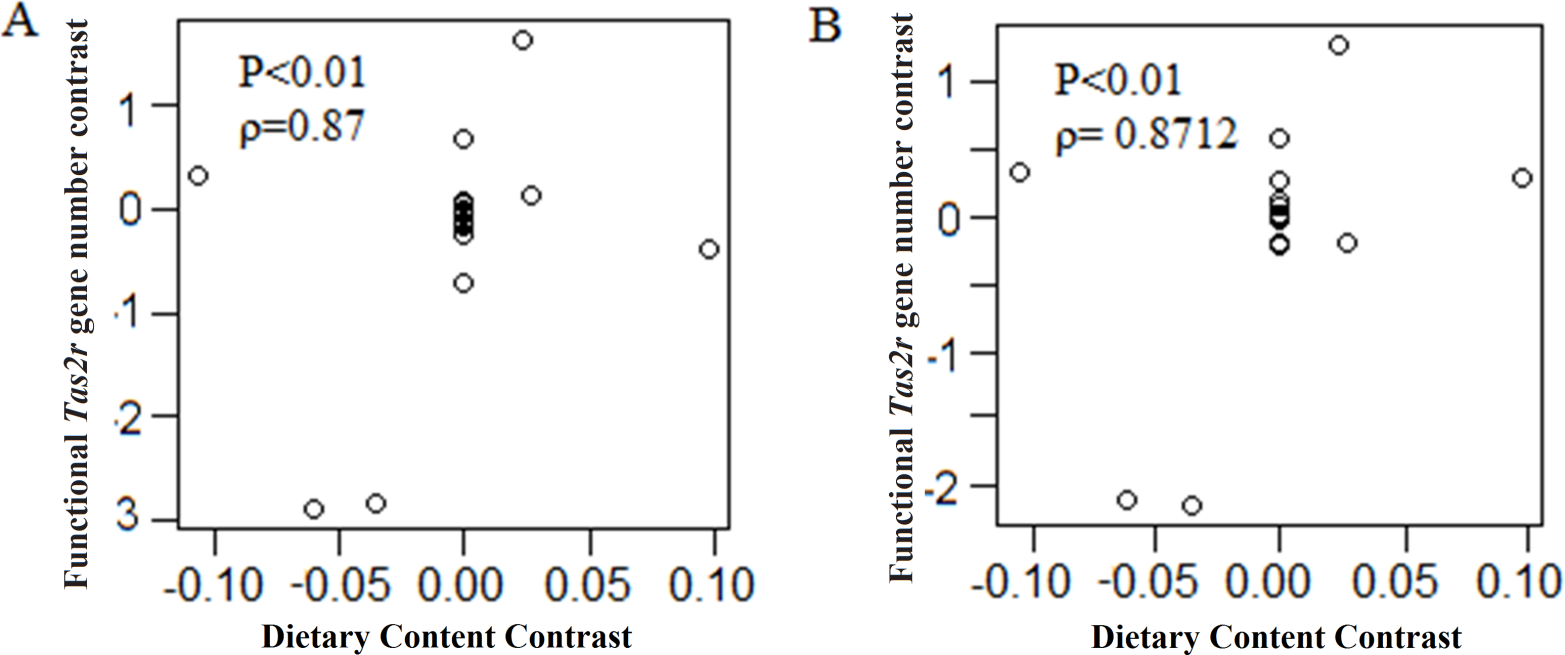
Dietary preferences impact the reptilian *Tas2r* gene repertoires. (A) PICs in putatively functional *Tas2r* gene number is positively correlated with PICs in diet content code; (B) PICs in total *Tas2r* gene number is also positively correlated with PICs in diet content code. The association was evaluated by the Spearman’s rank correlation coefficient (*r*).

To test the potential impact of the dietary preferences on the evolution of *Tas2r* gene repertoires, we divided the 19 reptiles into carnivores, insectivores, herbivores and omnivores (Table S1). Then we coded the diet as 0 (carnivore) and 1 (insectivore, herbivore and omnivore). After performing a regression analysis of *Tas2r* gene numbers against the diet codes, we detected a significant positive correlation between the number of functional *Tas2r* genes and dietary preference (*r* = 0.87  *P* < 0.01; Fig. 4A). The same correlation was also observed between the number of total *Tas2r* genes and dietary preference (*r* = 0.8712, *P* < 0.01; Fig. 4B). Our findings unambiguously indicated a significant positive correlation between the repertoire of *Tas2r* genes in reptiles and the abundance of potential toxins in their diets.

## Discussion

Snakes have evolved to be a nontraditional model organism with extreme physiological and morphological phenotypes, such as enhanced chemoreception (Castoe et al., 2013). Though studies on behavior and anatomy of Squamates have proposed them as alternative models for taste studies (Young, 1997), the genetic basis of taste sensation of snakes has not yet been assessed. This is partly owing to the belief that snakes rely more on vision, olfaction and even infrared sensory than on taste for feeding (Gamow & Harris, 1973; Grace et al., 1999; Halpern & Kubie, 1983; Walls, 1944). Anatomical studies revealed that snakes are lack of taste buds on the mucosa of jaw and tongue (Auen & Langebartel, 1977; Uchida, 1981; reviewed in Young, 1997). Consequently, it has been suggested that the sense of taste may be reduced (Auen & Langebartel, 1977; Uchida, 1981). In the present study, we identified *Tas2r* genes from the genomes of 8 snake species representing five families in Serpentes for the first time. Our results revealed that lineage-specific gene expansions and contractions leading to different numbers of *Tas2r*s in different lineages also happened in Squamates. The extensive gene contraction in this family found in birds and cetaceans (Dong, Jones & Zhang, 2009; Hillier et al., 2004) was here also identified in snakes. Reconciliation analysis indicated that many *Tas2r* genes have been lost in the common ancestor in the snake lineage (Fig. 3). By contrast, genes encoding olfactory and vomeronasal receptors all show considerable expansions in snakes (Castoe et al., 2013), implying that they may rely more on other sensory systems such as olfaction than on bitter taste sensation in detecting toxic compounds.

No *Tas2r* genes were detected in the common garter snake. It is likely because the genome of the common garter snake contains so much ambiguous sequencing (poly N) that might prevent identification of the true number of *Tas2r* genes. Studies regarding feeding habits showed that the common garter snake would exclude some toxic frogs after making oral contact with them, suggesting they could sense the bitterness of poisonous food (Brodie & Tumbarello, 1978; Dumbacher, Menon & Daly, 2009). Consequently, the numbers of *Tas2r* genes in the common garter snake must be regarded as tentative and needs to be validated further.

Interestingly, both of the 2 lizard species examined here displayed a noticeable expansion of the *Tas2r* repertoire, which was also detected in the western clawed-frog (*Xenopus tropicalis*) and coelacanth (*Latimeria chalumnae*) (Behrens, Korsching & Meyerhof, 2014; Wang & Zhao, 2015). In contrast to snakes, the tip of tongue in most lizards has a high concentration of taste papillae (Schwenk, 1985). In concordance with this anatomical evidence, responses to bitter-compound-coated preys has also been widely demonstrated in many lizards (Benes, 1969; Dumbacher, Menon & Daly, 2009; Paradis & Cabanac, 2004; Price-Rees, Webb & Shine, 2011; Pricerees, Brown & Shine, 2012; Shanbhag, Ammanna & Saidapur, 2010; Stangerhall et al., 2011). Additionally, the number of pseudogenes was considerably higher than that of snakes and other reptiles. To determine whether tandem duplication had taken place in reptilian *Tas2r*s as it has in mammals and birds (Wang & Zhao, 2015), we examined the genomic location for the *Tas2r* genes. The result indicated that some *Tas2r*s are aligned in arrays (Table S5). This is consistent with the birth-and-death model of multigene family evolution in which the more gene duplications occur, the more pseudogenes are found (Nei, Gu & Sitnikova, 1997).

The dramatic changes of *Tas2r* repertoire size may reflect different selection pressures and different evolutionary processes faced by each organism in accordance with constantly changing environments (Go, 2006). On one hand, as a driving force of evolution, diets including different sets of toxins have been suggested to have shaped the variation of *Tas2r* repertoire across species (Li & Zhang, 2014; Wang & Zhao, 2015). In the present study, we extended this hypothesis to a different group of Squamates. It has been demonstrated that animals with extremely narrow diets may have small functional *Tas2r* repertoires. For example, vampire bats which feed exclusively on blood have a considerably smaller percentage of intact *Tas2r*s than other bats (Hong & Zhao, 2014). Snakes are strictly carnivores, feeding on animals including small mammals, birds, eggs, frogs, lizards, snails, fish, insects or other snakes (Mehrtens, 1987; Parker & Gans, 1978). This food composition contains few toxic chemicals, except for some defensive toxic secretions released by insects or newts (Brodie, Ridenhour & Iii, 2002). On the other hand, the foraging pattern may also affect the *Tas2r* repertoire. For example, swallowing preys items whole without chewing results in less contact with poisonous foods. In minke whales, eight out of nine *Tas2r* genes identified from 12 whale genomes are pseudogenes (Feng et al., 2014): this perhaps also relates to foraging patterns. Considering the dietary and swallowing foraging pattern of snakes, a reduced *Tas2r* repertoire makes sense. However, the small *Tas2r* repertoires in snakes do not necessarily imply a reduced importance of bitter taste, as smaller repertoire size could be compensated for by a large tuning breadth to bitter substances, as has been shown in human, chicken and turkey *Tas2r*s (Behrens, Korsching & Meyerhof, 2014; Meyerhof et al., 2010). In contrast, the green anole and the Japanese gecko are lizards with diets that mainly consist of small insects like grasshoppers, crickets, flies spiders, and other arthropods. Since most of the prey can release defensive toxic secretions (Braswell et al., 1980; Jensen et al., 2008). Therefore, lizards may encounter more bitter substances than snakes and more *Tas2r* genes may be required to help avoiding certain harmful compounds in insects. Our PICs analysis clearly showed a significant positive correlation between the repertoire of *Tas2r*s in reptiles and the amount of potential toxins in their diets. However, this analysis will be strengthened when more *Tas2r* data of reptiles are available. Furthermore, our results do not necessarily mean that there are no other factors influencing the *Tas2r* repertoire. Extra-oral functions in other systems outside the oral cavity (e.g., in central nervous system, respiratory system or cardiovascular system) may also drive the evolution of *Tas2r*s genes (Behrens, Prandi & Meyerhof, 2014). Some studies have suggested neutral (rather than diet-related) reasons that are responsible for variations in chemosensory repertoire size. For example, Go found that the contraction of avian *Tas2r* repertoire was concomitantly with reduction of the genome size (Go, 2006). In order to determine the potential relationship between the *Tas2r* repertoire and genome size in reptiles, we calculated the average genome size of reptiles using data from Animal Genome Size Database (http://www.genomesize.com/), which provide genome size as haploid DNA contents (*C*-values, in pg). As the *Tas2r* repertoire of snakes was smaller than birds whereas the average genome size of snakes (2.14 pg) was larger than birds (1.43 pg), we inferred that genome size reduction was not the necessary cause for the contraction of the *Tas2r* repertoire in snakes (Table S6). Meanwhile, the average genome size of snakes (2.14 pg) and lizards (2.15 pg) were approximately the same, suggesting that the contraction and expansion of *Tas2r* genes were not related to genome size in the Squamates clade.

Apart from taste receptors, signaling pathways downstream of taste receptors are indispensable for taste function. For instance, calcium homeostasis modulator 1 (*Calhm1*) mediates purinergic neurotransmission of bitter taste stimulant. *Calhm1* knock-out mice showed severely damaged reactions to bitter stimuli (Taruno et al., 2013). Actually, pseudogenization of *Calhm1* gene occurred in some species. For example, Feng found that *Tas2r* genes were pseudogenized in 11 whale species. Among these species, the Beluga Whale (*Delphinapterus leucas*) and the Omura’s whale (*Balaenoptera omurai*) showed indels in *Calhm1* gene, resulting in pseudogenization and functionless. However, the *Calhm1* gene of 9 other whales is intact and putatively functional (Feng et al., 2014). By TBLASTN, we successfully identified intact *Calhm1* genes in each genome of the eight snake species (Data S3 and S4). Furthermore, to explore the functional implications of *Calhm1* in snakes, we estimated the *ω* ratio for the *Calhm1* genes with a likelihood approach by PAML. The approach was based on the ratio of non-synonymous to synonymous substitution rates (dN/dS, *ω*), with *ω* being <1, equal to 1 and >1 indicating negative or purifying selection, neutral evolution and positive selection, respectively. The *ω* ratio for the *Calhm1* genes is significantly smaller than 1 (*ω* = 0.12, Table S7), suggesting that *Calhm1* genes are under strong purifying selection and thus putatively functional in snakes. Combined with the genomic data of intact and deduced functional *Tas2r* genes in snakes, our result explicitly indicated that snakes still retain the genetic basis for bitter taste perception.

We systematically identified the *Tas2r* genes in 19 reptiles, and present the first description of the *Tas2r* repertoires of snakes. Our analysis of genomic data demonstrates that snakes may indeed possess bitter taste perception. These results point to a reduction of *Tas2r*s in snakes-at least in the nine species we analyzed-and provide novel insights into the evolutionary biology of taste perception and food selection of snakes and other reptiles. Future expressional and behavioral studies regarding bitter taste of reptiles will be interesting to pursue.

##  Supplemental Information

10.7717/peerj.3708/supp-1 Data S1 All intact Tas2r gene sequences

10.7717/peerj.3708/supp-2 Data S2 All intact Tas2r protein sequences

10.7717/peerj.3708/supp-3 Data S3 *Calhm1* gene sequences of snakes

10.7717/peerj.3708/supp-4 Data S4 *Calhm1* protein sequences of snakes

10.7717/peerj.3708/supp-5 Figure S1 BI tree of 154 intact Tas2r genes of reptiles

10.7717/peerj.3708/supp-6 Figure S2 NJ tree of 154 intact Tas2r genes of reptiles

10.7717/peerj.3708/supp-7 Table S1 References of the dietary preferences and genomic contig N50 statistics for the 19 reptiles studied

10.7717/peerj.3708/supp-8 Table S2 Classification of partial *Tas2rs* in the genome assemblies of reptiles

10.7717/peerj.3708/supp-9 Table S3 Syntenic analysis for partial genes

10.7717/peerj.3708/supp-10 Table S4 References of divergence time tree taken from http://www.timetree.org/

10.7717/peerj.3708/supp-11 Table S5 Genomic locations for all reptile *Tas2r* genes studied. Potential tandem duplicated genes were highlighted in shadow

10.7717/peerj.3708/supp-12 Table S6 Genome Size in reptiles Genome size (pg) is the amount of DNA in one copy of a species’ chromosomes or the haploid nuclear DNA content.

10.7717/peerj.3708/supp-13 Table S7 Likelihood ratio tests of selective pressures on *Calhm1* genes of snakes Branch model in PAML was used to compute the *ω* value.

## Additional Information and Declarations

### Data Availability

The following information was supplied regarding data availability: The raw data has been supplied as a Supplementary File.
