# Supplementary material for: Genomic evidence of bitter taste in snakes and phylogenetic analysis of bitter taste receptor genes in reptiles
Source: PeerJ. 2017 Aug 18;5:e3708. doi: 10.7717/peerj.3708 (PMC5564386; doi:10.7717/peerj.3708)
Supplement: Data S1 [file peerj-05-3708-s001.docx]

>Adder_Tas2r1

ATGTCAGTGTCAGGAGTTCACAACTGGCTCTGCCTTATTATCACCACAGCTGTGACTCTCGTCGGGATGACTGGAAACGGATTCATCTTTCTTTCAGACTGCCAGGATTGGATCCGAAGCAAAACATCGTCAGGTCCTGGCCTCTTGCTGATGATGCTCAGCCTGACCCGATTTATTTTCCTCGGACTCACGCTCAGCTTACATTGTTTAAGCTTCCTTGATATTAATCGCCCAAAATTTGCCGGAAGTGTCACCATTTTCTTCTGGGCCTTTTTCAATGCCACCACCCTGTGGATTACTACCTGCCTTGGAATATTCTACTGTGTGAAAATTGTCAACTTCAGCCAGCCCTTCCTGGTGAAAGCGAAGCTGAGGATTTCCAGCATGATTCCCCATCTGCTCGTCGCGGTAGTCTTGGTTTCCTTGATTTCGGCTCTTCCTTTCCTCTGGATCGACGATCACAGCCAGTCTGACAACACAGAGGATGTACCTGAGATGAGGGCTCGGATGTTGCTGCTCAGTATACTGTATATTCTGGGGACTTTTCCATCGTTTGTGATATTTTTAATTTCCTCTGGATTTTTAATTTACTCCCTTGTGCAGCATGTGAAGAGGATGCAGAACAGCTCAGTTGGCTTTCGAGATCAAAGGATGGACGTTCATCTGAAGACCACCAAGATATTGACCTCCTTCTTCATCCTCTATACCGCAACCTTTGTAACAGAAATCTCAATGACCTTTTCCCCCAGTCCCTGGACATTAGTTATATCCAACATGGTGGTTAGTTCCTATAATTCAGGGCACACCGTTGCTTTGATTGTCATGAATTCCAAGCTAAGGGGACGATTAAGCAAGATGTTCTGGTGTTTCAGAAAACAGACATGA

>American_alligator_Tas2r1

ATGACTCCTTTAAATGTTGTTACTTTGATAGGTTTAGCAATTGAGTTTGTTGTGGGTATTATAGCAAATGGATTGATAGTAGGTTTTAATTGCATTGCATGGATTAAAAGTCAAAAACTAGATTCGTGTGCCTTAGTCCTGATTAGCCTGGGGACCTCCAGATTTTTTTTACTGTATGCAATATTGATTAATAATATTTTCTTTGCAATTCCTAAGAAGAGGATTGAACAATGCAATATGTGGAGAGCTATCAATTTTACCTGGATGTATCTGAGCACTCTCAGTCTCTGGTTTGCCACTTGGCTCGCTGTCTTCTACTGTGTGAAGATCACCAGCTTCAACCAACCCCTTTTCCTCTGGCTGAAGCTGAGATTTTCAGGGCTACTTCCATGGCTTATTCTGGGCTCCTTGCTGGTCTCTTTGGCCACCTCTCTCCCTTCAGTCAATGTAATACATATAAATTACTTAAACAATTCAATTAATAATTTGTCCAGAAACATCACAGTGGTATGTCTATATAAGACTAACACATCTCTTAGTTCCTTAATTTTGACCGTGCTTGGACAGTACTCTCCTTTTGTTTTATTTTTTGTTCCATCTCTGTTGCTAGTCACTTCTCTGTTGAGACATACAAAAAGAATGGGAGAAAACATGAGCACTTCTAGGGACATCAGTGCAGAGGCTCATATCAGAGCAATTAAAGCTCTCCTCTCTTTCATTTTCCTGTACATTTTCTATATGTTGGCACAGTTTTTTACATTGACAAATAAGTTCGCTACCAGCAGCCCCTACCTTATATGGCTCTGTATTATGATACTAGGTGGATATCCCTCTGGGCATTCTGTTATCCTGATTCTCAGCAATCCCAAACTGAAAGAAGCAGCACTAAAGGGCTTGCACAATGCCAGGTGTCTGCAGGAAGATGAGAGTCAGTGA

>American_alligator_Tas2r2

ATGGAAGATAACAGAGGCAACATTTCAGAAGGCAATATCTGGGAGCCAGATATGTCTGTCCTAATAACTGTACTCTTTGAGACTTTTCTCGGGATCTCACTAAATGCTTTCATTATAGCTGTGAACTGCATTGATTGGGTCAAAAAGAGACGCCTTTCCACAAGTGATCAGCTTTTGACCATTCTCAGTTTCAGCAGGATTTGCTTATTGCTCTCAGAAGATGCAGAGTTTGTTTCTTCAACATTTAATCCTTCGTTTTATAACAGCAAGTCCGGATTCTTAATGTTTGCAGGTCTGGCTTGGTTCCTGAGCACATCCAGCCTGTTCTTCGCTGCCTGCTTGTCTGTTTATTACTGTGTGAAGATTGCAAACTTCAGCTGCCGTTTCTTCATCACGCTGAAACGAAAAATCTCCCAGCTCATGCCATGGTTGCTGCTGGTCTCAGTGATGATTTCCTTACTCAGCAACCTCCCTATCTTCATTGCCATTTATAACGTCTCAGATAATAGCTGCAACTCCAGCTGCTCACAAAACCACACAGGAGACAATGTCACACAAAAAAAATTGTTTATGGAGATGATTATTGTCTTTTCCTTTGGCTCTTCTATAGGTTTCACCATATTATGCATCTCAGCTGTTCTCTTACTCTTCTCACTCTGGAGACACATCCGACACATGAAAGGGAGCTCAGCTGGTGTCGGGAAGCCTAGCATGGAGGCACATGTGAAAGCTGTGAAAATGGTAATGTGGGTCTTATTCATCAATCTTATTCACTTCCTAGTTTGGTTAAGCTTCATTACACTTGCATTTTCACCAAATATTTTTGTGCAACATTTTCTTACTCAGGTTACAATTTTTTGTCCATCAATACATGCTCTTGTACTGATTCTCAGCATTCCCAAATTGAAACAGGCACTGGCCAGGATCCTGCACTACGTAAAATGCAAGGGCTGTGCAAAAGAGGGAATACCTTAA

>American_alligator_Tas2r3

ATGGAAGATAACAGAGGCAACATTTCAGAAGGCAATATCTGGGAGCCAGATATGTCTGTCCTAATAACTGTACTCTTTGAGACTTTTCTCGGGATCTCACTAAATGCTTTCATTATAGCTGTGAACTGCATTGACTGGGTCAAAAAGAGATGCCTTTCCACAAGTGATCAGCTTTTGACCATTCTCAGTTTCAGCAGGATTTCCCTATTGCTCTCAGAAGATGCAGACTTTGTTTCTTCAACATTTAATCCTTCGTTTTATTACAGCAAGTCCGGATACTTCAAGTTTACAGGTGTGGCTTGGTTTCTGAGCACATCCAGCCTGTTCTTCGCTGCCTGCTTGTCTGTTTATTACTGTGTGAAGATTGCAAACTTCAGCTACCGTTTCTTCATCACGCTGAAACGAAAAATCTCCCAGCTCATGCCATGGTTGTTGCTGGTCTCAGTGATGATTTCCTTACTCAACAGCCTCCCTATCTTCATCGCCATTTATAACATCTCAGATAATAGCTGCAACTCCAGCTGCTCACAAAACCACACAGGAGACAATGTCACAGGAGACACAATTCTTCTGAACACGATTATTGTCTTTTGCTTTGGCCTTTCTATAGGTTTCACCATATTATGCATCTCAGCTGTCCTCTTAATCTTCTCACTCTGGAGACACATCCGACACATGCAAGGGAGCTCAGCTGGTGTGGGGAAGCCTAGCATGGAGGCACATGTGAAAGCAGTGAAAATGGTAATGTGGGTCTTATTCATCAATCTTATTCACTTCCTCGCTTGGTTAAGCTTCATTACATTTGCAATTTCACCAAATATTTTTGTGCAACGTTTTCTTATTCAGGTTACAATTTTTTGTCCATCGATACATGCTCTTGTACTGATTCTCAGCATTCCCAAATTGAAACAGGCACTGGCCAGGATCCTGCACTATGTAAAATGCAAGGGCTGTGCAAAAGAGGGACTACCTTAA

>American_alligator_Tas2r4

ATGGTGGATAAAAGCTGTGCCTTCCTAAGTGCTATAGAGCTATCACCAATCATTCTCTTTTATCTGAGCATTGTGGCAATTGAATCTGTGACAGGGATTGTTGGAAATGGATTTATTTTGGTCATCAACTTAGCCAGCTGGGTCAGGAACAGAGTAGTGTCTTCCTGTGATATGATTCTGATCTTCCTGAGCTTCTCCAGGCTCTGTCTACAGTCCTGCATGCTGATGGATTTTGTCTGCAACTTGTTTTATCCATCTTTTTATAATCAGGAAGATGTATATGAAAACTTCAAGGCTATCTTCGTGTTCCTGAACAACTCCAGCCTTTGGTTTGCCACCTGGCTTGGTGTGTTTTACTGTGCCAAGATTGCCAACTTCAACCACTCCTCCTTTCTCTGGCTTAAACAAAACATTTCCAGCCTGGTGCCATGGCTATTAGCAGGATCATTGCTGTTCTCCTTTGGAAGCAGCCTTTCTTTCTACTGGGATATCTACAAGGTGTACTGCAACTACTCCACTGCCTTCCCCTTGGAAAACACCACAGAGCTGAAAGTGATAAAGAACACTAATTTGTTTTATGTGATTTTTCTCTGCAATGCCAGTCTATCCCTGCCTACAATAGTGTTTGTTTCCTCCATTGTACTGCTGATTTCCTCCTTGTGGAGACATACCAAACGAATGCAAAATAATGGAACTGGTCTCAGAGATCCCAGCACAGAAGCTCACAGAGGCGCCATCAAATCTGTCTTTTCTTTCTTAATCCTATACTTTTTCAATTTAATTGCTTTAATTCTCACCTTGTCCAACATCTTCTCAGCTTATGGCACCTGGGATATCCTATGCATGCTTGTGATGAGTGCCTACCCCATGGTGCACTCTGTAATCTTGATTCTGGGCAATCCCAAATTAAGAAGGGTATCACTTAATTTTCTTCACTATGCCAGTTGTCATTTTAGAGGAGGACCTAAGTGA

>American_alligator_Tas2r5

ATGACTGTGAGAAGAAATATGTTCAGTTCTGTTTCCATCATTGCTATAACCATTGTGTTGATTGAGATCAGTGTAGGATTACTGGGAAATGGATTTATTGTGGCCATTAACTGGACTGACTGGATCAAAAGCAGGAAGCTGTCCTCCTGTAACACAATCTTGACATCTTTGGGCATCTCTAGACTTCTTTTGCAAGGGACAGCAATAGTGTTTCGCTCCTATTCTTTCTTCACTCTGGACACGCATAAACTGGACAATGTGCGTATCACATTGCGAGTCATACGGATGTTTGCCAACATGACCAGTGCTTGGCTTGCCAGCTGTCTCAGTGTTTTCTACTGTGCAAAGATCGCCACTTTCACTCACCCTCTCTTCCTTCGAGCGAAGCAGAGAATCTCTGGGATGGTTCCACAGCTTCTCCTGGGGTCGCTACTGCTTGCCTTATTCACATCCATACCAACAGTCTGGGCTAATCATGATGTTTACCTTTGTAACTCCAAGGGGAGCCTTCTGGGAAACACCACATCAGCCAAGGTTAACTCAAACGTCATATATCTTTACTTCAGCTTTCTTTACACCGTAATGGCTTTCTTTCCTTTCCTCATATTCTTGGCATCATCCATGCTACTGATGGTCTCTTTGTGGAGACACAGCAGGTGCATGCAGGATTATGCACCTGACCTTCAGGACTCCAGGACACGGGCTCATGTAAGTGCGATTAAATCTCTGATCTCTTTCCTCATCCTTTATACTTTCAGTTTTGTAGGAGAGACATTGCAAACAATGCCTACTTGTCTGACTGACAACACCTGGACACCTGCAGTTACTTCTGTGGTGGTTGCTGCATACCCTTCAGGGCACTCCATTGTTCTCATTCTTCTTAATCCCAGGCTGAAAACAGCATTGGTGCAGATTCTTCGCCACATCAAGTGCCAGCTGAGACTTTCTTAA

>American_alligator_Tas2r6

ATGTTGCCTCCACTTCTTATACTATTATTCACCATTTTAGGGATTGAGATCATTGCTGGCTTTATGGGAAATGGATTTATTGCAGCCCTAATTGGCAGTGATTGGATCAGAAATAGAAAAATCTCTTCTTCTGACATGATCCTGATCTCTTTGGGCATATCGAGATTTGTCTTGCAGGGGACCATAATAGTGTACATCCACAGTCTCTACTTTCCAGGCATGCCCAAGCTGGCTACTTTATATAAAGCATTCTGTATCTTGTGGATGTTCGTAAACCATGCCAGTCTGTGGTTCAGCACCTGGCTCAGTGTCTTCTACTGTGTGAAGATTATCAACTTTACGCAACTGATTTTACTGCGAATGAAGCTGAGAATTTCTGGGATGGTTCCCTGGTTTCTTCTGGGGTCAGTGCTGGTCTCTTCTATCACCACATTGCCAATGTTTTGGATTTTTCCAAGCATTTCCTCCCACAACTCTACTGGAAATCATGTAAACAACTCTGTGAAGACCACTGCTTTGGACACCTCATCGCTCAGCATTGCTTCTCTTTATTGCGCAGGTTGTTTCTTTCCTCTTACAATATCATTCTTCACTTCAGTCCTGTTGATTGTTTCTCTGTGGAAGCACACCAAAAAGATGCAACACAACACAACCAGCTGCCAAGATCCCAGGACAAACGTTCATGCAAATGCCATTAAAGCTCTGGTGTCTTTCCTTATCCTCTATCTTTCCAGTTTCATCGCTCAAATCCCTTTGATACTGTTAGCTTCGCAAAATAGCCATATCTGGGAAGTTGCAGTCTCATTAGTAGTGGTTGCTGCATATCCATCAGGACACTCCATTATCTTGATCTTAATTAATTCCAAACTGAAACAGGCATCAGTGAGATTGTTCAACTATACAATGTACCCTCTGACAAAAGGGACTCCTTAA

>American_alligator_Tas2r7

ATGTCATCACTCTCTGTCATTATTTTTATTATTTTATATGGTGTTCAGTTCTTTGTAGGCATTATCACTAATATATTTATAGTGACTGTGAACGTGATTGACTGGACTAAAGACATTAAGTTGTCTTCAAATGATCAAATTCTGGTATATTTGGGACTGTCTAATCTTTTTGTGCAGTGCACAGCCACAGCTGCTGACTTCTGCTTTTTTTTCTGGACAGACCTACTCTACTCAGGTTTCAGCTCGCAAACTTTTTTCTTTTTTGTATTTTTTGGCAGTATCTGTGCTTCCTGCTTTACTGGATATCTATGTACTTGCTATTATGTGAAGATAACTGACAGTACTTATCCTCTTTACCTGAGGATGAAGATGGCATTTATCAAGAACTTACCCTGGCTTCTCCCATGGATCATTGCAACAAGCTTTGGATTGAGTCTGGCTGCAGTTTGGGATGCATCTAAGAAAGTTTCCCTTGACATGACAGCTAATTTCAGCACCAATTATACCAAACCATTGCTTTTGTTTCATTACTCCACTGCCTTCCGCATCATCCTCCTTTTGCTGGAATGTATTTGGCCTCTTATAGTAACATCATTTTTAGTCCTGAAGCTCATCAAGACCCTCTGCAAGCACATCAGGAACATGGAGAGGACCATGGCCTTTGGCCAACCTAATCTGGATGCCCACAAACATGCCACCCGGACCCTGACATCTCTCCTTATCCTTTTTATTTCCTACAACCTGCTTTGGAGTATACTCGTCTATGATATTTTTTCATATCCCAGCACTGGGTTTTTAATTTGCATCACTTTGTTTGCCACACTCACATCCGTACAGGCCATAACCTTGATCCTGTGTAACCGAAGAATGAAGCAGAAAGCCTTGAGGATCCTACAGAGCATTAGACAATTTTCAGGTGGATAG

>American_alligator_Tas2r8

ATGGAAGGCAACAGAAGCAATATTACAGAAGAGGACGTGGCTAGACCAAGTATATCTCTCGTAATAATCTTGCTCTTTACGGCTTTTGTTGGGATCTCAGTCAATACTTTCATTGTGGCAGTGAATTGCACTGATTGGGTCAGAAGGAAGCGCCTCTCCACAATTGATCAGATCCTGACGATTCTTGGCTTCACCAGGTTTTCCTTGTCGTGCATAGCAGTAGTAGACAGTTTTCGTCAACCATTTCATTCTTGGCCCTATGGATTTATAGACAAAACATTCAGTATTTTAAACTGGTTTCTAAATGTTTTGAACCTGTGGTTTGGTGTCTGTCTGGGTATTTTTTATTGTGTGAAAATTGCAAACTTCAGTCACCATTTCTGCATTTCTCTGAAACTAAAGATCTCCAGGCTCATGCCATGGTTGCTGATGGCTTCCGTGCTTCTCGCCATTTTTAACACCTTCCCTGCAGTCACGTTTATTTTTAAAACTCAATTTAAGAAATCCAACTCCAGCATCCCAGAAAATAACAAAGGAGAAGATATACCACAAATAATATATTTTCTTCATCTGTTTTCTCTCTTCGTCGTTGGATTTCCTCTGTGTTTCACCATACTATGTATTTCAACCTTTCTCTTACTCTTGTCTCTCTGGAGACACACCCGGCAGCTTAATTCAAGCAGCAACAACAACCCTAGCATGCATGCACATATTCTAGCAGTAAAGATAATAATGTCCTTCTTTATCATCCATGTCATCCACTTCTCAGCTTGGTTAATCGTGTTGGCAGCAAGTATTCCAAATAGAAAATTACAGCGGTTATTTTTGTTTCAGATCGCAAATAGTTGTCCATTGACACATTCTGTTTTACTGATTCTGAGCTATCCCAAACTGAAACAGGCATTAACCAGGATTCTGCATTACATGGGTTGTGCTAAAGGAGTGTCTTAA

>American_alligator_Tas2r9

ATGGAAGGTAACAGAGGCAACATTTCAAAAGGCAATACCTGGGAGCTAGATCTCTCTCTCCTAATAGCTGTACTCTTTGAGACTTTTCTCGGGATCTCACTAAATGCTTTCATTATAGCTGTGAACTGCATTGATTGGGTCAAAAAGAGATGCCTTTCCACAAGTGATCAGCTTTTGACCATTCTCAGTTTCAGCAGGATTTGCCTATTGCTCTCAGAAGATGCAGACTTTGTTTCTTCAACATTTAATCCTTCGTTTTATCACACCAAGTCTACACGCCTAATGCTTGCAGGTGTGGCTTGGTTCCTGAGCACATCCACCCTGTTCTTCGCTGCCTGCTTATCTGTTTATTACTGTGTGAAGATTGCAAACTTCAGCTGCCGTTTCTTCATCACGCTGAAACGAAAAATCTCCCAGCTCATGCCATGGTTGCTGCTGGTTTCAGTGATGATTTCCTTACTCAACAGCCTCCCTATCTTCATCGCCATTTATAATGTCTCAGATAATAGCTGCAACTCCAGCTGCTCACAAAACCACACAGGAGACAATGTCACAGAAGACAGAATTCTTTTGGACATAATTTTTGGCTATTCCTATGGCATTTCTATAGGTTTCACCATATTATGCATCTCAGCTGTTCTCTTACTCTTCTCACTCTGGAGACACATCCGACACATGCAAGGGAGCTCAGCTGGTGCCGGGAAGCCTAGCATGGAGGCACATAAGAAAGCTGTGAAAATTGTAATGTGGGTCTTATTCCTCAATGTTATTCACTTCCTAGTTTGGTTAAGCTTCATTACATTTGCAGTTTCACCAAATATTTTTGTGAAACATTTTCTTATTCAGGTTACAATTTTTTGTCCATCGATACATGCTCTTGTACTGATTCTCAGCATTCCCAAATTGAAACAGGCACTGGCCAGGATCCTGCACTATGTAAAATGCAAGGGCTGTGTAAAAAAGGGAATACCTTAA

>Green_Anole_Tas2r1

ATGGCTGCTTACCCAATGTATCCATTTGCTATCTTTTCCTGGAGTATTATCGGTATTTTGTGGATTGTTTCCCTTTCTGGGAATGGATTTATCTTCACTGTGACTGTGCTGCAATGGCTCCAGAAGAGGAAGATGCCACCTTGTGACTTCCTCCTGTCTTGTTTGAGTGCCTCCAGATTGCTAACACAGTTTAATTATATGGCCAGCTATTTTTTGCCTTTCTTTTATTCGCCAAGTATAAGAAAAATGTTTTTTTTCTCCAGGGTCTTTCTTCATATGGCCAGTCTTTGGTGTGTCTCCTGGCTCAGCATTTTCTACTGTGTGAAAGTCATCAACTTTTCCAGCTCTTTACTCCTTTGGCTAAAGTTAAGGATCAATCTGCTTGTACCCAAACTACTTGGAATATCAATGGTCATTTTCATGGTCTTTTCTCTTCCTTCCATCTTCACATTTCATAAATTCAATAAACCATGTAATCAGACGATAACACCACCAACCAGCCATGAACCTGAAGATAGCATGTGGATTCGTTTTTTTCCAGTGCAGATAACTTTCACTTGCATAAATTTCAGCATGAACATAGCAGCAACCCTTCTTTTGCTCATCTCTTTGTGGAGACATGTGAGAAACCTCAGAAAGAGTGGAACTAGTGTTCAGGACCTCAACACTCAGGTCCACCTCAAAGTCATGAGGCTTTTGTTCATCACTCTCTTACTCTACCTTTTATTTATTGCTTGTTCGATAACAATGACAACTGGCTTTTTTCATGTTCAAGAAAACCAAGCACTGATTTCAGAGATAATGATTTCCATATTTCCTTCAGTGCATCCCATAATATTAATATGGACCAATCCAAAACTCAAAGATGTGGCTGCTCACATGTTAAACATAAGAGAAAGACCTTAA

>Green_Anole_Tas2r2

ATGGATAATAACTTAATTTCTCCATTGGGTATTTTTACATGGACCATTATAGAAGGTATATCCATGGTTGCCATTTTAGGAAATGGATTTATCATAGTTGTGAGTGGGAACCGATGGCTCCAAACCAGGAAGATGGTCCCTTCTGATTTTCTCTTGACTAGTTTGAGCATCTCCAGAGTGTTTTGGCATGTAACCTTTGGACTTAGCTATGTTTTGGAGGTCAGCATTGGTGACATCTTTATGTATTCTTCTGCACAGGAAGCTATCGACTTTATCAGCACGTTTTCTAGCATGGCCAGCCTCTGGTGTGCTTCATGGCTTAGTGTTTTCTACTGTGTGAAGGTCACCAACTTTGCCAACCGCTTTTTACTCTGGCTGAAGCCAAGGATCAATGTGCTCTCAGTTAGACTGCTTGGAATGTCAATAAGTAGTCTTGTGTTCATGTCTGTCCCCTTCTTCCAGCATTACGCTGAAGCAAAAAAGCGGTGCAATCTGACTGGGAGCCTGCCACTGAACACCAGCCAAAGAAACGATTGCAAATTCTTACTTTTAATTTTTCGTCATTTTCAGGTAATTGTTGCTACCATGAATTTCGTCATCAGCATAACTGCAACCATTCTTTTGCTCACCTCTCTGTGGAAACACACCAGGAATCTGAAAAAGAGTGGTATTGATGCAAAAGACCTAAGTGCTCAGATCCATATTAATGTCATGAAGCCGTTGGTGCTTTATATTTTCCTCTACCTTTCATATTTTGCTGGTATTCTAAATTTTGCAAGTCATTCTGTGCACAATGTTGATGCTGTGGAGCTTTTGTCTGATGTCCTTCGTACCATATTTCCTGCAGCACATACCATAACATTAGTTTTGAGCAATCCAAAACTGAAAGCACTGTTAGTTCGTACTCTAAATATAAGACAAAAGGTTAATCTTGTCTCCAAGGACGAAAAGACAAACCAAATTTCCAAATGTTGA

>Green_Anole_Tas2r3

ATGGCTGCTAACCTAGTGTCTCCATTTGCTATAATCTCCTGGAGCATTATAGGCATTTTGTGCATTGTTTCCCTTTCTGGGAATGGATTTATCTTCATTGTGACTGTGCTGCAATGGCTCCAGAAGAGGAAGATGCCACCTTGTGACTTCCTCCTGACCTGTTTGAGTGCCTCCAGATTGCTAACACAGTTGAATGCTATGGCCATCTATTTTATGGAACTCTTTTATCCTTCAAGTAGAAGTGCAATGCTCTTTTTCTCCTGGGTCCTTCTCAATATGGCCAGTCTTTGGTGTGTCTCCTGGCTCAGCATTTTCTACTGTGTGAAGGTCATCAACTTTTCCAACTCTTTACTCCTTTGGCTCAAGTTAAGGATCAATCTGCTTCTACCCAAACTACTGGGAATATCAGTGGTCATTTTCATGGTCTCTTCTCTTCCTTCCATCTTCACATTTCATAAATGCAATGAACCATGTAATCAGACTGTAACACCCCTAATTAACGAGAAACCTGGCAACATGTGGATCAGTTTTTTCCCAGTGGAGATAACATTTACTTGCATAAATTTCAGCATGAACATAGCGGCAACCCTTCTTTTGCTCATCTCCTTGTGGAGGCATGTGAGAAATCTCAGAAAGAATGGTACTAGTGTTCGGGACCTCAACACTCAGGTCCACCTCAAAGTCATGAGGCCTTTGTTGATCACTCTCTTACTCTATCTTTTATTTATTGCTAGTTTGATAACTATGGCAACTGGCTTTTTAAATTTGCAAACAAAACAAGCACTGATTGGAGAGATAATGGTTACCATATTTCCTTCAGTGCATGCCATAATATTAATATGGACCAATCCAAAACTCAGAGAAGTGGCTGCTCACATGCTAAACATCAGACAAAGTGCTTAA

>Green_Anole_Tas2r4

ATGGTTAGCATTCCAACTTCTCCAGTTGATATCCTTAGATGGACCATTCTGGGAATTGTATCCTTGTTTACTCTTTTAGGAAATGGATTCATCATAGTTGTACTGGGATATCAAGGGCTTCAAAAGAAAAATATATTGCCTCATGATATCCTCTTGATAGGTTTGAGCGCCTCCAGGATAATGTTGCAGTTGCTGAGTTCAGCAAACTATATTTTGTGTTTCATCTCAGAGACCTACAGGGATACTTACAAACAGGATGTTGTACTTCTTTCCTGGAACGTTTTCAACATGACTAACTTGTGGTCTTCAACCTGGCTAAGTGTTTTATACTGTGTGAAGGTCACTAACTTTGCCAATTGCCTCTTCCTCTGGCTGAAGCCAAGGATCAACATGCTTGTACTCAGGCTGCTTGGGATGTCAATAGTGATTTCCATTATCTTCTCTGTTCCTTCGGTCATTAAATACCTTCAACAGAAAAAGTGGGACAATCTGACTAGAAACTTGTCAGTGAGTGCCATCCAATGTATGGATTATAAGAACAGATTCATAATTTTTCTGGATATGCAGCTCTTTTATGTTTCCATAACTTTCTGCATAAGTTTAATTGCCTCCACTCTTTTGCTTGTCTCACTGTGGAAGCACATTAGGAATCTGAAGAAGAGTGGCCTAGGTGGCAAAGATCTCAGCACTCAGGTCCATATAAATGTCATTACACTGTTGCTGTCTTATATCTTCTTCTATCTTTTATATTTCACTGGTTTCATAATTTTGGGAACTAATGTTTTCAACTATGAAAGTCTTGAAAGGTTAATTTTTAAGTTCCTGGCAATTTCATTTCCTTGTGTACACTGCATTATGTTAATTTTGACTAATCCCAAACTAAAAGAAATGGCTGGTCATATTCTGAATATCACACGAAGAGCTTCCTAA

>Green_Anole_Tas2r5

ATGGCTGCTAACCTAGTGTCTCCATTTGCTATAATCTCCTGGAGCATTATAGGCATTTTGTGCATTGTTTCCCTTTCTGGGAATGGATTTATCTTCACGGTGACTGTGCTGCAATGGCTCCAGAAGAGGAAGATGCCACCTTGTGACTTCCTCCTGACTTGTTTGAGTGCCTCCAGATTACTAACACAGTTGAATGCTATGGCCATCTATTTTATGCAACTCTTTTATCCTTCAGCTAGAAGTGCAATGCTCTTTTTCTCCTGGGTCTTTCTCAATATGGCCAGTCTTTGGTATGTCTCCTGGCTCAGCATTTTCTACTGTGTGAAGGTCATCAACTTTTCCAACTCTTTACTCCTTTGGCTCAAGTTAAGGATCAATCTGCTTCTACCCAAACTACTTGGAATATCAGTGGTCATTTTCATGGTCTCTTCTCTTCCTTCCATCTTCACATTTCATAAATGCAATGAACCATGTAATCAGACTGTAACACCCCTAATTAATGAGAAAGCTGACAACATGTGGATTAGTTATTTCCCAGTGGAGATAACATTTACTTGCATAAATTACAGCGTGAACATAGCAGCAACCCTTCTTTTGCTCATCTCTTTGTGGAGACATGTGAGAAACCTCAGAAAGAATGATACTAGTGTTCAGGACCTCAACACTCAGGTCCACCTCAAAGTCATAAGGCCTTTGTTGATCACTCTCTTACTCTACCTTTTATATATTGCTAGTTTGATATTAATGGATACTGGCTTTTTTTATTTTCAAGCAAACCGATCACTGATTGGAGAGATAATGGTTACCATATTTCCTTCAGTGCATGCCATAATATTAATATGGACCAATCCAAAACTCAGAGAAGTGGCTGCTCACATGCTAAACATCAGACAAAGAGCTTAA

>Green_Anole_Tas2r6

ATGTTTTCTCTCCAAATTACTGCCTTTCTCGTAGTTGCAGCTGACTTGACTCTGGGTGGACTCATTTCCAATGGCTTTATAGCTACAGTGATTATACGAAAATGGATCAAATGCAGAAGCCTTGCTTCCAGTGAACAGCTCCTTCTGGTTCTGGGAATATCCAACGTTTTTGCCATCATTTTACAGACTGCATCTGTAATTGGTGAAAATGTGTTTATCTGCAGCGACCAGTTGATATTACCAATAATCTTTTTCTTTGTTTTCTTTGTCACATTCTTCAGATTTTGGCTTACTGCCTGGCTCTCTCTCTTCTATTGCATCAAGATAGTGAACAGCACCCATGTCCTGTTGGTTTGGTGCAAGATGAGGATATCTTGGCTAATACACCGTCTCCTGTTAGGATCCCTACTCATTTCCTTGTTCATTTCCTTTTTTGCATTTCATGAGTTTCTTTTTGAATTCCAGAGCAATAGAACAGCCAGCGTTGCAAACAGGACTCAAGAACAGACATTAAGAAAGACTGTTGATTATTTCAAAGTTTTATTTTTGGCTATCGGTACTTCTTGTCCCCTTCTTGTGGTTTTATTTTGTTCCATCTTGTCTATTGTGTCACTCTGCAGACATATCCACAGGATGACAAGAGAAAAGTCAAGTTTTAGGAGCATCCAAGCAGAAGCTCATCTCAAGGCAGCTCAGACAATGCTCTCTCTCTTATTCTTTTATGTACTGTTTTATGTGGGGGAAACATTAAGTATGACCATACATTTTGAGAATGGGAAACAAATTTCTGCTATATTTGTGGTGCTGCTGTACTCTCATGCTCAGGCTGCCATTCTGGTGCTGGTAAATTCCAAGCTAAAGAGGACAGCTACCCAGATTCTTCTAAGAATTTCTCAGGAACTATGCAGACATACTGTTTGTTCACAGATTTAA

>Green_Anole_Tas2r7

ATGAGAAGACAAATTACACAGCGATACATGATTTCAGACATTTTTTTTTTCTATAAAATGACAACAATGGTTAACAATTCAATTTCTCCATTTGATATTCTTAAATGGACCATCCTGGGAATTATATCTGTGTTTACTCTTTTAGGAAATGGATTCATCATAGTTGTACTGGGATATCAAGGGCTTCAAAAGAGAAATATATTGCCTCATGATATCCTCTTGATAGGTTTGAGTGCCTCCAGACTAATGTCGCAGATGCTGAGTTCCACAAGCTATCTTCTGTATTTCGAGAACGACATGGGTATTTTTAACCAGGATGTTGTACTTATTTGCTGGAACTTTTTCAACATGACTAGCATGTGGTCTGCAACCTGGCTTAGTGTTTTATACTGTGTGAAGGTCACTAACTTTGCCAACTGCCTCTTCCTCTGGCTGAAGCCAAGGATCAACATGCTTGTACTCAGGCTGCTTGCGATGTCAGTAGTCATTTCCAGTATCTTCTTTGTTCCTTCAGTCCTTGAATACTTTCAACAGAAAAAGTGGGACAATCTGACTAGAAACTCACCCGTGAGTGCCAATCAAAGTGAGGGTTATAATAATGGATTCATTATCCTTCTTGATATGCAGCTCTTTTATGTTTCCATAACTTTCTGCATAAGTGTAATTGCATCCACGCTTTTGCTTGTCTCACTGTGGAAGCACATTAGGAATCTGAAGAAGAGTGGCCTAGGTGGCAAAGATCTTAGCACTCAGGTCCATATGAATGTCATTACACTATTGCTGTCTTATATAATTTTCTATATTTTACATTTCACTGGTTTCATAATTTTGATAAGTGATGTTTCCAGATTTAGAAGTGTTGCCACGCTAGTTACTTCCATCCTGATAACTTCATTTCCTTGTGTACACAGCATTATGTTAATATTGACTAATCCCAAACTAAAAGTAATGGCTGGTCATATTCTGGGTATCATGCGAAGAGCTTCCTAA

>Green_Anole_Tas2r8

ATGAGAATTTTATTCTTCAGGCACCACAACGTAATTCCTCTCAGACATTTCTCCCAGAAAGTAACAGCAATGGCTCTCATAATGTCTCCACTTGGCGTTCTTGCTTGGGCTACTTTTGGAATTTTGAATGTCATGGCCCTTTTAGGAAATGGATTTATCATAGTGGTGAATGGACATCAGTGGCTCCAGAGCAAGAAGATGATCCCTTACAAGTTCCTCTTGACTACTTTGAGCACCTCCAGGTTTCTGTTGCAGATGGATTCCGTAGTGGGCCATTTTATGTATCTTATCTTTGCAGAGATCCAGAAAGAGACCCATCTATATGCTTCCAGAGCAGAGGTTGTGAATTTTATCTGGATGTTCTTGAACATGGTCAGCCTCTGGAATGCCTCGTGGCTCAGTATGTTGTATTGTGTGAAGGTTACCAATTTTGCCAACAGGCTCTTCATCTGGCTGAAGGCAAGGGTCAATATGCTTGTACCCAGACTACTTGGAATCTCAATAATAGTATCTACAGTTTTCTTTTTTCCATCAGCAGCCAAGTATTATAGAAAGAAAAAGTGGTGCAATTTAACAGATGCCGTCCCGAGGAACAGTAGCCAAAGGGAGGGTTGTAATGATGCTTTCGATGTTTTTCATTTTCCGCAGTTATTTCTTGCTTCAGTTAATTTCGGCTTGACCCTCACTGCATCCTGTCTTTTGCTCACTTCTCTGTGGAAACACACAAATAATCTGGAAAAAAGTGGTGCTGCTTTTAAGGATCTTAGCACTCAGCTCCATTTCAAAGTTATGATGCCTTTGCTGGTCTCTTTATTGTTCTATGTTTTATATTTTCCTTGTTTCGTGTTAGCTGTAGGTGATATTTTTGAATTTGGAAGACTTGAGCGATGGGCTTCTGAGATAGTACTGCCTCTGTATGCTTTCGTACAGTCCATAATATTAATACTGACCAATCCTGAACTAAAAAAAGTAGCTGCTAGCATTCTAATAATCAGACAACGAGCTTCATGA

>Green_Anole_Tas2r9

ATGGATAATAACTTAATTTCTCCATTGGGCATCTTTAGATGGACCATGGTAGGAAGTATATCCATGGTTTCCATTTTAGGAAATGGATTTATCATAATTGTGAGTGGGAACCGATGGCTCCAAAACAGGAAGATGGCCGCTTCTGATTTACTCTTGACTAGTTTGAGTATCTCCAGAATTTTTTTGCATGTAACCTTTGGACTTTACTATGTTTTAGAAGTCAGCATTGGTGACACCTATATGTGTACTTTTGCCTATGATGCTGTCATCTTTGCCTGTATGTTTTCTAGCATGGCCAGCCTCTGGTGTGCTTCATGGCTTAGTGTTTTCTATTGTGTAAAGGTCACCAACTTTGCCAACTGCTTTTTACTTTGGCTGAAGCCAAGGATCAATGTGCTCTCAATTAGACTGCTTGGAATGTCAGTAATTAGTCTTATGGTCATCTTTGTTCCCTTCTTCTGGAGTTACACTGAAGACAAAAAGCGGCGCAATCTGACAGGGAGCCTGCCAGTGAACATCAGCCAAAGAATGGACTGCAAAGCCTTATTTTTTATTTTTTATCCTTTTCAGTTAAGTGTTTTATCCATGAATTTCATCATCACCATAACTGCAAACGTTCTTTTGATCACCTCTTTGTGGAAACACACACAGAATCTGAAAAAGAGTGGTATTGTTGCAAAAGACCTAAGTACTCAGATCCATATTACTATCATGAAACCTTTGTTGTGTTATATTTTGCTCTACCTTTTATTTTTCACAGGTATGCTAATTTTTTTAGGCAGTTTTGCGTACACTTTTAATGGTAAGGGTTTTTTATCTGACATCATTTTTACCACATTTCCTTCAGCACACACCATAATATTAATCTTGACCAACCCAAAACTGAAAGCCCTGTTAATTTGTCATTTAAATATAAGATCAAAGGCTTAA

>Green_Anole_Tas2r10

ATGTCTCCTCCATTTGGTATCTTCTCCTGGAGCATTCTAGGTATTTTGTGGATTGTTTCCCTTTCAGGGAATGGATTTATCTTCACTGTGACTGTGCGGCAATGGCTCAAGAAGAGGAAGATGCCACCTTCTGAATTCCTTCTGACCTGTTTGAGTGCCTCCAGATTACTAACACTGTTGACTACTATGGTCAGTTATTTGCCTCTCTTTTATTCTTCAGGTAGAAGTGCAATGCTCCTTATTCCCTGGGTCTTTCTCAATATGGCCAGTCTCTGGTGTGTCTCCTGGCTCAGCATTTTCTACTGTGTGAAGGTCATCAACTTTTCCAACTCTTTACTCCTTTGGCTAAAGTTAAGGCTCAATCTGCTTCTACCCAAACTACTTGGAATATCAATGGTCATTTCCATGGTCTCTTTTCTTCCTTTCATCTTCATATTTCATCAATGCAACAAGCCATGTAATCAGACTGTAACACCACCAATCAACCATGAAGATGAAGCTGATGACAGCATGTGGATTCGTTTTATTCCACTGCAGATAACTTTTACTAGTATAAATTTCAGTGTGAACATAGCAGCAACCCTTCTTTTGCTCATCTCCTTGTGGAGGCATGTGAGAAACCTCAGAAAGAATGGTACTAGTGTTCAGGACCTCAACACTCAGGTCCACCTCAAAGTCATGAGGCCTTTGTTGATCACTCTCTTACTCTACCTTTTATATATTGCTAGTTTGATATTAATGCATACTGGCTTTTTTTATTTACAAGCAAACCTATCACTGATTGGAGAGATAATGATTACCATATTTCCTTCAGTGCATGCCATAATACTAATATGGACTAATCCAAAACTCAGAGGAGTGGCTGCTCACATGCTAAACATCAGACAAAGAGCTTAA

>Green_Anole_Tas2r11

ATGGATTGCAGCTCAATTTCTCCACTTCATATCCTTATGTGGAGCATCGCAATAACTGGAAACATAGTTGCTCTTTTAGGAAATGGATTTATCACAGTTGTTCAAGGTCACCAGTGGCTTCAAAAAAGAAAGATTTTGCCTTGTGATTTCATCTTAATTAATTTAAGTGCCTCCCGATTTATGATGCTGTTGTCGAATTCCGTGAACTATATCTTGTATTCCATCTCCTCGGAGAGTTATCTGCGTTCTTATAAAAAGGCATATCTAATGATTACCTGGACTTTCATGAATATGGCCAGTCTCTGGTCTGCCACATGGCTAAGTATTTTCTACTGTGTGAAGGTTGCTAACTTTACCAACTGCCTCTTTCTCTGGCTGAAGACGAGGATCAACATGCTTGTGCCCAGGCTGCTTGGAATGTCAATAGTCATTTCCAGCATCTTCTCTGTTCCTTCAGTCATTGAATATTTTGGACAAATAAGGGGTGGCAACTTGACGATAATCTTGCCGCTGAATGTCAGTCAAAATGAGCGTTATGCTAAAGGTTTATTTCCTCTGTATCTGACTTATACTTCCATAAATGTCTGCATAAGTATAATTGCATCCAGTCTTTTGCTTGCCTCCTTATGGAAACACACGAGGAATCTGAAAAAAAGTGGTCTAAGTGGTAAAGACCTCAGCACTCAGGTTCACAAGAATGTCATAATAGTGGTATTCTCTTATGTTTTTTTTTACCTTACATTTTTCACTGCTTTAATAATTGAGGTAACTAATGTTTTTAAGCCTCGAAGTCCTGAATCGTTAATAGTTGAAATTTTGGCAACTTCATTTCCTTCTACACACTGCATTGTATTAATATTGACTAATCCCAAACTGAAAGAAATGGCTGCTCGCATTCTGAATATTGGATAA

>Green_Anole_Tas2r12

ATGTCTCCATTTGCTATCATCTCCCTGAGCATTCTAGGTATTTTGTGGATTGTTGCCCTTTCAGGGAATGGATTTATCTTCACGGTGACTGTGCTGCAATGGCTCCAGAAGAGGAAGATGCCACCTTGTGAATTCCTTCTGACCTGTTTGAGTGCCTCCAGATTACTAACAGAATTCAATTCTATGGCCATCTATCTTTCACGTCTCTTTTATTCTTCAAGTAAAAGAGCAATGCTCCTTATTCCCTGGGTCTTTCTCAATATTGCCACTCTCTGGTGTGTCTCCTGGCTCAGCATTTTCTACTGTGTGAAGATCATCAACTTTTCCAACTCTTTACTCCTTTGGCTAAAATTAAGGATCAATCTGCTTTTACCCAAACTACTTGGAATATCAATGGTCATTTTCATGGTCTCTTCTCTTCCTTCCATCTTCACATTTTTTAATTACAAAGAACCATGTAATCAGACTGTAACACCACTATCTAACCAGGAAGCTGACCTCAGCATGTGGATTAGTTTTGCTCCACTGCAATTAACTTTCACTTGCATAAATTTCATCATGAACATAGCGGCAACCCTTCTTTTGCTCATCTCCTTGTGGAGACATGTGAGAAACCTCAGAAAGAGTGGAACTAGTGTTCAGGACCTCAACACTAAGGTCCACCTCAAAGTCATGAGGCCTTTGTTGATCACTCTCTTATTCTATCTTTTATTTATTGCTAATTTAATAGTAATGATAATTGACTTTTTTGATTTACAAACAAACTTGTCACTGATTGGAGAGATAATGGTGTCTGTATTTCCTTCAGCACATCCCATAATATTAATATGGACCAATCCAAAACTCAAAGAAGTGGCTGCTCACACTGCTCAACATCAGACAAAGAGCTTAAAAAAAGGAGATGGTGGCAACAGGTATCCACCCCCGTCTTCGAACAGAGATTGCTTCTATCAGTGTGCGAGGTAA

>Green_Anole_Tas2r13

ATGTTCTCTCTCGCAACCATTGCCTTTGCGGTAGCTGCAGTTGTCTTTGCTCTCAGTGGATTCCTCTCCAATGGCTTTATAGCTGCAGTAATACTCAGGGAATGGACCAAATCCAGGAGCCTTGCTTCCAATGAACAACTCCTTCTGAGCTTGGCTGCATCCAATTTCTGGGCCACAGCATTGCTGAGTCCATTTTACATCAATGCCACCTTAAGGGATTACAGCATCAGCGAGATTTTCCTGTTGCCAGGACTCTACCTTCTTGCTACTTTTGTCATAATGTCCAGATTCTGGTTCACTGCCTGGCTATGCTTCTTCTATTGCATCAAGATAGTAAACAGCACCCACTTTCTCTTCCTCTGGTGCAAACTGAGGATACTGTGGCTAATACCCAGGTTTCTAGCAGGATCTCTGTTCTGCTCTTTTCTTTTTTCTTTGTTTGTGTTACAGATTACTTCCAGACAAGCCAAAAGCAACATAACAGTGAATATTACAAACACAACTGAAGTGAAGTCACTGAAGCATACAGTCAATACCTTTGAAGCCTTCTTTTTAGCTGTTGGTTCTGGTTGTCCTCTTCTTGTGGTTCTACTTTGCTCCATCCTAGTCGTTGCCTCACTTTGCAGGCATGTCTGCCGGATTGCAGGTAAAGACTCTCATGGCAGGAATCTCCAAACCGAAGCTCACATCAAAGCAGCCTGGACAGTGCTCTCCCTCCTGCTCCTTTACGTATCATATTATGCAGCACAGACACTGTCTATTGTTGTGACATTGGGAAAAGATGACGGGACCTTGGTGGCAATGGTGAGAATGGTGTACCCCTCTGCCCAGGCTTCCATCTTGATGCTGGTTAACCCCAAGCTAAAGCAAGCAGCTATGCAGATGCTTCAAAGAGCCAAGGTGTGA

>Green_Anole_Tas2r14

ATGGCTAGCAACTCACCTTCTCCACTTGATATCCTTATCTGGACTATTATAGTAATCGAATACATTGTTTCTTTTCTAGGAAATGGATTTATCATGGTTGTTCATGGACATCAATGGCTTCAAAAAAGAAAGATGTTACCTTATGGTTTCCTCTTAATTAGTTTAAGCACCTCCAGATATATGATGCATCTGCAATCTTCACTGAACTATATTCTGCATATTGCCTTCTCCGAGCCCTGTATCGGTTTTTCTATTACAAAAATTGGAGATGTTAACTGGATCTTCTTCAACATGATCAGTGTTTGGGCTGACACATGGCTAAGTGTTTTATACTGTGTGAAGGTCACTAACTTTGCCAACTGCCTCTTCCTCTGGCTGAAGCCAAGGATCAACAATCTTATACCCAGACTGTTTGGAATGTCAATAGTCATTTCCAGTATATTCTCTGTTCCTTCAGTTATTTACTTTCTAGGACAAAAAAGCGGGGGCAATTTGACTGTAATCTTGCCACTGAATGTCAGCCAAAATGATCCTTGTAGTAAACACTTACTTCATCTGCAGCTGATTTATACTTCCATAACTTTCTGCACTAGTGTTATTGCATCCACTCTTTTGCTTGCCTCACTGTGGAAGCACACAAGGAATCTAAAAAAAAGTAGTCTGGGTGGTAAAGACCTCAGAACTCAAGTCCATATGAATGTCATAATACTGTTGCTGTCTTATGTTTTCTTCTACCTTGTATTTTTTACTGGTTTAATCCTTTTTAAAACTGATGTTATTAATATGACAATTCTCGAGATCTTGGGAACTTCATTTCCTTCTGCACACTCCATTATATTAATATTGACTAATCCCAAACTAAAGGCTGTGGCAGTTCGCATTCTGAATATCAGCCAAAGAGCTTCATAA

>Green_Anole_Tas2r15

ATGGCTAACAATATGTCTATGGTTGAAATCATGTTATTGATCATTTTTGAATTTGTGTCTATTATTGGTATTTTGGGAAATGGATTCATTATAGTTGTGAATGGATACCAATGGTTCCAAAACAGGAAGATGATTCCTTGTGATTTCCTCCTGACCAGCCTGAGTACATCCAGGTTTATCATGCAATTGGATTTTTTTATCTACTACATTTTGTACTTGACTCTAAAGATTAATTTCAAACTTTTCTTAGATGATTTTCTTTTCTTTTCCTGGATGTTTTTCAACATGATCAGCAACTGGTGTGCCACATGGCTCAGTGTTTTCTATTGTGTAAAGGTCGCCAACTTTGCCAACCCTCTCTTCCTTTGGCTGAAAGCAAGGATCAATATGCTTCTACCCAGGCTGCTTGGACTGTCCATAGCTGTTTTCATGGTCTCTTATCTTCCTTCACTTGTTGATTATTTTGGTCACACAAAGTGGTGCAATGTGACAGAAACTCTGCCAGAGAACGCCAGCCAAATAGAGGTTTGTTTCATGGCTCCCATTACTTTTCTTCCCATTCAACTTTCTTTTTATGTCATAAATTTGTGCCTAAGCACAATTGCATCCATTCTTTTGCTTGTCTCTCTGTGGAGACACACAAGAAATCTCAAGAAAAGTGATTTTGGTGTTAAGGACCTCAGCACTCAGGTCCACATTAAAGTCATGGCATTTTTGTTGTTCTGGATCTTCTTCTATTTTGTAGATTTCATTGCTCTGATAGTTTATGCTGGCCTCAACATTGGGACAGATTTTGGGCTAGTTCAAGAACTGCTTGTTGCTATTTGGATGTCTGCATTTCCTTCTGCACACTCTATTATATTAATATTAACCAATCCTAAACTGAAAGAAATGTGTATTTGCATCATAAAAGAAATGTATGCTCACATCATAAACATCAGACACAGTACTTTCTAA

>Green_Anole_Tas2r16

ATGACTCCCATAACGTCTCCAGTTGGCGTTCTTACTTGGGCTACTTTTGGAATTTTGAACATCATGGCCCTTTTAGGAAATGGATTTATCATAGTGGTGAATGGACATCAGTGGCTCCAGAGCAAGAAGATGATCCCTTGCAAGTTCCTCTTGGCTAGTTTGAGCACCTCCAGGTTTCTGTTGCAGATGGATTCCGTAGTGGGATATTTTCTGTATCTTATCTTTGTAGAGATCCAGAAAGAGACCCAACTATATGTTTCCAGAGCAGAAGTTGCGAATTTTATCTGGGTGTTCTTGAACATGGTCAGCTTCTGGTGTGCCTCATGGCTCGCTATGTTTTATTGTGTGAAGGTTACCAATTTTGCCAACAGGCTCATCATCTGGCTGAAGACAAGGATCAATATGCTTATACCCAGACTTCTTGGATTGTCAATAATAGTCTCTACAGTTTTCTTTTTTCCTTCAGTTGCCAATTATTATAGAAGGAAAAAGTGGTTCAATTCATCAGATGTCTTTCCTTTCACTGGTTTTCTTCTTCCACAGTTATTTCTTTCTTCATTAAATTTCAGCTTGACCCTCACTGCATCCTGTCTTTTGCTCACCTCTCTGTGGAAACACATGAGTAATCTGAAAAAAAGTGGTGTGGCTTTTCAGGATCTTAGCACTCAACTCCATTTCAAAGTTTTGATGCCTTTGCTGGTCTCTTTATTGTTCTATGTTTTATATTTTCCTTGTTTTGTGTTAGCTGTAGGTGAGATTTTTGAATTTGGAAGAATTGAGCGACTGGCTTTTGATATAATGCTGTCTCTGTATGCTTTTGTACACTCTGTAGTATTAATGCTGACCAATCCTGAACTAAAAAAAGTAGCTGCCAGCATTTTAAACATCAGACAACGAGCTCCATGA

>Green_Anole_Tas2r17

ATGACTAGCGACAATATAGTTAAAGTTGATTTCCCTGTTTGGATCATTTTTGGAACTCTGTCTCTTATTGGTATTTTGGGAAATGGATTCATCATGGCTGTGAACGGACTTCAGTGGCTTCAAAACAGAAAGATAATCCTTTGTGATTTTCTCCTGACCAGCGCAAGTACCTACAGATTTATCATGCAGTTGACTCTTCTGCTATACAACATCTTTTACTACATCCCAGAGAATATTCACTGTATTTACAGAATTGATCTTCTGTTCTTTTCCTGGATGTTTTCTAATATGATCAGCTACTGGTGTGCCACATGGCTCAGTGTTTTCTACTGTGTGAAAGTCGCCAACTTTGCCAACCCCCTCTTCCTCTGGCTGAAAACAAGGATCAATATGCTTGTACCCAGGCTGCTTGGATTGTCCATAGCAGTTTTCACAGTCTCTTGTCTTCCTTCAATTGTTGATTATTTTGGACAAACAAAGTGGGACAATCTGACAGAAATCTTGCAAGAGAATACCAGCCAAAGAAACATTTGTGACATTCCTCACATGACTTTTCTTCCCATTCAACTTTCATTTTATGTCATAAATTTGTGCCTAAGCACAATTGCAATCATTCTTTTGCTTGCCTCTCTGTGCAAACACATAAGAAATCTCAAGAAAAGTGGTGTTGGTATTAAGGACCTCAGCACTCAGGTCCACATTAAAGTCATGACATTTTTGTTGCTCTGGCTCTTCCTCTACTTTTTAGATTTCATTGGTATGATAATTTATACTAACAACACTGTTAAAACAATAAAATTGGAAGGGGTGCTTATTGACATCTTGATGTCTGCATTTTCTTCCGCTCATCCCATTATATTAATATTAACCAATCCTAAACTGAAAGAAATGTCTGCTTGCATCATTAAAAAAATGTATGCTCGTGTCATAAACATCAGATGTAGCACTTTATAA

>Green_Anole_Tas2r18

ATGTCTTCACCTCAGATCATTCTCTTCATTCTGGGTCTTGTTGATTTGGCTCTCGGTGGACTCATCTCCAATGGCTTTATACTTACAGTGATTCTCAGGGAATGGAACAAAAGCAGAAGCCTCGCTTCCATTGAACAGCTCATTCTGAGCCTGGTTCTGTCCAACCTAGGAGCAACTCTGCTAGTGCTTCCAATGTTCATCAATGACTATATCTTCCCAATTTTCACAACGAATATTACATATCTAATAATGTACCCTTTAAGTGATTATTTGATCCTTTTCAGACATTGGTTCACTGCTTGGCTCTGTTTCTTCTATTGCTTCAAGATCGTCAAGAGTACCCATTCGCTCTTCCTTTGGTTCAAACTGAAGACATCATGGCTAGTACCACAACTTATTGCAGGATCTTTGGTTGTTTCCTTGTTTATTGCCCTTCCAATGTTTTTCTTGGTCCTTACAGATTTCCTAAGCAACATGACAATGAATAATACAAAAATTAGTACAGAAATGTTACGGAATCGTACTGTTAAGGCGCCTGAAATCTTTTTTTTAATTGCTGGCTCTGGTTCACCCCTTCTTGTGATCTTAGTTTGCTCTATCCTAGTTGTGGCCTCACTCTGTAAACATGTCTACCGGATGAAGTGTAAACAGCACCATTCCGGGAGCATCCAAACAAAAGCTCATGTCAAGGCAGCTGGGACTGTGTTCTCCATCCTGTTCCTTTACCTTTCGTTTTATATGGTGCAGACCTTGTCTATGACTGTAACTGTGGGAAAAATGGAAGGGACCTTTCTCACAGTAGTTGTGATTGCATATCCCTCTGCTCAGGCTTATATACTGTTACTGGTTAATCCCAAATTAAACCAGGCAGTTAATCAGATGCTTCCAAGAAGAGTGACCTAA

>Green_Anole_Tas2r19

ATGTCTCCATTTGCTATCATCTCCTGGAGCATTCTAGGCATTTTGTGCATTGTTTCCCTTTCAGGGAATGGATTTATCTTCACTGTGACTGTGCTGCAATGGCTCCAGAAGAGGAAGATGCCACCTTGTGAATTCCTCCTGACCTGTTTGAGTGCCTCCAGATTACTAACAGAGTTGGATTGCATGGCCATCTATTTTATGCATCTATTTTCTTTTTCTGGTAGTAGAAGAATACTCTATTTCTTCTGGATCTTTTTCGATATGGCCAGTCTCTGGTGTACCTCCTGGCTCAGCATTTTCTACTGTGTGAAGGTCGTCAACTTTTCCAACTCTTTACTCCTTTGGCTAAAGTTAAGGATCAATCTGCTTCTACCCAGACTACTTGGAATATCAATGACCATTTTCATGGTCTCTTCAATTCATTCCATCTTCAGGTTTTTCAAATACAAAGAACCATGTAATCAGACTGTAACACCACTAACTAATGAGGATACTGACATCAGCATGTGGATTAGTTTTATTCCAGTACAGATAACTTTCACTTGCATAAATTTCAGCATGAACATAGCGGCAACCCTTCTTTTGCTCATCTCCTTGCGGAGGCATGTGAGAAACCTCAGAGGGAATGGTACTAGTGTCCAGGACCTCAACACTCAAGTCCACCTCAAAGTCATGAGGCCTTTGTTCATCACTCTCTTACTCTACCTTTTATTTATTGTTAGTTTGATAATAATGATAAGTAACTTTTCCCTGTTTCAAACAAATCTATCACTGAGTACAGAGATAACGATGTCCATATTTCCTTCAGCACATTCCATAATATTAATATGGACCAATCCAAAACTCCGAGAAGTGGCTGTTCACATGCTAAACATCAGACAAAGAGCTTAA

>Green_Anole_Tas2r20

ATGGATTACAGCTCAATTTCTCCACTTCATATCCTTATATGGAGCATCGCAATAACTGGAAACATAGTTGCCCTTTTAGGAAATGGGTTTATCACAGTTGTTCAAGGTCACCAATGGCTTTTAAAAAGAAAGATTTTGCCTTGTGATTTCCTCTTAATTAATTTAAGTGCCTCCAGATTTATTATGCTGCTGTTAAATTCTGTGAACTATATTATGTATTTCATCTGCTCAGAGAGCTCTCTGATTTCTTATAAAAAGGCATATACATTGATTACCTGGACTTTCATGAATATGGCCAGCCTCTGGTCCGCCACATGGCTAAGTGTTTTCTACTGTGTGAAGGTCGCTAACTTTACCAACTGCTTCTTTCTCTGCCTGAAGCCGAGGATCAACATGCTTGTGCCCAGGCTGCTTGGAATGTCAATAGTCATTTCCAGCATCTTCTCTGTTCCTTCAGTCATTGAATATCTTGGACAAATAAGGGGTGGCAACTTGACTGTAATCTTATCACTGAATGTCAGCCAAAATGAGTCTTATATTAAACCCTTATTTCATCTGCAGCTGACTTATACTTCCATAAATGTCTGCATAAGTATAATTGCATCCAGTCTTTTGCTTGCCTCATTATGGAAACACACAAGGAATCTGAAAAAAAGTGGTCTAGGTAGTAAAGACCTTAGCACTCAGGTTCACAAGAATGTCATAATAGTGGTGTTCTCTTTTGTTTTTTTTTACCTTGCGTATTTCACTGCTTCAATAATTGCAGCAAGTGATGTTTTTAAGCCTCATCGTCCTGAATTTTTAATAGTGGATGTTTTGGCAACTTCATTTCCTTCGACACACTGCATTGTATTAATATTGACTAATCCCAAACTGAAAGAAATGGCTGCTCGCATTCTGAATATCAGATAA

>Green_Anole_Tas2r21

ATGTCTCCTCCATTTGGTATCTTCTCCTGGAGCATTCTAGGTATTTTGTGGATTGTTTCCCTTTCAGGGAATGGATTTATCTTCACTGTGACTGTGCGGCAATGGCTCAAGAAGAGGAAGATGCCACCTTCTGAATTCCTTCTGACCTGTTTGAGTGCCTCCAGATTACTAACACTGTTGACTACTATGGTCAGTTATTTGCCTCTCTTTTATTCTTCAGGTAGAAGTGCAATGCTCCTTATTCCCTGGGTCTTTCTCAATATGGCCAGTCTCTGGTGTGTCTCCTGGCTCAGCATTTTCTACTGTGTGAAGGTCATCAACTTTTCCAACTCTTTACTCCTTTGGCTAAAGTTAAGGCTCAATCTGCTTCTACCCAAACTACTTGGAATATCAATGGTCATTTCCATGGTCTCTTTTCTTCCTTTCATCTTCATATTTCATCAATGCAACAAGCCATGTAATCAGACTGTAACACCACCAATCAACCATGAAGATGAAGCTGATGACAGCATGTGGATTCGTTTTATTCCACTGCAGATAACTTTTACTAGTATAAATTTCAGTGTGAACATAGCAGCAACCCTTCTTTTGCTCATCTCCTTGTGGAGGCATGTGAGAAACCTCAGAAAGAATGGTACTAGTGTTCAGGACCTCAACACTCAGGTCCACCTCAAAGTCATGAGGCCTTTGTTGATCACTCTCTTACTCTACCTTTTATATATTGCTAGTTTGATATTAATGCATACTGGCTTTTTTTATTTACAAGCAAACCTATCACTGATTGGAGAGATAATGATTACCATATTTCCTTCAGTGCATGCCATAATACTAATATGGACCAATCCAAAACTCAGAGGAGTGGCTGCTCACATGCTAAACATCAGACAAAGAGCTTAA

>Green_Anole_Tas2r22

ATGTCTCCATTTGGTATCTTCTCCTGGAGCATTCTAGGTATTTTGTGGATTGTTTCCCTTTCAGGGAATGGATTTATCTTCACAGTGACTGTGCGGCAATGGCTCAAGAAGAGGAAGATGCCACCTTGTGAATTCCTTCTGATCTGTTTGAATGCCTCCAGATTACCTACACTGTTGAATACTATGGCCATCTATTTTATGCATCTCTTTTATTCTTCAGGTAGAAGAATGATGCTCCTTATTCCCTGGGTCTTTCTCAATATTGCCAGTCTCTGGTGTGTCTCTTGGCTCAGCATTTTCTACTGCGTCAAGGTCATCAACTTTTCCAACTCTTTACTCCTTTGGCTAAAGTTAAGGCTCAATCTGCTTCTACCCAAACTAGTTGGAATATCGATGGTCATTTCCATGGTCTCTTCTCTTTCTTCCATTTTCACATTTCATCAGTGCAATGAACCATGTAATCAGACTGTAACACCACCAACCAAACATGAAGCTGAAAACTGTATGTGGATTAGTTTTTTTCCACTGAAGATAACTTTTGCTTTCATAAATTTCAGTGTGAACATAGCAGCAACCCTTCTTTTGCTCATCTCCTTGTGGAGGCATGTGAGAAACCTCAGAAAGAAGGGTACTAGTGTTCAGGACCTCAACACTCAAATCTACCTCAAAGTCATGAGGCCTTTGTTGATCACTCTCTTACTCTACCTTTTATATATTGCTAGTTTGATTACAATGAGTGGCTTTTTAAATTTGCAAAGAAAACAAGCACTGACTGCAGAGATAATGATTACCATATTTCCTGCAATGCATCCCAGAATAATAATATGGACCAATCCAAAACTCAAAAATGTGGCTGCTCACATGTTAAACATCAAGCAAAGACCTTAA

>Green_Anole_Tas2r23

ATGGTTAGCAATTCGACTTCTCCACTTGATATCCTTATCTGGATCATTGTGGGAATAGTTACCATGTTTTCCTTCTTAGGAAATGGATTCATCACAATAGTACAGGGACACCAATGGCTTCAAAATAGAAAGATTTTGCCATGTGATTTTCTTTTGACCAGTCTGAGCACCTCCAGATTTTTGATGCAGTTACTGTCTTCAGTGAACTATTTTCTGTATTTCATCTCTTTAGAGTCCTATATGAATCCCATCAAACAGGCAATTGTATATGTTATCTGGTTGTTTTTTAACATGGTCAGCCTCTGGTCTGCCACGTGGCTAAGTGTTTTCTACTGTGTGAAGATCACTAACTTTGCCAATTGCCTCTTCCTTTGGCTGAAGCCAAGGATCAACGCACTTGTACTCAGGCTGCTTGGAATATCAATAGTCATCTCCAGCATCTCCTCGCTCCCGTCCATCATCGAATATATTGGGCAAAAAAAGGGGGGCAATTTGACGGGAAGTGCCAACCACAGTGAGGCTTATAACCACAGAAATATGCTTCCTCTGCATGTCACTTTTGCTTTCATAAATTTCACCATTAACATAACTGCAACCATTGTTTTGCTCACCTCACTATGGAAGCACACAAGGAATCTGAAGAAGAGTGGTGTTGGTGGCAAAGACTTTAACACTAAGGTCCATTTCAATATCATAATACCATTGCTGTTTTATGTCGTCTTCTACTTTGTTCATATCTCCAGTCAGATAATTGTTTCAAATGAAATTACCATAGTTGGATCAGTGAAACAACGAATTACTGATATCATGGTGTCTACATTCCCAACTGTGCACTCTATTATATTAATATTGACTCATCCTAAACTGAGAGAAACGGTTGTTCGCATTCTGAATATCAAACGAAGAATTTGA

>Green_Anole_Tas2r24

ATGTCCCCTCTTGACTCCATTGTTTTTTTGGTAACCGCAGTTGTCTTAACCATCAGTGGACTCATCTCCAATGGCTTTATTGTTACAACAATTACCATTAAATGGATCAAATTCAGGAGCCTTGCTTCTAGTGAACTGCTCTTTCTGACTTTGAGTCTTTCCAATTTTGGGGCCGGAGTGTTCCTGCTTCCATTTTATATTGATGATTCCACAATATTCAGCTTCAAGCAAAACATGGCACTAAAAATACTCTTCCCAGTTGCTGTATTTGCTGTCTTCTCCAGATTCTGGCTCACTGCTTGGCTCTGTGTCTTCTATTGCATCAAGATAGTGAACAGCACCCATTTCCTCTTCCTTTGGTGCAAACTCAGGATATCATGGCTAGTAACACATCTTATTACAGGATCTCTGGTCATATCCTTTTTTGCTTCTCTGGGGGCATTGGAAAAGGATTCTATACACCTCCAAAGCAACGTAACAACAATGTTCCCAACATTGAGCCAAGGAAAATCACTGAAAGCTAGTGACTTTCGTTTCCAATTATTCTTTTTAATTTTTGGTTCATGTTCTCCTCTTCTTATAGTTTTTCTTTGCTCTACCTTGGTTGTTGCCTCACTCTCTAGACATGTTTGTCGGATGACAGATAATAATAACTTTCAAAGAAGAGCTCATTTCAAGGCAACTGGGACAGTGCTCTCCTTGCTGTTGGTTTATCTTTCATTTTTCATGGCACAGATTTTGTCTATGGCTGCAAATGTAACGTGCACTGGAAGACAATTTATTTCATCAGTGATGATCGCATATGCTCCAGTCCAGGCTGCCATCCTGGTGCTGAGCAACCCCAAATTAAAGCAGGCATTAACTGTGATGGTTCAAAGAGCAAAGCCTTAA

>Green_Anole_Tas2r25

ATGGATAATAACTTAATTTCTCCATTGGGCATCTTTAGATGGACCATGGTAGGAAGTATATCCATGGTTTCCATTTTAGGAAATGGATTTATCATAGTTGTGAGTGGGAACCGATGGCTCCAAAACAGGAAGATGGCCGCTTCTGATTTACTCTTGACTAGTTTGAGTATCTCCAGAGTTTGTTTGCATGTAACCTTTGGACTTTTCTATGTTTTAAAAGTCAGCATTGGTGATGCCTATATGGGTACTTCTGCCTATGATGCTATCATCTTTGCCTGTATGTTTTCTACCTTGGCCAGCCTCTGGTGTGCTTCATGGCTTAGTGTTTTCTATTGTGTGAAGGTCACCAACTTTGCCAACCGCTTTTTACTCTGGTTGAAGCCAAGGATCAATGTGCTCTCAATTAGACTGCTTGGAATGTCAGTGATTAGTCTTGTGGTCATCTCTGTTCCCTTCTTCTGGAGTTACGCTGAAGAAAAAAAGCGGTGCAATCTGACAGGGAGCCTGCCAGTGAACATCAGCAAAAGATGCCAAGCCTCACTTTTTATTTTTCATCCTCTTCATTTAAGTGTTGCTTCCATGAATTTCATCATCACCATAACTGCAAATGTTCTTTTGATCATCTCTTTGTGGAAACACACGCAGAATCTGAAAAAGAGTGGTATTCTTGCAAAAGACCTAAGTACTCAAATCCATATTACTATCATGAAGCCTTTGGTGTGTTATATTTTGCTCTGCCTTTTATTTTTCACAGGTATGCTGTTTTTATCAGGTAGTTTTGTGTACACTTTTGATGCTAAGAATTTTTTATCTGACATCATTTTTACCACATTTCCTTCAGCACACACCATAATATTAATCTTGACTAATCCAAAACTGAAAGCACTGTTAATTCGTACTTTAAATATAAGATGA

>Green_Anole_Tas2r26

ATGGATAATAACTTAATTTCTCCATTGGGTATTTTTACATGGACCATTATAGAAGGTATATCCATGGTTGCCATTTTAGGAAATGGATTTATCATAGTTGTGAGTGGGAACCGATGGCTCCAAGCCAGGAAGATGGTCCCTTCTGATTTTCTCTTGACTAGTTTGAGTATTTCCAGAGTGTTTTTGCATGTAACCTTTGGACTTATCTATGTTTTGGAAGTCAGCATTGGTGAAACCTTTATGTATACTTTTGCATGGGAAACTATAAGCTTTGTCTGGGTATTTTCTAACATGGCCAGCTTCTGGTGTGCTTCATGGCTTAGTGTTTTCTACTGTGTGAAGGTCACCAACTTTGCCAACCGCTTTTTACTCTGGTTCAAGCCAAGGATCAATGTGCTCTCAGTTAGACTACTTGGAATGTCAATAAGTAGTCTTGTGTTCATGTCCATTCCCTTCTTCCAGAGTTACGCTGAAGAAAAAAAGCAGTGCAATCTGACAAAGAATCTGCAAGTGAATGTCAGCAAAATAGAGGTTTGCAGAGCCTTATTTTTAATTTTTCGTCGTTTTCAGTTAATTGTTGTTTTGATGAATTTCATCATCAGCATGATTGCAACCATTCTTTTGCTCACCTCTTTGTGGAAACACATAAGGAATCTGAAAAAGAGTGAAATTGGTGCAAAAGACCTAAGTGCTCAGGTCCATATTAATGTCATGAAGCCTTTGGTGTTTTATATTTTCCTCTACCTTTCATATTTTGCCGGTGTGCTAAATTTTGCAAGTCATTCTGTGCACAATGTTGATGCTATGGAGCGTTTGTCTGACATCCTTCTTACCATATTTCCTGCAACACACACCATAATATTACTTTTGAGCAATCCAAAACTGAAAGCATTGTTAGTTCGCACTCTAAATACAAGACAAAAGGTTGATCAAGAAAAGGGGCACCAGACATGCATTTCTTGTCTCCAAGGATGA

>Green_Anole_Tas2r27

ATGTTTTCTCCTGGGTACATTGCTTTTTTGGTAACTGCAGCTGTCTTGAACATCAGCGGGTTCATCTCTAATGGCTTTATTGTTACTGTGATGATCACTGCATGGACTAAAAGCAGGAGACTTGCATCCAGTGAACAGCTCCTTCTAAGCCTGGGTCTGTCCAATTTGTGGGTGACAATTGTCCTGATTGTGTTCTGCTTCGGTTTTGCAACTTTAACCAATTTCAATGATCAAATTTTCCTTTTCTCCTTTTTCAGCTTTGCTGTGGTAGTCAGGTATTGGCTCACTGTCTTGCTATGTTTTTTCTACTGCATCAAGATTGTGAACAGTACCCACACTTTCTTCCTTTGGTGCAAACTGAGGATATCATGGTTAATACCCCGACTTCTGGTGGGATCTATTATTATCACCTTGTTAGCTTTCGTTATGATTTTAAGTTTTATGTATATACTACCACCCCCGGCCAATGTTACAACAGTGATCCATGCAATGTCACATAGTGAAAGCATCAAGAGTTTAATTGTGTTCTTTTTAACTGTTGGATCTGGTTGTCCTTTCCTTTTGGTTTTATTATGCTCCATTTTAGTTGTTGCATCACTCTGTGGACACGTCTGTCAAATGACAGGTAAAGAATCCCATCTCAGGAGTTTCCAGACAAAAGCTCATGTTCAGGCAGCTCGGACAGTGCTCTCTCTGCTATTGCTTTTTCTTTCATTTTTTGTGGCACAGACTTTGTCTATGACTGTAGATATAGGATATAATGAAAGGTTATTTATTTTCACAGTGATGACAATATACTCTCCTGCTCAGGCTGCCATTCTGGTGTTGAATAACCCCAAATTAAAGCAGGCCTTAGCCGTGATGGTTCAAAGAACAGTCTTAATATGTGAGGAGAAAAATTGA

>Green_Anole_Tas2r28

ATGTCTTCACCTCAGCTCATTTCCTTCATTCTGGCTCTTGTTGACTTGGCTCTCGGTGGATTCATCTCCAATGGCTTTATACTTACAGTGATTCTCAGGGTATGGAACAAAAGCAGAAGTCTTGATTCCAGTGAACAGCTTCTTCTGAGCCTGGTTCTGACCAATTTGTGGGCAACTGTCTTAGTCATTCTCACTTGCATCAATGACTACATCATCCCCATGTTTCCCAAGAGTTTAATGTACTCTTTAAATGATTTTATTATCATCTGCAGACATTGGTTCACTGCTTGCCTCTGTGTCTTCTATTACATCAAGATTGTGAATAGCACTCATTCCCTCTTCCTTTGGTGCAAACTGAGGATATCCTGGCTAGTACCACGACTTATTGCAGGATCTCTGGTTGTTTCATTGTTTCTTGTGTTATTTATGTCATTTTTTACTCTTATAAATATCCAGAGAAACACAACATTAATTGGGACCCAAATGAATGAAGAAATCTCACAGCATCATAACACTGGTATTCATGAAATCTTGTTTTTAATTGTTGGCTCTGGTTCACCCCTTCTTGTGATCTTAGTTTGCTGCATTCTGGTTGTTGCCTCACTCTGTAAGCACGTCTACCGGATGAAGAGTAAAGAACATAATTCTAGGAGTATCCAAACTAAAGCTCATATCAAAGCAACTGGAGTGGTGCTCTGCATCCTTCTCCTTTATCTGTTGTTTTATGTGGCGCAGACCTTTTCTCTGATTGTAATTAAGGGAAAAATTGAAATAATCTTGGTCACAACAACAGTGTATGTGTACTCTTGTGCTCAGGCCTATGTTCTGCTGCTGGTTAACCCCAACTTAAACCAGGCAGCTATTCAGGTACTTCCAAGAAGAGAAACCTAA

>Green_Anole_Tas2r29

ATGGTGAACAATATGTCTACAGTTAAAATCTTTTTTTTGATCATTTTTGAAATTGTGTCCTTTATTGGTATTTTGGGAAATGGATTCATTATAGTTGTGAATGGACACAAGTGGTTCCAAAGCAGGAAGATGATCCCTTCCGATTTCCTCCTGACCAGCCTGAGTACATCCAGGTTTATCATGCAGTTGAGTCTTCTGATAAACTACGTTCTGCTCTTCAGCCTAAAGAATAATTTCCGTTTTGCCGTAGAAGATGTTATGTTCTTTTCCTGGATGTTTTCCAACATGATCAGCCACTGGTGTGCCACAGGGCTCTGTGTTTTCTATTGTGTGAAGGTCGCCAACTTTGCCAACCCCCTCTTCCTCTGGCTGAAAGCAAGGATCAATATGCACCTACCCAGGCTGCTTGGACTTTCCATAGCAATTTTCATGGTTTCTTGTCTTCCTTTCCTTTTTGAATATTTCGGACACAGAAAGTGGTGCAATCTGACAGAAATTCTGCCGGAGAATGCTAGCCAAAGCGAGTTTGGTGACACTCCTGCCATTGTTTTTCTTCCTATGCAATTTTCTTTTTATGTCATAAATTTGTGCCTAAGCACAATTGCATCCATTCTTTTGCTCGTTTCTCTGTGGAGACACACAAGAAATCTCAAGAAAAGTGGTGTTGGTGTTAAGGACCTCAGTACTCAGGTCCACATTAAAGTCATGGCATTTTTGTTGTTCTGGATCTTCTTCTACTTTGCAGATTTAATTGCTCTGATCATTTATGCTGACCTCATTAATAGTATTGGGACAGTTCAAGGACTGCTTTTGGGAATCTCGATGTCTGCATTTCCTTCTGCACACTCCATTATATTAATATTAACCAATCTTAAATTGAAAGAAATGTTTGATTACATCATTAAAAACATATGCTCATATCATAGACATCAGGAACAGAATATGGAAAAAGGGCATTCCCTACAAGACAGGAAAAGACATTCCCTCCCTATTTAA

>Green_Anole_Tas2r30

ATGGATTGCAGCTCAATTTCTCCACTTCATATCCTTATGTGGAGCATCACAGTAATTGAAAACATAGTTGCTCTCTTAGGAAACGGATTTATCACAGTTGTTCAAAGTCACCAGTGGCTTCAAAAAAGAAAGATTTTGCCTTGTGATTTCATCTTAATTAATTTAAGTGCCTCCCGATTTATGATGATGTTGTTGACTTCCGTGCACTATATCCTGTATTCCATCTCCTCGGAGAGTTATCTGCGTTCTTATGAAAAGGCATATCTAATGATTACCTGGACTTTCATGAATATGGCCAGTCTCTGGTCTGCCACATGGCTAAGTATTTTCTACTGTGTGAAGGTCGCTAACTTTACCAACTGCCTCTTTCTCTGGCTGAAGACGAGAATCAACATGCTTGTGCCCAGGCTGCTTGGAATGTCAATAGTCATTTCCAGCATCTTCTCTGTTCCTTCAGTCATTGAATATCTTGGACAAATAAGGGGTGGCAACTTGACGATAATCTTGCCGCTGAATGTCAGTCAAAATGAGCATTATACTAAACGTTTACTTCCTCTGCATCTGACTTATACTTCCATAAATGTCTGCATAAGTATAATTGCATCCAGTCTTTTGCTTGCCTCATTATGGAAACACACGAGGAATCTGAAAAAAAGTGGTCTTGGTGGTAAAGACCTCAGCACTCAGGTTCACAAGAATGTCATAATAGTGGTAGTCTCTTATGTTTTTTTTTACCTTGCATTTTCCACCTCTCTAATAATTGAGGTAACTAATGTTTTTAAGCCTCAAAGTCCTGAAACGTTAATAGTTGAAATTTTGTCAACTTCATTTCCTTCTACACACTGCATTGTATTAATATTGACTAATCCCAAACTGAAAGAAATGGCTGCTCGCATTCTGAATATTGGTTAA

>Green_Anole_Tas2r31

ATGGATAATAACTTAATTTCTCCATTGGGTATTTTTACATGGACCATTATAGAAGGTATATCCATGGTTGCCATTTTAGGAAATGGATTTATCATAGTTGTGAGTGGGAACCGATGGCTCCAAGCCAGGAAGATGGTCCCTTCTGATTTTCTCTTGACTAGTTTGAGTATTTCCAGAGTGTTTTTGCATGTAACCTTTGGACTTATCTATGTTTTGGAAGTCAGCATTGGTGAAACCTTTATGTATACTTTTGCATGGGAAACTATAAGCTTTGTCTGGGTATTTTCTAACATGGCCAGCTTCTGGTGTGCTTCATGGCTTAGTGTTTTCTACTGTGTGAAGGTCACCAACTTTGCCAACCGCTTTTTACTCTGGTTCAAGCCAAGGATCAATGTGCTCTCAGTTAGACTACTTGGAATGTCAATAAGTAGTCTTGTGTTCATGTCCATTCCCTTCTTCCAGAGTTACGCTGAAGAAAAAAAGCAGTGCAATCTGACAAAGAATCTGCAAGTGAATGTCAGCAAAATAGAGGTTTGCAGAGCCTTATTTTTAATTTTTCGTCGTTTTCAGTTAATTGTTGTTTTGATGAATTTCATCATCAGCATGATTGCAACCATTCTTTTGCTCACCTCTTTGTGGAAACACATAAGGAATCTGAAAAAGAGTGAAATTGGTGCAAAAGACCTAAGTGCTCAGGTCCATATTAATGTCATGAAGCCTTTGGTGTTTTATATTTTCCTCTACCTTTCATATTTTGCCGGTGTGCTAAATTTTGCAAGTCATTCTGTGCACAATGTTGATGCTATGGAGCGTTTGTCTGACATCCTTCTTACCATATTTCCTGCAACACACACCATAATATTACTTTTGAGCAATCCAAAACTGAAAGCATTGTTAGTTCGCACTCTAAATACAAGACAAAAGGTTGATCAAGAAAAGGGGCACCAGACATGCATTTCTTGTCTCCAAGGATGAAAAGACAACCAAATTTCCAAATGTTAA

>Green_Anole_Tas2r32

ATGACAACAATGGTTAGCAATTCAACTTCTCTACTTGATATCCTTAAATCGACCTATCTGGGAATAGTATCTGTGTTTACTCTTTTAGAAAATGGATTCATCATAGTTGTATTGGGATATCAAGGACTTCAGAAGAGAAATATATTGCCTCATGATATCCTCTTGATAGGTTTGAGCGCCTCCAGACTAATGTCGCAGATGCTGAGTTCCACAAGCTATCTTCTGTATTTCAAGAAAGACATGGGTATTTTTAAACAGGATGTTGTATTTATTTCCTGGAACTTTTTCAACATGACTAGCATGTGGTCTGCAACCTGGCTTAGTGTTTTATACTGTGTGAAGGTCACTAACATTGCCAACTGCCTCTTCCTCTGGCTGAAGCCAAGGATCAACATGCTTGTACTCAGGCTGCTTGCGATGTCAGTAGTCATTTCCAGTATCTTCTTTGTTCCTTCAGTCCTTGAATACTTTCAACAGAAAAAGTGGGACAATCTGACTAGAAACTCACCCGTGAGTGCCAATCAAAGTGAGGGTTATAATAACGAATTCATTAACCTTGATGTGCAACTCTTTTATGTTTCCATAACTTTCTGCATAAGTATAATTGCATCCACGCTTTTGCTTGTCTCATTGTGTAAGCAGATTAGGAATCTGAAGAAGAGTGGCCTAGGTGGCAAAGATCTCAACACTCAGGTCCACAGGGATGTCATTACACTGTTGCTGTCTTACATCTTCTTCTATATTGTACATTTCACTGGTTTCATAATTTTGAAAAATGATGTTTCTAGGCATCGAAGTCTTGAAATGTTAGTTATTCAGATTCTGACAATTTCATTTCCTTGTGTACACAGCATTATGTTAATATTGACTAATCCCAAAGTAAAAGAAATGGCTGGTCATATTCTGAATATTACACAAAGAGCTTCTTAA

>Green_Anole_Tas2r33

ATGGATAATAACTTAATTTCTCCATTGGGCATCTTTAGATGGACCATGGTAGGAAGTATATCCATGGTTTCCATTTTAGGAAATGGATTTATCATAGTCGTGAGTGGGAACCGATGGCTCCAAAACAGGAAGATGGCCGCTTCTGATTTACTCTTGACTAGTTTGAGTATCTCCAGAGTTTGTTTGCATGTAACCTTTGGACTTTACTATGTTTTAGAAGTCAGCATTGGTGACACCTATATGTATACTTCAGCCTATGAAGCTGTCAGCTTTGCCTGTACGTTTTCTAGCATGACCAGCCTCTGGAGTGCTTCATGGCTTAGTGTTTTCTATTGTGTGAAGGTCACCAACTTTGCCAACCGCTTTTTACTTTGGCTGAAGCCAAGGATCAATGTGCTCTCAATTAGACTGCTTGGAATGTCAGTAATTAGTCTTGTGGTCATCCCCGTTCCCTTCTTGTGGAGATACGCTGAAGAAAAAAAGCAGTGCAATCTGACAGGGAGCCTGCCAGTGAACATCACCCAAAGAATGGTTTGCAAAGACTTATTTTTTATTTTTTATCCTTTTCAGTTAAGTGTTTTATCCATAAATTTCATCATCACCATAACTGCAAATGTTCTTTTGATCACCTCTTTGTGGAAACACACACAGAATCTGAAAAAGAGTGGTATTGTTGCAAAAGACCTAAGTACTCAGATCCATATTACTATCATGAAGCCTTTGGTGTGTTATATTTTGCTCTACCTTTTATTTTTCACAGGTATGCTAATTTTTTCAAGTCGTTTTGCGTACACTTTTAATGGTAAGAGTTTTTTATTTGACATCCATCTTACCACATTTCCTTCAGCACACACCATAATATTAATCTTGACCAACCCAAAACTGAAAGCACTGTTAATTCGTACTTTAAATATAAGATCAAAGGCTTAA

>Green_Anole_Tas2r34

ATGCTTTCACCTCAGTTCATTTTCTTCATTCTTGCTGTTATTGACTTGATGCTTGGTGGACTCATCTCCAATTGCTTTATAATTACTGTGATTCTCAGAGAATGGACCACAAGCAGAAGCCTTGCCTCCACTGAACAATTCTTTCTGAGTCTTTCTTTGACCAATTTAGGGGCGACTGTGGTACTGATTCCAAGTTACATCAATGCCTACATCTTCCCCATATTCACAAGAAATTTCATCATGCTAATCGTGTACCCTTTGGATGATTTTCTTGTCCTCTCCAGACATTGGTTCACTGCTTGGCTCATTGTCTTCTATTGTATCAAGATTGTAAACAGCACCCATTCCCTTTTTCTTTGGTGCAAACTGAAGATATCCTGGTTAGTACCATGGCTTATTGCAGGATCTCTGGTTGTTTCCTTGTTTTTTGCCCTTTTTAAATTATATATTATTCTTATGAAAATCCAAAGCAATACAACAATGATTGATATAGAAACGAATGAAGAAATGTCAGGGTATCATACTATTGGTGTTCATGAAATCTTGGTTTTAATTGTTGGCTCTGGTTCATCCCTTCTCATAGTTTTGGTTTGCTCCATCCTAATTTTGGCCTCACTCTGTAAGCATGTCTACCGGTTGAAGTGTAAAGAACACCATTCCAGAAGCATCCAAACTAAAGCTCATGTCAAGGCATCTGGGACTATACTCTTCAGCCTGTTTCTTTATATTTCATTTTATGTGGTGCAGACCTTGGTTATGACTGCAAATGTAGGGAAAATCGAAGGGACCTTTCTCACAATAGTGGTGATTGCATATCCTTCTGCTCAGGCTTGTATCCTGCTGCTGGTGAACCGTAAATTTAACCAGGCAGCCACTCAGATCCTTCCAAGATGCGACACCTAA

>Green_Anole_Tas2r35

ATGGATAATAACTTAATTTCTCCATTGGGTATTTTTACATGGACCATTATAGAAGGTATATCCATGGTTGCCATTTTAGGAAATGGATTTATCATAGTTGTGAGTGGGAACCGATGGCTCCAAACCAGGAAGATGGTCCCTTCTGATTTTCTCTTGACTAGTTTGAGCATCTCCAGAGTGTTTTGGCATGTAACCTTTGGACTTAGCTATGTTTTGGAGGTCAGCATTGGTGACATCTTTATGTATTCTTCTGCACAGGAAGCTATCGACTTTATCAGCACGTTTTCTAGCATGGCCAGCCTCTGGTGTGCTTCATGGCTTAGTGTTTTCTACTGTGTGAAGGTCACCAACTTTGCCAACCGCTTTTTACTCTGGCTGAAGCCAAGGATCAATGTGCTCTCAGTTAGACTGCTTGGAATGTCAATAAGTAGTCTTGTGTTCATGTCTGTCCCCTTCTTCCAGCATTACGCTGAAGCAAAAAAGCGGTGCAATCTGACTGGGAGCCTGCCACTGAACACCAGCCAAAGAAACGATTGCAAATTCTTACTTTTAATTTTTCGTCATTTTCAGGTAATTGTTGCTACCATGAATTTCGTCATCAGCATAACTGCAACCATTCTTTTGCTCACCTCTCTGTGGAAACACACCAGGAATCTGAAAAAGAGTGGTATTGATGCAAAAGACCTAAGTGCTCAGATCCATATTAATGTCATGAAGCCGTTGGTGCTTTATATTTTCCTCTACCTTTCATATTTTGCTGGTATTCTAAATTTTGCAAGTCATTCTGTGCACAATGTTGATGCTGTGGAGCTTTTGTCTGATGTCCTTCGTACCATATTTCCTGCAGCACATACCATAACATTAGTTTTGAGCAATCCAAAACTGAAAGCACTGTTAGTTCGTACTCTAAATATAAGACAAAAGGTTAATCTTGTCTCCAAGGACGAAAAGACAAACCAAATTTCCAAATGTTGA

>Green_Anole_Tas2r36

ATGTCTTCTTTTCAGTTCATCCTCCTCATTTTAGCTCTTGTTGACTTGGCTCTCGGTGGACTCATCTCCAATGGCTTTCTACTTACAGTGATTCTCAGGGAATGGAACAAAAGCAGAAGCCTTGATTCCAGTGAACAGCTTCTTCTGAGCCTGGTTCTGACCAATTTGTGGGCATCTGTGATATTGATCCCAGTTTACATCAATGATTATATTATCCCCATATACCCCAGGAATTTTGGAAAACAAATAATGTACCCTTTAGGTGATTTTCTTGTCATCTCCAGACATTGGTTCACTGCTTGGCTCTGTGTCTTTTATTGCATCAAGCTTGTGAACAGCACTCATTCCTTCTTCCTTTGGTGCAAACTGAGGATATCCTGGCTAGTACCGCGATTTATTGCAGGGTCTCTGGTTGTTTCCTTGTTTTTTGGCTTTTTAATGGCATTTTTAAATTATAGAAATATCCAAAGCAATACAACAATGACTGACATAAAAAGGAAAGAAGACACGTTTCGCTATCGTAGCATTGATGTTCCTCAAATATTATTTTTAATTGTTGGCTCTGGTCCACCTCTTCTCATGATTTCAGGTTGCTCCATCCTAGTTGTTGTCTCACTCTGCAGACACATGTACCGGATGAAGTGTAAAGAACATTTTTCTAAGAACCTCCAAATTAAAGCTCACATCAAGGCAGCTGGGATTATACTCTCAATTCTATTCCTTTATCTTATATTTTATGTGGTGCAAACCTTTTCTCTGCTACTAACTATGAAAAAAATGGAAGGGATTTTTGTTACAGTACTAATTATTGTATATCCCTGTGCTCAGGCTTACATCCTGCTGCTAGGTAACCCCAAGTTAAACCAGGCAGCTGCACAGGTGTTTCCAAGAAGAGAAACCTAA

>Saltwater_Crocodile_Tas2r1

ATGGAAGGCAACAGAAGCAATATTATAGAAGAGGACATCACTGAACCAAGTATATCTCTCCTAATAATCTTGCTCTTCACAGTTTTTGCTGGGTTCTCAGTCAATACTTTCATTGTGGCAGTGAATTGCACTGATTGGGTCAAAAGGAAGCGCCTCTCCACAACTGATAAGATCCTGACCATTCTTGGCTTCACCAGGTTTTCCTTGTCGTGCATAGCAGTAGTAGCCAGTTTTCATCAACTATTTCATTCTTGGACCTATGGATTTATAGAAAAAACATGCAGTATTTTAAACTGGTTTCTAAATGTTTTGAACCTGTGGTTTGGTGTCTGTCTGGGTATTTTTTATTGTGTGAAAATTGTAAACTTCAGCCACCGTTTCTGCATTTCTCTGAAACTAAAAATCTCCAGACTCATGCCATGGTTGCTGCTGGCTTCTGTGCTTCTCACCATTTGTAACACCTACCCTGCAGTCATGTTTATATCTAAAATTCAATATAAAAAATCCAACTCCAGCATCCCAGAAAATAACAAAGGAGAAGATATCCCACAAACACTGAATTTGCTTCAACTGTTTTTTCTCTTTGGCATTGGATTTCCTCTGTGTTTCACCATACTATGTATTTCAACTTTTTTCTTACTCTTGTCTCTCTGGAGACACACCTGGAAGCTTAATTCAAGCAGCAACAACTACCCTAGCATGCATGCGCATATTCTAGCGGTAAAGATAATAATGTCCTTCTTTATCATACATGTCATCCACATCTCAGCTTGGTTCATGGTGATGGCAGCAAGAATTCCAAATAGAAATTTACAGCGCTTATTTTTGTTTCAAATCGCAAATGGTTGTCCATTGATACATTCTGTTTTACTGATCCTGAGCAATCCCAAACTGAAACAGGCGTTAACCAGGATTCTGCATTACATGGGTTGTGCTAAAGAAGGAGTGTCTTAA

>Saltwater_Crocodile_Tas2r2

ATGTTCAGTTCTGTTTCCATCATTGCTATAACCATTGTGTTGACTGAGATCAGTATAGGAGTACTGGCAAATGGATTTATTGTGGCCATTAACTGGACTGACTGGAACAAAAGCAGGAAGCTGTCCTCCTGTAACACGATCTTGACATCTTTGGGCATGTCTAGACTTCTTTTGCAAGGGACAGCAATAGGGTTTCGCTCCTATTCTTTCTTCACTCCGGACACGCACAGACTGGACAATGTGCGGATCACATTGCGAGTCATACGGATGTTTGCCAACATGACCAGTACTTGGCTTGCCAGCTGTCTCAGTGTTTTCTACTGTGCAAAGATTGCCACTTTCACTCACCCTCTTTTCCTTCGAGTGAAGCAGCGAATCTCTGGGATGGTTCCACAGCTTCTCCTGGGGTCACTGCTGCTTGCCTTATTCACGTCCATCCCAACGGTTTGGGCTAATCATGATGTGTACCTTTGTAACTCCAAGGGGAACCTTCTGGGAAACACCACGTCAGCCAAGGTTAACTCAAATGTCATATACCTTTACTTCAGCTTTCTCTACACCGTAATGGCTTTCTTTCCTTTCCTCATATTCTTGGCATCATCCATGCTACTAATGGTCTCTTTGTGGAGACACAGCAGGTGCATGCAGCATTATGCACCTGACCTTCAGGACTCCAGGACACAGGCTCATGTAAGTGCAATTAAAGCTCTGATCTCTTTTCTCATCCTTTATACTTTCAGTTTTGTAGCAGAGACATTGCAAACAATGCCTACGTGTCTGACTGACAACACCTGGACTCCTGCAGTTACTTCTGTGGTGGTTGCTGCATACCCTTCAGGGCACTCCATTGTTCTCATTCTTCTTAATCCCAGGCTGAAAACAGCATTGATGCAGATTCTTCACCACATCAAGTGCCAGAAGAGACAGAGTTTCTTAAACTCTGCCACAAGTTGCTAG

>Saltwater_Crocodile_Tas2r3

ATGTTGCCTCCACTTCTTATACTATTATTCACCATTTTAGGGATTGAGATCATTGCTGGCTTTATGGGAAATGGATTTATTGCAGTCCTTATTTGCAGCGATTGGATCAGAAATAGAAAAATCTCTTCTTCTGACATGATCCTGATCTCTTTGGGCATATCAAGGTTTGTCTTGCAGGGGACCATAACAGTGTACATCCACAGTCTCTACTTTCCAGGCATGCCCAAGCTGGCTACTTTATATAAAGCATTCTATATCTTGTGGATGTTTGTAAACCACGCCAGTCTGTGGTTCAGCACCTGGCTCAGTGTCTTCTACTGTGTGAAGATTATCAACTTTACACAACTGATTTTACTGCGAATGAAGCTGAGAATTTCTGGGATGGTTCCCTGGTTTCTTCTGGGGTCAGTGCTGGTCTCTTCTATCACCACGTTGCCAATGTTTTGGATTTTTCCCAGCATTTCCTCCCCCAATTCTACTGGAAATCATGTAAACAACTCTGTGAAGAGCACTGCTTTGGACACCTCCTCGCTCAGCATTGCTCCTCTTTATTGCGCAGGTTGTTTCTTTCCTCTTATAATATCATTCTTCACTTCATCGTTATTGATTGCTTCTTTGTGGAAGCACACCAAAAAGATGCAACACAACACAACCAGCTGCCAAGATCCCAGGACAAGCGTTCATATAAATGCCATTAAAGCTCTGGTGTCTTTCCTTATCCTCTATCTTTCCAGTTTCATTGCTCAGATCCCTTTGATACTGTCAGCCTCACAAAATACCCCTACCTGGGAAGTTGCGGTCTCATTAGTAGTGGTTGCTGCATATCCATCAGGACACTCCATTATCGTGATCTTAATGCATTCCAAACTGAAACAGGCATCGGTGAGATTTTTCACCTGCACAATGTACCATCTGACAAAAGGGACTCCTAAATCTCATTAG

>Saltwater_Crocodile_Tas2r4

ATGACTCCTCTTAGTGTTGTAACTTTGTTAGGCTTAGTAATTGAGTTTGTTGTGGGTATTGTAGCAAATGGATTGATAGTAGGTTTGAATTGCATTGCATGGATTAAAAGTAAAAAACTAGATTCGTGTGCCTTAGTCCTGATTAGCCTGGGGACCTCCAGATTTTTCCTACTGTTTACACTATTGATTAATAATATTTTCTTTGTAATTCCTAAGAAGAGTAATGAACAGTGCAATACATGGAGAGCTTTCTTTTTTATCTGGATGTATCTGAGCACTCTCAGTCTCTGGTTTGCCACTTGGCTCGCTGTCTTCTACTGTGTGAAGATCACCAGCTTCAACCAACACCTTTTCCTTTGGCTGAAGTCAAGATTGTCAGGGCTACTTCCATGGCTTATTCTGGGCTCCTTGCTGGTCTCCTTGGCCACCTCTCTCCCTGCAGTCAATGCTATACATATAGATTACTTAAACAATTCAATTAATAATTTGTCCAGAAACATCACAGTGGCATGTATATATGAGACTAACACATCTCTTAGTTTCTTAATTTTGACCATGTTTGGACATTACTCTCCTTTTGTTTTATTTTTTGTTCCATCTCTGTTGCTAGTCACTTCTCTGTTGAGACATACAAAAAGAATGGGAGAAAACATGAGCACTTCCAGGGACATCAGTGCAGAAGCACATATCAGAGCAATTAAAGCTCTCCTCTCTTTCATTGTCCTGTACATTTTCTATATGGTGGCACAGCTAATTACATTGACAATGAAGTTTGCTGCCAGCAGCTCCTACCTTTTATGGTTCTGTATTATGATACTAGCTGGATATCCCTCGGTGCATTCTGTTATCCTGATTCTCAGCAATCCTAAACTGAAAGAGGCAGCACTAAAGGGCTTGCACAATGCCAGGCGTCTCCAGGAAGATGAGAGTCAGTGA

>Saltwater_Crocodile_Tas2r5

ATGGTTGGAAATGGATTTATTTTGGCCATCAACTTAGCCAGCTGGGTCAGGAACAGACTAGTATCTTCCTGTGATATGATCCTGATCTTCCTGAGCTTCTCCAGGCTCTGTCTACAGTCCTGCATGCTGATGGATTTTGTCTGCAACTTGTTTTATCCATCCTTTTATAATCAGGATGATGTATTTGAAAGCTTCAAGGCTATCTTCATGTTCCTGAACAACTCCAGCCTTTGGTTTGCCACCTGGCTTGGTGTTTTTTACTGTGCCAAGATTGCCAACTTCAGCCACTCCTCCTTTCTCTGGCTTAAACAAAACATTTCCAGCCTGGTGCCATGGCTATTAGCAGGATCATTGCTGTTCTCCTTTGGGAGCAGCCTTCCTTTCTACTGGGATATCTACAAGGTGTACTGCAACTACTCCACTGCCTTCCCCTTGGCAAACACCACAGAGCTGAAAGTGATAAAGGACACTAATTTGTTCTATGTGATTCTTCTCTGCAATGCCAGTCTATGCCTGCCTACAATAGTGTTTGTTTCCTCCATTGTACTGCTGATTTCCTCCTTGTGGAGACATACCAAACAAATGCAAAATAATGGAACTGGTCTCAGAGACCCCAGCACAGAAGCTCACAGAGGCGCCATCAAATCTGTCTTTTCTTTCTTAATCCTATACTTTTTTAATTTAATTGCTTTAATTCTCACTTTGTCTAACATCTTCTCAGCTTATGGCACCTGGGAAATCCTATGCATGATTGTCATGAGTGCCTACCCCATGGTGCACTCTGTAATCTTGATTCTGGGCAATCCCAAGCTAAGAAGGGTATCACTGAAATTTCTTCACTATGTTAGTTGTCACTTCAGAAGAGGACCTAAGTGA

>Burmese_Python_Tas2r1

ATGTCAGTCTCAGCACTTTGCAATTTGCTCTGTCTAATTATCATCATAGCTGTGACTCTTGTCGGGATGTTAGGAAATGGATTCATCGTTATTTCAGATTGCTGTGGTTGGATCAGAAGCAAAACACAGTCACCTCCTGATCTCCTGTTGATGGCACTCAGTCTGACCCGTCTTCTTTTTTTGGGAATAACTCTCAGCCTCCATTGCTTGAGCTTCCTTGATATTAATAATCCCAAATATGCAGGAAAAGCCATCGTTTTCTTCTGGGCCTTTTTCAATGCAATCACCTTGTGGATTACTACCTGCCTTGGAGTTTTTTACTGTGTGAAAATTATCAACTTCACCCAGCCCTTCCTTGTGAAAATTAAGTTGAGGATTTCCAGGATGGTTCCCCATTTGCTTGTAGCAGTAATGTTGGTTTCTTTGATTTCTGCTCTTCCTGTCATCTGGATTGGAGACTGCAACTGCTCTTATAACACAACACAGGTTCTTCCAAATGGTGGACATAAGGTGTTTCCAAGAATGACCTCCCAGACTTTTTTACTTAGTCTACTATATATTATAGGGACTTTTCCATCTTTTGTAATATTTTTGATTTCTTCTGTTTTCTTAATTTACTCCCTTGTGCACCATGTCAAGAGGATGCAACAGAACCCACTTGGTTTTAGAGATCAGAAGATGAATGTTCATTTGAGAGCCACTAAAATCCTGACCTCCTTTCTTATCCTCTATGCTGCAACTTTTGTATCAGAAATCTCAATGACGTTCTTCCCCAGTCCCTGGACATCAGTCATATCCATAATTGTGGTTACTTCATATAATTCAGGACACACCATTGCTTTGATTGTCATGAATTCCAAATTAAGGGAAGGATTAAGCAAGATGTTCCACTGTCTGAGAAAACAGACATGA

>Chinese_alligator_Tas2r1

ATGACTCCTTTAAATGTTGTTACTTTGATAGGTTTAGTAATTGAGTTTGTTGTGGGTATTATAGCAAATGGATTGATAGTAGGTTTTAATTGCATTGCATGGATTAGAAGTCAAAAACTAGATTCGTGTGCCTTAGTCCTGATTAGCCTGGGGACCTCCAGATTTTTTTTACTGCATGCAATATTGATTAATAATATTTTCTTTGCAATTCCTAAGAAGAGGATTGAACAATGCAATATGTGGAGAGCTATCAATTTTACCTGGATGTATCTGAGCATTCTCAGTCTCTGGTTTGCCACTTGGCTCGCTGTCTTCTACTGTGTGAAGATCACCAGCTTCAACCAACCCCTTTTCCTCTGGCTGAAGCTGAGATTTTCAGGGCTACTTCCATGGCTTATTCTGGGCTCCTTGCTGGTCTCCTTGGCCACCTCTCTCCCTTCAGTCAATGTAATACATATAAATTACTTAAACAATTCAATTGATAATTTGTCCAGAAACATCACAGTGGTATGTCTATATAAGACTAACACATCTCTTAGTTACTTAATTTTGACCATGCTTGGACAGTACTCTCCTTTTGTTTTATTTTTTGTTCCATCTCTGTTGCTAGTCACTTCTCTGTTGAGACATACAAAAAGAATGGGAGAAAACATGAGCACTTCCAGGGACATCAGTGCAGAGGCTCATATCAGAGCAATTAAAGCTCTCCTCTCTTTCATTTTCCTGTACATTTTCTATATGTTGGCACAGTTTTTTACATTGACAAATAAGTTCGCTACCAGCAGCCCCTACCTTATATGGCTCTGTATTATGATACTAGCTGGATATCCCTCTGGGCATTCTGTTATCCTGATTCTCAGCAATCCCAAACTGAAAGAAGCAGCACTAAAGGGCTTGCACAATGCCAGGTGTCTGCAGGAAGATGAGAGTCAGTGA

>Chinese_alligator_Tas2r2

ATGCAATACAGACCAAAGGGTAGTGCTTCCACTAACCCCATTCCTGCAGAGCAGCGTTACGCGCCGACTTGTTTTTCCAGAAGAGATTTTGTGACATCTTCAGACATGGAAGGCAACAGAGGCAACATTTCAAAAGGCAATATCTTGGAGTCAGATATGTCTGTCCTCATAACTGTACTCTTTGAGACTTTTCTCGGGATCTCACTAAATGCTTTCATTATAGCTGTGAACTGCATTGATTGGGTCAGCAGGATTTGCTTATTGTTTGCAGAACATGTAGAGCTTGTTACTTCAACATTTAATCCTTCGTTTTATAACAGCAAGTCCAGATACTTCATGTTTGCAGGTCTGGCTTGGTTCCTGAGCACATCCAACCTGTTCTTCGCTGCCTGCTTGTCTGTTTATTACTGTGTGAAGATTGCAAACTTCAGCTGCCGTCTCTTCATCACGCTGAAACAAAAAATCTCCCAGCTCATGCCATGGTTGCTGCTGGTCTCAGTGATGATTTCCTTACTCAGCAGCCTCCCTACCTTCGTGGCCATTTATAATGTCTCAGATAATAGCTGCAACTCCAGCTGCTCACAAAACCACACAGGAGACAATGTCACACAACACAGAATTCTTTTGCACATGATTATTGTCTTTTCCTTTGGCTTTTCTATAGGTTTCACCATATTATGCATCTCAGCTGTTCTCTTACTCTTTTCACTCTGGAGACACATCCGACACATGCAAGGGAGCTCAGCTGGTGTCGGGAAGCCTAGCATGGAGGCACATGTGAAAGCAGTGAAAATGGTACTGTGGTTCTTATTCATCAATCTTATTCACTTCCTATCTTGGTTAAGCTGCATTACACTTATATTTACAACAAATATTTTTGTGCAACATTTTCTTATTCAGGTTACAATTTTTTGTCCATCGATCCATGCTCTTATACTGATTCTCAGCATTCCCAAATTGAAACAGGCACTGGCCAGGATCCTGCGCTATGTAAAATGCAAGGGCTGTGCAAAAGAGGGAATACCTTAA

>Chinese_alligator_Tas2r3

ATGACTGTGAGAAGAAATATGTTCAGTTCTGTTTCCATCATTGCTATAACCATTGTGTTGATTGAGATCAGTGTAGGATTACTGGGAAATGGATTTATTGTGGCCATTAACTGGACTGACTGGATCAAAAGCAGGAAGCTGTCTTCCTGTAACACAATCTTGACATCTTTGGGCATCTCTAGACTTCTTTTGCAAGGGACAGCAATAGTGTTTCGCTCCTATTCTTTCTTCACTCTGGACACGCATAAACTGGACAATGTGCGTATCACATTGCGAGTCATACGGATGTTTGCCAACATGACCAGTGCTTGGCTTGCCAGCTGTCTCAGTGTTTTCTACTGTGCAAAGATCGCCACTTTCACTCACCCTCTCTTCCTTCGAGTGAAGCAGAGAATCTCTGGGATGGTTCCACAGCTTCTCCTGGGGTCGCTACTGCTTGCCTTATTCACATCCATACCAACAGTCTGGGCTAATCATGATGTTTACCTTTGTAACTCCAAGGGGAGCCTTCTGGGAAACACCACGTCAGCCAAGGTTAACTCAAACATCATATATCTTTACTTCAGCTTTCTTTACACCGTAATGGCTTTCTTTCCTTTCCTCATATTCTTGGCATCATCCATGCTACTGATGGTCTCTTTGTGGAGACACAGCAGGTGCATGCAGGATTATGCACCTGACCTTCAGGACTCCAGGACACGGGCTCATGTAAGTGCGATTAAAGCTCTGATCTCTTTCCTCATCCTTTATACTTTCAGTTTTGTAGGAGAGACATTGCAAACAATGCCTACTTGTCTGACTGACAACACCTGGACACCTGCAGTTACTTCTGTGGTGGTTGCTGCATACCCTTCAGGGCACTCCATTGTTCTCATTCTTCTTAATCCCAGACTGAAAACAGCATTGGTGCAGATTCTTCGCCACATCAAGTGCCAGCAGAGACAGAGACTTTCTTAA

>Chinese_alligator_Tas2r4

ATGGAAGGCAACAGAGGCAACATTTCAAAAGGCAATATCTTGGAGTCAGATATGTCTGTCCTCATAACTGTACTCTTTGAGACTTTTCTCGGGATCTCACTAAATGCTTTCATTATAGCTGTGAACTGCATTGATTGGGTCAAAAAGAGACGCCTTTCCACAAGTAATCAGCTTTTGACCATTCTCAGTTGCAGCAGGATTTGCCTATTGCTCTCAGAAGATGCAGAGTTTGTTTCCTCAACATTTAATCCTTCGTTTTATCACACCAATTCTGCACGCCTAATGTTTGCAGGTGTGGCTTGGTTCCTGAGCACATCCAACCTGTTCTTCGCTGCCTGCTTGTCTGTTTATTACTGTGTGAAGATTGCAAACTTCAGCTGCCGTCTCTTCATCACGCTGAAACAAAAAATCTCCCAGTTCATGCCATGGTTGCTGCTGGTCTCAGTGATGATTTCCTTACTCAGCAACCTCCCTATCTTCATTGCCATTTATAACATCTCAGATAATAGCTGCAACTCCAGCTGCTCACAAAACCACACAGGAGACAATGTCACAGGAGACAGAATTCTTTTGGACATAATTTTTGGCTATTGCTTTGGCTTTTCTATAGGTTTCACCATATTATGCATCTCAGCTGTTCTCTTACTCTTCTCACTCTGGAGACACATTCGACATGTGCAAGGGAGCTCAGCTGGTGCCGGGAAGCCTAGCATGGAAGCACATGTGAAAGCAGTGAAAATGGTAATGTGGCTCTTATTAATCAATGTTATTCACTTCCTAGTTTGGTTAAGCTTCATTACACTTGCATTTTCACCAAATATTTTTGTGCAACATTTTCTTATTCAGGTTACAATTTTTTGTCCATCAATACATGCTCTTGTACTGATTCTCAGCATTCCCAAATTGAAACAGGCACTGGCCAGGATCCTGCACTATGTAAAATGCAAGAACTGTGCAAAAGAGGGAATACCTTAA

>Chinese_alligator_Tas2r5

ATGGTGGATAAAAGCTGTGCCTTCCTAAGTGCTATAGAGCTATCACCAATCATTCTCTTTTATCTGAGCATTGTGGCAATTGAATCTGTGATAGGGATTGTTGGAAATGGATTTATTTTGGTCATCAACTTAGCCAGCTGGGTCAGGAACAGAGTAGTGTCTTCCTGTGATATGATTCTGATCTTCCTGAGCTTCTCCAGGCTCTGTCTACAGTCCTGCATGCTGATGGATTTTGTCTGCAACTTGTTTTATCCATCTTTTTATAATCAGGATGATGTATATGAAAACTTCAAGGCTATCTTCATGTTCCTGAACAACTCCAGCCTTTGGTTTGCCACCTGGCTTGGTGTGTTTTACTGTGCCAAGATTGCCAACTTCAACCACTCCTCCTTTCTCTGGCTTAAACAAAACATTTCCAGCCTGGTGCCATGGCTATTAGCAGGATCATTGCTGTTCTCCTTTGGAAGCAGCCTTCCTTTCTACTGGGATATCTACAAGGTGTACTGCAACTACTCCACTGCCTTCCCCTTGGAAAACACCACAGAGCTGAAAGTGATAAAGAACACTAATTTGTTTTATGTGATTTTTCTCTGCAATGCCAGTCTATCCCTGCCTACAATAGTGTTTGTTTCCTCCATTGTACTGCTGATTTCCTCCTTGTGGAGACATACCAAACAAATGCAAAATAATGGAACTGGTCTCAGAGACCCCAGCACAGAAGCTCACAGAGGCGCCATCAAATCTGTCTTTTCTTTCTTAATCCTATACTTTTTCAATTTAATTGCTTTAATTCTCACCTTGTCCAACATCTTCTCAGCTTACGGCACCTGGGATATCCTATGCATGCTTGTGATGAGTGCCTACCCCATGGTGCACTCTGTAATCTTGATTCTGGGCAATCCCAAATTAAGAAGGGTATCATTGAAATTTCTTCACTATGCCAGTTGTCATTTTAGAGGAGGACCTACGTGA

>Chinese_alligator_Tas2r6

ATGTCATCACTCTCTGCCATTATTTTTATTATTTTATATGGTGTTCAGTTCTTTGTAGGCATTATCGCTAATATATTTATAGTGACTGTGAACGTGATTGACTGGACTAAAGACATTAAGTTGTCTTCAAATGATCAAATCCTGGTATATTTGGGACTGTTCAATGTTTTTGTGCAGTGCACAGCGACAGCTGCTGACTTCTGCTTTTTTTTCTGGACAGACCTACTCTACTCTGGTTTCAGCTCGCAAACTTTTTTCTTTTTCGTATTTTTTGGCAGTATCTGTACTTCCTGCTTTACTGGATATCTATGTACTTGCTATTATGTGAAGATAACTGACAGTACTTATCCTCTTTACCTGAGGATGAAGATGGCATTTATCAAGAACTTACCCTGGCTTCTCCCATGGATCATTGCAACAAGCTTTGGATTGAGTCTGGCTGCAGTTTGGGATGTATCTAAGAAAGTTTCCCTTGACATGACAGCTAATTTCAGCACCAATTATACCAAACCATTGCTTTTGTTTCATTACTCCACTGCCTTCCGCATCATCCTCCTTTTGCTGGAATGTATTTGGCCTCTTATAGTAACATCATTTTTAGTCCTGAAGCTCATCAAGACCCTCTGCAAGCACATCAGGAACATGGAGAGGACCATGGCCTTTGGCCAACCTAATCTGGATGCCCACAAACATGCCACCCGGACCCTGACATCTCTCCTTATCCTTTTTATTTCCTACAACCTGCTTTGGAGTATACTCGTCTATGATATTTTTTCATATCCCAGCACTGGCTTTTTAATTTGCATCACTTTGCTTGCCACACTCACATCCGTACAGGCCATAACCTTGATCCTGTGTAACCGAAGAATGAAGCAGAAAGCTCTGAGGATCCTACAGAGCATTAGACAATTTTTAGGTGGATAG

>Chinese_alligator_Tas2r7

ATGGAAGGCAACAGAAGCAATATTACAGAAGAGGACGTGGCTAAACCAAGTATATCTCTCGTAATAATCTTGCTCTTTACAGCTTTTGTTGGGATCTCAGTCAATACTTTCATTGTGGCAGTGAATTGCACTGATTGGGTCAGAAGGAAGCGCCTCTCCACAACTGATCAGATCCTGACCATTCTTGGCTTCACCAGGTTTTCCTTGTCGTGCATAGCAGTAGTAGACAATTTTTGTCAACCATTTCATTCTTGGACCGATGGATTTATAGACAAAACATTCAGTATTTTAAACTGGTTTCTAAATGTTTTGAACCTGTGGTTTGCTGTCTGTCTGGGTATTTTTTATTGTGTGAAAATTGCAAACTTCAGTCACCATTTCTGCATTTCTCTGAAACTAAAGATCTCCAGGCTCATGCCATGGTTGCTGATGGCTTCCGTGCTTCTCGCCATTTTTAACACCTTCCCTGCAGTCATGTTTATTTTTAAATTTCAACATAAGAAATCCAACTCCAGCATCCCAGAAAATAACAAAGGAGAAGATATACCACAAATAATATATTTTCTTCATCTGTTTTCTCTCTTCGGCATTGGATTTCCTCTGTGTTTCACCATACTATGTATTTCAACCTTTCTCTTACTCTTGTCTCTCCGGAGACACACCCGGCAGCTTAATTCAAGCAGCAACAACAACCCTAGCATGCATGCCCATATTCTAGCGGTAAAGATAATAATGTCCTTCTTTATCATCCATGTCATCCACTTCTCAGCTTGGTTATTCGTGATGGCAGCAAGTATTCCAAATAGAAAATTACAGCGGTTATTTTTGTTTCAGATCGCAAATGGTTGTCCATTGACACATTCTGTTTTACTGATTCTGAGCAATCCCAAACTGAAACAGGCATTAACCAGGATTCTGCATTACATGGGTTGTGCTAAAGGAGTGTCTTAA

>Chinese_Softshell_Turtle_Tas2r1

ATGCTTACTCCAGTTGCTCTCATCTCTCTGATCCTTTTGGGATTTGAGTCCCTTGTTTCAAATCTTGGAAATGGATTTATCATAGTTGTGATTTTCTCTAACTGGATCAAAAGCAGAAAGCTGGCCTCCTGTGAGCACATCCTGATCTCCCTGAGCATCTCCAGATTTCTCTTACAGTGGCTTGTAATGCTGAGCAACTTCATCTATGTCAGTTTTCCAACGACTTCTGCACTGGGATGCAAACACAAAGCATTTGGAATCCTGTGGGCTTATCTAAACTTAGTCAGTCTCTGGTGTGCCACCTGTCTTAGCTTCTTCTACTCTGTAAAGATTACCAACTTTACCCAACCCCTCTTTCTCTGGCTGAAGCTGAGAATTGCTTGGCTGGTGCCCAGACTGCTTCTGGGGTCCTTGATCGTTTCCTTGGTCAGCACCATGCCATTAATCTGGAGTGATATTGGTTTTGATTTATGCAACTCAACAAAAAGTCTAGAACGAAACACAACCTGGAATGATGCTAAGGATATCCCATATATCTTCTTTGTTCCTGTGCAAATCCTTGTATTGGTCATCCCTTTCATCATATTCTTGGTTTCATCTACCCTATTGCTCATCTCTCTATGGAAACACACCAAGAAAATGAAAAATAATGTTACTCGTTTCAAAGATCTCAGTGTGGAGGCTCATATAGGCGCCATGAAATCTCTGCTCTCTTTTTTTATTCTTTACATTATGTATTTTGTTACTGTAATGGTTCTTTTGACATCTATAATCAAATTTCAACACCCGGTACGTTTACCTTATGAAGTTCTATTGTCTGCATATCCTTCTGGACACCCCGTTATTTTAATTCTGACCAATCCAAAATTGAAACAGGTGGCAGTGAAGATTCTGCATCAAATCAAATGCCAATTGAGAGAAGGGACTTTATAA

>Chinese_Softshell_Turtle_Tas2r10

ATGAGTCTTGCAATTATTATTGCTTTGAGCATTTTAGTAATTGAGCTTATTATAGGGATTATAGCAAATGGGTTGATAATCATTGTAAATTGCACTGCGTGGATCAGAAGCCGAAAACTGACTTCATGTGACATGATCCTGACTAGTCTGGGCATCTCCAGATTTTTCCTACAATGTATGATATTTGCTAACAATATCTTTTTTGCATCATCTCCAGTGATGGATGGGCAGTGTGATATATGGAGAATCATCTACATTGTTTGGATGTATCTAAGTATTCTCAGTCTCTGGTTTGCCACCTGGCTCAGTGTCTTCTACTGTGTGAAGATCGCCAACTTCAGCCAACCCCTCTTCCTCTGGCTGAAAAGGAGAATATCAGGGCTACTACCCAAGCTGCTCATGGGATCCTTGCTGGTCTCCTTGGTCACCTGTGTCTCTTCAGTCAATGCCATAGATAGAAAGTATGTAGGTAATTCAACAAATAATATGTCAATGAACACCAGAGTGAAATGTAGACCTAAATCTAATTTATCTTCTGGTCTCGTTATTTTGTCCATGCTTGGATACTCCTTTCCTTTCTTTATATTTATCATTCCCTCCGTACTACTAATCATTTCTCTGTGGAGACATACCAAGAGGATGGAGAAAAACACAAGCAGCTCCCAAGACACTATTGTGGAGGCTCATGTCAGTGCAGTTAAAGGTCTGGTTTCTTTCATTTTCTTCTATGTTTCATATTTTATGGCACAAGTAATATTTTCAATAGGAACATCTGCCCACAGCAATGTCTACTTTATATGGTTTTGGGTAGTGGTAATGACTGCTTATCCTTGTGGGCATGCCATTGTCTTGATTCTGAGTAATCCCAAACTGAAGAGGGCAGCGGTGAGGGCTTTGAACTATGCAAAGTGCAGGCTGAAAGATGGGGCTTCATAA

>Chinese_Softshell_Turtle_Tas2r11

ATGGGAGACAATGATTATGACATTGAACAAGAGGATGACATCACCGTACCAACCATAATTATGCTGGTAATCTTAGTAGCTGAGGATTTTGTCGGGATGTGGATAAATTCCTTCATTGTGGCCATCAATTGCTTTGAACGTATCAAACACAGGGGCCTGTACTCAAGTGACAATATCTTGACTGTTGTGGCATTCTCAAGATTTTGCGTCTTGCTCAAAACAACCTTACAGACTTTTTGCTCAATATTTTACCTAGAGATCTATTACATGGACACCGTGTTCCAAGCCTTCCGAGCTGTGACCTGGTTTCTGAACTCTTCCAATCAATGGTTTGCTGCTTGCTTGGGTGTATTTTACTGTGTGAAAATTGCAAACTTCAGCCACCCCCTTTTCATCTCTCTAAAATTCAGAATCTCCAGATTGGTGCCATGGTTGCTTCTGGTTTCTGTGCTTTTTTCCTTGTTCAGCAGCCTTCCTTTCCTTAATACTCCTTACAAAATACGGTATAATAATTTCAACTCCAGCCTTCGAGGGGTTTATTACACTGTGAAGAATGTCACAGTGGAAACGTCTGTTTCTCACGTACTTTTTATCTGTGGCACTGGATTTTCCGCAGCGTTCACCATATTCATCATTTCAGCCTTTCTCTTGCTGTTCTCTCTCTGGAGACACACCCGGCGGATGCAAAACAACTCCAGCTGTTTTAGGAGCCCTTGCGTGGAGGCCCATATTCAAGCCATGAAAGCCATCATGTCGTTCTTCCTCATCAATGTTGTTAACTTCATAGCTTTGCTGATCTTGTTAACAAATACACTTGAAGAAACCTCTCTTATGGGCATTGCCTGTACAATCATCATAGATGCTTGTCCATCAGTACATTCCATTGTCTTGATTCTGAGCAATCCCAAACTGAAGACCACATTGATTAAAGTTCTGCATTATGCAAAGTGCAAGGGGTGA

>Chinese_Softshell_Turtle_Tas2r2

ATGCTTTCTGCAATTATTATTGCTTTGATTGTTTTAGGAATTGAGCTCATTATAGGGAGTATAGCAAATGGACTGATGATTATTGTGAATTGCCTAGAATGGATCAGAAGCAGAAAACTGACCCGTTGTGACATGATCTTGACTAGTCTGGGCATCTCCAGATTTTTTCTACAGTGCATGATATTCATTAACAGTATCGTCCTTCAATTATGTCAAGATATAAATAGATCTTGTGATACTTTAAGTTATTTCTTTGTTGCTTGGATGTACCTAAGTACTCTCAGTCTCTGGTTTGCCACCTGGCTCAGTGTCTTCTACTGTGTGAAGATCGCCACATTCAGCCAGCCCCTTTTCCTCTGGCTGAGGCAGAAAATATCAGGGCTACTGCCACCGTTACTCCTGAGTTCCTTATTGGTTTCCTTGCTCACCTGCTTCCCTTCAGTCAATACTGTATATAGAAATAGTTCAATGAATAATCTGTCAGGAAACACCACAGTGGAATCTAAATGTGTGATTGATTTATTTTCTGGCCTGTCTATGTTCTCTACCATTGGATTTTATTCTCCCTTCATTATATTTATTGTTTCCTCTGCACTGTTAATCACTTCTCTGTGGAAACACAGCAAGAGGATGAGAAAAGCCATGAGCAGCTCTAAGGATACCATTACTGAGGCTCATGTCAGAGCAATTAAAGGACTGATCTCTTTCATTTTCTTCTACAGTTCATATTTTGCAGTACTAGTCATATTTTTGATAGAAATATTCAGGAACAACTTCGGCTTCTTGTTGTTGTGGGGAGTGATAATGGCTGCTTATCCCTCTGGGCACTCTGTTATCCTAGTGCTGGGGAATCCCAAACTGAAGAAGGTAGCAGTGAAGGCTTTGCACTATGCCCAGTGCAGGCTAAGAAATGAGGTTTCATAA

>Chinese_Softshell_Turtle_Tas2r3

ATGTTTTTCATTTTTATTGCTTTGATCATTTTAGGAATGGAGCTCATTGTAGGTATGATAGCAAACGGACTGATGGTTGTTGTGAATTGCCTAGAGTGGATCAGAAGCAGAAATCTGAGCTATTGTGACATGATCCTGACTAGCTTGGGTATCTCCAGATTTTTCTTACAGTGCATGATAATCATTAATAGTGCCGTCTATCAAATGTTTTCTGAGGACAATGCATTTCTTGCTTTGATGGGAACCATCAATTTTGTCTCAAATTTTGTAAATACTCTCAGTCTCTGGTTCGCCACCTGGTTGAGTGTCTTCTACTGTGCAAAGATCGCCAACTTCAGCCAACCTCTCTTCTTCTGGCTGAAGTGTAAAATATTGGTGTTAATGCCACAGCTACTCATGGGAACCTTTCTGGTCTCCTTTGTCACCAGTCTTCCTTCAGTCAATTCCGTAAATAGAAAGTACATAAACAATTCAGTGACTTATCTATCAGGAAACACCACAGGGGAGTGGACATACTACGCTAACTTTTCTTCTGGACTTTTCGTTTTGTACATGCTTAGCCATTCCTTTCCCTTCATTATATTTATTGTTTCCTCTGCACTGTTAATCATGTCTCTGTGGAGACACACCAAAAGGATGGAAAAAAACACGGGCAGCTGCAGGGACACTGTTACTCAGGTTCATGTTAGAGCAATTCAAGGACAGCTTTCTTTTATTTTCTTCCACATTACTTATTTTGTGGCACAAGTAATATTATTCACGAGATTATTTTCCAACAGCCTCTCCAACGCGATGTGGTGTATTGCGATAATGGTTGCTTATCCCGCTGGGCATTCTGTTATCCTAGTTCTAAGTAATCCCAAACTGAAGAAGGTAGCAATGAGGGCTTTACATTGTGCCAGGTGCAGGCTGAGAGATGAGGTTTCATAA

>Chinese_Softshell_Turtle_Tas2r4

ATGTTTTTCATTTTTATTGCTTTGATCATTTTAGGAATGGAGCTCATTGTAGGGATGATAGCAAACGGACTGATAGTGGTTGTGAATTGCCTAGAGTGGATCAGAAGCAGAAATCTGAGCTATTGTGACATGATCCTGACTAGCTTGGGTATCTCCAGATTGTTCTTTCAGTGCATGATAATCATTAATACTACCATCTATCAAATATCTTCAGAGGATAATGCACATCTTGATTTGCTGAGAACCCTGGATTTTCTCTGGTGTTTTACAAGTACTCTCAGTCTCTGGTTTGCCTCCTTGTTGAGTGTCTTCTACTGTGCAAAGATTGCCAATTTCAGCCAACCCATCTTCCTCTGCCTGAAGTGGAGACTACTAGGGCTAATGCCACAGCTACTCATGGGAACCTTTCTGGTCTCCTTTGTCACCAGTCTCTCTTCAGTTTATTCCATTGATCGGAAGTACATAAACAATTCAATGACTAATCTACCAGGAAACACCACAGGGGAGTGGACATACTACACTAACTTTTCTTCTGGACTTTTCATTTTGTACATGCTTTGCCATTCCTTTCCCTTCATTATATTTATTGTTTCCTCTGCACTGTTAATCATGTCTCTGTGGAGACACACTAAAAGGATGGAAAAAACCACAGGCAGCTGCAGGGACACTGTTACCCAGGTTCATGTCAGAGCAATTCAAGGACAGCTCTCTTTCATTATCTTCCACATTTCTTATTTTGTTGCACAGGTAATATTATTCACAGAATTATTTGCCAACAGTCTCTCCACCTCGGTGTGGTGTGGTGTGATAATGGTGGCTTATCCCTCTGTGCACTCTGTTATCCTAGTTCTGGGTAATCCCAAACTGAAGAAGGTATCAATGAGGGCTTTACATTGTGCCAGGTGCAGGCCGAGCGATGAGGTTTCATAA

>Chinese_Softshell_Turtle_Tas2r5

ATGAAAAAATCATTAGTTCCTTCTGATATTTTTTATCTGATCATATCAACAATGCAATTATCAGCAGGAGTGATTGCAAATGGATTTATTGTTGGCTTAAATTGCATTTATTGGGTCAAATTCAGAACACTGACATCCTATGATATGATCCTGACCAGTCTGGCCTTCTCCAGATTTTGTCTACAATTATTTTTATCATTAAACAATTTCCTTTATAAGTTTGATCCAGATATCTTTTACGCATTTCAAACACCAAATCTTTTTCTCGTTGTCTGGATATTTATGAACCAAGTGAGTCTCTGTTTTGCAAGCTGCCTTTCTGTGTTCTACTGTGTGAAGATTGCCACTTTCAATCTGTCTGTCTTCAACTGGTTAAAACCGAAACTCTCCAAACTGGTGCCATGGCTGCTTTTGGGCTCTCTACTGCACTCCTTGATTACTACAGTTGTTTTTACATTTGTCAGCTATTTCTTTGAGATAACCTCTCGCAACTTTACAGACCATCCATCACGAAATATCACAATAACAGACAAGAGAAAGAACCTCGCAAAGATTGTTTTTCTTATACATAGCATAGGATCAGGTTTTCCCCTTAGCCTATTTATTGCTTCATCTGGTTTGTTAATCCTATCCCTTTGGAAGCACATCAGGAAAATGAATCTTAATTCAGACTTTAATCCAAGTTTCAGGAATCCCAGTACGGAGGCCCATTTGCGTGCAATTAAATCGGTGCTGTCTTTTTTGTTCCTATACATTATGTATTTTGCAGTTTCAACAGTAACAATTGGAATCTTATCCCATTTCACTGATGAATGGAAAATTATTATGTTTTCGTTTGGGGTTGCTGCCTATCCTTTTGTACACTCCACTATCTTGATTCTAGGCAACCCCAAATTAAAACAGGCCTCAGCAAAGATTTTGCATTCTGCTAATTGCTGTTTCAGATAA

>Chinese_Softshell_Turtle_Tas2r6

ATGATGAAAAGTCATTTAGCTCCTTCTGTTATTTATTACCTGATTGTATTGGCAATTGAATTATCAGCAGGAGTTGTTGCAAATGGATTTATTGTTGGTTTGAATTGTATTGATTGGGCCAAAAGCAGAACACTGACTTCCTATGATATAATCATAACCAGTCTGGCTTTCTCCAGATTTTGCCTACAATTCTTGGTGACAATAGACAATTTCTTCTCTATGTTATTCCCAAATTTTTTTGATATATTTGAAAGGCTACATTCTTATCTAGTTACCTGGATGTTCATAAACCAAGTGAGTCTCTGTTTTGCAACCTGCCTTTCTGTGTTCTACTGTTTGAAGATTGCCACTTTGAACCAGTCCCTCTTCAGATGGTTAAAATTAAGAATCTCCAAACTGACGCCATGGCTGCTTTTGGGCTCTTTGCTGTACTGCTTGGTTACCACAGTGTGTTTTACATTCTTCAGCTATTCTTATTCGGTATCCTTTTACAACTTCACAGATAATAGCACAATGTCCTACAACAGAAAGAAAGCTATGGAATTCACCTTTCTGGTGCACAGCATAGGATCTATCTTGCCCCTTATCATATTTATTGCTTCCTCTGTTTTGTTGTTCCTATCCCTTTGGAAACATATCAGGAAAATGAACTTTAATTTAGACTTTATTCCAAGTTTCAGGAACCCCAGTATGGAGTCTCATGTGCGTGCACTTAAATCTGTGCTGTCCTTCTTCATCCTCTACAATATTTATTATGCAGCTTCAACATTCTCAATAGGATACATACCCTGTTTCAGTGAGAAATGGAAGGCTATGTTCTGGACAGTTTTAGCTGCTGCTTACCCTTCTGTGCACTCCATTATCTTGATTCTGGGAAACGCCAAATTAAAATTGTCCTCCTCAAAAATTCTGTATTGTACCAATTCCTGTTTCAGACAGGTTACGTCATAG

>Chinese_Softshell_Turtle_Tas2r7

ATGCAGAGAAACATGTTTTCTTTCATTATTTCTTTGATCATTTTAGGAATGGAGCTCATTGTAGGGATGATAGCAAATGGACTGATGGTTGTTGTGAATTGCCTAGAGTGGATCAGAAGCAGAAATCTGACCTGTTGTGACATGATCCTGACTAGCTTGGGTATCTCCAGATTTTTCTTACAGTGCATGATAATCATTAATACTACCATCTATCAAATATCTTCAGAGGATAATGTACATCTTGATTTGATGAGAACCCTGGATTTTCTCTGGAGTTTTACAAGTACTCTCAGTCTCTGGTTTGCCTCCTTGTTGAGTGTTTTCTACTGTGCAAAGATTGCCAACTTCAGCCAAACCGTCTTCCTCTGTGTGAAGGGGAGACTACTAGGACTAATACCACAACTACTCATGGGAACCTTTCTGGTTTCCTTTGTCACCAGTCTCTCTGCAGTCTATTCCATAGATAGGAAGTACATAAACAATTCAGTGATTAATCTCTCAGGAAAAACCACAGGGGAGTGGACATTCTACACTAACATTTCTTCTGGACTTTTCATTTTGTACATGCTTAGCCATTCCTTTCCCTTCATTATATTTATTGTCTTCTCTGCACTGTTAATCACGTCTCAGTGGAGACACACTAAAAGGAAGGAAAAAAACACGGGCAGCTACAGGGACACTGTTACCCAGGTTCATGTCAGAGCAATTCAAGGACAATTCTCTTTCATTATCTTCCACATTTCTTATTTTGTTGCACAGGTAATATTATTCACAGGATTCTTCGCCAAAAGCCTCTCCAACTCGATGTGGTGTCTTGTGATAATGGTGGCTTATACCTCTGGGCACTCTGTTATTCTAGTTCTGGGTAATTCCAAACTGAAGAAGGTAGCACTGAGGGCTTTACATTGTGCCAGGTGCAGGCTGAGAAATGAGGTTTCATAA

>Chinese_Softshell_Turtle_Tas2r8

ATGTTTTTCATTTTTATTGCTTTGATCATTTTAGGAATGGAGCTCATTGTAGGGGTGATAGCAAACGGACTGATGGTTGTTGTGAATTGCCTAGAGTGGATCAGAAGCAGAAATCTGACCTGTTGTGACATGATCCTGACTAGCTTGGGTATCTCCAGATTGTTCTTTCAGTGCATGATAATCATTAATACTACCATCTATCAAATATCTTCAGAGGATAATACACATCTTGATTTGCTGAGAACCCTGGATTTTCTCTGGTGTTTTACAAGTACTCTCAGTCTCTGGTTTGCCTCCTTGTTGAGTGTCTTCTACTGTGCAAAGATTGCCAACTTCAGCCAAACCGTGTTCCTCTGCCTGAAGTGGAGACTACTAGGGCTAATACCACAGCTACTCCTGGGAACCTATCTGGTTTCCTTTTTCACCAGTCTCGCTTCAGTCTATTCCATAGATAGGAAGTACGTAAACAATTCAGTGATTAATCTCTCAGGAAAAACCACAGGGGAGTGGACATTCTACACTAACATTTCTTCTGGACTTTTCATTTTGTACATGCTTAGCCATTCCTTTCCCTTCATTATATTTATTGTCTTCTCTGCACTGTTAATCACGTCTCAGTGGAGACACACTAAAAGGAAGGAAAAAAACACGGGCAGCTACAGGGACACTGTTACCCAGGTTCATGTCAGAGCAATTCAAGGACAATTCTCTTTCATTATCTTCCACATTTCTTATTTTGTTGCACAGGTAATATTATTCACAGGATTCTTCGCCAAAAGCCTCTCCAACTCGATGTGGTGTCTTGTGATAATGGTGGCTTATACCTCTGTGCACTTTGTTATCCTAGTTCTGGGTAATTCCAAACTGAAGAAGGTAGCAGTGAGGGCTTTACACTGTGCCAGGTGCAGGCTGAGAGATGAGGTTTCATAA

>Chinese_Softshell_Turtle_Tas2r9

ATGTTTTCTTTCATTATTTCTTTCATCATTTTAGGAATGGAGCTTATTGTAGGGATGATAGCGAATGGACTGATGATTGTTGTGAATTGCCTAGAGTGGATCAGAAGCAGAAATCTGAGCTATTGTGACATGATCGTGACTAGCTTGGGTATCTCCAGATTTTTCTTACAGTGTTTGATAATCATTAATAGTATCATCTATCAAATATCTACAGAGATCAATACACATATTGCTTTGATGAAAACCTTCAGTTTTCTCTGGAGTTATACAAGTATTCTCAGTCTCTGGTTTGCCACTTGGTTGAGTGTCTTCTACTGTGCAAAGATCGCCAACTTCAGCCAAGCCATCTTTCTCTGGTTGAAGTGGAGAATACCAGGGCTAATGCCACAGCTACTCATGGGATCCTTTCTGGTCTCCTTTGTCACCAGTCTCCCTTCAGTCTATTTCATGGATATAAAGTACATAGACAATTCAGTGAATAATCTATCAGGAAAAACCATGGGGGAATGGACATACAACACTAACTTTTTTTTTGGCTTTTCTATTTTGTATATACTTGGCCATTCCTTTCCCTTCGTTATATTTATTGTTTCCTCTGCACTGTTAATCACGTCTCTGTTGAGACACACCAAAAGGATGGAAAAAACCACAGGCAGCTGCAGGGACACTGTTACGCAGGCTCATGTCAGAGCAATTCAAGGACAGGTCTCTTTCATTTTCTTCTACATTTTTTATTTTGTGGCACAAGTAATTTTAATCTCAGGATTATTTACCGACAGCCTCTCCAACATGTTGTGGTTTCTTGTGATAATTGTTGCTTATCCCGCTGGGCACTCTGTTATCCTTGTTCTTGGTACTCCCAAAGTGAAGAAGAGAGCAGTGGGGGCTTTGCACTGTGCCAGGTGCAGGATGAGAGATGACGTTTCATAA

>Corn_Snake_Tas2r1

ATGTCCCCACTTCTTCAAACCATCTTCCAGGTTGCGATCACAGCCCACAGCTCCATGGGTATAATTGCAAGTGGCTTTATTGTGGTTGCCGGCTACTCAACGTGGCTGAAAGGAGAAAAAATACCTACTTGTGAAGTCATCCTGATGTGCCTCAGTTCGTCTCGGATTCTCCTACAGGGAACCATCCTGCACTGCACATTTTCTTCAACGTTATATCTGTGGAATGTCCTCAAGATACAGTCAGTTCTCCTGGTGTTGTCCAGCACAGCTTGTCTGTGGTTCGCTGCCTGCCTCAGTGTCTTCTACTGCGCCAAAATCGCCACTTTCACTCACCGGTACTTTATCTTGGTGAAGCTGAGGATCGCAGAGATGGTTCCGATGTTTCTGGTGGGGTCAGCGGTGGTTTCTTTGATTTCGTGCCTCCCTTTCATCTGGATGGATGACAACATCTTGCTCTGCAACTCCACCGGGAGCCATCTGAAAAATCTCACCGTGGAGAACCATATGAGAAACATCTCCTACCTCAAAGCCTTCTCCATATATCTCACTTGGGCCGTACTTCCGCTTCTGCTTTTCGTGGCCTCGTCCACGTTGTTAATTGCGTCTCTGTGGAGACACACCGAGCAGATGAGGCAGAGCACCATGGGTTTGAAGGACCCGAGGACCAAGGCGCACGTCGAGGCCATCAAATCCCTAATCTCTTTCCTAATACTCTACGTCTGCAGCTTTGTAGCTGACGTACTGCTTGGGATTCCTTCCTGCAGAGCTAGACATGAATGGAAAAGGAACATCTGTTTGCTGGTGATTGCCGTGTGCCCTTCCGTGCACTCCGTTCTGTTAATTTTCTTTAATTTCAGACTGAAGGTGGTGCTCAAAAACATCCTGCTTTACCTAGCGTGCCTTCAGAAAAAAAGACTACCAGTAAGCCCTCGACTTACAACCATTCATTTAGTGACTTTCATTTAG

>Corn_Snake_Tas2r2

ATGTTGGTGTCAGGAGTTCACAACTGGCTGTGCCTCCTTATCATCACAGCTGTGACTCTCGTGGGGATGACTGGAAATGGGTTCATTTTTATTTCAGACTGCTGTGATCGGATCCGAAGCAAAACACAGACGGGTTCAGATCTTCTGTTGATGTCCCTCAGCCTAACCCGGTTTATTTTCCTTGGAATCACACTCGGCTTCCATTGTATTAGCTTCCTGGATATTAATCAACCCCAATACGCTGGAAGTATCACTATTTTCTTCTGGACTTTTTTCAATGCCATCACCTTGTGGATTACCACTTGCCTTGGAGTCTTTTACTGTGTGAAAATTGTCAACTTCACTCAGCACTTCCTCGTGAAAATGAAGCTGAGGATTTCCAGGATGGTTCCCCATTTGCTCGTCGCGGTGATGCTGGTTTCCTTGATCTCGGCTCTTCCTTTCTTCTGGATTGAGGATCATACCCGATTTTATAACAACACAGAGCGTATACACGAGATGATGCCCCAGATGTTCCTCTTCAGCATGCTGTATATTCTAGGGACTTTTCCGTCGTTTCTCATATTTTTAATTTCCTCTGGTTTTTTAATTTACTCCCTTGTGCACCACACAAAGAGGATGCAGAACAATTCACTTGGTTTTAGAGATCAAAGGATGGACGTTCATCTGAAGACCACTAAAATCCTGACCTCTTTCCTTATCCTCTATGCCGCAACCTTTGTAGCCGAAATCTCGATGACCTTTTCCCCCAGTCCCTGGGCGTCAGTTATGTCCAACATGGTGGTTAGTTCATATAATTCAGGACATACCATTGCTTTGATTGTTATGAATTCCAAACTAAGAGAACGACTGAGCAAGATGTTTCAGTGTCTGAGAAAACAGACATGA

>Diamondback_Terrapin_Tas2r1

ATGCTTACTCCAGTTAGTCTCATCTCTATGATACTTTTGGGACTTGAGTCCCTTGTTGCAAGCCTGGCAAATGGATTTATTATAGTTGTGATTTTCACTGGCTGGATTAAAAGCAGAAAGGTGGCCTCCTGTGAGCTCATTCTGACCTCCCTGAGTATCTCCAGGTTTCTCCTACAATGGATTGTAATGCTGAGCAACGTCATCTATATCGTTTTTCCAAGGACTTCTGCACTAGGATGCAAACACAAAGGATTCGGAATCCTGTGGAATTTTCTAAACATGGTCAGTCTCTGGTGTGCCGCCTGGCTTAGCGTCTTCTACTCTGTAAAGATTGCCAACTTCACCCGACCCTTCTTTCTCTGGTTAAAGCTGAGAATTGCTTGGCTGGTGCCAAGGCTGCTCCTGGGGTCCCTGATGGTTTCCTTGGTCAGTACCATCCCATTAGTCTGGACTGATGTTGGGATTGATCTGTGCAACTCAAGAAAAATCCCGGAAGGAAACAGAACCTTAAATGATACTAAGGATATCCCATATCTCTTCTTTATGCCTATGGAAATCATTGTATCTGCCATCCCTTTCATCATATTCTTATTTTCATCCATCCTATTGCTCATCTCTCAATGGAAACACACCAAGAAAATGAAAAACAATGTTACTGGTTTGAAAGATCTCAGTGTGGCGGCCCATACCAATGCCATGAAATCTCTGCTCTCCTTTTTTATCCTCTTCATTATATATTTTGTGACTATAATAGTCATCTTGGCAGGCACAATCAGATTTCAAAACCCTGCCCGTTTATCTTATGAAGTTCTACTTTCTGCATATCCTTCTGGACACCCCATTGTTTTGGTTCTGACCAATCCAAAACTGAAACAGTTGTCAGTGAAGATTCTGCATCAAATCAAATGCCAACTAAGAGAAGGGACTTCATAA

>Diamondback_Terrapin_Tas2r2

ATGTGGATAAATTCTTTCATTATGGCTGTGAATTGTGTTGAATGTGTCAAGCAGAGGTGCCTGTCTTCAATGATAATATCTTGGCTGTCCTGGCATTCTCAAGATTTTGCTTCTTGCTCAAAACAACTTTACAGACTTTTTGCTCAACATTTACCAGAGATCTATTACTGCACTCCGTGTTCAAGCATTCAGAGCGGTCACCTGGTTTTTGAACTCTTCCAATCAATGGTTCGCTGCCTGCTTGTGTGTATTTTACTGTGTGAAAATTGCAAACTTCAGCCACCCCTCTTCATCTGGCTAAAATTTAAAATCTCCAGGCTGGTGCCATGGTTGCTCCTGGGATCTGTGCTTTTCTCCTTGTTCAGCAGCCTCCCTTTCCTTAATGCTATTTACAAAATAGAGTGTAATGATTTCAATTCCAGCCTCAAAAGACATTACAATGCGAAAAATGTCACAGTGGAAACATCTGTGTCTCAAGTACTTTCTATCTGTGGCACTGGATTTTCCATGGCGTTCACCATATTTATCATTTCAGCCTCTCTGTTGTTGTTCTCTCTCTGGAGACACACACAACAGATGCAAAACAACTCAAGTAGTTTCAGGAGCCCCTGCATGGAGGCCCACATTCAAGCAATGAAAACTATCGTGTCTTTCTTCCTCATCAATATTGTTAACTTTATAGCTTTGCTGACCTTGTTGACGAATATATATCAAGAAACATCTCCTGCCAGCATTGCCTGTACAATCATTGTAGATGCTTGTCCATCAGTACATTCCGTTATCTTGATTCTGAGCAATCCCAAACTGAAAAAAACTTTGATTAAAGTTCTGCATTATGCAAAGTGCAAGAGATGA

>Diamondback_Terrapin_Tas2r3

ATGTTTCCTACAATTATTATTTGTTTGATCATTTTAGGAATTGAGTTCATTACAGGGATTATAGCAAACGGATTGATGATTGTTGTGAATTGCAGTGAGTGGATCAGAAGCAGAAAACTGACCTGTTGTGACATGATCCTGACTAGCCTGGGCATCTCCAGATTTTTCCTGCAGTGCATGATAAACATTAACAATATCTTAACTCACCTACCTCAAGACATGAATGAACTGTGTACTATATTGAGAATAGTGACTGTTTTCTGGATATTTCTAACTACTCTCAATCTATGGCTTGCCACCTGCCTCAGTGTCTTCTACTGCGTGAAGATCGCCAACTTCAGCCAATCTCTCTTCCTCTGGCTGAAGCTGAGAATATCAGGGCTAGTGCCACAGCTACTTATGGGATCCTTTCTGGTCTCCTTGGTCACCTGTCTCCCTTCAGTCAATACCATAGATAGAAAGTACATAGACCATTCAATGAATACTCTATCAGGAAACACCACCGGGGAATGTCGATATAAGGTTGATTTTTCTTCTAGATTCTTTATTTCGTCCATGCTTGGTTATACCTCTCCCTTCATTATATTTATTATTTCCTCCATACTGTTAATCAAGTCCTTGTGGAGACACAGCAAGAGGATGGAAAAAACCACAAGCACCTCCAGGGACACAGTTACTGAGGCTCATGTCAGAGCAATTAAAGGACTGATCTCTTTTATTTTCTTCTACATTTCTTATTTTGTGGCACTAGTAATATTTTTGTTAGAAATATCTACCATCAGCTCCTACTATGTATGGATAGTGATAATGGGTGCTTATCCCTCTGGGCACTCTGTTATCCTAATTCTGGGTAATCCCAAACTGAAGAGGGTAGCAGTGAGGGCTTTTCACTATGCCGGGTGCAGGCTGAGAGGGGCGGCTTCATAA

>Diamondback_Terrapin_Tas2r4

ATGTTTCCTATAATTATTGGTTTGATCATTTTAGGAATTGAGTTCATTACAGGGATTATAGCAAACGGATTGATGATTGTTGTGAATTGCAGTGAGTGGATCAGAAGCAGAAAACTGACCTGTTGTGACATGATCCTGACTAGCCTGGGCATCTCCAGATTTTTCCTACAGTGCATGCTAATGATTAATGGTACCGTCTTTCAATTATGTGCAGAGATGAATGAACAGTGTGCTATGTTGACAACATTGACTTTTGTCTGGCTGTTTCTAAATACTCTCAGTCTATGGTTTGCCACCTGGCTCAGTGTCTTCTACTGCGTGAAGATCGCCAACTTCAGCCAATCTCTCTTCCTGTGGCTGAAGCGGAGAATATCAGGGCTAGTGCCACAGCTACTCATGGGATCCTTTCTGGTCTCCTTGGTCACCTGTCTCCCTTCAGTCAATACCATAGATAGAAAGTACATAGATTATTCATTGAATTGTGTATCAGGAAACACCACCGGGGAATGTCAATATACGTTTGATTTTTCTTCTAGATTCTTTATTTTGTCCATGCTTGGATATGCCTCTCCCTTCATTATATTTATTATTTCCTCCATACTGTTAATCACTTCCCTGTGGAGACACAGCAAGAGGATGGAAAAAACCACAAGCACCTCCAGGGACACAGTTACTGAGGCTCATGTCAGAGCAATTAAAGGACTGATCTCTTTTATTTTCTTCTACTTTTCTTATTTTGTGGCACTAATCTTCTCAGAATTATTTGCCAACATCAGCTCCTACTATGTATGGATAGTGATAATGGGTGCTTATCCTTCTGGGCACTCTGTTATCCTAATTCTGGGTAATCCCAAACTGAAGAGGGTAGCAGTGAGGGCTTTACACTATGCTGGGTGCAGGCTGAGAGGGGCGGCTTCATAA

>Gharial_Tas2r1

ATGGAAGGTAACAGAAGCAACATTTCACAAGGCAATTTCCTGGAGTCAGATACGTTTGTCCTAATTGTGCTCTTTGAGACTTTTCTTGGGATCTCACTAAATGCTTTCATTATAGCTGTGAACTGCATTGATTGGGTCAAAAAGAGACGCCTTTCCACAAGCGATCAGCTTTTGACCATTCTCAGTTTCAGCAGGATTTGCCTATTGTTCGCGGAAGATGCAGAGTTTGCTTTTTTAACTTTTAATCCTTCATTTTATTACAGCCAGTCCGCATACTTAATGTTTGAAGGTGTGGCTTGGTTCCTGGCCACATCCAGCCTTTTCTCCGCTGCCTGCTTGTCTGTTTATTACTGTGTGAAAATTGCAAACTTCAGCTGTCGTTTCTTCATCACCCTTAAACAAAAAATTTCCCAGCTCATGCCATGGTTGCTGCTGGTTTCAGTGATGATCTCCTTACTCAGCAGCCTCCCTACCTTCATTGCCCTTCATAACGTCTCAGATAATAGCTGCAACTCCAGCTGCTCACAAAACCACAAAGGAGAAAATGTCACACACAAAAACAATTTTTTGGACATAGTTTTTATCAATTTCTTTGGCTTTTCTATAGCTTTCACCATATTATGCATTTCAGCTGTTCTCTTACTCTTCTCACTCTGGAGACACATCCGAAACATGCAAGGGAGCTCAGCTGGTGTCGGGAAGCCTAGCATGGAGGCACATATGAAAGCAGTGAAAATGGTAATGTGGGTCTTATTCATCAATGTTATTAACTTCCTAGTTTGGTTAAGCTGCATTACATTTTTTTTGTCATCAAGTATTTATGTGCAACGTTTTCTTACTCAGGTTTCAATTGTTTGTCCATCGATACATGCTCTTATACAGATTCTCAGCATTCCCAAATTGAAACGGGCACTGGCCAGGACCCTGCACTATGTAAAATGCAAGGGCTGTGCAAAAGAGGGAATACCTTAA

>Gharial_Tas2r2

ATGGAAGGCAACAGAAGCAATATTACAGAAAAGGACATTGCTGAACCAAGTATATCTCTCCTAATAATCTTGCTCTTCACAGTTTTTGCTGGGTTCTCAGTCAATGCTTTCATTGTGGCAGTGAATTGCACTGATTGGGTCAGAAGGAAGCACCTCTCCACAACTGATAAGATCCTGACCATTCTTGGCTTCACCAGGTTTTCCTTGTCGTGCATAGCAGTAGTAGCCAATTTTCGTCAACCATTTCATTCTTGGACCTATGGATTTATAGACAAAACATGCAGTATTTTAAACTGGTTTCTAAATGTTTTGAACCTGTGGTTTGGTGTCTGTCTGGGTATTTTTTATTGTGTGAAAATTGTAAACTTCAGCCACCGTTTCTGCATTTCTCTGAAACTAAAAATCTCCAGACTCATGCCATGGTTGCTGCTGGCTTCCGTGCTTCTCGCCATTTGTAACACCTACCCGGCAGTTATGTTTATGCCTAAAATTCAAGATAAAAAATCCAACTCCAGCATCCCAGAAAATAACAAAGGAGAAGATATCCCACAAATAGTGAATTTGCTTCAAGTGTTTTTTCTCTTTGGCATTGGATTTCCTCTGTGTCTCACCATACTATGTATTTCAACCTTTCTCTTACTCTTGTCTCTCTGGAGACACACCTGGCAGCTTAATTCAAGCAGCAACAACTACCCTAGCATGCATGCACATATTCTAGCGGTAAAGATAATAATGTCCTTCTTTATCATCCATGTCATCCACTTCTCAGCTTGGTTCATCGTGATGGCAGCAAGAATTCCAAATAGAAGTTTACAGCGCTTATTTTTGTTTCAAATCGCAAATGGTTGTCCATTGACACATTCTGTTTTACTGATCCTGAGCAATCCCAAACTGAAACAGGTGTTAACCAGGATTCTGCATTACATGGGTTGTGCTAAAGAAGGAGTGTCTTAA

>Gharial_Tas2r3

ATGGATTTATTGCAGTACTTATTTGCAGCGATTGGATCAGAAATAGAAAAATCTCTTCTTCTGACATGCCTGATCTCTTTGGGCATATCGAGATTTGTCTTGCAGGGGACCATAATAGTGTACATCCACAGTCTCTACTTTCCAGGCATGCCCAAGCTGGCTACTTTATATAAAGCATTCTGTATCCTGTGGATGTTTGTAAACCATGCCAGTCTGTGGTTCAGCACCTGGCTCAGTGTCTTCTACTGTGTGAAGATTATCAACTTTACACAGCTGATTTTACTGCGAATGAAGCTAAGAATTTCTGGGATGGTTCCCTGGTTTCTTCTGGGGTCAGTGCTGGTCTCTTCTATCACCACATTGCCAATGTTTTGGATTTTTCCCAGCATTTCCTCCCCCAACTCTACTGGAAATCATGTAAACAACTCTGTGAAGAGCACTGCTTTGGACACCTCCTCACTCAGCATTGCTTCTCTTTATTGCTCAGGTTGTTTCTTTCCTCTTACAATATCATTCTTCACTTCAGTCTTATTGATTGTCTCTCTGTGGAAGCACACCAAAAAGATGCAACACAACACAACCAGCTGCCAAGATCCCAGGACAAACATTCATGTAAATGCCATTAAAGCTCTGGTGTCTTTCCTTATCCTCTATCTTTCCAGTTTCATTGCTCAGATCCCTTTGATACTGTCAGCCTCACAAAATAGCCATACCTGGGAAGTTGCAGTCTCATTAGTAGTGGTTGCTGCATATCCATCAGGACACTCGATTATCTTGATCTTAATGCATTCCAAACTGAAACAGGCATCAGTGAGATTTTTCAACTGCACAATGTACCATCTGACAAAAGGGACTCCTAAATCTCATTAG

>Gharial_Tas2r4

ATGTTCAGTTCTGTTTCCATCATTGCTATAACCATTGTGTTGACTGAGATCAGTGTAGGATTACTGGCAAATGGATTTATTGTGGCCATTAACTGGACTGACTGGAACAAAAGCAGGAAGCTGTCCTCCTGTAACACGATCTTGACATCTTTGGGCATGTCTAGACTTCTTTTGCAAGGGACAGCAATAGTGTTTCGCTCCTATTCTTTCTTCACTCCGGACACGCACAGACTGGACAATGTGCGTATCACATTGCGAGTCATACGGATGTTTGCCAACATGACCAGTACTTGGCTTGCCAGCTGTCTCAGTGTTTTCTACTGTGCAAAGATCGCCACTTTCACTCACCCTCTCTTCCTTCGAGTGAAGCAGCGAATCTCTGGGATGGTTCCACAGCTTCTCCTGGGGTCGCTGCTGCTTGCCTTATTCACGTCCATCCCAACAGTTTGGGCTAATCATGATGTGTACCTTTGCAACTCCAAAGGGAACCTTCTGGGAAACACCACGTCAGCCAAGGTTAACTCAAATGTCATATACCTTTACTTCAGCTTTCTCTACACCGTAATGGCTTTCTTTCCTTTCCTCATATTCTTGACATCATCCATGCTACTGATGGTCTCTTTGTGGAGACACAGCAGGTGCATGCAGCATTATGCACCTGACCTTCAGGACTCCAGGACACAAGCTCATGTAAGTGCAATTAAAGCTCTGATCTCTTTCCTCATCCTTTATACTTTCAGTTTTGTAGCAGAGACATTGCAAACAATGCCTACGTGTCTGACTGACAACACCTGGACACCTGCAGTTACTTCTGTGGTGGTTGCTGCATACCCTTCAGGGCACTCCATTGTTCTCATTCTTCTTAATCCCAGGCTGAAAACAGCATTGGTGCAGATTCTTCGCCACATCAAGTGCCAGAAGAGACAGAGTTTCTTAAACTCTGCCACAAGTTGCTAG

>Gharial_Tas2r5

ATGGAAGGTAACAGAAGCAACATTTCACAAGGCAATTTCCTGGAGTCAGATACGTTTGTCCTAATTGTGCTCTTTGAGACTTTTCTTGGGATCTCACTAAATGCTTTCATTATAGCTGTGAACTGCATTGATTGGGTCAAAAAGAGACGCCTTTCCACAAGCGATCAGCTTTTGACCATTCTCAGTTTCAGCAGGATTTGCCTATTGTTCGCGGAAGATGCAGAGTTTGCTTTTTTAACTTTTAATCCTTCATTTTATTACAGCCAGTCCGCATACTTAATGTTTGAAGGTGTGGCTTGGTTCCTGGCCACATCCAGCCTTTTCTCCGCTGCCTGCTTGTCTGTTTATTACTGTGTGAAAATTGCAAACTTCAGCTGTCGTTTCTTCATCACCCTTAAACAAAAAATTTCCCAGCTCATGCCATGGTTGCTGCTGGTTTCAGTGATGATCTCCTTACTCAGCAGCCTCCCTACCTTCATTGCCCTTCATAACGTCTCAGATAATAGCTGCAACTCCAGCTGCTCACAAAACCACAAAGGAGAAAATGTCACACACAAAAACAATTTTTTGGACGTAGTTTTTATCAATTTCTTTGGCTTTTCTATAGCTTTCACCATATTATGCATTTCAGCTGTTCTCTTACTCTTCTCACTCTGGAGACACATCCGAAACATGCAAGGGAGCTCAGCTGGTGTCGGGAAGCCTAGCATGGAGGCACATATGAAAGCAGTGAAAATGGTAATGTGGGTCTTATTCATCAATGTTATTAACTTCCTAGTTTGGTTAAGCTCCATTACACTTTTTTTGTCACCAAATATTTACGTGCAACATTTTCTTACTCAGGTTTCAATTTTTTGTCCATTGATACATGCTCTTATACTGATTCTCAGCATTCCCAAATTGAAACGGGCACTGGCCAGGATCCTGCACTATGTAAAATGCAAGGGCTGTGCAAAAGAGGGAATACCTTAA

>Gharial_Tas2r6

ATGGTGGATAAAAGCTGTGCCTTCCTAAGTGCTATAGAGCTATCACCCATCATTCTCATTTATCTGAGCATTGTGGCAACTGAATCTGTGACAGGGATTGCTGGAAATGGATTTATTTTGGCCATCAACTTAGCCAGCTGGGTCAGGAACAGAGTAGTGTCCTCCTGTGATATGATCCTGATCTTCCTGAGCTTCTCCAGGCTCTGTCTACAGTTCTGCATGCTGATGGATTTTGTCTGCAACTTGTTTTATCCATCTTTTTATAATCAGGATGACGTATTTGAAAGCTTCAAGGCTATCTTCATGTTCCTGAACAACTCCAGCCTTTGGTTTGCCACCTGGCTTGGTGTTTTTTACTGTGCCAAGATTGCCAACTTCAACCACTCCTCCTTTCTCTGGCTTAAACAAAACATTTCCAGCCTTGTGCCATGGCTATTAGCAGGATCATTGCTGTTCTCCTTTGGGAGCAGCCTTCCTTTCTACTGGGATATCTACAAGGTGTACTGCAACTACTCCACCGCCTTCCCCTTGGCAAACACCACAGAGCTGAAAGTGATAAAGAACACTAATTTGTTTTATGCGATTCTTCTCTGCAATGCCAGTCTATGCCTGCCTACAATAGTGTTTGTTTCCTCCATTGTACTGCTGATTTCCTCCTTGTGGAGACATACCAAACAAATGCAAAATAACGGAACTGGTCTCAGAGACCCCAGCACAGAAGCTCACAAAGGCGCCATCAAATCTGTCTTTTCTTTCTTAATCCTATACTTTTTCAATTTAATTGCTTTAATTCTCACCTTGTCCAACATCTTCTCAGCTTATGGCACCTGGGAAATCCTATGCATGATTGTGATGAGTGCCTACCCCATGGTGCACTCTGTAATCTTGATTCTGGGCAATCCCAAGCTAAGAAGGGTATCAGTGAAATTTCTTCACTATGCTAGTTGTCACTTTAGAGGAGGACCTAAGTGA

>Gharial_Tas2r7

ATGACTCCTCTTAGTGTTGTCACTTTGTTAGGCTTAGTAATTGAGTTTGTTGTGGGTATTGTAGCAAATGGATTGATAGTAGGTTTGAATTGCATTGCATGGATTAAAAGTAAAAAACTAGATTCGTGTGCCTTAGTCCTGATTAGCCTGGGGACCTCCAGATTTTTCCTACTGTGTACACTATTGGTTAATAATATTTTCTTTATAATTCCTAAGATGAATAATGAACAGTGCAATACGTGGAGAGCTTTCTATTTTATCTGGATGTATCTGAGCACTCTCAGTCTCTGGTTTGCCACTTGGCTCGCTGTCTTCTACTGCGTGAAGATCACCAGCTTCAACCAACACCTTTTCCTTTGGCTGAAGCTGAGATTGTCAGGGCTACTTCCATGGCTTATTCTGGGCTCCTTGCTGGTCTCCTTGGCCACCTCTCTCCCTACAGTCAATGCTATACATATAGATTACTTAAACAATTCAATTAATAATTTGTCCAGAAACATCACAGTGGCATGTATATATGAGACTAACACATCTCCTAGTTTCTTAATTTTGACCATGTTTGGACATTACTCTCCTTTTGTTTTATTTTTTGTTCCATCTCTGTTGCTAGTCACTTCTCTGTTGAGACATACAAAAAGAATGGGAGAAAACATGAGCACTTCCAGGGACACCAGTGCAGAGGCTCATATCAGAGCAATTAAAGCTCTCCTCTCTTTCATTGTCCTGTACATTTTCTATATGGTGGCACAGGTAGTTACATTGTCAAAGAAGTTCGCTGCCAGCAGCCCCTACCTTTTATGGTTCTGTATTATGATACTAGCTGGATATCCCTTGGTGCATTCTGTTATCCTGATTCTCAGCAATCCTAAACTGAAAGAGGCAGCACTAAAGGGCTTGCGCAATGCCAGGTGTCTCCAGGAAGATGAGAGTCAGTGA

>Gharial_Tas2r8

ATGTCATCATTCTCTGCCATTATTTTTCTTATTATATATGGTGTTCAGTTTTTTGTAGGCATTATCACTAATATATTTATAGTGACTGTGAATGTGATTGACTGGACTAAAGACATTAACTTGTCTTCAAATGATCAAATCATTGTATATTTGGGACTGTCTAATCTTTTTGTGCAGTGCACAGCCACAGCTGCTGACTTCTGCTTTTTCTTCTGGACAGACCTACTCTACTCAAGTTTCAGCTCGCAAACTTTTTTCTTTTTTGTATTTTTTGGCAATTTCTGTACTTTCTGGTTTACTGGCTATCTATGCGTCTGCTATTATGTGAAAATAACTGACAGTACTTATCCCCTTTTCCTGAGGATGAAGATGACATTTATCAAGAACTTACCCTGGCTTCTCCTGTGGATGACTGTAGCAAGCTTTGGATTGAGTTTGGCTGCAGTTTGGGATGTATCTAAGAAAGTTTCCCTTGACATGACAGCTAATTTCAGCACCAATTATATCAAACCATTGCCTTTGTTTCATAACTCCAATGCCTTCCGCATCATCCTCATTTTACTGGAATGCACTTGGCCTCTTATAGTAACATCATTTTTAGTCCTGAAGCTCATCAAGACCCTCTGCAAGCACGTCAGGAACATGGAGAGGACCATGGCCTTTGGCCAACCTAATCTGGATGCCCAAAAACATGCCACCAGAACCTTGGCATCTCTCCTTATCCTTTTTATTTCTTACTACCTGCTTTGGAGTATACTCCTCTATGATATTTTTTCATATCCCAGCACTGGGTTTTTAATTTGCTTTACTTCGCTTGCCACACTCATATCCATACAGGCCACAACCTTGATCCTGTGTAACCAAAGAATGAAGCAGAAAGCCTTGAGGATCCTACAGAGCATTAGACAATTTGCAGGTGGATAG

>Green_Sea_Turtle_Tas2r1

ATGATCATTGTGAATTGCACTGAGTGGATCAGATGCAGAAAACTGACCTGTTGTGACATTATCCTGACTAGCCTGGGCATCTCCACATTTTTCCTACAGAGGACAATATTCATTAACAATATCTTCTTTGCATTATCTCCAGTGTTGAATGGACAGTGTGATATATGGAGAAACCTCTATTTTCTTTGGATGTATCTAAATACTCTCAGCCTCTGGTTTGCTACCTGGCTCAGTGTCTTCTACTGCGTGAAGATTGCCAACTTCAGCCAACCTCTCTTCCTCTGGCTGAAGCAGAGAATATCAGGGCTAATGCCACAGCTACTCATGGGCTCCTTGCTGGTCTCCTTGGTCACCTGTCTCCCTTCAGCCAATGTCATAGATAGAAAGTACATAGACAATTCAACGAATAATCTGTCAGGAAACACCAGAGTGGAATGTAGACATAATTCATCTTCTGGCCTTGTTATTTTGTACATGCTTGGATATTCCTTTCCTTTCTTTATATTTGTTGTTTCCGCCGTACTGTTAATCACTTCTCTGTGGAGACACACCAGTAGGATGGAGAAAAACACAAACCTCTCCAGTGACACCATTACTGAGGCTCATGTCAGTGTAATTAGAGGTCTGATTGCTTTCATTTTCTTCTGCATTTCTTATTTTGTGGCAATAGTGCCATTTTTATTAAAAGTATTTGCCCTGAGCAATCTCTATTTCAGATGGTTCTGTGTAGTGATAATGGCTGCTTATCCTTCTGGGCACTCTGTTATCCTGATTCTGGGTAACTCCAAACTGAAGAGGGTAGCAGTGAGGGCTTTGAACTATGCAAAGTTCTGGCTGAGAGATGAGGCTTCATAA

>Green_Sea_Turtle_Tas2r2

ATGTTTCCTGCAATTATTATTGGTTTGATCATTTTAGGAATTGAGTTCATTACAGGGATTATAGCAAACGGATTGATGATCGTTGTGAATTGCAGTGAGTGGATCAGAAGCAGAAAACTGACCTGTTGTAACATGATCCTGACTAGCCTGGGCATCTCCAGATTTTTCCTACAGTGCACGATAATCATTAACAATATCTTATTTCAACTACCTCACACTATGAATGAACTGTGTGCTATGTTGAGAACATTGGCTGTTGTCTGGATGTTTCTAAATACTCTCAATCTATGGTTTGCCACCTGGCTCAGTGTCTTCTACTGCGTGAAGATCGCCAACTTCAGCCAACCTCTCTTCCTCTGCCTGAAGCGGAGAATATCAGGGCTAATGCCACAGCTACTCATAGGATCCTTTCTGGTCTCCTTGGTCACCTGTTTCCCTTCAGTTAATGGCATAGACAGAAAGTACATAAACAATTCAATGAATAATCTGTCAGAAACCACCATCGGCGAATGTCAATATAAGGTTGATTTATCTTCTAGCTTCTTTATTTTGTCCATGCTTGGATATTCATTTCCCTTCATTATATTTATGATGTCCTCCATACTGTTAATCATTTCCCTGTGGAGACACAGCAAGAGGATGAAAAAAACCACGAGCAGCTCCAGGGACACCATTACCGAGGTTCATTTCAGAGCAATTAAAGGACTGGTCTCTTTCATCTTCTTCTACATTTCTTATTTTGTGGCACTAGTAATATTTTTGTTAGAAATATCTACCATCAACACCTACTTCTTATGGATAGTGATAATGGGTGCGTATCCCTCTGGGCACTCTGTTATCCTAATTCTGGGTAATCCCAAACTGAAGAGGGTAGCAGTGAGGGCTTTGCACTATGCTGGATGCAGGCTGAGAGATGCAGTTTCACAAAACTGCTCATGA

>King_Cobra_Tas2r1

ATGTCTCCACTTCTTCGAACCATCTTCCAGGTTGCGATCACTGCCCACAGCTTCATGGGTATAGTTGCAAGTGGCTTTATTGTGGTTGCCGGCTACTCAACGTGGCTGAAAGGGAAAAAAGTGCCCACTTGTGAAGTCATCCTGATGTGCCTCAGTTCCTCACGGATTCTCCTACAGGGCACCATCCTGCACTGCACATTCTCTTCCACCTTATACCTGTGGAATGTCCTCAGGATACAGACAGTCCTCCTGGTGTTGACCAGCACAGCTTGTCTGTGGTTTGCTGCCTGCCTCAGTGTCTTCTACTGTGCCAAAATCGCCACTTTCACTCACCGGTACTTTGTCCTGGTGAAGCTGAGGATCGTCAAGATGGTTCCCATGTTTCTGGGTGGGTCAGCAGTGGTCTCTTTGATTTCGTGCCTGCCTTTCATCTGGATGGATGACAACGTCCCGCTCTGCAACTCCACCGGGAATCCTCTGAAGAACGTCACCGTGGAGAACCACGCAGGAAGCATCTCCTACCTGAAAGTCTTCTCCATATATTTGATTTGGGCCGTCCTTCCGCTTCTGCTTTTCGTGGCCTCGTCCACGTTGTTAATTGCATCCCTGTGGAAACACACCGAGCAGATGAGGCAGAGCACCATGGGTTTGAAGGATCCGAGGACAGAGGCGCACGTTGCGGCCATCAAATCCCTAATCTCGTTCCTAATACTCTACATCTGCAGCTTTGTAGCTGATGTGCTGCTTGGGTTTCCTTCCTGCGAAGCTAGAAGAGAATGGAAAAGAAACACCTGTTTGCTGGTGATTGCTGTGTGCCCTTCCATCCACTCCGTTCTGTTAATTTTCTTTAATTTCAGACTGAAGATGGCGTTCAAAAGCCTCCTGCTTTACCTAGTGTGCCGTCAGAAAAAAGACTATAGGTAG

>King_Cobra_Tas2r2

ATGTCGGTGTCAGGAGTTCACAACTGGCTCTGCCTCCTTATCATCATTGTTGTGACTCTTGTGGGGATGACCGGAAACGGGTTCATCTTTATTTCAGACTGCTGCGATTGGATCCAAAGCAAAACACACTCAGGTTCGGATCTCCTGTTGATGTCCCTCAGCCTAACACGGTTTATTTTCCTTGGAATCACGCTTGGCTTCCATTGTATTAGCTTCCTGGATATTAATCAACCCAAATATGCCGGAAGTGTCACCATTTTCTTCTGGACCTTCTTCAATGCCACCACTCTTTGGATTACCACCTGCCTGGGAGTCTTCTACTGTGTGAAAATTGTCAACTTCACCCAGCCCATCCTTGTGAAAATGAAGCTGAGGATTTCCAACGTGGTTCCCCATCTGCTCGTCGCAGTGATGCTGGTTTCCTTGATCTCGGCTCTTCCTTTCCTCTGGATTGAGGATCACACCCAATCTTATAACAACACAGAGCGTAGACAGGAGATGATGGCCCAGATGTTCCTCTTCGGTATGCTATACATTCTAGGGACTTTCCCCTCGTTTGTCATATTTTTCATTTCCTCTGTTTTTTTAATCTACTCCCTTGTGCACCACGCGAAGAGGATGCAGAACAACTCACTTGGCTTTCGAGATCAAAGGATGGATGTTCATCTGAAGACCATTAAAATCCTGACCTCCTTCCTCATCCTATATGCTGCAACCTTTGTAGCAGAAGTCTCAATGACCTTTTCCCCTAGCCCCTGGGCATCGGTTATGTCCATAACAGTGGTTAGTTCATATAATTCAGGACACACCGTTGCTTTGATTGTCATGAATTCCAAACTAAGGGAACAATTAAGCAAGATGTTCCAGTGTCTGAGAAAAAGACATGAAGGACTGAGCCCAGACTGA

>Painted_Turtle_Tas2r1

ATGTTTCCTACAATTATTATTTGTTTGATCATTTTAGGAATTGAGTTCATTACAGGGATTATAGCAAACGGATTGATGATTGTTGTGAATTGCAGAGAGTGGATCAGAAGCAGAAAACTGACCTGTTGTGACATGATCCTGACTAGCCTGGGCATCTCCAGATTTTTCCTGCAGTGCATGATAAACATTAACAATATCTTAACTCACCTACGTCAAGACATGAATGAACTGTGTACTATATTGAGAATAGTGACTGTTTTCTGGATATTTCTAACTACTCTCAATCTATGGCTTGCCACCTGCCTCAGTGTCTTCTACTGCGTGAAGATCGCCAACTTCAGCCAATCTCTCTTCCTCTGGCTGAAGCTGAGAATATCAGGGCTAGTGCCACAGCTACTTATGGGATCCTTTCTGGTCTCCTTGGTCATCTGTCTCCCTTCAATCAATACCATAGATAGAAAGTACATAGACCATTCAATGAATAATCTATCAGGAAACACCACCGGGGAATGTCGATATAAGGTTGATTTTTCTTCTAGATTCTTTATTTCGTCCATGCTTGGTTATACCTCTCCCTTCATTATATTTATTATTTCCTCCATACTGTTAATCAAGTCCTTGTGGAGACACAGCAAGAGGATGGAAAAAACCACAAGCACCTCCAGGGATACAGTTACTGAGGCTCATGTCAGAGCAATTAAAGGACTGATCTCTTTTATTTTCTTCTACATTTCTTATTTTGTGGCACTAGTAATATTTTTGTTAGAATTATGTACCATCAGCACCTACTATGTATGGATAGTGATAATGGGTGCTTATCCTTCTGGGCACTCTGTTATCCTAATTCTGGGTAATCCCAAACTGAAGAGGGTAGCAGTGAGGGCTTTTCACTATGCTGGGTGCAGGCTGAGAGATGAGCTTTCATAA

>Painted_Turtle_Tas2r2

ATGTTTCCTGTAATTATTATTTGTTTGATCATTTTAGGAATTGAATTCATTACAGGGATTATAGCAAATGGATTGATGATTGTTGTGAATTGCAGTGAGTGGATCAGAAGCAGAAAACTGACCTGTTGTGACATGATCCTGACTAGCCTGGGCATCTCCAGATTTTTCCTACAGTGCATGCTAATGATTACTGGTATCTTCTTTCAATTATGTGCAGAGATGAATGAACAGTGTGCTATGTTGACAACATTGACTGTTGTCTGGCTGTTTCTAAATACTCTCAGTCTATGGTTTGCCACCTGCCTCAGTGTCTTCTACTGCGTGAAGATCGCCAACTTCAGCCAACCTCTCTTCCTCTGGCTGAAGCGGAGAATATCAGGGCTAGTGCCACAGCTACTCATGGGATCCTTTCTGGTCTCCTTGGTCACCTGTCTCCCTTCAGTCAATACCATAGATAGAAAGTACATAGATTATTCATTGAATTGTCTATCAGGAAACACCACCGGGGAATGTCAATATACGTTTGATTTATCTTCTAGATTCTTTATTTCGTCCATGCTTGGATATGCCTCTCCCTTCATTATATTTATTATTTCCTCCATACTGTTAATCACTTCCCTGTGGAGACACAGCAAGAGGATGGAAAAAACCACAAGCACCTCCAGGGACACAGTTACTGAGGCTCATGTCAGAGCAATTAAAGGACTGATCTCTTTTATTTTCTTCTACATTTCTTATTTTGTGGCACTAATCTCCTCAGAATTATTTGCCAACATCAGCTCCTACTTCATATTATTATGGGTAGTGATAATAGGTGCTTATCCCTCTGGACACTCTGTTATCCTAATTCTGGGTAATCCCAAACTGAAGAGGGTAGCAGTGAGGGCTTTTCACTATGCTGGGTGCAGGCTGAGAGGGGCGGTTTCATAA

>Painted_Turtle_Tas2r3

ATGCTTACTCCAGTTGGTCTCATCTCTATGATACTTTTGGGACTTGAGTCCCTTGTTGCAAGCCTGGGAAATGGATTTATTATAGTTGTGATTTTCATTGGCTGGATCAAAAGCAGAAAGGTGGCCTCCTGTGAGCTCATTCTGACCTCCCTGAGTATCTCCAGGTTTCTCCTACAATGGATTGTAATGCTGAGCAACGTCATCTATATCGTTTTTCCAAGGACTTCTGCACTAGGATGCAAACACAAAGGATTCGGAATCCTGTGGAATTTTCTAAACATGGTCAGTCTCTGGTGTGCCGCCTGGCTTAGCGTCTTCTACTCTGTAAAGATTGCCAACTTCACCCGACCCTTCTTTCTCTGGTTGAAGCTGAGAATTGCTTGGCTGGTGCCAAGGCTGCTCCTGGGGTCCCTGATGGTTTCCTTGGTCAGTACCATCCCATTAGTCTGGACTGATGTTGGGTTTGATCTGTGCAACTCAAGAAAAATCCCAGAAGGAAATACAACCTTGAATGATACTAAGGATATCCCATATCTCTTCTTTATGCCTATGGAAATCATTGTATCTGCCATCCCTTTCATCATATTCTTGTTTTCATCCATCCTATTGCTCATCTCTCAATGGAAACACACCAAGAAAATGAAAAACAATGTTACTGGTTTGAAAGATCTCAGTGTGGCAGCCCACACCAATGCCATGAAATCTCTGCTCTCCTTTTTTATCCTCTTCATTATATATTTTGTGACTATAATAGTCATCTTGGCAGGCACAATCAGATTTCAAAACCCTGCCCGTTTATCTTATGAAGTTCTACTTTCTGCATATCCTTCTGGACACCCCATTGTTTTGGTTCTGACCAATCCAAAACTGAAACAGTTGTCAGTGAAGATTCTGCATCAAATCAAATGCCAACTAAGAGAAGGGACTTCATAA

>Painted_Turtle_Tas2r4

ATGATGAAAAAATCTTTAGCTCCTGCTGTTATTTTTTATCTGATCATCTCAGCAATGGAATTATTAGCAGGAGTTGTTGCAAATGGATATATTGTTGCCTTAAATTGTATCAACTGGGTCAAAAGCAGAAAACTGACTTCCTATGATAAGATAATAACCAGTCTGGCCTTCTCCAGATTTTGCCTACAAGTCTTCGTGTCATCAGACAATTTCTTATATAAGTTATATCCAGATTTCTTTTATATGACTGAAACGTCAAGCCCTTATGCAGTTATTTGGATGTTTATAAATCAAGTGAGTCTCTGTTTTGCAAGCTGCCTTTCTGTGTTCTACTGTGTGAAGATTGCCTCTTTCAACCAAGCCCTCTTCAACTGGTTAAAACTGAAACTCTCCAAACTGGTGCCATGGCTGCTTTTGGGCTCTGTGCTGTACTGCTTGGTTACTACAGTTGCTTTTGCAGTGTTCACATATTCTTCCCACAACTCCACAGATTGTCTATCAAGAAATGTCACGATTTCAGACAACAACAAGAACCGTGCAGTGTTCACTTTTCTGATACACGGCATAGGATCTATTTCCCCCATTATTCTATTTATTGCTTCATCTGCTTTGTTAATCATATCCCTTTGGAGACACATCAGGAAAATGAACCTTAATTCAGACCTTAATCCAAGTTTCAGGAACTCAACTATGGATGCCCATGTGCGTGCACTTAAATCTGTGGTGTTCTTTTTCATCCTCTACAATATTTATTATATGGCTTCAACATTGTCAATTGGAAGCCTAGCCTATGTCAGTGATGAATTGAGAATTACAGTGTGTACAATTATAATTGCTGCCTATCCTTCTCTACACTCCATTGTCTTGATTCTGGGCAACCCCAAATTAAAACTGGCCTCAGCAAGGATTCTGCATTCTGCCAATTGCTGA

>Painted_Turtle_Tas2r5

ATGAAAAAGTCTTTAGCTACTGCTTTTATTTTTTATATGATTATCTCAGCAATAGAATTCTCAGTGGGAGTTGTTGTAAATGGATATATTGTTGCCGTAAATTGTATCGACTGGGCCAAAAGCAGAACGCTGACTTCCTATGATAAGATCATAACCAGCCTGGCCTTGTCCAGATTTTGCCTACAATTCTTCATGACAGACTATTCCTTATTTAAGTTATATTCACATCTCTTTGTTAGATTTCAAACAGCTCAGCTTTATATAGCTATCTGGTTGTTCATAAACCAAATGAGTCTCTGTTTTGCAAGCTGCCTTTCTGTGTTCTACTGTGTGAAGATTGCCACTTTCAACCAGTCCCTCTTCAGCTGGTTAAAACTGAAAATCTCCAAACTGGTGCCATGGCTACTTTTGGGATCTGTGCTTTACTGCTTGGTTACCACAGTTGCTTTTACATTGTTCAGCTATTCCTATTGCTTATCCTCTCACAACTCCACAGATCGTCTATCAACAAATAGCACGATGTCAGACAAGATAAAGAACCTTATGGAACTCACTTTTCTGATACACAGCGTAGGATCTATTTTCCCCCTTATTGTATTTATTGTTTCATCTGTTCTGTTAATCATATCCCTTTGGAGACACATCAGGAAAATGAACCTTAATTCAGACCTTAATCCAAATTTCAGAAACCCCAGCACGGACGCCCATGTGCGTGCTCTTAAATCTGTGGTGTCCTTTTTCATCGTCTACAATATTTATTATGTGGCTTCAACATTCTCAATAGGAAACATATCTTATTTGAATGCTGAATGGAAAATGAGGGTGTTTTTATTTATCAGTGCTGCTTACCCTTCTGTACACTCCATTATCTTGATTCTGGGCAACCCCAAATTAAAACTGGCCTCAGGAAAAATTCTGCATTCTGCCAATTGCTGTTTCAGATAG

>Painted_Turtle_Tas2r6

ATGATGAAAAAATATTTAGCTCCTGCTGTTATTTTTTATCTGATCATCTTAGCAATAGAATTATCAGCAGGAGTTATTACAAATGGATATATTGTTGCCTTAAATTGTATCAACTGGGCCAACAGCAGAACACTGACTTCCTATGATAAGATCATAACCAGTTTGGCCTTCTCCAGATTTTGCCTACAAATCTTGGTGACATTAGACAATGTTTTATCTAAGATATATCCAAATATCTTTGATAGATTTCAAACACTACAGCCGTACCTAGTTACCTGGATGTTCATAAACCAAGTGAGTCTCTGTTTTGCAAGCTGCCTTTCTGTGTTCTACTGTGTGAAGATTGCCTCTTTCAACCAATCCCTCTTCAGCTGGTTAAAACTGAAAATCTCCAAACTGGTGCCATGGCTGCTTTTGGGCTCTATGCTGTACTGCTTGGTTACTACAGTTGCTTTTACATTGTTTAGTTATTCCTACTGGGTATTTTCTCACAACTCCACAGATTGTCTATCAAAAAATGGTACAATATCAGACAATAAAAATAACCTTTTGGAGTTCACTTTTAGCGTAGGATCTATTTCCCCTCTTATTGTATTTATTGCTTCATCTGTTCTGCTAATCATATCCCTTTGGAGACACATCAGGAGAATGAACCTTAATTCAGACTTTAATCCAAGTTTTTGGAACCCCAGCATGGATGCCCATGTGCGTGCACTTAAATCTGTGGTGTCCTTTTTCATCATCTACACTATTTATTATGTGGCTTCAACATTCTCAATAGGAAACCTATCCTATTTCAATGATGAATTGAAAATTATGGTGTGTACATTTGTAGCTGCTGTCTACCCTTCTCTGCACTCCATTATCTTGATTCTGGGCAACCCCAAATTAAAACTGGCCTCAGCAAAAATTCTGCATTCTGCCAATTCCTGTTTTGGAGAGGTTACTTCATAA

>Painted_Turtle_Tas2r7

ATGGGGGACAATGATTGTAACTTCGTAAAAGAGGATGACATCACTAACTCAAGTATAATTGTCCTAGTAATCTTAGTATGTGAGGCTTTTGTCGGGATGTGGATAAATTCTTTCATTATGGCTGTGAATTGTGTTGAATGTGTCAAGCAGAGGTGCCTGTCTTCAACTGATAATATCTTGGCTGTCCTGGCATTCTCAAGATTTTGCTTCTTGCTCAAAACAACTTTACAGACTTTTTGCTCAACATTTTACCCAGAGATCTATTACCTGCACTCCGTGTTCCAAGCATTCAGAGCGGTCACCTGGTTTTTGAACTCGTCCAATCAATGGTTCGCTGCCTGCTTGTGTGTATTTTACTGTGTGAAAATTGCAAACTTCAGCCACCCCCTCTTCATCTGGCTAAAATTTAAAATCTCCAGGCTGGTGCCATGGTTGCTCCTGGGATCTGTGCTTTTCTCCTTGTTCAGCAGCCTCCCTTTCCTTAATGCTATTTACAAAATAGAGTGTAATGATTTCAATTCCAGCCTCAAAAGACATTATAATGCGAAAAATGTCACAGTGGAAACATCTGTGTCTCAAGTACTTTCTATCTGTGGCACTGGATTTTCCATGGCGTTCACCATATTTATCATTTCAGCCTCTCTGTTGTTGTTCTCTCTCTGGAGACACACACAACAGATGCAAAACAACTCAAGTAGTTTCAGGAGCCCCTGCATGGAGGCCCACATTCAAGCAATGAAAACTATCGTGTCTTTCTTCCTCATCAATATTGTTAACTTTATAGCTTTGCTGACCTTGTTGACGAATATATATCAAGAAACATCTACTGCCAGCATTGCTTGTACAATCATTGTAGATGCTTGTCCATCAGTACATTCCACTATCTTGATTCTGAGCAATCCCAAACTGAAAAAAACATTGATTAAAGTTCTGCATTATGCAAAGTGCAAGAGATGA

>Brown_Spotted_Pit_Viper_Tas2r1

ATGTCGGTGTCAGGAGTTCACAACTGGCTCTGCCTTATTATCACCACAGCTGTGACTCTTGTCGGGATGACTGGAAACGGATTCATCTTTCTTTCAGACTGCCATGATTGGATCCGAAGCAAAGCACCGTCGGGTCCTGGCCTCCTGCTGATGACGCTCAGCCTGACCCGATTTATTTTCCTCGGAATCACGCTCGGCTTCCATTGTTTTGGCTTCCTTGATATTAATCGACCCAAATATGCTGGACGTGTCACCATTTTCTTCTGGACCTTTTTCAATGCCACCACCCTGTGGATTACTACCTGCCTTGGAGTATTCTACTGTGTGAAAATTGTCAACTTCAGCCAGCCCTTCCTGGTGAAAATGAAGCTCAGGATTTCCAGCATGGTTCCCCATTTGCTCGTTGCGGTAGTCTTGGTTTCCTTGATTTCGGCTCTTCCTTTCCTCTGGATTGACGATCACAACCAGTCTGACAACCCAGAGGGTGTACGTGAGGTGAGGGTTCAGATGTTCCTGTTCAGCATACTGTATATTCTAGGGACTTTTCCATCGTTTGTGATATTTTTAATTTCCTCTGGTTTTTTAATTTACTCCCTTGTGCACCACGTGAAGAGGATGCAGAATAGCTCAGTTGGCTTTCGAGATCAAAGGATGGACGTTCATCTGAAGACCACTAAGATCTTGACCTCCTTCCTCATCCTCTATGCCGCAACCTTTGTAGCAGAAATCTCAATGACCTTTTCTCCCAGTCCCTGGACAATGGTTATATCCAACATAGTGGTTAGTTCATATAATTCAGGACACACCGTTGCTTTGATTGTCATGAATTCCAAACTGAGGGGACGATTATGCAAGATGTTCTGGTGTTTCAGAAAACAGACATGA

>Japanese_Gecko_Tas2r1

ATGGCTTACCTACTGTTGGTAACTGGCTTCATACTTCTCGTTATGGAAACCCTCTCTGGAATGATAACAAACGGATTTATCGTCCTCATCATTTGCATGGATTGGTTCAAAAGCAAGAAATTGCCCCCAACTTACTTGATCCTGGGCTGTCTTGGACTGTCCAGACTTCTGTGGCAGGCAATCGTGATTCTGAAGGTAACCATGACTTTCTTTTTTAGGAGCACCTATATACAGAATTACACACGGTTAACATTTATCACCATGTGGCTTTTTGCAAACACTTTGAACCTTTGGTTTGCCGCCTGGCTCAGTGTAATGTACTTTGTGAAGATCGCCATCTTCTCCCACCCCGTTTTCCTCCAAGTGAAGCAAAGATTCTCTGGGCTGCTCCTACGGCTGCTTCTCGGCTCAGTCGCCTTCTCTGCTTTTATGAACATAGCGATTATCACGGCATCAAACTACGGCTTGTCCACTTGCAATCCTTACAAAATTCAACCAAGCAACTTCAGCGATACAGACATTAAAATATCTCACTCGTGTAAGTACTTCATCATTGTAACTACTGCCCCCCATTGCCTTCCTATTATGATATTTTTGTCGTCATCCGTATTGTTAATAACCTCTCTATGGAAGCACACAAGACGTCTGCGATGCAACAGGACCGGTCCCAGGGACCTCAGCACACAAACTCACTTGACTGCCATCAAAGCTCTGGCTTCCTTCGCTATTCTGTACCTGTTCAGTTTTGTAGCGTTCACTTCACAATCACTTCTGATCTGGATAAGCCGCCGGCGCGACTGGACATCCATATTTTTTGAAAATGCGAGTGCTGTGTATCCCACTGGACATGCCATTATCCTGATATTAATTAATCCCAAACTGAAACAGGCATGGGTCCGGATGATGCATCACCTAAAATGCCATTTGAGAGAAGCATAG

>Japanese_Gecko_Tas2r10

ATGAGTGATGAAATGTCAGCATCCCTTAGGAGTCTTTGTCTGTCTGTGTTTGGCATGATGAACATTGTGGCCTTACTGGGGAATGGATTTATCATAGCTGTGAATGGCTACAGTTGGCTCCAGAGTCGGAAACTGATCCCTTGTGGCCTTCTCCTGAGCTGCCTGAGCGCCTCCAGATTTTTGACACAGGGGATTCTCACGATAAACCAATGTCTTTATCTCCGCTCTCCAGGGACTTATGAGTTTAGCTGTACAGAACAGTTCATGAACATGGCCTGGAACTACTGCAACATGGCCAGCTTCTCCAGTGACACCGCTCTCAATGTTTTCTACTGCCTGAAGATCACCACCTTTGCTCACCCTCCATTCCCCTGGTTGAAGTCAAGAATTGACCGGTTCATGCCAAGACTGCTTGCAATACCATGTATTGCTTTTGTGTTTTTCTCTCTCCCTTCGTACGTAGTTTATCTGAGTCAAGGAAGTTGCCACTTTCTGAGAAGAAACATGACGGAGCGGAGAAACCCAAAAAACAATAAAGTGTTTAAGATGTTGTCTCCTGTCCAGTTCACTCTCCCCGCCCTCTGTTTCCTTATATGCTCAGCTGCATCCGTTCTTCTGTTCATCTCTCTGTGGAGGCACATGAGGAACCTGAAAAAGAATGGCCTGGACGTGAAGGATCTCAGCACTCGAGCCCATCTCAGTGTCATGAAGTCTCTGCTGTGCTTTCTTTTCTTCTTTGTTCTATATTTTGTTGCAACGAATGTTGCATTTTTCAGTGATTTCAGGATCAGCAGCCTGGAGCACCTGATCTCCATCATTCTGCTTTCTTCCTATCCTTCTGTTCATTCAATCGTCTTAATAGTTACCAATCCCAAACTGAAGGAAACGTGTGTTCGGATTCTGAACATTGGGAAGAGATCCTCATGCTCTTCAAGCAACAGTCGTGCTGAATGA

>Japanese_Gecko_Tas2r11

ATGGCTACCTTATTAACAAAAGTCAGTGTTGTATTTCTGGTTATAGAAGCCCTCATCGGAATAGTGGCAAATGGATCTATTATCATCATTAATTTTATTGACTGGTTCAGAAACAGGAAACTGTCCCCGACAGACCTGATCCTTGTCTGTCTGGGCTCATCCAGACTTTTGCTGCAGGCAGTAGTGATTCTGGGTGCAACTCTATTTAACAATACACCAAGCAACGTACCATTTACGCTGATGGGCATATGGATATTTTCAAATGTTGTGAACCTTTGGTTTGCTGCCTGCCTCAGTGTTTTCTACTTAGCAAAGATCGCCGTCTTCTCCCACCCACTTTTCCTCCAAATCAAGCGAGGATTGCCTTGGCTGGTGCCTTGGCTGCTTCTGGGCTCAGTGGTCTTTTCTGTTGTCAGGACTATGATTGTTACCATGAGCTGGAACTATGGCTTCTTTACCTGCAATCCCTACAAATTACTTTCAATCAACAGGAGCAATGCAGAAATGAACATGTCACCCGTGTGTACGCATCTCACTGCTCTAACTGCCGTCTTTGATTTTATTCCCTTTATGGTATTTCTATCATCAACCATCTTCTTAGTAAGTTCTCTGTGGAAGCACATGAGATGTGTGCAACGCAATGGAACTGGCACCGGGGATTTCAACACACAAGCGCACTTGAGAGCCATCAAAGCTCTGGCTTCCTTTGCAGTTCTGTACCTCATCAGTCTTACAGCAATCATTTCACAACCAGTACTGGCCTGGAACAAACACACTTGGACAATTCTGCTCTTTAATGTGAGTGCTGTGTACCCCTCCGGACATGCAATTATCCTGATATTAATTAATCCCAAACTGAAACAGGCATGGGTCAGGATGATACATCACTTCAAATGCCATTTGAGGAAAGTACCATCTTAA

>Japanese_Gecko_Tas2r12

ATGGCTACTTTATTAACAAAAATCTGCTTTGTATTTCTGGTTATGGAAACCCTCTTTGGAATGGTGGCAAATGGATCTATTGTCCTCATTAATTTCATTGACTGGTTCAGAAACAGGAAACTGTCCCCGACAGACCTGATCCTGATCTGTCTGGGCTTATCCAGACTTTTGCTGCAGGCTGTAGTGATTCTGGATGTAACTATATTTATTTTGCTTAACAGTACAGTAAGCAAAGTACTGTTCGCGCTGAACGGTGTATGGATGTTTACAAACATGATGAACCTCTGTTTTGCTGCCTGCCTCAGTGGTTTCTACTTAGCAAAGATCGCTGTATTCTCCCACCCCGTTTTTCTCCAATTCAAGCAACGATTCCCTGGGCTGGTGCCTTGGCTGCTTCTGGGTTCAGTGGTCTTCTCTGTTCTCAGGACTCTTCTGGTTATCCTGAGATGGAACTATGCCTTCTCTACCTGTAATCCCTTCAAATCACTTTTAAGCAATAGGAGCAATGCAGAAATGAAGATGCCACCTGTTTGTAGGCATATCACTGCTCTAAATGCTGTTTTCGATTTTATTCCCTTTATGGTATGTCTGTCATCGTCCATCTTTTTAATAAGTTCTCTGTGGAACCACATGAGAAGAGTCCAAAGCAATGGAATTGGCACCAGAGATCTCAACACACAAGCGCACTTGAGAGCCATCAAAGCTCTGGCTTCCTTTGTGGTTCTATACCTCATCAGTTTTGCAGCGAACACTTCACAAGCAGAACTGGTCTGGAATTACCAACACACTTGGACAACTCTGCTCTTTAATGTGAGTGCTGTATACCACTCTGGACATGCCTTTATCCTGATATTAATTAATCCCAAACTGAAACGGGCATGGGTCAGGATGATACATCACTTCAAATGCCATTTGAGGAAAGTACCGTCTTAA

>Japanese_Gecko_Tas2r13

ATGGCAACCTTATTAACACTAGTCTGCTTTGTATTTCTGGTTATGGAAACCCTCATTGGAATGGTGGCAAATGGATCTATTATCGTCATTAATTTCTTTGACTGGTTCAGAAACAGGAAACTGTTCCCGACAGACCTGATCCTGATCTGTCTTGGCTTATCCAGACTTTTCCAGCAGGCAGCAGTTATTCTGGATATAACTCTCCTTATCTTGTTTAACCGTGTACTGAGCAATTTACTGTTCATGATGACCATCATATGGACATTTACAAACACCTTAAACCTTTGGTTTGCTACCTGCCTCAGCATTTTCTACTTAGCAAAGATCGCCGTCTTCTCCCATCCCGTTTTTCTCCAAATCAAGCGACGCTTGCCTGGGCTGGTGCCGTGGCTCCTTCTAGGCTCAGTGATCTTCTCTGTTGTCAGGACTATGATTGTTATCACAAACTGGAATTATGGCTTGTCTACCTGCGATCCCTTCAGACCGCTTTCAATCAACAGGAGCAATGTTGAAATGAACCTGCCACCAGTGTGTGGGCATATCACTGCTCTAACTACTGTCTTCGATTTTATTCCCTTTATGGTATTTCTGTCATCGTCCATCTTTTTAATAAGTTCTCTGTGGAAGCACATGAGAAGAGTCCAAAGCAATGGAACTGGCACCAGGGATCTCAACACACAAGTCCACTTGAGTGCCATCAGAGCTCTGGCTTCCTTTGCAGTTCTGTACCTCATCAGCTTTGTAACAACGATTTCACAGTTAGTACTGGTCTGGGAAAACCAACACACTTGGAACATTCTGCTCTTTAACGTGAGTGCTTTGTACCCCTCCGGACATGCCATTATCCTGATATTAATTAATCCCAAATTGAAACAGGCATGGGTTAGGATGATACATCACTTCAAATGCCGTTTGGGGAAAGTACCGTCTTAA

>Japanese_Gecko_Tas2r14

ATGGCTCCCTTATTAACAATAGTCTGCTTTGTATTTCTGGTTATGGAAACCCTCATTGGGATGCTGGCAAATGGATCTATTGTCCTCATTCATTTCATTGACTGGTTCAGAAAGAGGAAACTGTCCCCGACGGACCTGATCCTGATATGTCTTGGTTCATCCAGACTTTTGCTGCAGGCAGTAATGATTCTGGGTGTAACTCTATTTATTTGGTTTAACCATATAACCAGCAATTTAGTGTTCACAATGATCATTGGATGGATGTTTACGAACACTGTGAACCTTTGCTTTGCTGCCTGCCTCAGTGTTTTCTATTTAGCAAAGATCGCCATCTTCTCCCACCCAGTTTTTCTCCAAGTCAAGCGACGATTGCCTGAACTGGTGCCTTGGCTGCTTCTTGGCTCAGTGGTCTTCTCTGCTGTCAGGACTCTTATAGTTACTACAAGCTGGAACTATGGCTTCTTTACTTGCACTCCCTACAAATTACTTTCAATTAACAGGAGCAACGTTGAAATGAACATGCCACCTGTGTGTATGCATATCACTGCTCTAGCTGCCGTCTTCGATTTAATTCCTTTTATGATATTTCTGTCATCGTCCATCTTTTTAATAAGTTCTCTCTGGAAGCACATGAGATGTGTGCAACGCCGTGGAATTGGTGCCAAAGATCTCAACACACAAGCGCACTTGAGTGCCATCAAAGCTCTGGCTTCTTTTGCAGTTCTGTACGTCATCAGTTTTGCAGCGGTCACTTCACAATTCGTACTCGGCTGGACCAAGCAATACACTTGGACAATTCCGCTCTTTAATGTGATTGCCCTATACCCCTCTGGACATGCCATTATCCTGATATTAATTAATCCCAAACTGAAACAGGCGTGGGTCAGGATGATACATCACTTAAAATGTTGTTTGGGCTAA

>Japanese_Gecko_Tas2r15

ATGGCTACCTTATTAACAAAAGTCCTCTTTCCATTTCTCGTTATGGAAACCCTCATTGGAATGGTGGCAAATGGATCTATTGTCCTCATTCATTTCATTGACTGGTTCAGAAAGAGGAAGATGTCCCCGACGGACCTGATCCTGATCTGTCTTGGCGTAGCGAGACTTTTGCTGCATGCAGTAATTATTCTGGATGCAACTCTATTTACTTTACATAAGAATACACTGAGCAACATACATTTCACGCTAAACAGTGTATGGACATTTATGAGCACTGTGAACCTCTGGTTTGCTGCCTGCCTGAGCGTTTTCTACTTAGCAAAGATCGCTGTATTCTCGCACCCTGTTTTTCTCCAAGTCAAGCAAAGATTCTCTGGGCTGGTGCCTTGGCTGCTCCTTGGCTCCGTGGTCTTATCTGCTGTCGTGACTATTATTATTATCACGAGCTGGAGCTTGTCAACCTGCAATCCCTACGAACCACTTTCAATCAACAAGAGCAATGCAGAAATGAACATGCCAGACATGTGCTGGCATATTGCTGCTCAATCTGCTGTCTTTGATTTTATTCCCTTTATGGTATGTCTGTCATCGTCCATCTTTTTAATAAGTTCTTTGTGGAGGCACATGAGAAGAGTCCAAAGCAATGGAACTGGCACCAGGGATTTCAACACACAAGCGCACTTGAGTGCCATCAAAGCTCTGGCTTCCTTTGCAGTTCTGTACCTCATCAGTATTGCAGCGGACATTTCACAATTCGTACTGGTCTGGGACAACCAACACACTTGGTCAATTCTGCTCTTTAATGTGAGTGCTGTGTACCCCTCCGGACATGCCATTATCCTGATATTAATTAATCCCAAATTGAAACAGGCATGGGCCAGAATGATACATCACTTAAAATGCCGTTTGAGGAAAGTACCATCTTAA

>Japanese_Gecko_Tas2r16

ATGGCTACCTTATTAACAATAGTCTGCATTCTATTTCTGGTTATGGAAACTGTCATTGGAATGGTGGCAAATGGATCTATTTTCCTCATTTTTTTCATTGACTGGTTCAGAAAGAGGAAACTGTCCCCGACGGACCTGATCCTGATCTGTCTTGGCTTATCCAGACTTTTGCTGCATGGAGTAATGATTCTGTATGTAACTCTATTTACTTTGCTTAACAATACCCGAATCAACCTACTGCTCGCGCTGACCGGTGTATGGATATTTACGAACACTGTGAACCTTTGGTTTGCTGCCTGTCTCAGTGTTTTCTACTTAGCAAAGATCGCTGTATTCTCTCACCCAGTTTTTCTCCAAGTCAAGCGAAGATTCTCTGGGCTGGTGCCTTGGCTGCTTCTCGGCTCAGTGGTCGTATCTGCTGTCGTGACTATCATTATGATCACAAGCTGGCAGTATGGTTACTCTACCTGCAGTCCTTACAAACCACTTTCAATCAACAGGAGCAATGCAGAAATGAACACGCAAAACGTGTGCAGGCATATCATTACTCTAGTTGCTGTCTTTGATTTTATTCCCTTTATGGCATTTCTGTCATCGTCCATCTTTTTAATAAGTTTTCTGTGGAAGCACATGAGAAGAGTTCAGAGCAATGGAACTGGAACCAGGGATCTCAACACACAAGCCCACTTGGGTGCCATCAAAGCTCTGGCTTCCTTTGCAGTTCTGTACCTCATCAGTTTTGCAGTGGACACTTCACATTCAGTACTGGTCTGGGACAGCCAACACACTTGGACAACTGTGCTCTATAATGTGAGTGTTGTGTACCCCTCCGGACATGCCATTATCCTGATATTAATTAATCCCAAACTGAAACAGGCATGGGTCAGGATGATACATCACTTCAAATGCCATTTGAGGAAAGTACCACCTTAA

>Japanese_Gecko_Tas2r17

ATGGCTACCTTATTAACAATAGTCTGCTTTGTATTTCTGGTTATGGAAACCTTCTTTGGAATGGTGGCAAATGGATCCATTGTCCTCATTAATTTCATTGACTGGTTCAGACACAGGAAACTGTCCCCGACGGACCTGATCCTGATCTGTCTGGGCTTATCCAGACTTTTGCTGCAGGCAGTAATGATTCTGGGTGTAACTGTATTTACTTTGCTTAACGATATACGGAGGAACGTATTGATCACGCTGGGCAGTATATGGGTATTTACAAACACTGTGAACCTTTGCTTTGCTGCTTGCCTCAGTGTTTTCTACTTAGCAAAAATCGCCATATTCTCCCACCCTGTTTTTCTGCAGTTCAAGCAACGATTCTCTGGGCTGGTGCCTTGGTTGCTTCTGGGCTCAGTGGTCTTCTCTGCTGTTGTGACTATTATTTTTATCACAAGCTGGAGCTATGACTTGTCTACCTGCAATCCCTACAAATCACTTTCAAGCAACAGGAGCAATGCAGAAATGAACATGCCACACGTTTGTAAGCATCTCGCTACTCTAACCGCTGTCTTCGATTTTATTCCCTTTATGATATTTCTGTCATCGTCCATCTTTTTAATAAGTTCGCTGTGGAAGCACATGAGAAGAGTCCAATGCAATGGAACTGGCACCGGGGATCTCAACACACAAGCGCACTTGAGTGCCATCAAAGCTCTGGCTTCCTTTGCAGTTCTGTACCTCATCAGTTTTGCAGTGGACGCTTCACATTCAGTACTGGTCTGGGACAGCCAACACACTTGGACACCTGTGCTCTATAATGTGAGTGCTGTGTACCCCTCCGGACATGCCATTATCCTGATATTAATTAATCCCAAACTGAAACAGGCATGGGTCAGGATGATGCATAACTTAAAATGCCGTTTGAGGAAAGTACCGTCTTAA

>Japanese_Gecko_Tas2r18

ATGTTGTGCTGTGTGCTGTTGATGGGAACTGTGAGTATCGCAGCAGAAGAAAGTCTACTCTTTTCTTTTGTGCTCAGAAAGATGGCCACCCTGTTGTCAATAACTGGCTTTGCGCTTCTCATCATGGAAACCCTCGTTGGACTGGTAGCAAATGGATTTATTGTCTTCATCAATTGTATTGACTGGTACAGAAACAGGAAACTATCCCCGACTGACTTGATCCTGATCTGTCTTGGCTCATCCAGAATCATGTGGCAGGCACTAGTAATGCTGCATGTAACTATGTTGTCCTTTTTTCTCCACACCTATGTATTGAAACGGGTACATTTAATAGTTATGATCATGTGGTTCTTTACAGAGACTATCAATCTATGCTTTGCTGCCTGTCTCGGTGTTTGGTATTTAACAAAGATCGCCATCTTCTCCCACCCTATTTTCCTCCAAGTGAAGCAAAGATTCGCTGGGCTTCTCCCATGGCTGCTTTTTGGCTCTGTGGTCTTCTCTTCTTTTATGACTATTATTAACTTCACGGAATCCTTCAGTGGCTTAGCCATTTGTGATCCCTACAAATTAGTTTTAAGCAACAGCTCTGATTCAGAAATGCAAAAGTCTCACTCTTGTATGGTCCTAGTCTTTTTACGTATTGTTTCACATTTCATCCCTTCTGTGATATTTTTGTCATCCACAGTCTTGTTGATAATATCTCTCTGGAAGCACATAAGACATTTGCAACACAATGGAACTGGAGTCAAGGATATCAACACCAGGGTTCATTTAACTGCCATCAAAGCTCTGGCTTCCTTTGCTATTTTGTACCTCTTCAGTTTGGTAGCCATCAATTTACAGTCAATGCTGGTTTGGGGAAGCAATGATCTTTCTTGGACATCTGTACTCTTTCACAATGTCAGTGATGTGTATCCCTCGGGGCATGCGGTTATCCTGATATTAATTAATCCCAAACTGAAACAGGCATGGATCAGGATGATACATCACTTAAAATGTCATGTGAGTGAAGCATCATCTTAA

>Japanese_Gecko_Tas2r19

ATGATGGTAGTGATGGACAGAGATCAGTTTTCCCCGCTGGGAGTTTTCTTTGTGATCATTTTTGGAATCGAGTCCATTGTTTCCCTATTGGGGAATGGATTCATCCTAGCTGTGTGCGGCCACAGCTGGCTGCGCAGCAAGAAGATGCTCCCTTGTGATTTCCTCTTGACCACACTGAGCCTCTCCAGGTTTCTTTTTCAGTTGGTTTCCACGAGCAGCCAATTTCTGTTTTTCAGTTCTCCAGAGACATACCTAGTTAGTGAAACAGAGCATGCATTTAAACTCTCCTGGGCTTATTTGAACATAGCCAGCCTCTGGTGTGCCACGTGGCTCAATGTCTTCTACTGTGTGAAGGTCACCAACTTTCCTCACCCGCTCTTTGCATGGCTGAAGCTGAGAATTGGGGCTCTGGTGCCCAGGTTCCTTGGAATATCTCTCCTAGCTTTCATAATCTGCTCCATTCCTCCAGTCCTGAGGTCTTTTGAAAATGAAAAATGCTGCAATCTCACAGGAAATCTACCAGAGAACACCAGCCAAAGTGAGGTTCATCACCGCCACTCTCGTATGGCTTTAAAATCTTTCCAGGTTTATTTTACTGCCATCAATTGTATCATCTGCTTAACAGCATCCCTTGTTTTGCTTCTCTCACTGTGGAGGCACATGAGAAATCTGAAGAAGAGCGGCCTCAGCACCAAGGATTTCAGCACTCAGGCCCACCTCAGCGTCATGAAGTCTTTGCTTCTCTCCCTCTTCTTCTACATTTTACATTTTACCGCCGTGATCTTTGCCCTCACTCATACTTTCAGGTATGGCAACCTTAAGCGGCTGATTTCTGATATATTCCACATTTTGTATCCCTCAGCACACTCCGTCATCTTGATAGTCACCAATCCCAAGCTGAGGAAAGCATGTACCCATGTTCTAGACCTCAGAAGTGCCTCATGA

>Japanese_Gecko_Tas2r2

ATGGAGGTTTCAGCATTCGTGATTATCAGTTCTATTTTTCTGGTTATTGAAACCCTTGTTGGACTGGTAGCAAATGGATTTATTGTCCTCATGAATTACATTGACTGGTTCAGAAGCAGGAAACTATCCCCAAATGACCAGATCTTGACCTGTCTTGCTTTGTCCAGACTTATGTGGCTGGCCTTCGTGATTCTGAATATGATTGCGGATTTCTATTCTATGGACAAGCATAACTGTCATTATGTATATCTAATGCTTCCCATCTTGTGGATATTTACAAACACTACCTCCACTTTGCTTGCCACCTGTCTCAGTGTTTTCTACTTGACAAAGATTGCCACCTTCTCCCACCCTGTTTTCCTCCAAGTGAAGCTAAGATTTTCGGGACTGGTGCCATGGTTGCTTCCGAGCTCGGTTGTCTTCTCTGCTATTACGGCTATTTTTTTAGTCACGGGCTTAAGCAATGGCTTCTCTATGTGTGATTCCAACAAATCACTTTTGAACATCACTGATTCAGGAATTAAATTGTCTGACTTATATATGTACATAGATATTCTAGCTATTGTCCCAAATTTTATTCCTTTAATGATATTTTTGTCATCGTCCATCTTGTTAATGACCTCTCTGTGGATGCACAGAAGACGTATGCAACGCAATGGAACTGGCATCCAGGATCTCAACACTCAAGTTCATTTGACTGCCATCAAAGCTCTGGCTTCCTTCGCTGTTCTGTACCTGTCTAGTTTTCTAGCAGTCATTGCTCAGGCAGTACTGACGTGGAATGATATGAGCAAAACTTGGCTTTTTATGTTGCTCAGTAACGTAACTGTCTCTTCTCCTTCTGGGCATGCCGTTATCCTGATATTAATTCATCCCAAACTAAAACAGGCATGGATCAGGATGCTGCTTCACTTCAAATGCTGCTCTACTGAAGTGCTTTCTTAA

>Japanese_Gecko_Tas2r20

ATGGTAGCAATGGACCCAAATCTGTTTTCCCCACTGGGGGTCTTTTTTCTGATCATTTTTGGAATCGAGTCCATTGTATCCGTGTTGGGGAATGGATTCGTCCTCGCCGTGAGCGGCCACAGCTGGCTCCGCAGCAAGAAGATGCTCCCTTGTGATTTCCTCTTGACCATTCTGAGCCTCTCCAGGTTTCTTTTGCAGTGGGTTACCACGGGCAGCCAAGTTGCATATTTCAGCTCCCCAGAGACGCATGTTTATAGTAAAGAACATCAGGCATTTGCCTTCTCCTGGGTTTATTTGAACACAGCCAGCCTCTGGTCTGCTACGTGGCTCAATGTCTTCTACTGTGTGAAGGTCACTAACTTTACCCACCCTCTCTTTTCATGGCTAAAGGTAAGAATTGGGGTGCTGGTGCCCAGGTTCCTCGGAATATCTCTGCTAATTTTCATAATCTCCTCTATTCGTCCAGTCATGAGGTCTTTTGAAGATGAAAAATGCCACAATCTCTCAGGAAGCCTGCCAGAGAACACCAGCCAAAGTGGGGTTCATGGCAGTAACTGTGTTGTGTTTTTAAATACTCTTCATATTTATTTTACTGCCATCAATTTTAGCATCTGCTTAACGGCATCCCTTGTTTTGCTTCTCTCTCTGTGGAGGCACATGAGAAATCTGAAGAAAGGCGGCCTCAGCACCAAGGACCTCAGCACTCAGGCCCACCTCAGCGTCATGAAGCCTTTGCTTCTCTCCCTCTTCTTCTACATTTTACATTTCGCCGCCATGATCCTCGTCTTCACTAAAGTTTACAGGTACGGCAATCTTGGGCAGCTGATTTGTGAAATATTGCTGTCTTCGTATCCTTCAGCACACTGTGTCATCTTGATATTCACCAATCCCAAGCTGAGGAAAGCATGTACCCTTGTTGCAAACCTCAGAAGAAGTGCCTCATGA

>Japanese_Gecko_Tas2r21

ATGGTAGTAATGCACAGAGATCTGTTTTCCCCACTGGGATTTTTTTTTCTGGTTGTTTTTGGAATTGAGTCCATTGTTTCCCTGTTAGGGAATGGATTTGTCCTAGTTGTGAATGGCCACAGTTGGCTCTGCAATGAGAAGATGCTCCCTTCTGATTTCCTCTTGTCCACGCTGAGCCTCTCCAGGTTTCTTTTGCAGTGGATTTCCATGAGCAGCCAATTTGTGTATTTTAGTTCTCCAGAGACATACAGAGATCATGAAAAATTATGGGCATTTACACTCTCCTGGGTTTATTTGAACACAGCCAGCCTCTGGTGTGCCACATGGCTCAATGTCTTCTACTGTGTGAAGGTCACCAACTTTGCTCACCCTCTCTTTGTGTGGCTGAAGCTGAGAATCGGGGTGCTGGTGCCCAGATTCCTTGGAATATCTCTGCTAACTTTCATAATCTGCTCTGTTCCTTCAATCATGAGGTCGTTTGAAAATGAAAAATGCTGCAATCTCACAGGAAATCTACCAGAGACCACCAGCCAAAGTGAGGCTCATCAGGACTATTCTCTTGTGTTTTTAAATACTCTCCATGCTTATTTTATTTCCATCAATTTTAGCATCTGTTTAACAGCATCCCTTGTTTTGCTTCTCTCTCTGTGGAGGCACACGAGGAATCTGAAGAAGGGCGGCCTCAGCACCAAGGACTTCAGCACTCAGGCCCACCTCAGAGTCATGAACCCTTTGCTTCTCTCCCTCTTCTTCTACATTTTACATTTTGCTGCCATGATCCTTGCCCTCAATGTTTTCACGTACGGCAAGCTCGAGCGACTGATTTGTGAGATATTCCTGTCTTCATATCCTTTAGCACACTCCATTATCTTAATATTTACTAATCCCAAACTGAGGAAAGTGTGTATCCATGTTCTAAACATCCGAAGAAGTAAGTGTCTCATGAAGAGGAAATGA

>Japanese_Gecko_Tas2r22

ATGGGAAGCGGTTCTGACAGCAGCACAACAGGCGAATTGTTTACCATCAACATGATTACCATCCTGGTTGGAACCATTTTACTGTTGGGAAGCATGTGGATGGATGTCTTCATCCTGACTGTCAGTGGCAGGGACTGGATGAAAAAGAAATGCCTGAGCACAACTGACGGGATCCTGACCTTACAAGGCAGCATCAGGATATATTTGTGGTGCACAAATCTAGTATGGCACATCCTGGATAAATTCTGCCCTTGGGTCACTGGGATAGGCTATGTTCTCGGAGTGTACACGTTCAGCTTCTGGTACTTGCTCTCTTGCAACATCTGGCTCACAACTTCCTTGTGCACCTACTACTGTGTGAAGATCGCAGACTTCAGCCACCCCTTCTTCATCCACTTGAAACTAAAACTCTCGGGACTGGTGTCGACCTGGCTCCTTGGCTCTGCAATTTTGTCTTTGGCCAGTACCCTACCCCTTGCATACACTATCTTGGAAACAGAAGATAATAGGAACTTTTCTAATTTCTTCCAAAATAAAAATGAGACTGACATCAGCAACACAGGGATGGCGTTCCGTATGTATGTTTTGCTGGCACTTGGACCTTCTGCAGCTTTTAGTATCTGTGCCATTTCTGCTGTCCTGTTACTTTTCTCTCTTTGGAGGCACCGTCAGAGGATGCTGGATAGTTGGGGGGGCCACAAGGGCCCCCAAACTGATGCCCATTTCCAAGCAGTGATCACTATATTGTTCCTTCTCGTCAACAATGTTGCTGTTTTTGTATCTATTGAACTGGTATTGTCAAGCAGCTATGGAGGAAGTTCAGTTTCTCCTTTTGGTCCTTCAACTGTTCTATATTGCTGTTTTTCAGTGGAGTCCTTGATATCAGTCTGGGGCAATAAGAAATTAAAAAACAAGTTGCGTAGGTCTTTCCATCTTGCTAGGTGCAGAGGATGTGTTCGCTGGTTGCATACACACAGTAACTGTGTTTGA

>Japanese_Gecko_Tas2r23

ATGATCTTCATCCTGGTTGGAACTATTTTGATGTTGGGAAGAACGTGGATGGATGTCTTCATCCTGACTGTCAATGTCAGGGACTGGATGGAAAAGAAACGCCTGAGCACAACTGACAGGATCCTGACCGTACAAGGCAGCATCAGGATATATTTGGGGTGCACAGAGATCATATGGCTCATCCTGGAGAAATTCTGCCCTTGGATCACTCGGGTAGGCTACGTTTTCAAAGCTTTCAAGTTCACCTTGTGGTACTTGATGTCTTGCAACGTCTGGCTCACAGCTTCCTTGTGCACCTACTACTGCGTGAAGATCGCAGACTTCAGCCACCCCTTCTTCATCCACTTGAAACTAAGACTCTCGGGACTGGCGGCAACCTGGCTCCTCGGCTCTGCCATTTTGTCTTTGGCCAGTACCCTATCCTTTGCATACATTGACTTGGAAATACGAGATAATAGCAACTTTTCTAATTGCTCCCAAAATAAAAATGTGGCTGACATCAGCCACACAGGGATGGCGTTCCGTATGTATGTTTTGCTGGGACTTGGACCTTTTGCAGCTTTTAGTATTTGTGCCATTTCTGCTGTCCTGTTACTTTTCTCTCTTTGGAGGCACCGTCAGAGGATGCTGGGTAGTTGGGGAGGCCACAGGAGCCCCCAGACCGACGCCCACTTCCAAGCAGTGATCACTATATTGTCCCTTCTCGTCAACAATGTTGTTGTTTTCATAGCTATTCTACTGCTCTTGTCAAGAGGCTTTGAACGAGATTTGATTTCTCAACGTGATCCTTCAGTTCTTCTCTATTGCTGTTTTTCAGTGGAGTCCTTAATATCAGTGTGGGGAAATAAGAAATTAAAAAACGAGTTAATTAGGTCTTTGCATTTTGCTAAGTGCAGAGGATGCTTTAGATAG

>Japanese_Gecko_Tas2r24

ATGGGGAGTGGTTCTGACAGCGGCACAACAGGCGAGTTCTTTGACCGCAACATGATTACCATCCTGGTTGGAACCATTTTACTGTTGGGAAGAATGTGGATGGATGTCTTCATCCTGACTGTAAATGGCAGGGACTGGATGGAAAAGAAACGCCTGAGCACAACTGACAGGATCCTGACCTTACAAGGCAGCATCAGGATATGTTTGGGGTGCTTGGAGTTGATACAGTCCATCCTGGAGAAATTCTGCCCTTGGGTCACTCGGATAGGCTATGTTCGCAAAGCGATTGAATTCAGCTTCCGGTACTTGATCTCTTGCAACATCTGGCTCACAACTTCCTTGTGCACCTACTACTGCGTGAAGATCGCAGACTTCAGCCACCCCTTCTTCGTCCACTTGAAACTAAGACTCTCGGGACTGGTGTCGACCTGGCTCCTCGGCTCTGCAATTTTGTCTTTGGCCGGTACCCTACCCCTTACATACTTCAACTTGGAAATACAAGATAATAGGAACTCTTCTAATTTCTCCCAAAATAAAAATGAGACTGACATCAGCCACACAGGGATGGCGTTCCGTTTGTATGTTTTGCTGGCACTTGGACCTTCTGCAGCTTTTAGTATCTGTGCCATTTCTGCTGTCCTGTTACTTTTCTCTCTTTGGAGGCACCGTCAGAGGATGCTGGGTAGTTGGGGGGGGCACAAGAGCCCCCAGACCGATGCCCACTTCCAAGCAGTGATCACGATATTGTCCCTTCTCGTCAACAATGTCATTATTTTCATATGTAATCAACTGGTATTGTCAAGAAGCTATGCAGGAAGTTCAGTCTCTGGTTTTGGTCCTTCAATTGTTCTCTATTGCTGCTTTTCAGTGGAGTCCTTGATATCAGTTTGGGGCAATAAGAAATTAAAAAACAAGTTACGTAGGTCTTTCCGTTTTGCTAAGTGCAGAGGATGTGTTAGCTAA

>Japanese_Gecko_Tas2r25

ATGACTATCATCCTGATTATGACTATTTTACTGTTGGGAAGCACGTGGATGGATGTCTTCATTCTGACTGTCAATTGCAGGGACTGGATGGAAAAGAAACGCCTGAGCACAACTGACAGGATCCTGACCTTACAAGGCAGCATCAGGATATGTTTGGGGTGCTTGGAGTTGATACAGTCCATCCTGGAGAAATTCTGCCCTTGGGTCACTCGGATAGGCTATGTTCTCGAAGTGTACACGTACATCTCGTGGTACTTGACGTTTTACAACGTCTGGCTCACAGCTTCCCTGTGCACCTACTACTGCGTGAAGATCGCTGACTTCAGCCACCCCTTCTTCGTCCACTTGAAACTAAGACTCTCGGGACTGGTGTCGACCTGGCTCCTCGGCTCTGCAATTTTGTCTTTGGCAAGTACCCTGCCCCTTGCATACACCACCATGGAAATACGAGATAATAGGAACTTTTCTAATTTCTCCCAAAATAAAAATGAGACTGACATCGCCCGCACAGTTTTGCTGGGACTTGGACCTTTTGCTGCTTTTAGTATCTGTGCCGTTTCGGCTGTCCTGTTACTTTTCTCTCTTTGGAGGCACCGTCAGAGGATGCTGGGTAGTTGGGGGGGCCACAGGAGCCCCCAGACTGATGCCCACTTTCAAGCAGTGATCACTATAGTGTCCCGTCTCGTCACCAGTGTTGTTGTTTTCATATCTATTCAACTGTTCTTGTCAAGCGGCTATGGAGGAAATTCAATTTCTCAACTTGGTCCTTCAATTGTTGCCTATTGCTGCTTTTCAGTGGAGTCCTTGATATCAGTTTGGGGCAATAAGAAATTAAAAAATGAGTTACGTAGGTCTTTCTGTTTTGCTAAGTACAGAGGATGTGTTAGCTAG

>Japanese_Gecko_Tas2r26

ATGTTGTGCTGTGAACTCTTGATGGAAAGTACGAGCATCGCAGCAGAAGAAACTCTACTCTTTTCTCTCGTACTCAGAAACATGGCCACCTTCTTGTCGATAACTGCCTTTATACTTCTCATCATGGAAATCCTCATTGGAACGGTTGCGAATGGATTTATTGTCCTCATCAGCTGCATTGAGTGGATCAGAAGCAGAAAACTATCCCCGATCAACTTGATCCTGATCTGTCTTGGCTCATCCAGACTCATGTGGCAGGCCATAGTAATGCTGCATGTAACTATGTTTTCCTTTTTCCCCCACACCTATATATTGAAACAGGTACATTCAATAATTACAGTCATATGGTTCTTTACAGACACTGTCAATCTATGCTTTGCTGCCTGTCTCGGTGTTTGGTATTTAACAAAGATCGCCATCTTCTCCCACCCTGTTTTCCTCTACGTGAAGCAAAGACTCTCTGGGCTGCTCCCATGGCTGCTGCTTGGCTCAGTGGCTTTCTGTGCTTTTATGACTGTTATTGTCTTCGCAAGATCTTTCAGTGACTTGGCCATTTGTGATCCCTACAATTTACTTTTAAACAACAGCTTTGATTTAGAAATTCAAAAGTCTCGCACCTGTATGGATATAAATATTTCACGTACTGTTCCAAAAGTCATCCCTTCTGTGATATTTTTGTCATCCACGGTCTTGTTAATAATATCTCTATGGAAGCACACAAGACATTTGCAACACAATGGAACTGGAGTCAGGGATATCAACACCAATGTTCATTTAAGTGCCATCAAAGCTCTGGCTTCCTTTGCTATTTTGTACCTCTCCAGTTTGGTAGCCATCAATTTACAAACAGTGCAGGTTTGGGGAAGCAATCATGATTCTTGGAGAACTGTACTTTTTCACAATGTCAGTGTTGTGTATCCATCTGGGCATGCTGTTATTCTGATATTAATTAATCCCAAACTGAAAAAAGCATGGGTCAGGATGATACATCATTTAAAATGTTGTGTGAGTGAAGCACCATCTTAA

>Japanese_Gecko_Tas2r27

ATGGCCACCTTGTTGTCGATAATTGGCTTTGCCCTTCTCCTCATGGAAACCCTTGTCGGGATGGTAGCAAATGGATTTATTGTCCTCATCAGCTGCACTGAGTGGATCAGAAGTAGGAAACTATCCCCAACTGATCTGATCCTGACCTGCCTTGGCTTGGCCAGATTTGCGTGGCAGGCCATAGTGATTCTGGAAGTAACTATGTATTCTTTTTTTCTGCGCATTTATCAATTGAATCATTGCCGTTTAGTGCTTAACATGTTGTGGTTATTTACATACAACGTCAACCTTTGGTTTGCCGCCTACCTCAGCATTTTGTACTTTGTGAAGATCACCACTTTCTCCCACCCTCTTTTCCTCCAAGTGAAGCAAAGATTCTCCAGGCTGCTCCCATGGCTGCTTCTCGGCTCAGTGGCCATCTCAGCTTTCATGACGATGATTATGACTGTTATCACAGTATTGAGCAGTGACTTAATCAATTGTGACCCCTACAAATTACTTTTAAATGATAGCTTTGATTTAGAAATTAAAAAGGCTCCTTCTTGTATGGACTTCGCCATTTCGGTTATTGTTCCAAATGTCATCCCTTCTGTGATATTTTTGTCATCCACAATCCTGTTAATAATATCTCTTTGGAAGCACGCAAGACATTTGCAACACAATGGAATCAGCACCACAGATCTCAACACCAGTGTCCACTTAAGTGCCATCAAAGCTCTGGCTTCTTTTGCTTTTTTGTACCTCTTCAGTTCAGTAGCAATCCATTTACAGGCAATGCCAATTTGGAGAAGCATTGATCACTCTTGGACAGATGTGCTTTTTCAGACTATGAGTGCTGTGTATCCCTCTGGTCATGCGGTTGTCCTGATATTAGTCAATCCCAAGCTGAAACAGGCATGGGTCAGGATGATGCACCACTTAAAATGTCATGTGAGTGAAGCACCATCTTAA

>Japanese_Gecko_Tas2r28

ATGATTGTCATCCTGGTTGGAACTATTTTGGTGCTGGCAAGAACGTGGATGGATGTCTTCATCCTGACCGTCAATGTCAGGGACTGGATGGAAAAGAAACGCCTGAGCACAGCTGACAGGATCCTGACCTTACAAGGCTGCATCAGGATATATTTGGGGTTCCCGGATATAATATGGCAAATCCTGGAGAAATTCTGTCCTTGGGTCACTTGGATACACTACGTTTTCAAAGCGCTCATATTCAGCTTCTGGTACTTGCTCTCTTGCAACGTCTGGCTCACAACTTCCTTGTGCAGCTACTACTGCGTGAAGATCGCAGACTTTAGCCACCCCTTCTTCGTCCACTTGAAACTAAGGCTCTCGGGACTGGTGTCGACCTGGCTCCTTGGCTCTGCAATTTTGTCTTTGGCCGGTACCCTGCCCCTTGCATACACCACCATGGAAATACGAGATAATAGGAACTTTTCTAATTTCTTCCAAAATAAAAATGAGACTGACATCAGGCACACAGGGATGGCGTCTCGTATGTATGTTTTGCTGGGCCTTGGGCCTTTTGCAGCTTTTAGTATCTGTGCCATTTCTGCTGTCCTGTTACTTTTCTCTCTTTGGAGGCACCGTCGGAGGATGCTGGGTAGTTGGGGGGGCCACAGGAGCCCCCAGGCCAACGCCCACTTCCAAGCAGTGATCACGATATTGTCCCTTCTCGTCAACAATGTTGTTGTTTTCATAGCTATTCTACTGCTCTTGTCAAGAGGCTTTAAAAGAAATTTGATTTCTCAACTTGGTCCTTCAATTCTTCTCTATTGCTGTTTTTCAGTGGAGTCCTTGGTATCAGTGTGGGGAAATAAGAAATTAAAAAACGAGTTGCGTAGGTTTTTGTATTTTGCTAAGTGCAGAGGGTGTGTTAGCGAGTTAAAAAGAATTGCTCACTGCTAA

>Japanese_Gecko_Tas2r29

ATGGCCACCTTGTTGTTGTTAACTGGCTTTGCCCTTCTCATCATGGAAACACTCGTCGGAATGGTAGCAAATGGATTTATTGTCCTCATCAGCTGCATTGACTGGATTAGAAGCAGGAAACTATCCCCCACTGAACTGATCCTGACCTGGCTTGGCTTGGCCCGACTTGCATGGCAGGCTGTAGTGATTCTGGATGTAATAATGCTGTTCTTTTTTCTGCACACTTATCTATTGGATCATGTCTTTTTAATGATTAACATGGGGTGGTACTTTATATACAATATCAACCTTTGGTTTGCTGCCTGCCTCAGTGTTTTGTACTTCGTGAAGATCACCACTTTCTCCCACCCTCTTTTCCTCCGAGTGAAGCAAAGATTCTCTGGGCTGCTCCCATGGCTGCTTCTCGGCTCACTGGCCTTCTCTGCTTTCATGACTATGACTATTACTGTTATCACAGTATCGAGCAGTGGCTTAACCAGCTGTGATCTCTACAAATTATTTTTAAACCATAGCTTTGATTCAAAAATTAAAAAGCCTCATACTTGTATGGATTTTGCCATTTCAGTTATTGTTCTGAATGTCATCCCTTCTGTGATATTTTTGTCATCCACAATCCTGTTAATAATATCTCTTTGGAAGCACACAAGGCTTTTGCAACACAATGGAATCAGCAACAAGGATCTCAATACCAGGGTTCACGTAACTGCCATCAAAGCTCTGATTTCCTTTGCTATTTTGTACCTCTCCAGTACTGTAGCCATCATTTTACAAGCAATGCCTGTAATTATTGATCGCTCTTGGACATCTATGTTTTTTCAAACAATGAGTTGTGTATATCCCTCTGTGCATGCTGTGGTCCTGATATTAATTAATCCCAAACTAAAACAAGCATGGGTCAAAATGATACATCACTTAAAATGTCATGTGAGTGAAGCACCATCTTAA

>Japanese_Gecko_Tas2r3

ATGGCTACCTTGTTGTCAATAACTGGCTTTGCACTTCTCATCATGGAAACCCTTGTTGGAATCATAGCAAATGGATTTATTGTCCTCATCAGCTGCATTGAGTGGATCAGAAGCAGGAAATTATCCCAGACTGACTTGATCCTGATCTGTCTCGCCTCATCCAGACTCCTGTGGCAGGCACTAGTAATGCTGCGTGTAACTATGCTTTTCTTTCCCCCCCTCATCTACAGATTGAAACTGGTACATTTAACAGTTACGATCATGTGGTTCTTTACAGACACCATCAATCTTTGCTTTGCTGCCTGTCTCGGTGTTTGGTATTTCACAAAGATCGCCATCTTCTCTCATCCTCTTTTCCTCCACGTGAAGCAAAGAATCTCTGGGCTGCTCCCATGGCTGCTGCTTGGCTCAGTGGTTTACTCTGTTTTTATGACTGTGACTGTTTTCACAGTATTGAACAGTGACACAACCATTTGTGATCCCTACAAATCATTTTTAAACAATACCTATGATTCAGAAATTCAAACGCCTCATTCTTGTATCGATCTACTCTTTTTTCGTATGGTTCCAACAGTCATCCCTTCTGTGATATTTTTGTCCTCCGCAATCTTGTTAATAATATCTCTGTGGAAGCACACAAGACATTTGCAACAAAACGGAATTGGAGTCAGGGATCTCAGCACCAACGTTCATTTAAGTGCCATCAAAGCTCTGGCTTCCTTTGCTATTTTGTACCTCTCCAGTTTGGTAGCCACCAATTTAGTGCTGGTTCGGAGAAGCAATGATGGTTCTTGGAGATCTGTACTTTATAATGTCAGTGCTGCGTATCCCTCTGGGCATGCTGTTGTCCTGATATTAATTAATCCCAAACTGAAACAGGCATGGGTCAGGATGATACATCACTTAAAATGTTGTGTGAGTGAAGCACCATCTTAA

>Japanese_Gecko_Tas2r30

ATGGAGGTTTCGGCATTCGTGATTATCAGTTCTATCCTTCTGGTTATTGAAACCCTTGTTGGACTGGTAGCAAATGGATTTATTGCCCTCATGAATTACATCGACTGGTTCAGAAGCAGGAAACTATCCCCAAATGACCAGATCTTGACCTGTCTTGCTTTGTCCAGACTTATGTGGCTGGCAGCCGTGATTCTGAATATAACTGCGCATTACTATTCTATGGACAAGCATAATTGTCATTATGTATATCTAATGCTTCCCATCTTGTGGATATTTACAAACACTTCCTCCACTTTGTTTGCCACCTGTCTCAGTGTTTTCTACTTGACAAAGATCGCCACCTTCTCCCACCCTGTTTTCCTCCAAGTGAAGCTAAGGTTTTCGGGACTAGTGCCATGGTTGCTTCCGAGCTCGGTTCTCCTCTCTGCTTTTACGGCTATTTTTTTAGTCACAGGCTTGAGCAATGGCTTCTCTATGTGTGATTCCAACAAATCACTTTTGAACATCACTGATTCAGGAATTAAATTGCCTGACTTATATATGTACATAGATATTCTAGCTACTGTCCCAAATCTCATTCCTTTAATGATATTTTTGTCATCGTCCATCTTGTTACTGACCTCTCTGTGGATGCACAGAAGACGGATGCAACGCAATGGAACTGGCATCCAGGATCTCAACACTCAAGTTCACTTGACTGCCATCAAAGCTCTGGCTTCCTTCGCTGTTCTGTACCTGTCTAGTTTTCTAGCAGTCATTGCTCAGGCAGTACTGATCTGGAATGATATGAGCGGCACTTGGCTTTTTATGCTGCTTAGTAACGTAACTGTTTCTTCTCCCTCTGGGCATGCCGTTATCCTGATATTACTTAATCCCACACTAAAACAGGCATGGATCAGGATGCTGCTTCACTTAAAATGCTGCTCGAGTGAAGTGCCTTCTTAA

>Japanese_Gecko_Tas2r31

ATGTCGGAATTTGATGCACTTAACGTTTTGTGTCTCGTCATTGTAGCCATTGTGATTGTCGTCGGGGTGATGGGAAATGGATTCATCGTCCTCGCTAACGGCCTCGACTGGATCCGAAGCAAAACGATGCCCCCTTCGGACATGATCCTGACCGCACTGAGTCTGTCCCGCCTCCTTTTCCTCGGGCTGGTACTGGCTGTCCACTGCTTGTTCTTCCTTGATGTCGACAATCCCAAGAGCTTACCCGAATCCCTCATTTTCTTCTGGGGATTTGCCAACGCCACTACCCTTTGGATGGCCACCTGCCTCGCAGTCTTCTACTGCATGAAACTCGTCAATCTCCCCCAGGTGTTCTTCGTGAAAATGAAACTGGGTCTCTCCAGGCTGGTTCCAAGGCTGCTCCTGGGTTCAGTGCTGGTCTCCTTCATCACTTCCTTTCCCATCATCTTCTTCGAAAAGTGTAGCCCCTGTTGCAATGAAACGAGGGTCGTTCGGGGTAACCGGAACACGACATGCCCCCAAAAGGTACTTTCGGGTATTATATACATCACGGGCAGTTCCCCGTCTTTCGTTATCGTTTTAGCTTCTTCGGTCCTACTGATTCGCTCCCTTCTTCACCATGCTCGGAAGATGCGACTGAACATGGGGGGCGTCAAGGACCACAGGATGGATGTTCACATCAAAGCCGTTAAAACCCTGGTCTCCTTTGTTATCCTCTTCACCGCGAGCTTTGTCGCTGTGGTCTCGCTGGCTATGTTCACAAGTCCTTGGACAATCGTCACATCAACGGTGGTGATCATCGTGTGTAACTCGGGACACTCTGTGATGCTGATTTCCATGAATCCTAAACTGAAGCAGCCACTAATCAGGAGTCTGCGGGGCAGCTTAGGCCGCACCCTGAGGAGAATATCAACGTCCTGCCCCAAGACTGCAGTAGGAAATTGCTCCTGTTAG

>Japanese_Gecko_Tas2r32

ATGTCCCTGAATGCTTTTATCGTCACAGTGAGTTGCATCAACGGGATGAAAAGCAAACAGCTGAAATCTATTGATAAAATCTTGGCTGCTCTGGGCATCACCAGGTTTTGCTACCTGGGCATGTTCCTGGGCAAGATTTTCTGGATGTCGATCTCTTCTCGAGTTTTCGAAGTGACTGCCCTGTACCAAATGTTCAAAGCGGCCATCTGGTTTCTGACCTGCGTCTGCTTCTGCTTCTCAGCCTGCCTGTGTTCGTTTTACTGCATAAAAATTGCCAACTTTGGGCACCGCCTGTTTGTTTACCTGAAGTTGAGAATCTCCAGACTGGTACCCTGGATGCTCCTGGTTTCAGTGCTTGGATCACTGCTGAACTCTTTCCCCTTTTTCAATGGCATTTATAATATAGCTTGCAAAAACAGCACTGGTTCCGGTGAATCGGCGAACCGAACGCTCGAAGATTTCACTTGGGAAACAAATTTGCTTAGTCTGTTTGCGTATTGCGGCGTGGGCTTTTCAGTGGTGTTCTCCATCTCCGTGGCCTCGTCCTGCCTCTTGCTATTTTCTCTGTGGAGACACGCCCACCTGATGCAGAACGGCTCACCCAGTTTCAGCAAGCTGAGCATGGCTGCCCATTTCCAGGCGGTGAAAACGATAATGTCGCTCTTGATCGTTGATAGTGTTAATTTCATAGGTCTGATGATCCTGCTGTCCAATGTCTTTTCGGAGAGAGGTCCTACAAACCGACTCATCACGATCATCGTCTATGTATGTCCCTCCGCGCAATCACAGATTGTCATCTGGGGCAACCCCAAGCTGAAAAGAGCATTCATTAGACTAACAAATTGTATCAGGCACATGTCCTTGGTTTGA

>Japanese_Gecko_Tas2r33

ATGCTGGTGATCGTAGGGACCGAGACAGTTCTTGGTGCTTGGATAAATGCTTTCATTATGACTGTCTGCTGCATCAACAGACTTAAAAACAAATCCCTCAGTGCTGCTGACCACATCTTGATGGTCCTGGCCACCAACAGGTTTTGCTTCTTGATCCTAGGAATGTTACGGGTACTGTGTAGGACCCTCAGCCCTCTGATCTACTACAAAGACTTTGTCTATCGAGGACTGAAAGCTATCCTCTGGTTCTTCATTTCTTCCAACCTCTGGCTCGCCGCTTGCCTGTGCCTCTTCTACTGTGTGAAGATTGCCAGTTTCAGCCATCCTTTATTCATCTCCCTGAAGCTAAAAATAACCAGGGCAGTTCCAGCGCTGCTCCTCGGCTCCGAGCTTTTATCGTTAGTCAACACCATCCCTTTCTTCAGTCTGATTTACACGGTGCAGTGCAACGTCTCAAACGACACGGCGTCAGGAAACATCAAGAGTCAGATCGACATGCACACCAATTGGCGCAATCTATTCTTCTTGTGTGGCTTTGGCTTTTCTTTGGTTTTTGCCATTTTTGTCACTTCGGCCGCCCTCTTGCTCGTTTCCCTTTGGCGGCACGCTCGGCAGCTGAGAGGCGTTTTGTCCGGCTACACGAGCCCCAGGATGGCCGCTCACGTCCGGGCAGTGAGAGTAATCACATACTTCCTGGTTACTTACCTTGTCAACTTTGTGGCTTTGATGCTCTTACTGACAGATGTGTTTTCAGAGGACAGCGCCCTAGACTTCCTCTGTACGATTGTTCTAAATGCTTGCCCTTCAGTACATTCCATTACCCTAATTCTGACCAATCCCAAATTCAAAAAAACATTCCTTCAGATGCTGCGCCACGGAGGCTGCAAGTGGTGA

>Japanese_Gecko_Tas2r34

ATGTCCTCCTTACTGTCAATTGGCTTGGCACTTCTGGTCTTGGAAACCCTCATCGGCATGGTGGCAAATGGATTTATCGTCCTCTTCATTTGCACTGACTGGTTCAGAAGCAGGAAAGTATCCCCAACTGATCTGATCTTGTGCTGCCTTGGTCTGTTGAGATTTATATGGCAGGTGACAGTGTTCCTCATCGTAATTATGACTTCCTTTTTCAAGTGCACCTTCGTACAGGTTAATGTATGGTTAGTGTTTGAAATAATGTGGGTATTTATGAACACCGTGCACCTTTGGTTTGCTGCCTGGCTCAGTGTGTTGTACTTCGTAAAGATCACCATGTTCTCCCACCCCGTTTTCCTTCAAGTAAAGCAAAGATTCTCTGGGCTGGTCCCATGGCTGCTTCTTGGCTCAGTGGTCTTCTCTGCTGCTGTGACCATTCTTATCACAGCCTCGACCTATATCTTCCCCATCTGCCACCACTACAAATCGCTTTCACGCAACAGCAGTGATTCAGAATGTAAGGCACCCCACTCCTGTAGCGACTTTGCTATGCTATCCATTGCCCCCAGTTTAATTCCGTTTTTGTTATTTTTGTCATCATCCATCTTGCTAATAATTTCTCTTTCAAAGCACTTAAGGCATTTGCAGCACAATGGAGTTGGTGTCATGGATCTCAACACACAAGCGCACTTGAGTGCCATCAAAGCTCTGGCTTCCTTCGCTGTTTTGTACCTTTCCAGTTTTGTAGCAGCCAATTCACTAATTATAATGCCCTGGATAGCCTACGAGCGCCATTGGACATCTACACTTGTTGAAAATGCGAGCGCTATATATCCTGCTGGGCACGCCGTTATCCTGATATTAATGAACCCCAAACTGAAACAGGCAGTGATCCAGATGCTGCATCACCTAAGATGCCGGTTGTGA

>Japanese_Gecko_Tas2r35

ATGGCAATGATTGACAGACATCTGTTTTCCCCACTGGGAGTTTTTTTTCTGGTTGTTTTTGGAATCGAGTCCATTGTTTCACTGTTAGGGAATGGATTCATCCTAGTTGTGAATGGCCACAGCTGGCTTCACAACAAGAAGATGCTCCCTTCTGATTTCCTCTTAACCACTCTGAGCCTCTCCAGGTTTCTTTCGCAGTGGGTTTCCATGACCGCCCAATTTATGTATTTCAGCTCTCCAGACACATATATCTATAGTAAAGAACAGCCTGCATTAAAGTTCTCCTGGTTGTATTTGAACACAGCCAGCCTCTGGTGTGCCACGTGGCTCAATGTCTTCTACTGTGTGAAGGTCACCAACTTTGCTCACCCTCTGTTTTCATGGCTGAAGCTAAGAATTGGGGTGCTGGTGCCCAGATTCCTTGGAATATCTCTCCTAGCTTTCATACTATGCTCTATTCATCCAGTCATGATGTATTTTGAAGATGAAAAGTGCCACTATCTCACAGGAAACCTGCCAGAGAACACCAGCAAAAGTGAGGCTCATGGCAGTGAGTGTGTTATGTTTTTAAGTCCTCTTCTGATTTCTTCTACTGCCATCAGTTTCAGCATCTGCTTAACGGCATCTGTTGTTTTGCTTCTCTCTCTGTGGAGGCACAAGAGGAATCTAAAGAAGGGCGGCCTCAGCCCCAAGGACTTCAGCACTCAGGCCCACCTCAGAGTCATTAACCCTTTGCTTCTCTCCCTGTTCTTTTATGTTTTACATTTTGCTGCCATAATCCTTATCTTCACTGGTGTTTTCAAGTATGGCAAGCTTCAACGGCTGATTTGTGAGATATTTCTATTTTTGTATCCTTCAGCACACTCCATCATCTTGATATTCACCAATCCCAAACTGAGGAAATTGTGTACCCGTGTTCTAAATCTCTGA

>Japanese_Gecko_Tas2r36

ATGGCAGCAATGGACAGAAACATGTTTTCCCCACTGGGTATATTTTTTCTGATCATTTTTGGGATTGAGTCCCTTGTTTCCTTGTTAGGGAACGGATTCATCGTAGTCGTGAATGGACAGAGCTGGCTACACAGCAGGAAGATGCTCCCTTGTGATTTCCTCTTGACCACTTTGAGCCTCTCCAGATTTCTTTTGCAGTGGGTTATCGTGAGCAGCCAATTTGTGTATTTCAGCTATTTAGAGACATATATAGATAGCAGAAAACATCAGGCATTCTCCCTTACCTGGATGTATCTGAACACAGCCAGCCTCTGGTGTGCCACGTGGCTCAGTGTCTTCTACTGTGTGAAGGTCACCAACTTTGCCCACCCTCTCTTTGTATGGCTAAAGCCAAGAATTGCGGTGCTGGTGCCCAGATTCCTTGGAATATCTCTCCTAGCTTTCATAATCTGCTCCGTTCATCCAGTCATGAGGTCTTTGGAAAATGAAAAATGCTGCAATCTCACAGGAAATCTAGTGGGAAACACCAGCCAAAGTAAGACTTGTGGCAGCCATTTTCTTATGTTGTTAAGTCCTCTCCAGTTCTCTTTTACTGCCATCAGTTTCAGCATCTGTGTATCCGCTTCTATTCTTTTGCTTCTCTCTCTGTGGAGACACACGAGGAATCTGAAGATGGGTGGCCTCAGCACCAAGGACTTCAGCACTCAGGCCCACCTAAGTGTTATGAAGCCTTTACTCCTTTTGCTCTTCTTCTACATTGTACATTTTGTTGCCATGATCATTACTATGGGTGATATTCTCCACTATGGCAAACTTGAGCGGCTGATTTCTGACATTGTCCTGACTTCATATCCTTCTGCACACTCTGTCATCTTGATATTCACCAATCCCAAACTGAAAAAAACGTGCATCCATATGCTAAACCTGAGAAGAGGTGCTTCCTAA

>Japanese_Gecko_Tas2r37

ATGGTACAAAGCTCCATGGGCCACCCACAGGGACAGAAGAAAGACAGCACCGCCATGGAATCTGCTATGCCGAGAGACAGGGCACTGGTACCCTCTTCCCTGGTGACTTTCTACCTTGCTGTTACAGGCACTGCCTACCTCTTGGTCATCATGACCAATGGGTTTATCATTGTGGTGAATCTCTCTGATTGGTCTAAAGGAAGAGGTTTGATGCCAAATGACAAGATCCTGTCCAGCCTCGCGCTGTCCAATCTTTGTTACTCCACTTCGTTTATCACAGACTATTTTTTCTCTCTCATCTGGGACGAGTTCTACAGCGTGTTCTACAACCTGCAAAGAGTCTTCATGACCCTTGATATCGCCACGAGCTTCTCGAGCTTCTGGTTCACTGCCTGGCTCAGCGTCTTCTACTGCGTGAAGATCGTCAGCTTCAAGCGGCTGCTTTTGCTGAAGCTGAAATTGCAGTTTCCCGGCCTCGTGCGTTGGCTCCTCCTTGGCTCAGCTTTGGTTTCTTTGGGAGCTGCGCTCCTGTTTCAATTGGCTTTTATGGTAGTATCACATCGGAAACCAGCCACCAATCAAACCACCAGTCTTTCCCCTGCCTCTAATTGTACGCGCAACCATGTGAATGCAGGAATCATACTCGTTCACATGTCCCCTGTTTACAGAGTCCTCATCATTATCATTGGCTGCTCCATTCCCTTGATGGTGGTGGTGTTCTCTTCGGTCCCGGTTCTTTGGTCCCTTTTCAGGCACACCCAAAAGCTGGAACAAACGTTGTCTCCTTCGCACTTGGAAGCTCACGTCAAGGCCGCTAAGGCCGTGCTGACACTCCTGCTGTGCTACATCATCTCGTTTGTTTGTCAGACATTAGTCAGAGCGGAGATCTACCGCAACTGGTATTACCCGTATTTTCTGTGTCTGATGGTGCAGCTGGCTACCTTATTGGCCCAAAGCACCATCCTAATCCGAAGTAATTCCAGGCTGATGCAAACGGCCACACATCTTCTTCCCTGCCCGCTAGGCCGACAGAGAAAAGGGAACAGCCGGAAGCCCACAGAACTGCAGGAAGTATAA

>Japanese_Gecko_Tas2r38

ATGGCCACCTTGTTGTCAATAACTGGCTTTGCAATTCTGGTCATGGAAACCCTCATTGGAATGGCAGCAAATGGATTTATTGTCCTCGTCAGCTACATTGAATGGATCAGAAGCAGGAAGCTATCCCCGACCGACCGGATCCTAATCTGGCTTGGCTTGGCCAGACTCATGTGGCAGGCAATAGTAATGCTGCATGTAACGATGCTTTCCTTTTCCCCCCACACCTATATATTGAAACAGATACATTTTGGAGTTGCGATGATGTGGTTCTTTGCAGACACTGTCAATGTTTGCTTTGCTGCCTGTCTTGGTGTTTGGTATTTAACAAAGATCGCCGTCTTCTCCAACCCTGTTTTCCTCCAAGTGAAGCAAAGAATATCTGGGCTGCTCCCATGGCTGCTGCTTGGGTCAGTGGCTTTCTCTGCTTTTATGACTATGACTATGACTGTTTTCGCAGTATCGAGCAGTGACATAACTATTCGTGATCCCTACAAATTACTTTTAAACAATAGCTTTGGTTCAGAAATACAAAAACCTCACTCTTGTATGGACCTAGTCTTTTTACGTATTGTTCCAACTGTCATCCCTTCTGTGATATTTTTGTCCTCCACAATCTTGTTATCAGTGTCTCTGTGGAAGCACGCAAGACATTTGCAACAAAACGGAATTGGAGGCAGGGATCTCAACACCAACGTTCATTTAAGTGCCATCAAAGCTCTGGCTTCCTTTGCTATTTTGTACCTCTCCAGTTTGGTAGCCACCAATTTACAAACAGTGCTAGTTTGTAGAAGCAATGATGGTTCCTGGGCATCTGTATTTTTTCACAATGTCAGTGCTGCGTATCCCTCTGGGCATGCGGTTGTCCTGATATTAATTAACCCCAAACTGAAACAGGCATGGGTCAGGATGATACATCACTTAAAGTGTCTTGTGAGTGAAGCAACATCTTAA

>Japanese_Gecko_Tas2r39

ATGGTAATGATGGACAGAGATCTTTTTTCCCCACTGGAAGTCTTTTTTCTGATCATTTTCGCAATTGAGTCCATGGTTTCCCTGTTGGGGAATGGATTCATCCTAGCCGTGTGCGGCCACAGCTGGCTGCGCAACAAGAAGATGCTCCCTTGTGATTTCCTCCTGACCACTCTGAGCCTCTCCAGGTTTCTTTCGCAGTGGGTTTCCACGAGCAGCCAATTTGTGCATTTCAGCTCTCCAGAGATGTATATATACAGTACAGCAGAGCAGGCATCTGCGATCTCCTGGAATTATTTCAACACAGCCAGCCTCTGGTGTGCCACATGGCTCAATGTCTTCTACTGTGTGAAGGTCACCAACTTTCCTCACCCGCTCTTTGCATGGCTGAAGCTGAGAATTGGGGCTCTGGTGCCCAGGTTCCTTGGAATATCTCTCCTCGCTTTCATAATCCTCTCTATTCATCCACTCATGAGGTATTTTGAAGATGAAAAATGTCACAATCTCACAGGATACCTGCCAGAGAACACCAGCCAAAAGGAGGCTCATGACATCAACTGTGTTAGGTTTTTAATTGCTCTTCAGATTTCTTCTATTGGCATCAGTTTCAGCATCTGCTTAACGGCATCTGTTGTTTTGCTTCTCTCTCTGTGGAGGCACAAGCAGAATCTGAAGAAGGGTGGCCTCAGCACCAAGGACTTCAGCACTCAGGCCCACCTCAGCATCATGAAGCCTTTGCTTCTCTCCCTTTTCTTCTACATTTTACATTTCGCCGCCACAATCCTTGCCCTCACTAATTCTTTCAAGTATGGCAAGATTGAGCAGCTGATTTGTGAGATATTCCAGTTTTCGTATCCTTCAGCACACTCCGTCATCTTGATAGTCACCAATCCCAAACTGAGAAAAGCGTGTACCCATGTTCTAAACCTCAGAAGACACACATCATGA

>Japanese_Gecko_Tas2r4

ATGGAGGTTTCCGTATTCATGATTATCAGTTATATCCTTCTGGTTATTGAAACCCTTGTTGGACTGGTGGCAAATGGATTTATTGTCCTCATGAATTACTTTGACTGGTCCAGAAGCAGGAAACTATCCCCATATGACCAGATCTTGACCTGTCTTGGCTTCTCCAGACTTGCATGGCTGGCAGTTGTGATTCTGGGTACAACTATGGATTGCTTTTCTATGGGCCAGCATACTTGTCATTATGCCTATCTAATGCCTCCCATCTTGTGGATGTTTACAAACTCTGCCACCATTTGGTTTGCCACCTGGCTCAGTGTTTTCTACTTGGCAAAGATCGCCACCTTCTCCCACCCTGTTTTCCTCCAGGTAAAGCTAAGATTTTCAGGACTGGTGCCACGGTTGCTTCTGGGCTCGGTTGTCTTCTCTGCTATTATGGCTATCACAAGCTTGAGCAATGGCTTCTCTATGTGTGATTTCGACAAATCACTTTTGAACATCAGTGATTCAGAAATTAACTTCACTGACTCCTATGCATACATAGATTTTCTAGCTGCTGCCCCAACTCTCATTCCCTTAATAATATTTTTGCCATCGTCCATCTTGTTAATGGCCTCTCTGTGGATGCACAGAAGACGGATGCAATGCAATGGAATTGGCATCCAGGATCTCAGCACTCAAGCTCACTTGACTGCCATCAAAGCTCTGGCTTCCTTCGCTCTTCTGTACCTGTCTTGTTTTTCAGCACTCATTGCTCAGGCAGTACTGATCTCAAATAATATGAGTGACACTTGGCTTTTTATGCTGCTTAATAACGTAGCTGTTTCTTCTCCCTCTGGGCATGCCGTTATCTTGATATTAATTAATCCCAAACTAAAACAGAGATGGGTCAGAATGCTGCTTCATTTAAAACGCTGCTCTAGTGAAGTGCCTTCTTAA

>Japanese_Gecko_Tas2r40

ATGATGGTTATGATGGAAAGAGATCTTTTCCCCCCACTGGAAGTCTTTTTTCTGGTCGTTTTTGGAATCGAGTCCATTGTTTCCCTGTTGGGGAATGGATTCATCCTAGCCGTGAACAGCCACAGCTGGCTCCGCAGCAAGAAGATGCTCCCTTGTGATTTCCTCCTGACCACTCTAAGCCTCTCCAGGTTTCTTTTGCAGTGGGTTTCCACAAGCAGCCAGTTTGTGTATTTCAGTTCTCCAGAGACATACATACATAGTGAAAAACGGCAGGCTTTTTTGCTCCCCTGGGTTTATCTGAACACAGCCAGCCTCTGGTGTGCCACGTGGCTCAACGTCTTCTACTGCGTGAAGGTCACCAACTTTCCTCACCCGCTCTTTTCATGGCTGAAGCTGAGAATTGGGGCTCTGGTGCCCAGATTCCTTGGAATATCTCTCCTCGCTTTCATAATCTGCTCCATTCCTCCAGTCCTGAGGTCTTTTGAAAATGAAAAATGCTGCAATCTCACAGGATACCTGCCAGAGAACACCAGCCAAAAGGAAGCTCATGACAGTCTTAGGTTTTTAATTATGCTTCAGATTTCTTCTACTGCCATCAGTTTCAGCATCTGCTTAACAGCATCTGTTGTTTTGCTTCTCTCACTGTGGAGGCACAAGAGGAATCTGAAGAAGGGCGGCTTCAGCACCAAGGACCTCAGCACTCAGACCCACCTCAGCGTCATGAAGCCTTTGCTTCTCTCCCTCTTCTTCTACATTTTGCATTTCGCCGCCACGATCCTTGCCTTCAACGATATTTTAAGGTACGGCAAGCTTCAGCATCTGATTTCTGATATATTCCTGTCTTCGTATCCTTCAGCACACTCTGTCATCTTGATATTCACCAATCCCAAACTGAGGAAGGTGTGTACCTATGTTCTAAACCTCCGAAGAAGTACCTCATGA

>Japanese_Gecko_Tas2r41

ATGGTTGTGATGGACAGAAATCTGTTTTCCCCACTGGGAGTCTTTTTTCTGATCGTTTTTGGAATCGAGTCCATGGTTTCCCTGTTGGGGAATGGATTCATCCTAGCAGTGTGCGGCCACAGCTGTCTCTGCAGCAAGAAGATGCTCCCTTGTGATTTCCTCCTGACCACACTGAGCCTCTCCAGGTTTCTTTTTCAGTTGGTTTCCATGAGCAGCCAATTTTGGTTTTTCAGTTCTCCAGAGACATACATAGATAGTGAAACAGAGCATGCATTTGCATTATTCTGGCATTATTTGAACATAGCCATCCTCTGGTGTGCCACATGGCTCAACGTCTTCTACTGTGTGAAGGTCACCAACTTTCCTCACCCGCTCTTTGCATGGCTGAAGCTGAGAATTGGGGCTCTGGTGCCCAGGTTCCTTGGAATATCTCTCCTCGCTTTCATAATCTGCTCCATTCCTCCAGTCCTGAGGTCTTTTGAAAATGAAAAATGCTGCAATCTCACAGGAAATCTACCAGAGAACACCAGCCAAAGTGAGGCTCATCCCCGCTATTCTCGTATGTTTCTTAAAACTATTCAGATTTATTTTACTGCCATGAATTTTAGCATCTGCTTTACAGCATCTGTTGTTTTGCTTCTCTCTCTGTGGAGGCACACGAGGAATCTGAAGAAGGGCGGCCTCAGCACCAAGGACCTCAGCACTCAGGCCCACCTCAGAGTCATGAACCCTTTGCTTCTCTCCCTCTTCTTCTACATTTTACATTTTGCCGCCATGATCCTTGGCCTCGCTAACGTTTTCGAGTTCGGCAAGCTTGAGCGGCTGATTTCTGATATATTCCTGTCTTCATATCCCACAGCACACTCCGTCATCTTGATATTCATCAATCCCAAGCTGAGGAAAGCGTGTACCCATGTTCTAAACCTCCGAAGAAGTGCCTCCTAA

>Japanese_Gecko_Tas2r42

ATGGGAAGCAATTCTGACTGCGGCAGCACAACAGGTGACTTGTTTGACATCCTGTTTGGAACCATTTTACTGTTTGGAAGAACATGCGTGGATGTCTTCATCCTGACTGTCAATGGCAGGGACTGGATGGAAGAGAAATGCCTGAGCACAACTGACAGGATCCTGACCTTACAAGGCAGCATCAGGATATGTTTGGGGTACATGAATCTAATATGGCACATCCTGGAGAAATTCTGCCCTTGGATCACTCGGATAGGCTATGTTTTCAAAGCGTTCATGTTCATCTTCTGGTACTTGATGTCTTGCAACATCTGGCTCACAACTTCCTTGTGCACCTACTACTGCGTGAAGATCGCAGACTTCAGCCACCCCTTCTTCGTCCACTTGAAACTAAGACTCTCGGGACTGGGGTCGACCTGGCTCCTTGGCTCTGCCATTTTGTCTTTGGCAAGTACCCTACCCTTTGCATACATTGACTTGGAAATACGAGATAATAGCAACTCTTCTAATTTCTCCCAAAATAAAAATGAGACTGACAGCCACACAGGGATGGCGTTCCGTATGTATGTTTTGCTGGGACTTGGAACTTTTGCAGCTTTTAGTATCTGTGCCATTTCTGCTGTCCTCTTACTTTTCTCTCTTTGGAGGCACCGTCAGAGGATGCTGGGTAGTTGGGGGGGCCACAGGAGCCCCCAGACCGACGCCCACTTCCAAGCAGTGATCACAATATTCTCCCTTCTCGTTAACAATGTAGCTGTTTTCATAGCTATTCTAGTGTTCTTGTCAAGAGTCTTTGAAGGAAATTCGATTTCTCAACTTGGTCCTTCAGTTGTTCTATATTGCTGTTTTTTAGTGGAGTCCTTGGTATCAGTATGGGGCAATAAGAAATTAAAAAACGAGTTACGAAGATCTTTGCATTTTGCTAAGTGCAGAGGATGTGTTAGCTAG

>Japanese_Gecko_Tas2r43

ATGGCCACCTTGTTGTCGATAACTGGCTTTGCCCTTCTCCTCATGGAAACCCTTGTTGGGATGGTAGCAAATGGATTTATTGTCCTCATCAGCTGCACTGAGTGGATCAGAAGTAGGAAACTATCCCCGACTGATCTGATCCTGACCTGTCTTGGCTTGGCCAGATTTGCGTGGCAGGCCATAGTGATTCTGGACGCAACTATGTATTCTTTTTTTCTGCGCATTTATCTATTGAATTATGTCCGTTTAGTGCTTACCATAGTGTGGTTATTTACATACAACGTCAGCCTTTGGTTTGCCGCCTACCTCAGCATTTTGTACTTTGTGAAGATCACCACTTTCTCCCACCCTCTTTTCCTCCGAGTGAAGCAAAGATTCGCCGGAATGCTCCCATGGCTGCTTCTCAGCTCAGTGGCCTTCTCTGCTTTCATGACTATGACTATTGCTGTTACCACAGTATTTAGCAGTGACTTAACCATTTGTGATCCATACCAATTATTTTTAAACAATAGCTACAATTCAGGTATGCAAAGGCCGTACTTGTGTAGGAAGCTTGTCATTGCAGTTACTGCTCCAACTTTCATTCTTTCCGTAATATTTTTGTCATCCACAATCTTGTTAATAATATCTCTTTGGAAGCACACAAGACATTTGCAACACAATGGAATCAGCACCAACGATCTCAACACCGGTGTCCACTTAAGTGCCATCAAAGTTCTGGCTTCCTTTGTTGTTTTGTACCTCTTCAGTTTAGTAGCAATCAATTTACAGGTACTGTTGGTTTGGAGACGCATTAATCGCCCCTGGACAGATGTGCTTTTTCAGACTATGAGTGCTGTGTATCCTTCTGGGCATGCGGTGGCCCTGATATTAATGAATCCCAAACTGAAACAGGCATGGGTCAGGATGATACATCACTTAAAATGTCATGTGAGTGAAGCACCATCTTAA

>Japanese_Gecko_Tas2r44

ATGGCGTCTCTCTTCTTCATATTAGCGCTTACACTTCTGGTTATGGAAACCCTCGTTGGAGTGGTAGCAAATGGATTTATTCTCCTCATCAATGGAATTGACTGGTTCAGAAGTAGGAAATTATCCCCAACTGACTTGACCCTGTGTTGTCTTGGCTTGTCCAGACTTGCATGGCAGGTAGTAGGGTTTCTGGATGCAATTATGTTTTTCTTTTTTCTGAGCACCTATCTATCCAATTCCATACAGTTAATGTTTCTCGTCTTGTCGATATTTACACACGCAGCCGATATTTGGTTTGCCACCTGGCTGGGTGTTTTGTATTTTGTAAAGATCACCATGTTCTCCCACCCAGTTTTCCTTCGAGTGAAGCAGAGATTCTCCGGGCTGGTCCCATGGCTGCTTCTGGGCTCAGTCGTCTTCTCCGCTGTTCTTACTATGAGTATTATCACAGCCTTGAACTATGACGCTTCCATTTGCAATCCCTACAAATCACGTCTAAGCAACAGCAACGATTCAGGAATTAAAATGCCTCACTTCTGTAGGAACGTAACCATTCTAGCTACTGCTCCCCATTTCGTTCCAATCGTGATATTTTTGTCATCCACCACCTTATTAATAGCCTCTCTGTGGAAACACACAAGGCGCGTGCAACACAATGGGACTGGCACCAAAGATCTCAGCACCCAAGCTCATTTGACTGCCATCAAAGCTCTGGCTTCCTTCCTTATTCTGTACCTGTCCAGTTTTGTGGCAGTCACTTTGCAATCACTAGTCACCTGGCGGAACGACAATGGCAGTTGGATTTCTGTACTTTTTCATAATGTGATTGCAGCGTATCCCTCTGGACATGCCGTTATCCTGATATTAATTAATCCAAGACTGAAACAGGCATGGGTTGGGATGCTACATCACTTAAAATGTCGTTTGAGAGAAGTACCATCTTAG

>Japanese_Gecko_Tas2r45

ATGGAGGTTTCGGTATTCATGGTAATCAGTTCTATCCTTCTGGTTATTGAAACCCTTGTTGGAATGGTAGCAAATGGATTTATTGTCCTCATGAATTACATTGACTGGTTCAGAAGAAGGAAACTATCCCCAAATGACCAGATCTTGACCTGTCTTGGCTTGTCCAGATTTTCATGGCTAGCAGTCGTATTTCTGGATATAACTAAGGGTTTCTTTTCTAAGGATAAGCATAATTGTCATTATGTATATCTAATGCTTCACATCGGGTTGATTTTTGCAAAAACTGCCACCATTTGGTTTGCCACCTGGCTCAGTGTTTTCTACTTGGCAAAGATCGCCACCTTCTCCCACGCTGTTTTCCTCCAAGTGAAGCTACGATTTTCGGGACTGGTGCCATGGTTGCTTCTGGGCTCAGTTGTCTTCTCTGCTATTATGGCTATTTCTTTAGTCACAAGCTTGAGCAATGGCTTCTCTATGTGTGATTCCAACAAATCACTTTTGAATGTCAGTGATTCAGGAATTAAATTGCCTGACTTATATATGTACATAGATTTTCTAGCTACTGCTCCAAATCTCATTCCTTTAATGATATTTTTGGCATCGTCCATCTTGTTAATGACCTCTCTGTGGATGCACAAAAGACGTATGCAAAGCTATGGATCTGGCATCCAGGATCTCAACACTCAAGTTCACTTGACTGCCATCAAAGCTCTTGTTCCCTTCACAGTTCTGTACCTATCTTGTTTTTTAGCAGTCATTGCTCAGGGAGTACTGATCTGGAATGATATGAGCGACACTTGGCTTTTTATGTTGCTTAGTAATGTAGCTGTTTTTTCTCCTTCTGGGCATGCTGTTATCCTGATATTAATTAATCCCAAACTAAAACAGGCATGGGTCAGGATGCTGCTTCACTTAAAATGCTGCTCGAGTGAAGTGCCTTCTTAA

>Japanese_Gecko_Tas2r46

ATGTATAACTTTTTAAGGAGAATCACTTTTACACTTTTGGTTATTGAAACCCTCGTCGGAATGGCAGCAAATGGATTTATTGTCCTCATCAATTGCATTGACTGGTTCAGAACCAGGAAACTATCCCCGAATGACCTGATCCTGTCCTGTCTTGGCTTGTCCAGACTTGCATGGCTGGTAGTCATGACTCTGGATAGAACTAAGGTATTCTTTTCTCTGGGTAATCATATTTGGGATACACATGTAATGCCTCCCATCGTGTGGATATTTACCAACTCCGCCAACATCTGGTTTGCCACCTGGCTCAGTGTTTTCTACTTGGCAAAGATTGCCATCTTCTCTCACCCCATTTTCCTCCAAGTGAAGCAAAGAATCTCTGCACTGGTGCCATGGCTGCTTCTCGGCTCAGTCATCTTCTCTGCTATGACGGCTGTTATTCTAATGACAAGCCTGAACAATGGCTTTGCCATGTGCAATCCCTCCAATGATTCAGAAATTAAAGAGCCTGACTCCTGGAAGTACTTGGATATTCTAGGTATGGCCCCAAATCTCATTCCCTTTCTGATATTTCTTTCATCGACCATCTTATTAATAAGCTCTCTGTGGAAGCACATGAGAAGAGTCCAAAGCAATGGGACTGGCACCGGCGATCTCAACACACAAGCGCACTTGAGTGCCATCAAAGCTCTGGCTTCCTTCGCTGTTCTGTACCTGTCTAGTTTTCTAGCAATCACTTCACAGGCAGTACTGATCTTAAATAACATGGATCACAGTTTGCCAGTGAGGCTGCTTGATCTTGTGGTTGCTGCTTATCCCTCTGCTCATACTATCATCCTGATACTAATTAATCCCAAACTGAAGCAGGCATGGGTCAGGATGCTACATCCCTTAAAATGCTGTTTGAGAGAAGTGCCAACTTAA

>Japanese_Gecko_Tas2r47

ATGGCTACCATATTCATGAGGATCACTTTTACCCTTCGGGTTACTGAAATCCTTGTTGGAATGGTAGCAAATGGATTTATTGTCCTCATCAATTGCATTGACTGGTTCAGAAGCAGGAAACTATCCTCAAATGACCTGATCCTGACCTGTCTTGGCTTGTCCAGACTTGCATGGCTGGTAATCATGATTCTGGAGGGAATGGAGAATTTCTTTTCTCTGGGCAATCGTGTTTGGAATGACGCACATCCAATGCCTCCAATCATGTGGATATTTACAAACTCTGCCAACATCTGGTTTGCCACCTGGCTCAGTGTTTTCTACTTGGCAAAGATTGCCATTTTCTCACATCCCATTTTCCTCCAAGTGAAGCAAAGAATCTCTGCACTGGTGCCATGGCTGCTTCTGGGCTCAGTCGTCTTCTCTGCTATGACGGCTGTCATTCTAATGACAAGCTTGAACAATGGTTTTTCCATACGCAGTCCCGCCAATGATTCAGAAAGTAAAAAGCCTGGCTGCTCGAACTACGTGGGTATTCTAACAGTAGCAACAAATCTCATTCCCTTTCTGATATTTCTGTCATCGACCATCTTATTAATAACCTCTCTGTGGAAGCACATAAGGCACGTACAATGCAATGGAATTGGTGTCAGGGATCTCAACATTCAAGTTCACTTGACTGCCATCAAAGCTCTGGCTTCCTTCGCTGTTCTATACCTGTCTAGTTTTCTAGCAATCATTTCGCAAGAAGTACTGATCAAGAATAACATGGATCACAGTTGGTCAGTGAGGCTGCTTGATCTTGTGGTTGCCGCTTATCCCGCTGGTCATACTATTATCCTGATATTAATTAATCCCAAACTGAAACAGACATGGGTCAGGATGCTACATCACGTAAAATGCTGTGAAGGGCCATCTTAA

>Japanese_Gecko_Tas2r48

ATGGCCACCTTGTTGTTGATCACTGGCTTTGCCCTTCTCATCATGGAAACCCTCATCGGAATGGTAGCAAACGGATTTATTGTCCTCTTCAGCTGCATTGAGTGGACCAGAAGCAGGAAACTATCCTCGACTGACCTGATCCTGACCTGTCTTGCCTTGGCCCGACTTGCATGGCAGGCCATAGTGATTCTGGATGTAACTATGTATTCTTTTTTTCTGCGCACGTATCTATTGAATCATGTATTTTTAATGATTAGCATGGTGTGGTTTTTTACATACAACACCAACCTTTCATTTGCTGCCTGCCTCAGCATTCTGTACTTTGTGAAGATCACCACTTTCTCCCACCCTCTTTTCCTCCAAGTGAAGCAAAGATTCTCTGGGCTGCTCCCATGGCTGCTTCTCGGCTCACTGGCCTTCTCTGCTTTCATGACTATGACTATTACTGTTATCACAGTATCGAGCAGGGGCTTAACCTTTTGTGATCCATACAAATTACTTTTCAACAATACCTTTGATTTAGAAATTAAAAATGATCGTCCTTGTACGGACTTTGCCATTTCGGTTGTTGTTCCAAATGTCATCCCTTCTGTGATATTTTTGTCATCCACAATCTTGTTAATAATATCTCTGTGGAAGCACACAAGACATATGCAACACAATGGAATCAGCACCACAGATCTCAACACCAGTGTCCACTTAACTGCCATCAAAACTCTGACTTCCTTTGCTATTTTGTACCTCTCCAGTTCTGTAGCCATCAATTTACAGATATTGCCCGCGTGGAGAATCATTGATCGCTCTTCGACAAATGTGCTTTTAGAAGCTATGAGTGCTGTGTATCCCTCTGGGCATGCAGTTGTCCTGATATTAATTAATCCCAAACTGAAACAGGCATGGGTCGGGATGATGCACCACCTAAAATGTCATGAGAGTGAAGCACCATCTTAA

>Japanese_Gecko_Tas2r49

ATGGCCACCTTGTTGTCGATCACTGGCTTTGCCCTTCTCATCATGGAAATCCTCATCGGGATGGTAGCAAATGGATTTATTGTCCTCATCAGCTGCACTGAGTGGATCAGAAGCAGGAAACTATCCCTGACTGACCTGATCCTGACCTGTCTTGGCTTGTCCAGACTCATGTGGCAGGCAACAGTGACGCTGCAAGTAACTATGCTTTCCTTTTTTCTGCGCACTTACCTATTGACTCATGTCTATTTGGTAATTAACATGGTGTGGTTCTTTACTTACATTATCAACCTTTCGTTTGCTGCCTGGCTCAGCATTATGTACTTTGTAAAGATCACCACTTTCTCCCACCCTATTTTCCTCCAAGTGAAGCAAAGATTCTCCGGGCTTCTGCCAAGGCTGCTTCTCAGCTCAGTGGCCGTCTCTGCTTTCATGACTGTGACTGTTGCTGTTATCACAGTATCAAGCGGTGGCTTAACCATTTGTGATCCCTACAAATTATTTTTAAACAATAGCTACAATTCAGATATTAAAAGGCCTCACTTGTGTAGGCAGCTTTTCATCACAGTTATTGCTCCAAATTTCATTCTTTCTATAATATTTTTGTCCTCCACAATCTTGTTAATAATATCTCTGTGGAAGCACACAAGACATTTGCAACACAATGGAATCATCACCAAGGATCTCAATGCCAGTGTTCACTTAACTGCCATGAAATCTCTGGCTTCCAGTGCTATTTTGTACCTCTTCAGTTTTGTCGCAATCAATTTACAATCAATATTGGTTTTGAGAAGCATTAATCACCCTTGGACAGATGTCCTTTTTCAAACTATGAGTGCTGTATATCCCTCTGGGCATGCTGTTGTCCTGATATTAATCAATCCCAAACTGAAAAAAGCATGGGTCGGGATGATGCACCACTTAAAATGTCATGAGAGTGAAGCACCATCTTAA

>Japanese_Gecko_Tas2r5

ATGGAGGTTTTGGCATTCATGATAATCGGTTCTATCCTGGTTATTGAAGCCCTTGTTGGACTGGTAGCAAATGGATTTATTGTCCTCATGAATTACATTGTCTGGTTCAGAAGCAGGAAACTGTCTTCAAATGACCAGATCATGACCTGTCTTGCCTTGTCCAGATTTTCATGGCTGGTAGTCGTAATTCTGAATATAATGAAGCATTTCTTTTCTATGGGCCAGCACACTTGTATTTATGCCTATCTAATGCTTCCCATCTTGTGGATATTTACGAACACTGCCTCCATTTGGTTTGCCACCTGGCTCAGTGTTTTCTACTTGGTGAAGATCGCCACCTTCTCCCACCCTGTTTTCCTCCAAGTGAAGCTAAGATTTTCAGGACTGGTGCCACGGTTGCTCCTGGGCTCGGTTGTCTTCTCTGCTATTATGGCTATCACAAGCTTGAGCAGCAGCTTCTCTATGTGTGATTCCGACAAATCACTTTTGAACATCACTGATTCAGGAATTAAATTGTCTGACTTAAACACAGATTTTCTAGCTACTGCTCCGACTCTCATTCCTTTAATGATATTTTCGTCATCGACCATCTTGTTACTGGCCTCTCTGTGGATGCACAGAAGACGGATGCAACGCAATGGAACTGGCATCCAGGATCTCAACACTCAAGTTCACTTGACTGCCATCAAAGCTCTGGCTTCCTTCGCTCTTCTGGGCCTGTCTAGTTGTTTAGTAGTCACTGCTCAGGCAGTACTGATCTGGAATAACATGAGCGACACTTGGCTTTTTATGTTGCTTAGCAACGTAACTGTCTCTTCTCCCTCTGTGCATACTGTTATCCTGATATTACTTAATCCCAAACTAAAACAGGCATGGGTCAGGATGCTGCTTCACTTAAAACACTGCTCTAGTGAAGTGCCTTCTTAA

>Japanese_Gecko_Tas2r50

ATGATGGTAGCGATGGACAGATATCTACTTTCCCCACTGGGAGTCTTTTTTCTGATCATTTTTGCAATCGAGTCCATTGTTTCCCTGTTGGGGAATGGATTCATCCTAGCCGTGAGCGTCCACAGCTGGCTGCACAGCAAGAAGATGCTCCCTTGTGATTTCCTCTTGACCACTCTGAGCCTCTCCAGGTTTCTTTTGCAGTGGGTTTCTTTGAGCAGCCGCAGTATGTATTTCAGATCTCTTCTAGAGACATATATATACAGTAAAGAACAGCAGGCATTTGGGTTCCTCTGGGTTTATTTGAACACAGCCAGCCTCTGGTGTGCCACATGGCTCAATGTCTTCTACTGTGTGAAGGTCACCAACTTCGCTCACCCTCTCTTTTCATGGCTAAAGCTAAGAATTGGGGTGCTGGTGCCCAGATTTCTTGGAATATCTCTCCTATCTTTCATAATCTGCTCTATTCATCCAGTCGTGGGGTATTTTGAAGATGAAAAATGCCACAATCTCACAGGATACCTTCCAGAGAACATCAGCCAAAGGGAGGCTGATGACACCAACTGTGCTATGCTTTTAAATACTCTTCAGATGTCTTTTACTGCCATCAGTTTCAGCATCTGCTTAACAGCATCCCTTGTTTTGCTTCTCTCTCTGTGGAGGCACACGAGGAATCTGAAGAAGGGTGGCCTCAGCACCAAGGACCTTAGCACTCAGGCCCACCTCAGAGTCATGAAGCCTTTGCTTCTCTCTCTCTTTTTTTACATTTTACATTTTGCTGCCATGATCCTTGCCCTCACTAATATTTTCAGGTACGGCAAGCTTGAGCAGCTGATTTGTGAGATATTTATGGCTTTATATCCCTCAGCACACTCCGTCATCTTGATAGTCACCAATCCCAAGCTGAGGAAAGTGTGTTCCCATGTTCTGAACCTCAGAAGAAGTGCCTCATGA

>Japanese_Gecko_Tas2r6

ATGTTGTGCTGTGTGCTGTTGATGGGAAGTGTGAGTATCGCAGCAGAAGAACGTCTACTCTTTTCTTTTGTGCTCAGAAAGATGGCCACCCTGTTGTCAATAACTGGCTTTGTGCTTCTCATCATGGAAACCCTCGTTGGACTGGTAGCAAATGGATTTATTGTCTTCATCAATTGTATTGACTGGTACAGAAGCAGGAAACTATCCCCGACTGACTTGATCCTGATCTGTCTTGCCTCATCCAGACTCATGTGGCAGGCACTAGTAATGCTGCATGTAACTATGTTGTCCTTTTTTCTCCACACCTATGTATTGAAACGGGTACATTTAATAGTTATGATCATGTGGTTCTTTACAGACACTGTCAATCTATGGTTTGCTGCCTGTCTCGGTGTTTGGTATTTAACAAAGATCGCCATCTTCTCCCACCCTATTTTCCTCCAAGTGAAGCAAAGATTCGCTGGGCTTCTCCCATGGCTGCTTTTTGGCTCTGTGGTCTTCTCTTCTTTTATGACTATTATTAACTTCACGGAATCCTTCAGTGGCTTAGCCACTTGTGATCCCTACAAATTAGTTTTAAGCAACAGCTCTGATTCAGACATTCAAAAGCCACACTCTTGTATGGGCCTAGTCTTTTTACGTATTGTTTCAAATTTCGTCCCTTCTGTGATATTTTTGTCATCCACAGTCTTGTTGATAATATCTCTCTGGAAGCACACAAGACATTTGCAACACAATGGAACTGGAGTCAAGGATATCAACACCAGGGTTCATTTAACTGCCATCAAAGCTCTGGCTTCCTTTGCTATTTTGTACCTCTTCAGTTTGGTAGCCATCAATTTACAGTCAATGCTGGTTTGGGGAAGCAATGATCTTTCTTGGACATCTGTACTCTTTCACAATGTCAGTGATGTGTATCCCTCGGGGCATGCGGTTATCCTGATATTAATTAATCCCAAACTGAAACAGGCATGGATCAGGATGATACATCACTTAAAATGTCATGTGAGTGAAGCATCATCTTAA

>Japanese_Gecko_Tas2r7

ATGATGGTAGCGATGGACAGAGATCAGCTTTCTCCGCTGGGAGTTTTTTTTCTGATTGTTTTTGGAATTGAGTCCATTGTTTCACTGTTAGGGAATGGATTCATCCTAGCCGTGAATGGCCACAGCTGTCTCTGCAGCAAGAAGATGCTCCCTTGTGATTTCCTCTTGACCACTCTGAGCCTCTCCAGGTTTCTTTTGCAGTGGGTTTCCATGAGCAGCCGATATATGTATTTCAGCTCTCCAGAGACATTCATATATAGTAAAAAACATCAGGCACTCAGGTTCCCCTGGGTTTATTTGAACACAGCCAGCCTCTGGTGTGCCACATGGCTCAATGTCTTCTACTGTGTGAAGGTCACCAACTTCGCTCACCCTCTCTTTATATGGCTAAAGCTAAGAATTGGGGTGCTGGTGCCCAGATTCCTTGGAATAACTCTCCTAGCTTTCATAATCTGCTCTATTCATCCAGTCGTGGGGTATTTTGAAGATGAAAAATGCCACAATCTCACAGGATACCTTCCAGAGAACACCAGCCAAAGGGCGGCTCATGACATCAAGTGTTTTATGTTATTAAATACTCTTCAGATGTCTTCTACTGGCATCAGTTTCAGCATCTGCGTAACAGCATCCATTGTTTTGCTTCTCTCTCTATGGAGGCACACGAGGAATCTGAAGAAGGGCGGCCTCAGCACCAAGGACTTCAGCACTCAGGCCCACCTCAGAGTCATGAAGCCTTTGCTTCTCTCCCTCTTCATCTACATTTTATATTTTGCCGCCATGATCCTTATCCACACTAATATGTTCAGGTACGGCAAGCTTGAGCAGCTGATCTGTGAGATATTCATGTCTTTGTATCCCTCAGCACACTCCATCATCTTGATATTCACCAATCCCAAATTGAGAAATGTATGTACCCATGTTCTGAAACTCAGAAGAAGTGCCTCATGA

>Japanese_Gecko_Tas2r8

ATGTTTTCTCTGCGAAACATCACACAGGAACCAGGAGAGCCACAATTTAATTCATCTTGTTCCCACTTTAGATATTTCTCTCCGCCGATGGTAGTGATGGACAGAGATCTGTTTTCCCCACTGGGAGTCTTTTTTCTGATCGTTTTTGGAATCGAGTCCATTGTTTCCCTGTTGGGGAATGGATTCATCCTAGCCGTGAATGGCCACAGCTGGCTCTGCAGCAAGAAGGTGCTCCTTTGTGATTTCCTCTTGATCACTCTGAGCCTCTCCAGGTTTCTTTGGCAGTCAGTTATCACAAGCAGCCAATTTCTGTATTTCAGATCTCCAGAGACATATATATATAGTAAAGAAGAGCAGGCATTCGGGTACCTCTGGGTTTATTTGAACACAGCCAGCCTCTGGTGTGCCACGTGGCTCAATGTCTTCTACTGTGTGAAGGTCACCAACTTTACTCACCCTCTCTTTTCATGGCTGAAGCCAAGAATTGGGGTGCTGGTTCCCAGATTCCTCGGAATATCTCTGCTAACTTTCATCATCTCCTCTATTCTGCCAGTCCTGAGGTCTTTTGAAGATGAAAAATGCCACAATCTCACAGGAAACCTCCCAGAGAACACCAGCCAGAGTGAGGTTCACGGCACCAACTGTGTTATGTTTTTAAATACTCTTCATATTTATTTTACTGCCATCAGTTTCAGCATCTGCTTAACGGCATCCCTTGTTTTGCTTCTCTCACTGTGGAGGCACAAGAGGAATCTGAAGAAGAGCGGCCTCAACACCAAGGACTTCAGCACTCAGGCCCACCTCAGCGTCATGAAGCCTTTGCTTCTCTCCCTCTTCTTCTACATTTTACATTTTGCCACCATGATCCTTGTCTTCAATAATATTTTCAGGTACGGCAAGCTTGAGCAACTGATTGGTGAGATATTCCTGTGTTCGTATCCTTCAGCACACTCTGTCATCTTGATATTCACCAATCCCAAATTGAGGAAAGCAAGTACCCATGTTCTGAACCTCAGAAGAAGTGCCCCATGA

>Japanese_Gecko_Tas2r9

ATGTTTTCTTGGCAAAACATCGTACAGGAACTAGAAGAGCCACAATTTAATTCGACTTGTTCCCATTTTTTGATCTTTCCGATGATGCTCGAGATGGACAGAGATCTGTTTTCCTCACTGGGAGTCTTTTTGCTGGGCATTTTTGGAATCGAGTCCATTGTTTCCCTGTTGGGGAATGGATTCATCCTAGCCGTGAATGGCCATAGCTGGCTCCGCAGCAAGAAGATGCCCCCTTGTGATTTCCTCTTGACTACTCTGAGCCTCTCCAGGTTTCTTTCGCAGTGGGTTTCTTTGAGTAGCCGTTGTATGTATTTCAGCTATCCAGAGACATATTTATATAGTAAAGAAGAGCAGGCATTCAATTTCTCCTGGTTTTATTTGAACACAGCCAGTCTCTGGTGTGCCACATGGCTCAATGTCTTCTACTGTGTGAAGGTCACAACCTTTACTCACCCTCTCTTTTCATGGCTGAAGCTAAGAATTGGGGTGATGGTGCTCAGATTCCTTGGAATATCTCTCCTACTTTTCATAATCTGCTCTATTCATCCAGTCGTGGAGTATTTTGAAGATGAAAAATGCCACAATCTCACAGAAAACCTGCGAGAGAACAGCAGCCAAAGGGAGGCTCGTGGCACCAACTCTTTAAGGTTTTTAAATACTCTTGAGATTTTTTTTACTGCCATCAGTTTCAGCATCTGCTTAACAGCATCTGTTGTTTTGCTTCTCTCTCTGTGGAGGCACATGAGGAATCTGAAGAAGAGCGGCCTCAGCACCAAGGACTTCAGCACTCAGGCCCACCTCAGAGTCATGAAGCCTTTGCTTCTCTCCCTTTTCTTCTACATTTTACATTTTGCCGCCATGATCCTTCTCCTCACGAAAACTTTCAGGTACGGCCAACTTGAGCAGCTGATTTGTGAGATATTCGTCTATTCATATCCTTCAGCACACTCCGTCATTTTGATATTCACCAATCCCAAGCTGAGGAAAGTGTGTTCCCATGTTTTAAACCTCAGAAGAAGTGCCTCATGA

>Speckled_Rattlesnake _Tas2r1

ATGTCGGTGTCAGGCGTTCACAACTGGCTCTGCCTTATTATCATCACAGCTGTGACTCTCGTCGGGATGACTGGAAATGGATTCATCTTTCTTTCGGACTGCCATGATTGGATCCGAAGCAAAGCACCATCGGGTCCCGGCCTCCTGCTGATGACACTCAGCCTGACCCGATTTATTTTCCTTGGAATCATGCTCGGCTTCCATTGTTTTAGCTTCCTTGATATTAATCGACCAAAATATGCCGGAAGTGTCATCAGTTTCTTCTGGACCTTTTTCAATGCCACCACCCTGTGGATTACTACCTGCCTTGGAGTATTCTACTGTGTGAAAATTGTCAACTTCAGCCAGCCTTTCCTGGTGAAAATGAAGCTCAGGATTTCCAGCATGGTTCCCCATTTGCTCGTTGCGGTTGTCTTGGTTTCCTTGATTTCGGCTCTTCCTTTCCTCTGGATTGATGATCACAATCAGTCTGACAACGCAGAGGGTGTACGTGAGCTGAGGGTTCAGACGTTCCTGTTCAGTATACTGTATACTCTAGGGACTTTTCCATCGTTTGTGATATTTTTAATTTCCTCTGGTTTTTTAATTTACTCCCTTGTGCACCACATGAAGAGAATGCAGAATAGCTCAGTTGGCTTTCGAGATCAAAGGATGGACGTTCATCTGAAGACTACTAAGATCTTGACCTCCTTCCTCATCCTCTATGCAGCAACCTTTGCAGCAGAAATCTCAATGATCTTTTCCCCCAGTCCCTGGACAACGGTTATATCCAACATAGTGGTTAGTTCATATAATTCAGGACACACCGTTGCTTTGATTGTCATGAATTCCAAACTAAGGGGACGATTAAGCAAGATGTTCTGGTGTTTCAGAAAACAGACATGA

>Spiny_Softshell_Turtle_Tas2r1

ATGTTTTCTATCATTATTTATTTCATCATTTTAGGAATGGAGCTCATTGTAGGGATGATAGCAAATGGACTGATGGCTGTTGTGAATTGCCTAGAGTGGATCAGAAGCAGAAATGTGACCTGTTGTGACATGATCCTGACTAGCTTGGGTATCTCCAGATTTTTCTTTCAGTGCATGATAATAATTAATAGCGCCATCTATAACATATCTTTAGAGGACAATGCACATCTTGCTTTGATGAGAACCTTGGCTTTTATCTCAAGCTTTCTAAATACTCTCAGTCTCTGGTTGGCCACCTGGTTGAGTGTCTTCTACTGCGCAAAGATCGCCAACTTTAGCCAACCCCTCTTCTTCTGGCTGAAGTGGAGAATATTAGGGCTAATGCCACAGCTACTCACGGGAACCTTTCTGGTCTCCTTTGTAACCAGTCTCCCTTCAATCAATTCCGTAAATAGGAAATACGTAAACAATTCAGTGAATAATCTATTAGGAAACACCACAGAAGAGTGGACATACTACACTAACTTTTTTTCTGGCCTTTCTATTTTGTACATGCTTGGCCATTCCTTTCCCTTTGTTATATTTATTGTTTCCTCTGCCCTGTTACTCACATCTCTGTGGAGACATACCAAAAGGATGGCAAAAATCACAAGCAGCTGCAGGGACACTGTTACGCAGGCTCATGTGAAAGCTATTCAAGGACAGCTCTCTTTCATTTTCTTCCACAGTTCTTATTTTGTGGCACAAGTAATATTATTCTCAGGATTTTTTACCAACAGCATCTCCAACTCTCTGTGGTGCATTGTGATAATGGCTGCTTATCCCTCTGGGCATTCTGTTATCTTAGTTCTGGGTAATCCCAAACTGAAGAAGGTAGCAGTGAGAGCTTTGCACTATGCCAGGTGCAGGCTGAGAGATGAGGTTTCATAA

>Spiny_Softshell_Turtle_Tas2r2

ATGCTTACTCCAGTTGCTCTCATCTTTCTGATCCTTTTGGGACTTGAGTCCCTTGTTTCAAATTTTGGGAATGGATTTATCATCGTTGTGATTTTCTCTAACTGGATCAAAAGCAGAAAGCTGGCCTCCTGTGAGCACATCCTGATCTGTCTGAGCATCTCCAGATTTCTCTTACAGTGGCTTGTAATGCTGAGCAACTTCATCTATATCAGTTTTCCAAAGACTTCTGCACTGGGATGCAAACACAAAGCATTTGGAATCCTGTGGGCTTATCTAAACTTAGTCAGTCTCTGGTGTGCCACCTGTCTTAGCTTCTTCTACTCTGTAAAGATTGCCAACTTCACCCAACCCCTCTTTCGCTGGCTGAAGCTGAGAATTGCTTGGCTGGTGCCAAGACTGCTTCTGGGGTCCTTGATCATTTCCTTGGTCAGCACCATCCCATTAGTCTGGAGTGATGTTGGTTTTGATTTATGCAACTCAACAAAAAGTCTAGAACGAAACACAACCTGGAATGATGCTAAGGATATCCCATATATCATCTTTGTTCCTGTGCAAATCCTTGTATTGATCATCCCTTTCATTATATTCTTCGTTTCATCTACCCTATTGCTCATCTCTCTATGGAAACACACCAAGAAAATGAAAAATAATGTTACTCGTTTCAAAGATCTCAGTGTGGAGGCTCATATTGGTGCCATGAAATCTCTGCTCTCTTTTTTTATTCTTTACATTATATATTTTGTTACTGTAATAGTCATTTTGACATCTTCAATCACATCTCAAAACTCTGTACATTTACCTTATGAAGTTCTATTGTCTGCATATCCTTCTGGACACCCCATTGTTTTAATTCTGACCAATCCAAAATTGAAACAGGTGGCAGTGAAGATTCTGCATCAAATCAAATGCCAATTGAGAGAAGGGACTTTATAA

>Spiny_Softshell_Turtle_Tas2r3

ATGATGAAAAATTCTTTAGCACTTTCTGATATTTTTTATCTGATCATAACAGCAGTTGAATTATCAGCAGGAGTTGTTGCAAATGGATTTATTGTTGGCTTAAATTGCATTGATTGGGCCAAAAGCAGAACCATGACTTCCTATGATATGATTATAACCAGTCTGGCATTCTCCAGATTTTGCCTACAATTCTTAGTGTCATCAGACAATTTCTTACATATTTTATATCCAGATGTCTGTGATATGGTTGAAATAATAATGATGATGTTAGTTATTTGGATGTTTATAAACCATGTGAGTCTCTGTTTTGCAAGCTGCCTTTCTGTGTTCTACTGTGTGAAGATTGCCACTTTCAACCAGTCTTTCTTCACCTGGTTAAAACTGAAACTCTCCAGATTGGTGCCATGGCTGCTTTTGGGCTCCCTCCTGTACTGCTTGGTTACAACAGTCACTTTTACATTTTTCAGCTATTTCTTTATGATAACCTCTCACATCTGTCCATATCGTCCATCAAGAAATATCACAATACCAGAAAAAGAAAAAAACCTTACAACGTTTGTTTTTCTGATACATGGAGTAGGATCAATTTTCCCTCTTATTCTATTTATTGCTTCATCTCTTTTGTTAATCATATCCCTTTGCAGACATATCAGGAAAATGGACCTTAATTCAGACCTTAATCCAAGTTTCAGGAACTCGAGGACAGATGCCCACGTGAGTGCACTTAAATCTGTGTTGTCCTTTTTCATCATCTACAATATTTTTTATGTGGCTTCAACACTCTCAATTGGAACTGGGTCCTATTTCAGTGCTCAATGGAAAATTATGCTTTGTACACTTGTAGTTGCTGCCTACCCTTCTGTGCACTCCTTTGTCTTGATTCTGGTCAACCCCAAACTAAAACTGGCATCAGCAAGGATTCTGCATTCTGCCAACTGCTGTTTCAGAGAAGTCACTTCATAA

>Spiny_Softshell_Turtle_Tas2r4

ATGGAAGACATTGATTATAACATTGAACAAGAGGATGACATCACCATACCACGCATAATTATGCTGGTAATCTTAGTAGCTGAGGCTTTTGTCGGGATGTGGATAAATTCCTTCATTGTGGCCACCAATTGCTTCCAGTGTGTCAAGCACAGGGGCCTGTCCTCAAGTGACAATATCTTGACTGTTGTGGCATTCTCAAGATTTTGCATCTTGCTCGAAACAACCTTACAGACTTTTTGCTCAACATTTTACCCAGAGATCTATTACATGGACTCCGTGTTCCAAGCCTTCCGAGCTGTGACCTGGTTTCTGAACTCTTCCAATCAATGGTTTGCTGCTTGCTTGGGTGTATTTTACTGTGTGAAAATTGCAAACTTCAGCCACCCCCTTTTCATCTCTCTAAAATTTAAAATCTCCAGATTGGTGCCATGGTTGCTCCTGGTTTCTGTGCTTTTTTCTTTGTTCAGCAGCCTCCCTTTCCTTAATACTCTTTACAAAATACGGTATAATGATTTCAACTCCAGCCTCAAAAGGACTTACCCTATGAAAAACGTCACAGTGGAAACATCTGTTTCTCACGTACTTTTTATCTGTGGCACTGGATTTTCCACAGCATTCACCATATTCATCATTTCAGCCTTTCTCTTATGGTTCTCTCTCTGGAGACACACCCGGCGGATGCAAAACAACTCCAGATGTTTTAGGAGCCCTTGCGTAGAGGCCCACATTCAAGCAATGAAAGCCATCATGTCGTTCTTACTCATCAATGTTGTTAACTTCATAGCTTTGCTGATCTTGTTGACAAATACACTTGAAGAAACCTCTGTTATGGGCATTGCCTGTACAATCATCATAGATGCTTGTCCATCAGTACATTCCATTGTCTTGGTTCTGAGCAATTCCAAATTGAAGACCACATTGATTAAAGTTCTGCATTATGCAAAGTGCAAGGGGTGA

>Spiny_Softshell_Turtle_Tas2r5

ATGCTTTCTGCAATTATTATTGCTTTGATCGTTTTAGGAATTGAGCTCATTATAGGGAGTATAGCAAACGGACCGATGATTGTTGTGAATTGCCTAGAGTGGATCAGAAGCAGAAAACTGACCCGTTGTGACATGATCTTGACTAGTCTGGGCATCTCCAGATTTCTTCTACAGTGCATGATATTCGTTAACAGTATCGTCCTTCAATTACTTCAAGATATAAATAGATCTTGTAATACTTCAAGTCATTTCTTTGTTGTTTGGATGTACCTAAGTACTCTCAGTCTCTGGTTTGCCACCTGGCTCAGTGTCTTCCACTGTGTGAAGATCGCCACATTCAGCCAGCCCCTCTTCCTCTGGCTGAGGCAGAAAATACCAGGGCTACTGCCACAGTTACTCTTGAGTTCCTTATTGATTTCCTTGCTCACCTGTTTCCCTTCAGTCAATACTGTATATAGAAATAGTTCAATGAATAATCTGTCAGGAAACACCACAGTGGAATGTAAATGTGTGATTGATTTATTTTCTGGCCTGTCTATGTTCTCTATCGTTGGATTTTATTCTCCCTTCATTATATTTATTGTTTCCTCTGCACTGTTAATCACTTCTCTGTGGAAACACAGCAAGAGGATGAGAAAAGCCATGAGCAGCTCCAAGGACACCATTACTGAGGCTCATGTCAGAGCAATTAAAGGACTGATCTCTTTCATTTTTTTTCTACAGTTCATATTTTGCAGCACTAGTCACATTTTTGATAGAATTATTCAACAACGACCTCTGTTTCTTGTTGTTGTGGGGAGTGATAATGGCTGCTTATCCCTCTGGGCACTCTGTTATCCTTGTGCTGGAGAATCCCAAACTGAAAAAGGTAGCAGTGAAGGCTTTGCATTATGCCCGGTGCAGGCTGAGAGATGA

>Timber_Rattlesnake_Tas2r1

ATGTCGGTGTCAGGCGTTCACAACTGGCTCTGCCTTATTATCATCACAGCTGTGACTCTCGTCGGGATGACTGGAAACGGATTCATCTTTCTTTCGGACTGCCATGAATGGATCCGAAGCAAAGCACCATCGGGTCCTGGCCTCCTGCTGATGACGCTCAGCCTGACCCGATTTATTTTCCTTGGAATCATGCTCGGCTTCCATTGTTTTAGCTTCCTTGATATTAATCGACCAAAATATGCCGGAAGCGTCATCAGTTTCCTCTGGACCTTTTTCAATGCCACCACCCTGTGGATTACTACCTGCCTTGGAGTATTCTACTGTGTGAAAATTGTCAACTTCAGCCAGCCCTTCCTGGTGAAAATGAAGCTCAGGATTTCCAGCATGGTTCCCCATTTGCTCGTTGCGGTAGTCTTGGTTTCCCTGATTTCGGCTCTTCCTTTCCTCTGGATTGATGATCACAGTCAGTCTGACAACGCAGAGGGTGTACGTGAGTTGAGGGTTCAGACGTTCCTGTTCAGTATACTGTATATTCTAGGGACTTTTCCATCGTTTGTGATATTTTTAATTTCCTCTGGTTTTTTAATTTACTCCCTTGTGCACCACATGAAGAGAATGCAGAATAGCTCAGTTGGCTTTCGAGATCAAAGGATGGACGTTCATCTGAAGACTACTAAGATCTTGACCTCCTTCCTCATCCTCTATGCAGCAACCTTTGCAGCAGAAATCTCAATGATCTTTTCCCCCAGTCCCTGGACAACGGTTATATCCAACATAGTGGTTAGTTCATATAATTCAGGACACACCGTTGCTTTGATTGTCATGAATTCCAAACTAAGGGGACGATTAAGCAAGATGTTCTGGTGTTTCAGAAAACAGACATGA
