# Supplementary material for: Genomic evidence of bitter taste in snakes and phylogenetic analysis of bitter taste receptor genes in reptiles
Source: PeerJ. 2017 Aug 18;5:e3708. doi: 10.7717/peerj.3708 (PMC5564386; doi:10.7717/peerj.3708)
Supplement: Data S2 [file peerj-05-3708-s002.docx]

>Adder_Tas2r1

MSVSGVHNWLCLIITTAVTLVGMTGNGFIFLSDCQDWIRSKTSSGPGLLLMMLSLTRFIFLGLTLSLHCLSFLDINRPKFAGSVTIFFWAFFNATTLWITTCLGIFYCVKIVNFSQPFLVKAKLRISSMIPHLLVAVVLVSLISALPFLWIDDHSQSDNTEDVPEMRARMLLLSILYILGTFPSFVIFLISSGFLIYSLVQHVKRMQNSSVGFRDQRMDVHLKTTKILTSFFILYTATFVTEISMTFSPSPWTLVISNMVVSSYNSGHTVALIVMNSKLRGRLSKMFWCFRKQT

>American_alligator_Tas2r1

MTPLNVVTLIGLAIEFVVGIIANGLIVGFNCIAWIKSQKLDSCALVLISLGTSRFFLLYAILINNIFFAIPKKRIEQCNMWRAINFTWMYLSTLSLWFATWLAVFYCVKITSFNQPLFLWLKLRFSGLLPWLILGSLLVSLATSLPSVNVIHINYLNNSINNLSRNITVVCLYKTNTSLSSLILTVLGQYSPFVLFFVPSLLLVTSLLRHTKRMGENMSTSRDISAEAHIRAIKALLSFIFLYIFYMLAQFFTLTNKFATSSPYLIWLCIMILGGYPSGHSVILILSNPKLKEAALKGLHNARCLQEDESQ

>American_alligator_Tas2r2

MEDNRGNISEGNIWEPDMSVLITVLFETFLGISLNAFIIAVNCIDWVKKRRLSTSDQLLTILSFSRICLLLSEDAEFVSSTFNPSFYNSKSGFLMFAGLAWFLSTSSLFFAACLSVYYCVKIANFSCRFFITLKRKISQLMPWLLLVSVMISLLSNLPIFIAIYNVSDNSCNSSCSQNHTGDNVTQKKLFMEMIIVFSFGSSIGFTILCISAVLLLFSLWRHIRHMKGSSAGVGKPSMEAHVKAVKMVMWVLFINLIHFLVWLSFITLAFSPNIFVQHFLTQVTIFCPSIHALVLILSIPKLKQALARILHYVKCKGCAKEGIP

>American_alligator_Tas2r3

MEDNRGNISEGNIWEPDMSVLITVLFETFLGISLNAFIIAVNCIDWVKKRCLSTSDQLLTILSFSRISLLLSEDADFVSSTFNPSFYYSKSGYFKFTGVAWFLSTSSLFFAACLSVYYCVKIANFSYRFFITLKRKISQLMPWLLLVSVMISLLNSLPIFIAIYNISDNSCNSSCSQNHTGDNVTGDTILLNTIIVFCFGLSIGFTILCISAVLLIFSLWRHIRHMQGSSAGVGKPSMEAHVKAVKMVMWVLFINLIHFLAWLSFITFAISPNIFVQRFLIQVTIFCPSIHALVLILSIPKLKQALARILHYVKCKGCAKEGLP

>American_alligator_Tas2r4

MVDKSCAFLSAIELSPIILFYLSIVAIESVTGIVGNGFILVINLASWVRNRVVSSCDMILIFLSFSRLCLQSCMLMDFVCNLFYPSFYNQEDVYENFKAIFVFLNNSSLWFATWLGVFYCAKIANFNHSSFLWLKQNISSLVPWLLAGSLLFSFGSSLSFYWDIYKVYCNYSTAFPLENTTELKVIKNTNLFYVIFLCNASLSLPTIVFVSSIVLLISSLWRHTKRMQNNGTGLRDPSTEAHRGAIKSVFSFLILYFFNLIALILTLSNIFSAYGTWDILCMLVMSAYPMVHSVILILGNPKLRRVSLNFLHYASCHFRGGPK

>American_alligator_Tas2r5

MTVRRNMFSSVSIIAITIVLIEISVGLLGNGFIVAINWTDWIKSRKLSSCNTILTSLGISRLLLQGTAIVFRSYSFFTLDTHKLDNVRITLRVIRMFANMTSAWLASCLSVFYCAKIATFTHPLFLRAKQRISGMVPQLLLGSLLLALFTSIPTVWANHDVYLCNSKGSLLGNTTSAKVNSNVIYLYFSFLYTVMAFFPFLIFLASSMLLMVSLWRHSRCMQDYAPDLQDSRTRAHVSAIKSLISFLILYTFSFVGETLQTMPTCLTDNTWTPAVTSVVVAAYPSGHSIVLILLNPRLKTALVQILRHIKCQLRLS

>American_alligator_Tas2r6

MLPPLLILLFTILGIEIIAGFMGNGFIAALIGSDWIRNRKISSSDMILISLGISRFVLQGTIIVYIHSLYFPGMPKLATLYKAFCILWMFVNHASLWFSTWLSVFYCVKIINFTQLILLRMKLRISGMVPWFLLGSVLVSSITTLPMFWIFPSISSHNSTGNHVNNSVKTTALDTSSLSIASLYCAGCFFPLTISFFTSVLLIVSLWKHTKKMQHNTTSCQDPRTNVHANAIKALVSFLILYLSSFIAQIPLILLASQNSHIWEVAVSLVVVAAYPSGHSIILILINSKLKQASVRLFNYTMYPLTKGTP

>American_alligator_Tas2r7

MSSLSVIIFIILYGVQFFVGIITNIFIVTVNVIDWTKDIKLSSNDQILVYLGLSNLFVQCTATAADFCFFFWTDLLYSGFSSQTFFFFVFFGSICASCFTGYLCTCYYVKITDSTYPLYLRMKMAFIKNLPWLLPWIIATSFGLSLAAVWDASKKVSLDMTANFSTNYTKPLLLFHYSTAFRIILLLLECIWPLIVTSFLVLKLIKTLCKHIRNMERTMAFGQPNLDAHKHATRTLTSLLILFISYNLLWSILVYDIFSYPSTGFLICITLFATLTSVQAITLILCNRRMKQKALRILQSIRQFSGG

>American_alligator_Tas2r8

MEGNRSNITEEDVARPSISLVIILLFTAFVGISVNTFIVAVNCTDWVRRKRLSTIDQILTILGFTRFSLSCIAVVDSFRQPFHSWPYGFIDKTFSILNWFLNVLNLWFGVCLGIFYCVKIANFSHHFCISLKLKISRLMPWLLMASVLLAIFNTFPAVTFIFKTQFKKSNSSIPENNKGEDIPQIIYFLHLFSLFVVGFPLCFTILCISTFLLLLSLWRHTRQLNSSSNNNPSMHAHILAVKIIMSFFIIHVIHFSAWLIVLAASIPNRKLQRLFLFQIANSCPLTHSVLLILSYPKLKQALTRILHYMGCAKGVS

>American_alligator_Tas2r9

MEGNRGNISKGNTWELDLSLLIAVLFETFLGISLNAFIIAVNCIDWVKKRCLSTSDQLLTILSFSRICLLLSEDADFVSSTFNPSFYHTKSTRLMLAGVAWFLSTSTLFFAACLSVYYCVKIANFSCRFFITLKRKISQLMPWLLLVSVMISLLNSLPIFIAIYNVSDNSCNSSCSQNHTGDNVTEDRILLDIIFGYSYGISIGFTILCISAVLLLFSLWRHIRHMQGSSAGAGKPSMEAHKKAVKIVMWVLFLNVIHFLVWLSFITFAVSPNIFVKHFLIQVTIFCPSIHALVLILSIPKLKQALARILHYVKCKGCVKKGIP

>Green_Anole_Tas2r1

MAAYPMYPFAIFSWSIIGILWIVSLSGNGFIFTVTVLQWLQKRKMPPCDFLLSCLSASRLLTQFNYMASYFLPFFYSPSIRKMFFFSRVFLHMASLWCVSWLSIFYCVKVINFSSSLLLWLKLRINLLVPKLLGISMVIFMVFSLPSIFTFHKFNKPCNQTITPPTSHEPEDSMWIRFFPVQITFTCINFSMNIAATLLLLISLWRHVRNLRKSGTSVQDLNTQVHLKVMRLLFITLLLYLLFIACSITMTTGFFHVQENQALISEIMISIFPSVHPIILIWTNPKLKDVAAHMLNIRERP

>Green_Anole_Tas2r2

MDNNLISPLGIFTWTIIEGISMVAILGNGFIIVVSGNRWLQTRKMVPSDFLLTSLSISRVFWHVTFGLSYVLEVSIGDIFMYSSAQEAIDFISTFSSMASLWCASWLSVFYCVKVTNFANRFLLWLKPRINVLSVRLLGMSISSLVFMSVPFFQHYAEAKKRCNLTGSLPLNTSQRNDCKFLLLIFRHFQVIVATMNFVISITATILLLTSLWKHTRNLKKSGIDAKDLSAQIHINVMKPLVLYIFLYLSYFAGILNFASHSVHNVDAVELLSDVLRTIFPAAHTITLVLSNPKLKALLVRTLNIRQKVNLVSKDEKTNQISKC

>Green_Anole_Tas2r3

MAANLVSPFAIISWSIIGILCIVSLSGNGFIFIVTVLQWLQKRKMPPCDFLLTCLSASRLLTQLNAMAIYFMELFYPSSRSAMLFFSWVLLNMASLWCVSWLSIFYCVKVINFSNSLLLWLKLRINLLLPKLLGISVVIFMVSSLPSIFTFHKCNEPCNQTVTPLINEKPGNMWISFFPVEITFTCINFSMNIAATLLLLISLWRHVRNLRKNGTSVRDLNTQVHLKVMRPLLITLLLYLLFIASLITMATGFLNLQTKQALIGEIMVTIFPSVHAIILIWTNPKLREVAAHMLNIRQSA

>Green_Anole_Tas2r4

MVSIPTSPVDILRWTILGIVSLFTLLGNGFIIVVLGYQGLQKKNILPHDILLIGLSASRIMLQLLSSANYILCFISETYRDTYKQDVVLLSWNVFNMTNLWSSTWLSVLYCVKVTNFANCLFLWLKPRINMLVLRLLGMSIVISIIFSVPSVIKYLQQKKWDNLTRNLSVSAIQCMDYKNRFIIFLDMQLFYVSITFCISLIASTLLLVSLWKHIRNLKKSGLGGKDLSTQVHINVITLLLSYIFFYLLYFTGFIILGTNVFNYESLERLIFKFLAISFPCVHCIMLILTNPKLKEMAGHILNITRRAS

>Green_Anole_Tas2r5

MAANLVSPFAIISWSIIGILCIVSLSGNGFIFTVTVLQWLQKRKMPPCDFLLTCLSASRLLTQLNAMAIYFMQLFYPSARSAMLFFSWVFLNMASLWYVSWLSIFYCVKVINFSNSLLLWLKLRINLLLPKLLGISVVIFMVSSLPSIFTFHKCNEPCNQTVTPLINEKADNMWISYFPVEITFTCINYSVNIAATLLLLISLWRHVRNLRKNDTSVQDLNTQVHLKVIRPLLITLLLYLLYIASLILMDTGFFYFQANRSLIGEIMVTIFPSVHAIILIWTNPKLREVAAHMLNIRQRA

>Green_Anole_Tas2r6

MFSLQITAFLVVAADLTLGGLISNGFIATVIIRKWIKCRSLASSEQLLLVLGISNVFAIILQTASVIGENVFICSDQLILPIIFFFVFFVTFFRFWLTAWLSLFYCIKIVNSTHVLLVWCKMRISWLIHRLLLGSLLISLFISFFAFHEFLFEFQSNRTASVANRTQEQTLRKTVDYFKVLFLAIGTSCPLLVVLFCSILSIVSLCRHIHRMTREKSSFRSIQAEAHLKAAQTMLSLLFFYVLFYVGETLSMTIHFENGKQISAIFVVLLYSHAQAAILVLVNSKLKRTATQILLRISQELCRHTVCSQI

>Green_Anole_Tas2r7

MRRQITQRYMISDIFFFYKMTTMVNNSISPFDILKWTILGIISVFTLLGNGFIIVVLGYQGLQKRNILPHDILLIGLSASRLMSQMLSSTSYLLYFENDMGIFNQDVVLICWNFFNMTSMWSATWLSVLYCVKVTNFANCLFLWLKPRINMLVLRLLAMSVVISSIFFVPSVLEYFQQKKWDNLTRNSPVSANQSEGYNNGFIILLDMQLFYVSITFCISVIASTLLLVSLWKHIRNLKKSGLGGKDLSTQVHMNVITLLLSYIIFYILHFTGFIILISDVSRFRSVATLVTSILITSFPCVHSIMLILTNPKLKVMAGHILGIMRRAS

>Green_Anole_Tas2r8

MRILFFRHHNVIPLRHFSQKVTAMALIMSPLGVLAWATFGILNVMALLGNGFIIVVNGHQWLQSKKMIPYKFLLTTLSTSRFLLQMDSVVGHFMYLIFAEIQKETHLYASRAEVVNFIWMFLNMVSLWNASWLSMLYCVKVTNFANRLFIWLKARVNMLVPRLLGISIIVSTVFFFPSAAKYYRKKKWCNLTDAVPRNSSQREGCNDAFDVFHFPQLFLASVNFGLTLTASCLLLTSLWKHTNNLEKSGAAFKDLSTQLHFKVMMPLLVSLLFYVLYFPCFVLAVGDIFEFGRLERWASEIVLPLYAFVQSIILILTNPELKKVAASILIIRQRAS

>Green_Anole_Tas2r9

MDNNLISPLGIFRWTMVGSISMVSILGNGFIIIVSGNRWLQNRKMAASDLLLTSLSISRIFLHVTFGLYYVLEVSIGDTYMCTFAYDAVIFACMFSSMASLWCASWLSVFYCVKVTNFANCFLLWLKPRINVLSIRLLGMSVISLMVIFVPFFWSYTEDKKRRNLTGSLPVNISQRMDCKALFFIFYPFQLSVLSMNFIITITANVLLITSLWKHTQNLKKSGIVAKDLSTQIHITIMKPLLCYILLYLLFFTGMLIFLGSFAYTFNGKGFLSDIIFTTFPSAHTIILILTNPKLKALLICHLNIRSKA

>Green_Anole_Tas2r10

MSPPFGIFSWSILGILWIVSLSGNGFIFTVTVRQWLKKRKMPPSEFLLTCLSASRLLTLLTTMVSYLPLFYSSGRSAMLLIPWVFLNMASLWCVSWLSIFYCVKVINFSNSLLLWLKLRLNLLLPKLLGISMVISMVSFLPFIFIFHQCNKPCNQTVTPPINHEDEADDSMWIRFIPLQITFTSINFSVNIAATLLLLISLWRHVRNLRKNGTSVQDLNTQVHLKVMRPLLITLLLYLLYIASLILMHTGFFYLQANLSLIGEIMITIFPSVHAIILIWTNPKLRGVAAHMLNIRQRA

>Green_Anole_Tas2r11

MDCSSISPLHILMWSIAITGNIVALLGNGFITVVQGHQWLQKRKILPCDFILINLSASRFMMLLSNSVNYILYSISSESYLRSYKKAYLMITWTFMNMASLWSATWLSIFYCVKVANFTNCLFLWLKTRINMLVPRLLGMSIVISSIFSVPSVIEYFGQIRGGNLTIILPLNVSQNERYAKGLFPLYLTYTSINVCISIIASSLLLASLWKHTRNLKKSGLSGKDLSTQVHKNVIIVVFSYVFFYLTFFTALIIEVTNVFKPRSPESLIVEILATSFPSTHCIVLILTNPKLKEMAARILNIG

>Green_Anole_Tas2r12

MSPFAIISLSILGILWIVALSGNGFIFTVTVLQWLQKRKMPPCEFLLTCLSASRLLTEFNSMAIYLSRLFYSSSKRAMLLIPWVFLNIATLWCVSWLSIFYCVKIINFSNSLLLWLKLRINLLLPKLLGISMVIFMVSSLPSIFTFFNYKEPCNQTVTPLSNQEADLSMWISFAPLQLTFTCINFIMNIAATLLLLISLWRHVRNLRKSGTSVQDLNTKVHLKVMRPLLITLLFYLLFIANLIVMIIDFFDLQTNLSLIGEIMVSVFPSAHPIILIWTNPKLKEVAAHTAQHQTKSLKKGDGGNRYPPPSSNRDCFYQCAR

>Green_Anole_Tas2r13

MFSLATIAFAVAAVVFALSGFLSNGFIAAVILREWTKSRSLASNEQLLLSLAASNFWATALLSPFYINATLRDYSISEIFLLPGLYLLATFVIMSRFWFTAWLCFFYCIKIVNSTHFLFLWCKLRILWLIPRFLAGSLFCSFLFSLFVLQITSRQAKSNITVNITNTTEVKSLKHTVNTFEAFFLAVGSGCPLLVVLLCSILVVASLCRHVCRIAGKDSHGRNLQTEAHIKAAWTVLSLLLLYVSYYAAQTLSIVVTLGKDDGTLVAMVRMVYPSAQASILMLVNPKLKQAAMQMLQRAKV

>Green_Anole_Tas2r14

MASNSPSPLDILIWTIIVIEYIVSFLGNGFIMVVHGHQWLQKRKMLPYGFLLISLSTSRYMMHLQSSLNYILHIAFSEPCIGFSITKIGDVNWIFFNMISVWADTWLSVLYCVKVTNFANCLFLWLKPRINNLIPRLFGMSIVISSIFSVPSVIYFLGQKSGGNLTVILPLNVSQNDPCSKHLLHLQLIYTSITFCTSVIASTLLLASLWKHTRNLKKSSLGGKDLRTQVHMNVIILLLSYVFFYLVFFTGLILFKTDVINMTILEILGTSFPSAHSIILILTNPKLKAVAVRILNISQRAS

>Green_Anole_Tas2r15

MANNMSMVEIMLLIIFEFVSIIGILGNGFIIVVNGYQWFQNRKMIPCDFLLTSLSTSRFIMQLDFFIYYILYLTLKINFKLFLDDFLFFSWMFFNMISNWCATWLSVFYCVKVANFANPLFLWLKARINMLLPRLLGLSIAVFMVSYLPSLVDYFGHTKWCNVTETLPENASQIEVCFMAPITFLPIQLSFYVINLCLSTIASILLLVSLWRHTRNLKKSDFGVKDLSTQVHIKVMAFLLFWIFFYFVDFIALIVYAGLNIGTDFGLVQELLVAIWMSAFPSAHSIILILTNPKLKEMCICIIKEMYAHIINIRHSTF

>Green_Anole_Tas2r16

MTPITSPVGVLTWATFGILNIMALLGNGFIIVVNGHQWLQSKKMIPCKFLLASLSTSRFLLQMDSVVGYFLYLIFVEIQKETQLYVSRAEVANFIWVFLNMVSFWCASWLAMFYCVKVTNFANRLIIWLKTRINMLIPRLLGLSIIVSTVFFFPSVANYYRRKKWFNSSDVFPFTGFLLPQLFLSSLNFSLTLTASCLLLTSLWKHMSNLKKSGVAFQDLSTQLHFKVLMPLLVSLLFYVLYFPCFVLAVGEIFEFGRIERLAFDIMLSLYAFVHSVVLMLTNPELKKVAASILNIRQRAP

>Green_Anole_Tas2r17

MTSDNIVKVDFPVWIIFGTLSLIGILGNGFIMAVNGLQWLQNRKIILCDFLLTSASTYRFIMQLTLLLYNIFYYIPENIHCIYRIDLLFFSWMFSNMISYWCATWLSVFYCVKVANFANPLFLWLKTRINMLVPRLLGLSIAVFTVSCLPSIVDYFGQTKWDNLTEILQENTSQRNICDIPHMTFLPIQLSFYVINLCLSTIAIILLLASLCKHIRNLKKSGVGIKDLSTQVHIKVMTFLLLWLFLYFLDFIGMIIYTNNTVKTIKLEGVLIDILMSAFSSAHPIILILTNPKLKEMSACIIKKMYARVINIRCSTL

>Green_Anole_Tas2r18

MSSPQIILFILGLVDLALGGLISNGFILTVILREWNKSRSLASIEQLILSLVLSNLGATLLVLPMFINDYIFPIFTTNITYLIMYPLSDYLILFRHWFTAWLCFFYCFKIVKSTHSLFLWFKLKTSWLVPQLIAGSLVVSLFIALPMFFLVLTDFLSNMTMNNTKISTEMLRNRTVKAPEIFFLIAGSGSPLLVILVCSILVVASLCKHVYRMKCKQHHSGSIQTKAHVKAAGTVFSILFLYLSFYMVQTLSMTVTVGKMEGTFLTVVVIAYPSAQAYILLLVNPKLNQAVNQMLPRRVT

>Green_Anole_Tas2r19

MSPFAIISWSILGILCIVSLSGNGFIFTVTVLQWLQKRKMPPCEFLLTCLSASRLLTELDCMAIYFMHLFSFSGSRRILYFFWIFFDMASLWCTSWLSIFYCVKVVNFSNSLLLWLKLRINLLLPRLLGISMTIFMVSSIHSIFRFFKYKEPCNQTVTPLTNEDTDISMWISFIPVQITFTCINFSMNIAATLLLLISLRRHVRNLRGNGTSVQDLNTQVHLKVMRPLFITLLLYLLFIVSLIIMISNFSLFQTNLSLSTEITMSIFPSAHSIILIWTNPKLREVAVHMLNIRQRA

>Green_Anole_Tas2r20

MDYSSISPLHILIWSIAITGNIVALLGNGFITVVQGHQWLLKRKILPCDFLLINLSASRFIMLLLNSVNYIMYFICSESSLISYKKAYTLITWTFMNMASLWSATWLSVFYCVKVANFTNCFFLCLKPRINMLVPRLLGMSIVISSIFSVPSVIEYLGQIRGGNLTVILSLNVSQNESYIKPLFHLQLTYTSINVCISIIASSLLLASLWKHTRNLKKSGLGSKDLSTQVHKNVIIVVFSFVFFYLAYFTASIIAASDVFKPHRPEFLIVDVLATSFPSTHCIVLILTNPKLKEMAARILNIR

>Green_Anole_Tas2r21

MSPPFGIFSWSILGILWIVSLSGNGFIFTVTVRQWLKKRKMPPSEFLLTCLSASRLLTLLTTMVSYLPLFYSSGRSAMLLIPWVFLNMASLWCVSWLSIFYCVKVINFSNSLLLWLKLRLNLLLPKLLGISMVISMVSFLPFIFIFHQCNKPCNQTVTPPINHEDEADDSMWIRFIPLQITFTSINFSVNIAATLLLLISLWRHVRNLRKNGTSVQDLNTQVHLKVMRPLLITLLLYLLYIASLILMHTGFFYLQANLSLIGEIMITIFPSVHAIILIWTNPKLRGVAAHMLNIRQRA

>Green_Anole_Tas2r22

MSPFGIFSWSILGILWIVSLSGNGFIFTVTVRQWLKKRKMPPCEFLLICLNASRLPTLLNTMAIYFMHLFYSSGRRMMLLIPWVFLNIASLWCVSWLSIFYCVKVINFSNSLLLWLKLRLNLLLPKLVGISMVISMVSSLSSIFTFHQCNEPCNQTVTPPTKHEAENCMWISFFPLKITFAFINFSVNIAATLLLLISLWRHVRNLRKKGTSVQDLNTQIYLKVMRPLLITLLLYLLYIASLITMSGFLNLQRKQALTAEIMITIFPAMHPRIIIWTNPKLKNVAAHMLNIKQRP

>Green_Anole_Tas2r23

MVSNSTSPLDILIWIIVGIVTMFSFLGNGFITIVQGHQWLQNRKILPCDFLLTSLSTSRFLMQLLSSVNYFLYFISLESYMNPIKQAIVYVIWLFFNMVSLWSATWLSVFYCVKITNFANCLFLWLKPRINALVLRLLGISIVISSISSLPSIIEYIGQKKGGNLTGSANHSEAYNHRNMLPLHVTFAFINFTINITATIVLLTSLWKHTRNLKKSGVGGKDFNTKVHFNIIIPLLFYVVFYFVHISSQIIVSNEITIVGSVKQRITDIMVSTFPTVHSIILILTHPKLRETVVRILNIKRRI

>Green_Anole_Tas2r24

MSPLDSIVFLVTAVVLTISGLISNGFIVTTITIKWIKFRSLASSELLFLTLSLSNFGAGVFLLPFYIDDSTIFSFKQNMALKILFPVAVFAVFSRFWLTAWLCVFYCIKIVNSTHFLFLWCKLRISWLVTHLITGSLVISFFASLGALEKDSIHLQSNVTTMFPTLSQGKSLKASDFRFQLFFLIFGSCSPLLIVFLCSTLVVASLSRHVCRMTDNNNFQRRAHFKATGTVLSLLLVYLSFFMAQILSMAANVTCTGRQFISSVMIAYAPVQAAILVLSNPKLKQALTVMVQRAKP

>Green_Anole_Tas2r25

MDNNLISPLGIFRWTMVGSISMVSILGNGFIIVVSGNRWLQNRKMAASDLLLTSLSISRVCLHVTFGLFYVLKVSIGDAYMGTSAYDAIIFACMFSTLASLWCASWLSVFYCVKVTNFANRFLLWLKPRINVLSIRLLGMSVISLVVISVPFFWSYAEEKKRCNLTGSLPVNISKRCQASLFIFHPLHLSVASMNFIITITANVLLIISLWKHTQNLKKSGILAKDLSTQIHITIMKPLVCYILLCLLFFTGMLFLSGSFVYTFDAKNFLSDIIFTTFPSAHTIILILTNPKLKALLIRTLNIR

>Green_Anole_Tas2r26

MDNNLISPLGIFTWTIIEGISMVAILGNGFIIVVSGNRWLQARKMVPSDFLLTSLSISRVFLHVTFGLIYVLEVSIGETFMYTFAWETISFVWVFSNMASFWCASWLSVFYCVKVTNFANRFLLWFKPRINVLSVRLLGMSISSLVFMSIPFFQSYAEEKKQCNLTKNLQVNVSKIEVCRALFLIFRRFQLIVVLMNFIISMIATILLLTSLWKHIRNLKKSEIGAKDLSAQVHINVMKPLVFYIFLYLSYFAGVLNFASHSVHNVDAMERLSDILLTIFPATHTIILLLSNPKLKALLVRTLNTRQKVDQEKGHQTCISCLQG

>Green_Anole_Tas2r27

MFSPGYIAFLVTAAVLNISGFISNGFIVTVMITAWTKSRRLASSEQLLLSLGLSNLWVTIVLIVFCFGFATLTNFNDQIFLFSFFSFAVVVRYWLTVLLCFFYCIKIVNSTHTFFLWCKLRISWLIPRLLVGSIIITLLAFVMILSFMYILPPPANVTTVIHAMSHSESIKSLIVFFLTVGSGCPFLLVLLCSILVVASLCGHVCQMTGKESHLRSFQTKAHVQAARTVLSLLLLFLSFFVAQTLSMTVDIGYNERLFIFTVMTIYSPAQAAILVLNNPKLKQALAVMVQRTVLICEEKN

>Green_Anole_Tas2r28

MSSPQLISFILALVDLALGGFISNGFILTVILRVWNKSRSLDSSEQLLLSLVLTNLWATVLVILTCINDYIIPMFPKSLMYSLNDFIIICRHWFTACLCVFYYIKIVNSTHSLFLWCKLRISWLVPRLIAGSLVVSLFLVLFMSFFTLINIQRNTTLIGTQMNEEISQHHNTGIHEILFLIVGSGSPLLVILVCCILVVASLCKHVYRMKSKEHNSRSIQTKAHIKATGVVLCILLLYLLFYVAQTFSLIVIKGKIEIILVTTTVYVYSCAQAYVLLLVNPNLNQAAIQVLPRRET

>Green_Anole_Tas2r29

MVNNMSTVKIFFLIIFEIVSFIGILGNGFIIVVNGHKWFQSRKMIPSDFLLTSLSTSRFIMQLSLLINYVLLFSLKNNFRFAVEDVMFFSWMFSNMISHWCATGLCVFYCVKVANFANPLFLWLKARINMHLPRLLGLSIAIFMVSCLPFLFEYFGHRKWCNLTEILPENASQSEFGDTPAIVFLPMQFSFYVINLCLSTIASILLLVSLWRHTRNLKKSGVGVKDLSTQVHIKVMAFLLFWIFFYFADLIALIIYADLINSIGTVQGLLLGISMSAFPSAHSIILILTNLKLKEMFDYIIKNICSYHRHQEQNMEKGHSLQDRKRHSLPI

>Green_Anole_Tas2r30

MDCSSISPLHILMWSITVIENIVALLGNGFITVVQSHQWLQKRKILPCDFILINLSASRFMMMLLTSVHYILYSISSESYLRSYEKAYLMITWTFMNMASLWSATWLSIFYCVKVANFTNCLFLWLKTRINMLVPRLLGMSIVISSIFSVPSVIEYLGQIRGGNLTIILPLNVSQNEHYTKRLLPLHLTYTSINVCISIIASSLLLASLWKHTRNLKKSGLGGKDLSTQVHKNVIIVVVSYVFFYLAFSTSLIIEVTNVFKPQSPETLIVEILSTSFPSTHCIVLILTNPKLKEMAARILNIG

>Green_Anole_Tas2r31

MDNNLISPLGIFTWTIIEGISMVAILGNGFIIVVSGNRWLQARKMVPSDFLLTSLSISRVFLHVTFGLIYVLEVSIGETFMYTFAWETISFVWVFSNMASFWCASWLSVFYCVKVTNFANRFLLWFKPRINVLSVRLLGMSISSLVFMSIPFFQSYAEEKKQCNLTKNLQVNVSKIEVCRALFLIFRRFQLIVVLMNFIISMIATILLLTSLWKHIRNLKKSEIGAKDLSAQVHINVMKPLVFYIFLYLSYFAGVLNFASHSVHNVDAMERLSDILLTIFPATHTIILLLSNPKLKALLVRTLNTRQKVDQEKGHQTCISCLQGKDNQISKC

>Green_Anole_Tas2r32

MTTMVSNSTSLLDILKSTYLGIVSVFTLLENGFIIVVLGYQGLQKRNILPHDILLIGLSASRLMSQMLSSTSYLLYFKKDMGIFKQDVVFISWNFFNMTSMWSATWLSVLYCVKVTNIANCLFLWLKPRINMLVLRLLAMSVVISSIFFVPSVLEYFQQKKWDNLTRNSPVSANQSEGYNNEFINLDVQLFYVSITFCISIIASTLLLVSLCKQIRNLKKSGLGGKDLNTQVHRDVITLLLSYIFFYIVHFTGFIILKNDVSRHRSLEMLVIQILTISFPCVHSIMLILTNPKVKEMAGHILNITQRAS

>Green_Anole_Tas2r33

MDNNLISPLGIFRWTMVGSISMVSILGNGFIIVVSGNRWLQNRKMAASDLLLTSLSISRVCLHVTFGLYYVLEVSIGDTYMYTSAYEAVSFACTFSSMTSLWSASWLSVFYCVKVTNFANRFLLWLKPRINVLSIRLLGMSVISLVVIPVPFLWRYAEEKKQCNLTGSLPVNITQRMVCKDLFFIFYPFQLSVLSINFIITITANVLLITSLWKHTQNLKKSGIVAKDLSTQIHITIMKPLVCYILLYLLFFTGMLIFSSRFAYTFNGKSFLFDIHLTTFPSAHTIILILTNPKLKALLIRTLNIRSKA

>Green_Anole_Tas2r34

MLSPQFIFFILAVIDLMLGGLISNCFIITVILREWTTSRSLASTEQFFLSLSLTNLGATVVLIPSYINAYIFPIFTRNFIMLIVYPLDDFLVLSRHWFTAWLIVFYCIKIVNSTHSLFLWCKLKISWLVPWLIAGSLVVSLFFALFKLYIILMKIQSNTTMIDIETNEEMSGYHTIGVHEILVLIVGSGSSLLIVLVCSILILASLCKHVYRLKCKEHHSRSIQTKAHVKASGTILFSLFLYISFYVVQTLVMTANVGKIEGTFLTIVVIAYPSAQACILLLVNRKFNQAATQILPRCDT

>Green_Anole_Tas2r35

MDNNLISPLGIFTWTIIEGISMVAILGNGFIIVVSGNRWLQTRKMVPSDFLLTSLSISRVFWHVTFGLSYVLEVSIGDIFMYSSAQEAIDFISTFSSMASLWCASWLSVFYCVKVTNFANRFLLWLKPRINVLSVRLLGMSISSLVFMSVPFFQHYAEAKKRCNLTGSLPLNTSQRNDCKFLLLIFRHFQVIVATMNFVISITATILLLTSLWKHTRNLKKSGIDAKDLSAQIHINVMKPLVLYIFLYLSYFAGILNFASHSVHNVDAVELLSDVLRTIFPAAHTITLVLSNPKLKALLVRTLNIRQKVNLVSKDEKTNQISKC

>Green_Anole_Tas2r36

MSSFQFILLILALVDLALGGLISNGFLLTVILREWNKSRSLDSSEQLLLSLVLTNLWASVILIPVYINDYIIPIYPRNFGKQIMYPLGDFLVISRHWFTAWLCVFYCIKLVNSTHSFFLWCKLRISWLVPRFIAGSLVVSLFFGFLMAFLNYRNIQSNTTMTDIKRKEDTFRYRSIDVPQILFLIVGSGPPLLMISGCSILVVVSLCRHMYRMKCKEHFSKNLQIKAHIKAAGIILSILFLYLIFYVVQTFSLLLTMKKMEGIFVTVLIIVYPCAQAYILLLGNPKLNQAAAQVFPRRET

>Saltwater_Crocodile_Tas2r1

MEGNRSNIIEEDITEPSISLLIILLFTVFAGFSVNTFIVAVNCTDWVKRKRLSTTDKILTILGFTRFSLSCIAVVASFHQLFHSWTYGFIEKTCSILNWFLNVLNLWFGVCLGIFYCVKIVNFSHRFCISLKLKISRLMPWLLLASVLLTICNTYPAVMFISKIQYKKSNSSIPENNKGEDIPQTLNLLQLFFLFGIGFPLCFTILCISTFFLLLSLWRHTWKLNSSSNNYPSMHAHILAVKIIMSFFIIHVIHISAWFMVMAARIPNRNLQRLFLFQIANGCPLIHSVLLILSNPKLKQALTRILHYMGCAKEGVS

>Saltwater_Crocodile_Tas2r2

MFSSVSIIAITIVLTEISIGVLANGFIVAINWTDWNKSRKLSSCNTILTSLGMSRLLLQGTAIGFRSYSFFTPDTHRLDNVRITLRVIRMFANMTSTWLASCLSVFYCAKIATFTHPLFLRVKQRISGMVPQLLLGSLLLALFTSIPTVWANHDVYLCNSKGNLLGNTTSAKVNSNVIYLYFSFLYTVMAFFPFLIFLASSMLLMVSLWRHSRCMQHYAPDLQDSRTQAHVSAIKALISFLILYTFSFVAETLQTMPTCLTDNTWTPAVTSVVVAAYPSGHSIVLILLNPRLKTALMQILHHIKCQKRQSFLNSATSC

>Saltwater_Crocodile_Tas2r3

MLPPLLILLFTILGIEIIAGFMGNGFIAVLICSDWIRNRKISSSDMILISLGISRFVLQGTITVYIHSLYFPGMPKLATLYKAFYILWMFVNHASLWFSTWLSVFYCVKIINFTQLILLRMKLRISGMVPWFLLGSVLVSSITTLPMFWIFPSISSPNSTGNHVNNSVKSTALDTSSLSIAPLYCAGCFFPLIISFFTSSLLIASLWKHTKKMQHNTTSCQDPRTSVHINAIKALVSFLILYLSSFIAQIPLILSASQNTPTWEVAVSLVVVAAYPSGHSIIVILMHSKLKQASVRFFTCTMYHLTKGTPKSH

>Saltwater_Crocodile_Tas2r4

MTPLSVVTLLGLVIEFVVGIVANGLIVGLNCIAWIKSKKLDSCALVLISLGTSRFFLLFTLLINNIFFVIPKKSNEQCNTWRAFFFIWMYLSTLSLWFATWLAVFYCVKITSFNQHLFLWLKSRLSGLLPWLILGSLLVSLATSLPAVNAIHIDYLNNSINNLSRNITVACIYETNTSLSFLILTMFGHYSPFVLFFVPSLLLVTSLLRHTKRMGENMSTSRDISAEAHIRAIKALLSFIVLYIFYMVAQLITLTMKFAASSSYLLWFCIMILAGYPSVHSVILILSNPKLKEAALKGLHNARRLQEDESQ

>Saltwater_Crocodile_Tas2r5

MVGNGFILAINLASWVRNRLVSSCDMILIFLSFSRLCLQSCMLMDFVCNLFYPSFYNQDDVFESFKAIFMFLNNSSLWFATWLGVFYCAKIANFSHSSFLWLKQNISSLVPWLLAGSLLFSFGSSLPFYWDIYKVYCNYSTAFPLANTTELKVIKDTNLFYVILLCNASLCLPTIVFVSSIVLLISSLWRHTKQMQNNGTGLRDPSTEAHRGAIKSVFSFLILYFFNLIALILTLSNIFSAYGTWEILCMIVMSAYPMVHSVILILGNPKLRRVSLKFLHYVSCHFRRGPK

>Burmese_Python_Tas2r1

MSVSALCNLLCLIIIIAVTLVGMLGNGFIVISDCCGWIRSKTQSPPDLLLMALSLTRLLFLGITLSLHCLSFLDINNPKYAGKAIVFFWAFFNAITLWITTCLGVFYCVKIINFTQPFLVKIKLRISRMVPHLLVAVMLVSLISALPVIWIGDCNCSYNTTQVLPNGGHKVFPRMTSQTFLLSLLYIIGTFPSFVIFLISSVFLIYSLVHHVKRMQQNPLGFRDQKMNVHLRATKILTSFLILYAATFVSEISMTFFPSPWTSVISIIVVTSYNSGHTIALIVMNSKLREGLSKMFHCLRKQT

>Chinese_alligator_Tas2r1

MTPLNVVTLIGLVIEFVVGIIANGLIVGFNCIAWIRSQKLDSCALVLISLGTSRFFLLHAILINNIFFAIPKKRIEQCNMWRAINFTWMYLSILSLWFATWLAVFYCVKITSFNQPLFLWLKLRFSGLLPWLILGSLLVSLATSLPSVNVIHINYLNNSIDNLSRNITVVCLYKTNTSLSYLILTMLGQYSPFVLFFVPSLLLVTSLLRHTKRMGENMSTSRDISAEAHIRAIKALLSFIFLYIFYMLAQFFTLTNKFATSSPYLIWLCIMILAGYPSGHSVILILSNPKLKEAALKGLHNARCLQEDESQ

>Chinese_alligator_Tas2r2

MQYRPKGSASTNPIPAEQRYAPTCFSRRDFVTSSDMEGNRGNISKGNILESDMSVLITVLFETFLGISLNAFIIAVNCIDWVSRICLLFAEHVELVTSTFNPSFYNSKSRYFMFAGLAWFLSTSNLFFAACLSVYYCVKIANFSCRLFITLKQKISQLMPWLLLVSVMISLLSSLPTFVAIYNVSDNSCNSSCSQNHTGDNVTQHRILLHMIIVFSFGFSIGFTILCISAVLLLFSLWRHIRHMQGSSAGVGKPSMEAHVKAVKMVLWFLFINLIHFLSWLSCITLIFTTNIFVQHFLIQVTIFCPSIHALILILSIPKLKQALARILRYVKCKGCAKEGIP

>Chinese_alligator_Tas2r3

MTVRRNMFSSVSIIAITIVLIEISVGLLGNGFIVAINWTDWIKSRKLSSCNTILTSLGISRLLLQGTAIVFRSYSFFTLDTHKLDNVRITLRVIRMFANMTSAWLASCLSVFYCAKIATFTHPLFLRVKQRISGMVPQLLLGSLLLALFTSIPTVWANHDVYLCNSKGSLLGNTTSAKVNSNIIYLYFSFLYTVMAFFPFLIFLASSMLLMVSLWRHSRCMQDYAPDLQDSRTRAHVSAIKALISFLILYTFSFVGETLQTMPTCLTDNTWTPAVTSVVVAAYPSGHSIVLILLNPRLKTALVQILRHIKCQQRQRLS

>Chinese_alligator_Tas2r4

MEGNRGNISKGNILESDMSVLITVLFETFLGISLNAFIIAVNCIDWVKKRRLSTSNQLLTILSCSRICLLLSEDAEFVSSTFNPSFYHTNSARLMFAGVAWFLSTSNLFFAACLSVYYCVKIANFSCRLFITLKQKISQFMPWLLLVSVMISLLSNLPIFIAIYNISDNSCNSSCSQNHTGDNVTGDRILLDIIFGYCFGFSIGFTILCISAVLLLFSLWRHIRHVQGSSAGAGKPSMEAHVKAVKMVMWLLLINVIHFLVWLSFITLAFSPNIFVQHFLIQVTIFCPSIHALVLILSIPKLKQALARILHYVKCKNCAKEGIP

>Chinese_alligator_Tas2r5

MVDKSCAFLSAIELSPIILFYLSIVAIESVIGIVGNGFILVINLASWVRNRVVSSCDMILIFLSFSRLCLQSCMLMDFVCNLFYPSFYNQDDVYENFKAIFMFLNNSSLWFATWLGVFYCAKIANFNHSSFLWLKQNISSLVPWLLAGSLLFSFGSSLPFYWDIYKVYCNYSTAFPLENTTELKVIKNTNLFYVIFLCNASLSLPTIVFVSSIVLLISSLWRHTKQMQNNGTGLRDPSTEAHRGAIKSVFSFLILYFFNLIALILTLSNIFSAYGTWDILCMLVMSAYPMVHSVILILGNPKLRRVSLKFLHYASCHFRGGPT

>Chinese_alligator_Tas2r6

MSSLSAIIFIILYGVQFFVGIIANIFIVTVNVIDWTKDIKLSSNDQILVYLGLFNVFVQCTATAADFCFFFWTDLLYSGFSSQTFFFFVFFGSICTSCFTGYLCTCYYVKITDSTYPLYLRMKMAFIKNLPWLLPWIIATSFGLSLAAVWDVSKKVSLDMTANFSTNYTKPLLLFHYSTAFRIILLLLECIWPLIVTSFLVLKLIKTLCKHIRNMERTMAFGQPNLDAHKHATRTLTSLLILFISYNLLWSILVYDIFSYPSTGFLICITLLATLTSVQAITLILCNRRMKQKALRILQSIRQFLGG

>Chinese_alligator_Tas2r7

MEGNRSNITEEDVAKPSISLVIILLFTAFVGISVNTFIVAVNCTDWVRRKRLSTTDQILTILGFTRFSLSCIAVVDNFCQPFHSWTDGFIDKTFSILNWFLNVLNLWFAVCLGIFYCVKIANFSHHFCISLKLKISRLMPWLLMASVLLAIFNTFPAVMFIFKFQHKKSNSSIPENNKGEDIPQIIYFLHLFSLFGIGFPLCFTILCISTFLLLLSLRRHTRQLNSSSNNNPSMHAHILAVKIIMSFFIIHVIHFSAWLFVMAASIPNRKLQRLFLFQIANGCPLTHSVLLILSNPKLKQALTRILHYMGCAKGVS

>Chinese_Softshell_Turtle_Tas2r1

MLTPVALISLILLGFESLVSNLGNGFIIVVIFSNWIKSRKLASCEHILISLSISRFLLQWLVMLSNFIYVSFPTTSALGCKHKAFGILWAYLNLVSLWCATCLSFFYSVKITNFTQPLFLWLKLRIAWLVPRLLLGSLIVSLVSTMPLIWSDIGFDLCNSTKSLERNTTWNDAKDIPYIFFVPVQILVLVIPFIIFLVSSTLLLISLWKHTKKMKNNVTRFKDLSVEAHIGAMKSLLSFFILYIMYFVTVMVLLTSIIKFQHPVRLPYEVLLSAYPSGHPVILILTNPKLKQVAVKILHQIKCQLREGTL

>Chinese_Softshell_Turtle_Tas2r10

MSLAIIIALSILVIELIIGIIANGLIIIVNCTAWIRSRKLTSCDMILTSLGISRFFLQCMIFANNIFFASSPVMDGQCDIWRIIYIVWMYLSILSLWFATWLSVFYCVKIANFSQPLFLWLKRRISGLLPKLLMGSLLVSLVTCVSSVNAIDRKYVGNSTNNMSMNTRVKCRPKSNLSSGLVILSMLGYSFPFFIFIIPSVLLIISLWRHTKRMEKNTSSSQDTIVEAHVSAVKGLVSFIFFYVSYFMAQVIFSIGTSAHSNVYFIWFWVVVMTAYPCGHAIVLILSNPKLKRAAVRALNYAKCRLKDGAS

>Chinese_Softshell_Turtle_Tas2r11

MGDNDYDIEQEDDITVPTIIMLVILVAEDFVGMWINSFIVAINCFERIKHRGLYSSDNILTVVAFSRFCVLLKTTLQTFCSIFYLEIYYMDTVFQAFRAVTWFLNSSNQWFAACLGVFYCVKIANFSHPLFISLKFRISRLVPWLLLVSVLFSLFSSLPFLNTPYKIRYNNFNSSLRGVYYTVKNVTVETSVSHVLFICGTGFSAAFTIFIISAFLLLFSLWRHTRRMQNNSSCFRSPCVEAHIQAMKAIMSFFLINVVNFIALLILLTNTLEETSLMGIACTIIIDACPSVHSIVLILSNPKLKTTLIKVLHYAKCKG

>Chinese_Softshell_Turtle_Tas2r2

MLSAIIIALIVLGIELIIGSIANGLMIIVNCLEWIRSRKLTRCDMILTSLGISRFFLQCMIFINSIVLQLCQDINRSCDTLSYFFVAWMYLSTLSLWFATWLSVFYCVKIATFSQPLFLWLRQKISGLLPPLLLSSLLVSLLTCFPSVNTVYRNSSMNNLSGNTTVESKCVIDLFSGLSMFSTIGFYSPFIIFIVSSALLITSLWKHSKRMRKAMSSSKDTITEAHVRAIKGLISFIFFYSSYFAVLVIFLIEIFRNNFGFLLLWGVIMAAYPSGHSVILVLGNPKLKKVAVKALHYAQCRLRNEVS

>Chinese_Softshell_Turtle_Tas2r3

MFFIFIALIILGMELIVGMIANGLMVVVNCLEWIRSRNLSYCDMILTSLGISRFFLQCMIIINSAVYQMFSEDNAFLALMGTINFVSNFVNTLSLWFATWLSVFYCAKIANFSQPLFFWLKCKILVLMPQLLMGTFLVSFVTSLPSVNSVNRKYINNSVTYLSGNTTGEWTYYANFSSGLFVLYMLSHSFPFIIFIVSSALLIMSLWRHTKRMEKNTGSCRDTVTQVHVRAIQGQLSFIFFHITYFVAQVILFTRLFSNSLSNAMWCIAIMVAYPAGHSVILVLSNPKLKKVAMRALHCARCRLRDEVS

>Chinese_Softshell_Turtle_Tas2r4

MFFIFIALIILGMELIVGMIANGLIVVVNCLEWIRSRNLSYCDMILTSLGISRLFFQCMIIINTTIYQISSEDNAHLDLLRTLDFLWCFTSTLSLWFASLLSVFYCAKIANFSQPIFLCLKWRLLGLMPQLLMGTFLVSFVTSLSSVYSIDRKYINNSMTNLPGNTTGEWTYYTNFSSGLFILYMLCHSFPFIIFIVSSALLIMSLWRHTKRMEKTTGSCRDTVTQVHVRAIQGQLSFIIFHISYFVAQVILFTELFANSLSTSVWCGVIMVAYPSVHSVILVLGNPKLKKVSMRALHCARCRPSDEVS

>Chinese_Softshell_Turtle_Tas2r5

MKKSLVPSDIFYLIISTMQLSAGVIANGFIVGLNCIYWVKFRTLTSYDMILTSLAFSRFCLQLFLSLNNFLYKFDPDIFYAFQTPNLFLVVWIFMNQVSLCFASCLSVFYCVKIATFNLSVFNWLKPKLSKLVPWLLLGSLLHSLITTVVFTFVSYFFEITSRNFTDHPSRNITITDKRKNLAKIVFLIHSIGSGFPLSLFIASSGLLILSLWKHIRKMNLNSDFNPSFRNPSTEAHLRAIKSVLSFLFLYIMYFAVSTVTIGILSHFTDEWKIIMFSFGVAAYPFVHSTILILGNPKLKQASAKILHSANCCFR

>Chinese_Softshell_Turtle_Tas2r6

MMKSHLAPSVIYYLIVLAIELSAGVVANGFIVGLNCIDWAKSRTLTSYDIIITSLAFSRFCLQFLVTIDNFFSMLFPNFFDIFERLHSYLVTWMFINQVSLCFATCLSVFYCLKIATLNQSLFRWLKLRISKLTPWLLLGSLLYCLVTTVCFTFFSYSYSVSFYNFTDNSTMSYNRKKAMEFTFLVHSIGSILPLIIFIASSVLLFLSLWKHIRKMNFNLDFIPSFRNPSMESHVRALKSVLSFFILYNIYYAASTFSIGYIPCFSEKWKAMFWTVLAAAYPSVHSIILILGNAKLKLSSSKILYCTNSCFRQVTS

>Chinese_Softshell_Turtle_Tas2r7

MQRNMFSFIISLIILGMELIVGMIANGLMVVVNCLEWIRSRNLTCCDMILTSLGISRFFLQCMIIINTTIYQISSEDNVHLDLMRTLDFLWSFTSTLSLWFASLLSVFYCAKIANFSQTVFLCVKGRLLGLIPQLLMGTFLVSFVTSLSAVYSIDRKYINNSVINLSGKTTGEWTFYTNISSGLFILYMLSHSFPFIIFIVFSALLITSQWRHTKRKEKNTGSYRDTVTQVHVRAIQGQFSFIIFHISYFVAQVILFTGFFAKSLSNSMWCLVIMVAYTSGHSVILVLGNSKLKKVALRALHCARCRLRNEVS

>Chinese_Softshell_Turtle_Tas2r8

MFFIFIALIILGMELIVGVIANGLMVVVNCLEWIRSRNLTCCDMILTSLGISRLFFQCMIIINTTIYQISSEDNTHLDLLRTLDFLWCFTSTLSLWFASLLSVFYCAKIANFSQTVFLCLKWRLLGLIPQLLLGTYLVSFFTSLASVYSIDRKYVNNSVINLSGKTTGEWTFYTNISSGLFILYMLSHSFPFIIFIVFSALLITSQWRHTKRKEKNTGSYRDTVTQVHVRAIQGQFSFIIFHISYFVAQVILFTGFFAKSLSNSMWCLVIMVAYTSVHFVILVLGNSKLKKVAVRALHCARCRLRDEVS

>Chinese_Softshell_Turtle_Tas2r9

MFSFIISFIILGMELIVGMIANGLMIVVNCLEWIRSRNLSYCDMIVTSLGISRFFLQCLIIINSIIYQISTEINTHIALMKTFSFLWSYTSILSLWFATWLSVFYCAKIANFSQAIFLWLKWRIPGLMPQLLMGSFLVSFVTSLPSVYFMDIKYIDNSVNNLSGKTMGEWTYNTNFFFGFSILYILGHSFPFVIFIVSSALLITSLLRHTKRMEKTTGSCRDTVTQAHVRAIQGQVSFIFFYIFYFVAQVILISGLFTDSLSNMLWFLVIIVAYPAGHSVILVLGTPKVKKRAVGALHCARCRMRDDVS

>Corn_Snake_Tas2r1

MSPLLQTIFQVAITAHSSMGIIASGFIVVAGYSTWLKGEKIPTCEVILMCLSSSRILLQGTILHCTFSSTLYLWNVLKIQSVLLVLSSTACLWFAACLSVFYCAKIATFTHRYFILVKLRIAEMVPMFLVGSAVVSLISCLPFIWMDDNILLCNSTGSHLKNLTVENHMRNISYLKAFSIYLTWAVLPLLLFVASSTLLIASLWRHTEQMRQSTMGLKDPRTKAHVEAIKSLISFLILYVCSFVADVLLGIPSCRARHEWKRNICLLVIAVCPSVHSVLLIFFNFRLKVVLKNILLYLACLQKKRLPVSPRLTTIHLVTFI

>Corn_Snake_Tas2r2

MLVSGVHNWLCLLIITAVTLVGMTGNGFIFISDCCDRIRSKTQTGSDLLLMSLSLTRFIFLGITLGFHCISFLDINQPQYAGSITIFFWTFFNAITLWITTCLGVFYCVKIVNFTQHFLVKMKLRISRMVPHLLVAVMLVSLISALPFFWIEDHTRFYNNTERIHEMMPQMFLFSMLYILGTFPSFLIFLISSGFLIYSLVHHTKRMQNNSLGFRDQRMDVHLKTTKILTSFLILYAATFVAEISMTFSPSPWASVMSNMVVSSYNSGHTIALIVMNSKLRERLSKMFQCLRKQT

>Diamondback_Terrapin_Tas2r1

MLTPVSLISMILLGLESLVASLANGFIIVVIFTGWIKSRKVASCELILTSLSISRFLLQWIVMLSNVIYIVFPRTSALGCKHKGFGILWNFLNMVSLWCAAWLSVFYSVKIANFTRPFFLWLKLRIAWLVPRLLLGSLMVSLVSTIPLVWTDVGIDLCNSRKIPEGNRTLNDTKDIPYLFFMPMEIIVSAIPFIIFLFSSILLLISQWKHTKKMKNNVTGLKDLSVAAHTNAMKSLLSFFILFIIYFVTIIVILAGTIRFQNPARLSYEVLLSAYPSGHPIVLVLTNPKLKQLSVKILHQIKCQLREGTS

>Diamondback_Terrapin_Tas2r2

MWINSFIMAVNCVECVKQRCLSSMIISWLSWHSQDFASCSKQLYRLFAQHLPEIYYCTPCSSIQSGHLVFELFQSMVRCLLVCILLCENCKLQPPLFIWLKFKISRLVPWLLLGSVLFSLFSSLPFLNAIYKIECNDFNSSLKRHYNAKNVTVETSVSQVLSICGTGFSMAFTIFIISASLLLFSLWRHTQQMQNNSSSFRSPCMEAHIQAMKTIVSFFLINIVNFIALLTLLTNIYQETSPASIACTIIVDACPSVHSVILILSNPKLKKTLIKVLHYAKCKR

>Diamondback_Terrapin_Tas2r3

MFPTIIICLIILGIEFITGIIANGLMIVVNCSEWIRSRKLTCCDMILTSLGISRFFLQCMININNILTHLPQDMNELCTILRIVTVFWIFLTTLNLWLATCLSVFYCVKIANFSQSLFLWLKLRISGLVPQLLMGSFLVSLVTCLPSVNTIDRKYIDHSMNTLSGNTTGECRYKVDFSSRFFISSMLGYTSPFIIFIISSILLIKSLWRHSKRMEKTTSTSRDTVTEAHVRAIKGLISFIFFYISYFVALVIFLLEISTISSYYVWIVIMGAYPSGHSVILILGNPKLKRVAVRAFHYAGCRLRGAAS

>Diamondback_Terrapin_Tas2r4

MFPIIIGLIILGIEFITGIIANGLMIVVNCSEWIRSRKLTCCDMILTSLGISRFFLQCMLMINGTVFQLCAEMNEQCAMLTTLTFVWLFLNTLSLWFATWLSVFYCVKIANFSQSLFLWLKRRISGLVPQLLMGSFLVSLVTCLPSVNTIDRKYIDYSLNCVSGNTTGECQYTFDFSSRFFILSMLGYASPFIIFIISSILLITSLWRHSKRMEKTTSTSRDTVTEAHVRAIKGLISFIFFYFSYFVALIFSELFANISSYYVWIVIMGAYPSGHSVILILGNPKLKRVAVRALHYAGCRLRGAAS

>Gharial_Tas2r1

MEGNRSNISQGNFLESDTFVLIVLFETFLGISLNAFIIAVNCIDWVKKRRLSTSDQLLTILSFSRICLLFAEDAEFAFLTFNPSFYYSQSAYLMFEGVAWFLATSSLFSAACLSVYYCVKIANFSCRFFITLKQKISQLMPWLLLVSVMISLLSSLPTFIALHNVSDNSCNSSCSQNHKGENVTHKNNFLDIVFINFFGFSIAFTILCISAVLLLFSLWRHIRNMQGSSAGVGKPSMEAHMKAVKMVMWVLFINVINFLVWLSCITFFLSSSIYVQRFLTQVSIVCPSIHALIQILSIPKLKRALARTLHYVKCKGCAKEGIP

>Gharial_Tas2r2

MEGNRSNITEKDIAEPSISLLIILLFTVFAGFSVNAFIVAVNCTDWVRRKHLSTTDKILTILGFTRFSLSCIAVVANFRQPFHSWTYGFIDKTCSILNWFLNVLNLWFGVCLGIFYCVKIVNFSHRFCISLKLKISRLMPWLLLASVLLAICNTYPAVMFMPKIQDKKSNSSIPENNKGEDIPQIVNLLQVFFLFGIGFPLCLTILCISTFLLLLSLWRHTWQLNSSSNNYPSMHAHILAVKIIMSFFIIHVIHFSAWFIVMAARIPNRSLQRLFLFQIANGCPLTHSVLLILSNPKLKQVLTRILHYMGCAKEGVS

>Gharial_Tas2r3

MDLLQYLFAAIGSEIEKSLLLTCLISLGISRFVLQGTIIVYIHSLYFPGMPKLATLYKAFCILWMFVNHASLWFSTWLSVFYCVKIINFTQLILLRMKLRISGMVPWFLLGSVLVSSITTLPMFWIFPSISSPNSTGNHVNNSVKSTALDTSSLSIASLYCSGCFFPLTISFFTSVLLIVSLWKHTKKMQHNTTSCQDPRTNIHVNAIKALVSFLILYLSSFIAQIPLILSASQNSHTWEVAVSLVVVAAYPSGHSIILILMHSKLKQASVRFFNCTMYHLTKGTPKSH

>Gharial_Tas2r4

MFSSVSIIAITIVLTEISVGLLANGFIVAINWTDWNKSRKLSSCNTILTSLGMSRLLLQGTAIVFRSYSFFTPDTHRLDNVRITLRVIRMFANMTSTWLASCLSVFYCAKIATFTHPLFLRVKQRISGMVPQLLLGSLLLALFTSIPTVWANHDVYLCNSKGNLLGNTTSAKVNSNVIYLYFSFLYTVMAFFPFLIFLTSSMLLMVSLWRHSRCMQHYAPDLQDSRTQAHVSAIKALISFLILYTFSFVAETLQTMPTCLTDNTWTPAVTSVVVAAYPSGHSIVLILLNPRLKTALVQILRHIKCQKRQSFLNSATSC

>Gharial_Tas2r5

MEGNRSNISQGNFLESDTFVLIVLFETFLGISLNAFIIAVNCIDWVKKRRLSTSDQLLTILSFSRICLLFAEDAEFAFLTFNPSFYYSQSAYLMFEGVAWFLATSSLFSAACLSVYYCVKIANFSCRFFITLKQKISQLMPWLLLVSVMISLLSSLPTFIALHNVSDNSCNSSCSQNHKGENVTHKNNFLDVVFINFFGFSIAFTILCISAVLLLFSLWRHIRNMQGSSAGVGKPSMEAHMKAVKMVMWVLFINVINFLVWLSSITLFLSPNIYVQHFLTQVSIFCPLIHALILILSIPKLKRALARILHYVKCKGCAKEGIP

>Gharial_Tas2r6

MVDKSCAFLSAIELSPIILIYLSIVATESVTGIAGNGFILAINLASWVRNRVVSSCDMILIFLSFSRLCLQFCMLMDFVCNLFYPSFYNQDDVFESFKAIFMFLNNSSLWFATWLGVFYCAKIANFNHSSFLWLKQNISSLVPWLLAGSLLFSFGSSLPFYWDIYKVYCNYSTAFPLANTTELKVIKNTNLFYAILLCNASLCLPTIVFVSSIVLLISSLWRHTKQMQNNGTGLRDPSTEAHKGAIKSVFSFLILYFFNLIALILTLSNIFSAYGTWEILCMIVMSAYPMVHSVILILGNPKLRRVSVKFLHYASCHFRGGPK

>Gharial_Tas2r7

MTPLSVVTLLGLVIEFVVGIVANGLIVGLNCIAWIKSKKLDSCALVLISLGTSRFFLLCTLLVNNIFFIIPKMNNEQCNTWRAFYFIWMYLSTLSLWFATWLAVFYCVKITSFNQHLFLWLKLRLSGLLPWLILGSLLVSLATSLPTVNAIHIDYLNNSINNLSRNITVACIYETNTSPSFLILTMFGHYSPFVLFFVPSLLLVTSLLRHTKRMGENMSTSRDTSAEAHIRAIKALLSFIVLYIFYMVAQVVTLSKKFAASSPYLLWFCIMILAGYPLVHSVILILSNPKLKEAALKGLRNARCLQEDESQ

>Gharial_Tas2r8

MSSFSAIIFLIIYGVQFFVGIITNIFIVTVNVIDWTKDINLSSNDQIIVYLGLSNLFVQCTATAADFCFFFWTDLLYSSFSSQTFFFFVFFGNFCTFWFTGYLCVCYYVKITDSTYPLFLRMKMTFIKNLPWLLLWMTVASFGLSLAAVWDVSKKVSLDMTANFSTNYIKPLPLFHNSNAFRIILILLECTWPLIVTSFLVLKLIKTLCKHVRNMERTMAFGQPNLDAQKHATRTLASLLILFISYYLLWSILLYDIFSYPSTGFLICFTSLATLISIQATTLILCNQRMKQKALRILQSIRQFAGG

>Green_Sea_Turtle_Tas2r1

MIIVNCTEWIRCRKLTCCDIILTSLGISTFFLQRTIFINNIFFALSPVLNGQCDIWRNLYFLWMYLNTLSLWFATWLSVFYCVKIANFSQPLFLWLKQRISGLMPQLLMGSLLVSLVTCLPSANVIDRKYIDNSTNNLSGNTRVECRHNSSSGLVILYMLGYSFPFFIFVVSAVLLITSLWRHTSRMEKNTNLSSDTITEAHVSVIRGLIAFIFFCISYFVAIVPFLLKVFALSNLYFRWFCVVIMAAYPSGHSVILILGNSKLKRVAVRALNYAKFWLRDEAS

>Green_Sea_Turtle_Tas2r2

MFPAIIIGLIILGIEFITGIIANGLMIVVNCSEWIRSRKLTCCNMILTSLGISRFFLQCTIIINNILFQLPHTMNELCAMLRTLAVVWMFLNTLNLWFATWLSVFYCVKIANFSQPLFLCLKRRISGLMPQLLIGSFLVSLVTCFPSVNGIDRKYINNSMNNLSETTIGECQYKVDLSSSFFILSMLGYSFPFIIFMMSSILLIISLWRHSKRMKKTTSSSRDTITEVHFRAIKGLVSFIFFYISYFVALVIFLLEISTINTYFLWIVIMGAYPSGHSVILILGNPKLKRVAVRALHYAGCRLRDAVSQNCS

>King_Cobra_Tas2r1

MSPLLRTIFQVAITAHSFMGIVASGFIVVAGYSTWLKGKKVPTCEVILMCLSSSRILLQGTILHCTFSSTLYLWNVLRIQTVLLVLTSTACLWFAACLSVFYCAKIATFTHRYFVLVKLRIVKMVPMFLGGSAVVSLISCLPFIWMDDNVPLCNSTGNPLKNVTVENHAGSISYLKVFSIYLIWAVLPLLLFVASSTLLIASLWKHTEQMRQSTMGLKDPRTEAHVAAIKSLISFLILYICSFVADVLLGFPSCEARREWKRNTCLLVIAVCPSIHSVLLIFFNFRLKMAFKSLLLYLVCRQKKDYR

>King_Cobra_Tas2r2

MSVSGVHNWLCLLIIIVVTLVGMTGNGFIFISDCCDWIQSKTHSGSDLLLMSLSLTRFIFLGITLGFHCISFLDINQPKYAGSVTIFFWTFFNATTLWITTCLGVFYCVKIVNFTQPILVKMKLRISNVVPHLLVAVMLVSLISALPFLWIEDHTQSYNNTERRQEMMAQMFLFGMLYILGTFPSFVIFFISSVFLIYSLVHHAKRMQNNSLGFRDQRMDVHLKTIKILTSFLILYAATFVAEVSMTFSPSPWASVMSITVVSSYNSGHTVALIVMNSKLREQLSKMFQCLRKRHEGLSPD

>Painted_Turtle_Tas2r1

MFPTIIICLIILGIEFITGIIANGLMIVVNCREWIRSRKLTCCDMILTSLGISRFFLQCMININNILTHLRQDMNELCTILRIVTVFWIFLTTLNLWLATCLSVFYCVKIANFSQSLFLWLKLRISGLVPQLLMGSFLVSLVICLPSINTIDRKYIDHSMNNLSGNTTGECRYKVDFSSRFFISSMLGYTSPFIIFIISSILLIKSLWRHSKRMEKTTSTSRDTVTEAHVRAIKGLISFIFFYISYFVALVIFLLELCTISTYYVWIVIMGAYPSGHSVILILGNPKLKRVAVRAFHYAGCRLRDELS

>Painted_Turtle_Tas2r2

MFPVIIICLIILGIEFITGIIANGLMIVVNCSEWIRSRKLTCCDMILTSLGISRFFLQCMLMITGIFFQLCAEMNEQCAMLTTLTVVWLFLNTLSLWFATCLSVFYCVKIANFSQPLFLWLKRRISGLVPQLLMGSFLVSLVTCLPSVNTIDRKYIDYSLNCLSGNTTGECQYTFDLSSRFFISSMLGYASPFIIFIISSILLITSLWRHSKRMEKTTSTSRDTVTEAHVRAIKGLISFIFFYISYFVALISSELFANISSYFILLWVVIIGAYPSGHSVILILGNPKLKRVAVRAFHYAGCRLRGAVS

>Painted_Turtle_Tas2r3

MLTPVGLISMILLGLESLVASLGNGFIIVVIFIGWIKSRKVASCELILTSLSISRFLLQWIVMLSNVIYIVFPRTSALGCKHKGFGILWNFLNMVSLWCAAWLSVFYSVKIANFTRPFFLWLKLRIAWLVPRLLLGSLMVSLVSTIPLVWTDVGFDLCNSRKIPEGNTTLNDTKDIPYLFFMPMEIIVSAIPFIIFLFSSILLLISQWKHTKKMKNNVTGLKDLSVAAHTNAMKSLLSFFILFIIYFVTIIVILAGTIRFQNPARLSYEVLLSAYPSGHPIVLVLTNPKLKQLSVKILHQIKCQLREGTS

>Painted_Turtle_Tas2r4

MMKKSLAPAVIFYLIISAMELLAGVVANGYIVALNCINWVKSRKLTSYDKIITSLAFSRFCLQVFVSSDNFLYKLYPDFFYMTETSSPYAVIWMFINQVSLCFASCLSVFYCVKIASFNQALFNWLKLKLSKLVPWLLLGSVLYCLVTTVAFAVFTYSSHNSTDCLSRNVTISDNNKNRAVFTFLIHGIGSISPIILFIASSALLIISLWRHIRKMNLNSDLNPSFRNSTMDAHVRALKSVVFFFILYNIYYMASTLSIGSLAYVSDELRITVCTIIIAAYPSLHSIVLILGNPKLKLASARILHSANC

>Painted_Turtle_Tas2r5

MKKSLATAFIFYMIISAIEFSVGVVVNGYIVAVNCIDWAKSRTLTSYDKIITSLALSRFCLQFFMTDYSLFKLYSHLFVRFQTAQLYIAIWLFINQMSLCFASCLSVFYCVKIATFNQSLFSWLKLKISKLVPWLLLGSVLYCLVTTVAFTLFSYSYCLSSHNSTDRLSTNSTMSDKIKNLMELTFLIHSVGSIFPLIVFIVSSVLLIISLWRHIRKMNLNSDLNPNFRNPSTDAHVRALKSVVSFFIVYNIYYVASTFSIGNISYLNAEWKMRVFLFISAAYPSVHSIILILGNPKLKLASGKILHSANCCFR

>Painted_Turtle_Tas2r6

MMKKYLAPAVIFYLIILAIELSAGVITNGYIVALNCINWANSRTLTSYDKIITSLAFSRFCLQILVTLDNVLSKIYPNIFDRFQTLQPYLVTWMFINQVSLCFASCLSVFYCVKIASFNQSLFSWLKLKISKLVPWLLLGSMLYCLVTTVAFTLFSYSYWVFSHNSTDCLSKNGTISDNKNNLLEFTFSVGSISPLIVFIASSVLLIISLWRHIRRMNLNSDFNPSFWNPSMDAHVRALKSVVSFFIIYTIYYVASTFSIGNLSYFNDELKIMVCTFVAAVYPSLHSIILILGNPKLKLASAKILHSANSCFGEVTS

>Painted_Turtle_Tas2r7

MGDNDCNFVKEDDITNSSIIVLVILVCEAFVGMWINSFIMAVNCVECVKQRCLSSTDNILAVLAFSRFCFLLKTTLQTFCSTFYPEIYYLHSVFQAFRAVTWFLNSSNQWFAACLCVFYCVKIANFSHPLFIWLKFKISRLVPWLLLGSVLFSLFSSLPFLNAIYKIECNDFNSSLKRHYNAKNVTVETSVSQVLSICGTGFSMAFTIFIISASLLLFSLWRHTQQMQNNSSSFRSPCMEAHIQAMKTIVSFFLINIVNFIALLTLLTNIYQETSTASIACTIIVDACPSVHSTILILSNPKLKKTLIKVLHYAKCKR

>Brown_Spotted_Pit_Viper_Tas2r1

MSVSGVHNWLCLIITTAVTLVGMTGNGFIFLSDCHDWIRSKAPSGPGLLLMTLSLTRFIFLGITLGFHCFGFLDINRPKYAGRVTIFFWTFFNATTLWITTCLGVFYCVKIVNFSQPFLVKMKLRISSMVPHLLVAVVLVSLISALPFLWIDDHNQSDNPEGVREVRVQMFLFSILYILGTFPSFVIFLISSGFLIYSLVHHVKRMQNSSVGFRDQRMDVHLKTTKILTSFLILYAATFVAEISMTFSPSPWTMVISNIVVSSYNSGHTVALIVMNSKLRGRLCKMFWCFRKQT

>Japanese_Gecko_Tas2r1

MAYLLLVTGFILLVMETLSGMITNGFIVLIICMDWFKSKKLPPTYLILGCLGLSRLLWQAIVILKVTMTFFFRSTYIQNYTRLTFITMWLFANTLNLWFAAWLSVMYFVKIAIFSHPVFLQVKQRFSGLLLRLLLGSVAFSAFMNIAIITASNYGLSTCNPYKIQPSNFSDTDIKISHSCKYFIIVTTAPHCLPIMIFLSSSVLLITSLWKHTRRLRCNRTGPRDLSTQTHLTAIKALASFAILYLFSFVAFTSQSLLIWISRRRDWTSIFFENASAVYPTGHAIILILINPKLKQAWVRMMHHLKCHLREA

>Japanese_Gecko_Tas2r10

MSDEMSASLRSLCLSVFGMMNIVALLGNGFIIAVNGYSWLQSRKLIPCGLLLSCLSASRFLTQGILTINQCLYLRSPGTYEFSCTEQFMNMAWNYCNMASFSSDTALNVFYCLKITTFAHPPFPWLKSRIDRFMPRLLAIPCIAFVFFSLPSYVVYLSQGSCHFLRRNMTERRNPKNNKVFKMLSPVQFTLPALCFLICSAASVLLFISLWRHMRNLKKNGLDVKDLSTRAHLSVMKSLLCFLFFFVLYFVATNVAFFSDFRISSLEHLISIILLSSYPSVHSIVLIVTNPKLKETCVRILNIGKRSSCSSSNSRAE

>Japanese_Gecko_Tas2r11

MATLLTKVSVVFLVIEALIGIVANGSIIIINFIDWFRNRKLSPTDLILVCLGSSRLLLQAVVILGATLFNNTPSNVPFTLMGIWIFSNVVNLWFAACLSVFYLAKIAVFSHPLFLQIKRGLPWLVPWLLLGSVVFSVVRTMIVTMSWNYGFFTCNPYKLLSINRSNAEMNMSPVCTHLTALTAVFDFIPFMVFLSSTIFLVSSLWKHMRCVQRNGTGTGDFNTQAHLRAIKALASFAVLYLISLTAIISQPVLAWNKHTWTILLFNVSAVYPSGHAIILILINPKLKQAWVRMIHHFKCHLRKVPS

>Japanese_Gecko_Tas2r12

MATLLTKICFVFLVMETLFGMVANGSIVLINFIDWFRNRKLSPTDLILICLGLSRLLLQAVVILDVTIFILLNSTVSKVLFALNGVWMFTNMMNLCFAACLSGFYLAKIAVFSHPVFLQFKQRFPGLVPWLLLGSVVFSVLRTLLVILRWNYAFSTCNPFKSLLSNRSNAEMKMPPVCRHITALNAVFDFIPFMVCLSSSIFLISSLWNHMRRVQSNGIGTRDLNTQAHLRAIKALASFVVLYLISFAANTSQAELVWNYQHTWTTLLFNVSAVYHSGHAFILILINPKLKRAWVRMIHHFKCHLRKVPS

>Japanese_Gecko_Tas2r13

MATLLTLVCFVFLVMETLIGMVANGSIIVINFFDWFRNRKLFPTDLILICLGLSRLFQQAAVILDITLLILFNRVLSNLLFMMTIIWTFTNTLNLWFATCLSIFYLAKIAVFSHPVFLQIKRRLPGLVPWLLLGSVIFSVVRTMIVITNWNYGLSTCDPFRPLSINRSNVEMNLPPVCGHITALTTVFDFIPFMVFLSSSIFLISSLWKHMRRVQSNGTGTRDLNTQVHLSAIRALASFAVLYLISFVTTISQLVLVWENQHTWNILLFNVSALYPSGHAIILILINPKLKQAWVRMIHHFKCRLGKVPS

>Japanese_Gecko_Tas2r14

MAPLLTIVCFVFLVMETLIGMLANGSIVLIHFIDWFRKRKLSPTDLILICLGSSRLLLQAVMILGVTLFIWFNHITSNLVFTMIIGWMFTNTVNLCFAACLSVFYLAKIAIFSHPVFLQVKRRLPELVPWLLLGSVVFSAVRTLIVTTSWNYGFFTCTPYKLLSINRSNVEMNMPPVCMHITALAAVFDLIPFMIFLSSSIFLISSLWKHMRCVQRRGIGAKDLNTQAHLSAIKALASFAVLYVISFAAVTSQFVLGWTKQYTWTIPLFNVIALYPSGHAIILILINPKLKQAWVRMIHHLKCCLG

>Japanese_Gecko_Tas2r15

MATLLTKVLFPFLVMETLIGMVANGSIVLIHFIDWFRKRKMSPTDLILICLGVARLLLHAVIILDATLFTLHKNTLSNIHFTLNSVWTFMSTVNLWFAACLSVFYLAKIAVFSHPVFLQVKQRFSGLVPWLLLGSVVLSAVVTIIIITSWSLSTCNPYEPLSINKSNAEMNMPDMCWHIAAQSAVFDFIPFMVCLSSSIFLISSLWRHMRRVQSNGTGTRDFNTQAHLSAIKALASFAVLYLISIAADISQFVLVWDNQHTWSILLFNVSAVYPSGHAIILILINPKLKQAWARMIHHLKCRLRKVPS

>Japanese_Gecko_Tas2r16

MATLLTIVCILFLVMETVIGMVANGSIFLIFFIDWFRKRKLSPTDLILICLGLSRLLLHGVMILYVTLFTLLNNTRINLLLALTGVWIFTNTVNLWFAACLSVFYLAKIAVFSHPVFLQVKRRFSGLVPWLLLGSVVVSAVVTIIMITSWQYGYSTCSPYKPLSINRSNAEMNTQNVCRHIITLVAVFDFIPFMAFLSSSIFLISFLWKHMRRVQSNGTGTRDLNTQAHLGAIKALASFAVLYLISFAVDTSHSVLVWDSQHTWTTVLYNVSVVYPSGHAIILILINPKLKQAWVRMIHHFKCHLRKVPP

>Japanese_Gecko_Tas2r17

MATLLTIVCFVFLVMETFFGMVANGSIVLINFIDWFRHRKLSPTDLILICLGLSRLLLQAVMILGVTVFTLLNDIRRNVLITLGSIWVFTNTVNLCFAACLSVFYLAKIAIFSHPVFLQFKQRFSGLVPWLLLGSVVFSAVVTIIFITSWSYDLSTCNPYKSLSSNRSNAEMNMPHVCKHLATLTAVFDFIPFMIFLSSSIFLISSLWKHMRRVQCNGTGTGDLNTQAHLSAIKALASFAVLYLISFAVDASHSVLVWDSQHTWTPVLYNVSAVYPSGHAIILILINPKLKQAWVRMMHNLKCRLRKVPS

>Japanese_Gecko_Tas2r18

MLCCVLLMGTVSIAAEESLLFSFVLRKMATLLSITGFALLIMETLVGLVANGFIVFINCIDWYRNRKLSPTDLILICLGSSRIMWQALVMLHVTMLSFFLHTYVLKRVHLIVMIMWFFTETINLCFAACLGVWYLTKIAIFSHPIFLQVKQRFAGLLPWLLFGSVVFSSFMTIINFTESFSGLAICDPYKLVLSNSSDSEMQKSHSCMVLVFLRIVSHFIPSVIFLSSTVLLIISLWKHIRHLQHNGTGVKDINTRVHLTAIKALASFAILYLFSLVAINLQSMLVWGSNDLSWTSVLFHNVSDVYPSGHAVILILINPKLKQAWIRMIHHLKCHVSEASS

>Japanese_Gecko_Tas2r19

MMVVMDRDQFSPLGVFFVIIFGIESIVSLLGNGFILAVCGHSWLRSKKMLPCDFLLTTLSLSRFLFQLVSTSSQFLFFSSPETYLVSETEHAFKLSWAYLNIASLWCATWLNVFYCVKVTNFPHPLFAWLKLRIGALVPRFLGISLLAFIICSIPPVLRSFENEKCCNLTGNLPENTSQSEVHHRHSRMALKSFQVYFTAINCIICLTASLVLLLSLWRHMRNLKKSGLSTKDFSTQAHLSVMKSLLLSLFFYILHFTAVIFALTHTFRYGNLKRLISDIFHILYPSAHSVILIVTNPKLRKACTHVLDLRSAS

>Japanese_Gecko_Tas2r2

MEVSAFVIISSIFLVIETLVGLVANGFIVLMNYIDWFRSRKLSPNDQILTCLALSRLMWLAFVILNMIADFYSMDKHNCHYVYLMLPILWIFTNTTSTLLATCLSVFYLTKIATFSHPVFLQVKLRFSGLVPWLLPSSVVFSAITAIFLVTGLSNGFSMCDSNKSLLNITDSGIKLSDLYMYIDILAIVPNFIPLMIFLSSSILLMTSLWMHRRRMQRNGTGIQDLNTQVHLTAIKALASFAVLYLSSFLAVIAQAVLTWNDMSKTWLFMLLSNVTVSSPSGHAVILILIHPKLKQAWIRMLLHFKCCSTEVLS

>Japanese_Gecko_Tas2r20

MVAMDPNLFSPLGVFFLIIFGIESIVSVLGNGFVLAVSGHSWLRSKKMLPCDFLLTILSLSRFLLQWVTTGSQVAYFSSPETHVYSKEHQAFAFSWVYLNTASLWSATWLNVFYCVKVTNFTHPLFSWLKVRIGVLVPRFLGISLLIFIISSIRPVMRSFEDEKCHNLSGSLPENTSQSGVHGSNCVVFLNTLHIYFTAINFSICLTASLVLLLSLWRHMRNLKKGGLSTKDLSTQAHLSVMKPLLLSLFFYILHFAAMILVFTKVYRYGNLGQLICEILLSSYPSAHCVILIFTNPKLRKACTLVANLRRSAS

>Japanese_Gecko_Tas2r21

MVVMHRDLFSPLGFFFLVVFGIESIVSLLGNGFVLVVNGHSWLCNEKMLPSDFLLSTLSLSRFLLQWISMSSQFVYFSSPETYRDHEKLWAFTLSWVYLNTASLWCATWLNVFYCVKVTNFAHPLFVWLKLRIGVLVPRFLGISLLTFIICSVPSIMRSFENEKCCNLTGNLPETTSQSEAHQDYSLVFLNTLHAYFISINFSICLTASLVLLLSLWRHTRNLKKGGLSTKDFSTQAHLRVMNPLLLSLFFYILHFAAMILALNVFTYGKLERLICEIFLSSYPLAHSIILIFTNPKLRKVCIHVLNIRRSKCLMKRK

>Japanese_Gecko_Tas2r22

MGSGSDSSTTGELFTINMITILVGTILLLGSMWMDVFILTVSGRDWMKKKCLSTTDGILTLQGSIRIYLWCTNLVWHILDKFCPWVTGIGYVLGVYTFSFWYLLSCNIWLTTSLCTYYCVKIADFSHPFFIHLKLKLSGLVSTWLLGSAILSLASTLPLAYTILETEDNRNFSNFFQNKNETDISNTGMAFRMYVLLALGPSAAFSICAISAVLLLFSLWRHRQRMLDSWGGHKGPQTDAHFQAVITILFLLVNNVAVFVSIELVLSSSYGGSSVSPFGPSTVLYCCFSVESLISVWGNKKLKNKLRRSFHLARCRGCVRWLHTHSNCV

>Japanese_Gecko_Tas2r23

MIFILVGTILMLGRTWMDVFILTVNVRDWMEKKRLSTTDRILTVQGSIRIYLGCTEIIWLILEKFCPWITRVGYVFKAFKFTLWYLMSCNVWLTASLCTYYCVKIADFSHPFFIHLKLRLSGLAATWLLGSAILSLASTLSFAYIDLEIRDNSNFSNCSQNKNVADISHTGMAFRMYVLLGLGPFAAFSICAISAVLLLFSLWRHRQRMLGSWGGHRSPQTDAHFQAVITILSLLVNNVVVFIAILLLLSRGFERDLISQRDPSVLLYCCFSVESLISVWGNKKLKNELIRSLHFAKCRGCFR

>Japanese_Gecko_Tas2r24

MGSGSDSGTTGEFFDRNMITILVGTILLLGRMWMDVFILTVNGRDWMEKKRLSTTDRILTLQGSIRICLGCLELIQSILEKFCPWVTRIGYVRKAIEFSFRYLISCNIWLTTSLCTYYCVKIADFSHPFFVHLKLRLSGLVSTWLLGSAILSLAGTLPLTYFNLEIQDNRNSSNFSQNKNETDISHTGMAFRLYVLLALGPSAAFSICAISAVLLLFSLWRHRQRMLGSWGGHKSPQTDAHFQAVITILSLLVNNVIIFICNQLVLSRSYAGSSVSGFGPSIVLYCCFSVESLISVWGNKKLKNKLRRSFRFAKCRGCVS

>Japanese_Gecko_Tas2r25

MTIILIMTILLLGSTWMDVFILTVNCRDWMEKKRLSTTDRILTLQGSIRICLGCLELIQSILEKFCPWVTRIGYVLEVYTYISWYLTFYNVWLTASLCTYYCVKIADFSHPFFVHLKLRLSGLVSTWLLGSAILSLASTLPLAYTTMEIRDNRNFSNFSQNKNETDIARTVLLGLGPFAAFSICAVSAVLLLFSLWRHRQRMLGSWGGHRSPQTDAHFQAVITIVSRLVTSVVVFISIQLFLSSGYGGNSISQLGPSIVAYCCFSVESLISVWGNKKLKNELRRSFCFAKYRGCVS

>Japanese_Gecko_Tas2r26

MLCCELLMESTSIAAEETLLFSLVLRNMATFLSITAFILLIMEILIGTVANGFIVLISCIEWIRSRKLSPINLILICLGSSRLMWQAIVMLHVTMFSFFPHTYILKQVHSIITVIWFFTDTVNLCFAACLGVWYLTKIAIFSHPVFLYVKQRLSGLLPWLLLGSVAFCAFMTVIVFARSFSDLAICDPYNLLLNNSFDLEIQKSRTCMDINISRTVPKVIPSVIFLSSTVLLIISLWKHTRHLQHNGTGVRDINTNVHLSAIKALASFAILYLSSLVAINLQTVQVWGSNHDSWRTVLFHNVSVVYPSGHAVILILINPKLKKAWVRMIHHLKCCVSEAPS

>Japanese_Gecko_Tas2r27

MATLLSIIGFALLLMETLVGMVANGFIVLISCTEWIRSRKLSPTDLILTCLGLARFAWQAIVILEVTMYSFFLRIYQLNHCRLVLNMLWLFTYNVNLWFAAYLSILYFVKITTFSHPLFLQVKQRFSRLLPWLLLGSVAISAFMTMIMTVITVLSSDLINCDPYKLLLNDSFDLEIKKAPSCMDFAISVIVPNVIPSVIFLSSTILLIISLWKHARHLQHNGISTTDLNTSVHLSAIKALASFAFLYLFSSVAIHLQAMPIWRSIDHSWTDVLFQTMSAVYPSGHAVVLILVNPKLKQAWVRMMHHLKCHVSEAPS

>Japanese_Gecko_Tas2r28

MIVILVGTILVLARTWMDVFILTVNVRDWMEKKRLSTADRILTLQGCIRIYLGFPDIIWQILEKFCPWVTWIHYVFKALIFSFWYLLSCNVWLTTSLCSYYCVKIADFSHPFFVHLKLRLSGLVSTWLLGSAILSLAGTLPLAYTTMEIRDNRNFSNFFQNKNETDIRHTGMASRMYVLLGLGPFAAFSICAISAVLLLFSLWRHRRRMLGSWGGHRSPQANAHFQAVITILSLLVNNVVVFIAILLLLSRGFKRNLISQLGPSILLYCCFSVESLVSVWGNKKLKNELRRFLYFAKCRGCVSELKRIAHC

>Japanese_Gecko_Tas2r29

MATLLLLTGFALLIMETLVGMVANGFIVLISCIDWIRSRKLSPTELILTWLGLARLAWQAVVILDVIMLFFFLHTYLLDHVFLMINMGWYFIYNINLWFAACLSVLYFVKITTFSHPLFLRVKQRFSGLLPWLLLGSLAFSAFMTMTITVITVSSSGLTSCDLYKLFLNHSFDSKIKKPHTCMDFAISVIVLNVIPSVIFLSSTILLIISLWKHTRLLQHNGISNKDLNTRVHVTAIKALISFAILYLSSTVAIILQAMPVIIDRSWTSMFFQTMSCVYPSVHAVVLILINPKLKQAWVKMIHHLKCHVSEAPS

>Japanese_Gecko_Tas2r3

MATLLSITGFALLIMETLVGIIANGFIVLISCIEWIRSRKLSQTDLILICLASSRLLWQALVMLRVTMLFFPPLIYRLKLVHLTVTIMWFFTDTINLCFAACLGVWYFTKIAIFSHPLFLHVKQRISGLLPWLLLGSVVYSVFMTVTVFTVLNSDTTICDPYKSFLNNTYDSEIQTPHSCIDLLFFRMVPTVIPSVIFLSSAILLIISLWKHTRHLQQNGIGVRDLSTNVHLSAIKALASFAILYLSSLVATNLVLVRRSNDGSWRSVLYNVSAAYPSGHAVVLILINPKLKQAWVRMIHHLKCCVSEAPS

>Japanese_Gecko_Tas2r30

MEVSAFVIISSILLVIETLVGLVANGFIALMNYIDWFRSRKLSPNDQILTCLALSRLMWLAAVILNITAHYYSMDKHNCHYVYLMLPILWIFTNTSSTLFATCLSVFYLTKIATFSHPVFLQVKLRFSGLVPWLLPSSVLLSAFTAIFLVTGLSNGFSMCDSNKSLLNITDSGIKLPDLYMYIDILATVPNLIPLMIFLSSSILLLTSLWMHRRRMQRNGTGIQDLNTQVHLTAIKALASFAVLYLSSFLAVIAQAVLIWNDMSGTWLFMLLSNVTVSSPSGHAVILILLNPTLKQAWIRMLLHLKCCSSEVPS

>Japanese_Gecko_Tas2r31

MSEFDALNVLCLVIVAIVIVVGVMGNGFIVLANGLDWIRSKTMPPSDMILTALSLSRLLFLGLVLAVHCLFFLDVDNPKSLPESLIFFWGFANATTLWMATCLAVFYCMKLVNLPQVFFVKMKLGLSRLVPRLLLGSVLVSFITSFPIIFFEKCSPCCNETRVVRGNRNTTCPQKVLSGIIYITGSSPSFVIVLASSVLLIRSLLHHARKMRLNMGGVKDHRMDVHIKAVKTLVSFVILFTASFVAVVSLAMFTSPWTIVTSTVVIIVCNSGHSVMLISMNPKLKQPLIRSLRGSLGRTLRRISTSCPKTAVGNCSC

>Japanese_Gecko_Tas2r32

MSLNAFIVTVSCINGMKSKQLKSIDKILAALGITRFCYLGMFLGKIFWMSISSRVFEVTALYQMFKAAIWFLTCVCFCFSACLCSFYCIKIANFGHRLFVYLKLRISRLVPWMLLVSVLGSLLNSFPFFNGIYNIACKNSTGSGESANRTLEDFTWETNLLSLFAYCGVGFSVVFSISVASSCLLLFSLWRHAHLMQNGSPSFSKLSMAAHFQAVKTIMSLLIVDSVNFIGLMILLSNVFSERGPTNRLITIIVYVCPSAQSQIVIWGNPKLKRAFIRLTNCIRHMSLV

>Japanese_Gecko_Tas2r33

MLVIVGTETVLGAWINAFIMTVCCINRLKNKSLSAADHILMVLATNRFCFLILGMLRVLCRTLSPLIYYKDFVYRGLKAILWFFISSNLWLAACLCLFYCVKIASFSHPLFISLKLKITRAVPALLLGSELLSLVNTIPFFSLIYTVQCNVSNDTASGNIKSQIDMHTNWRNLFFLCGFGFSLVFAIFVTSAALLLVSLWRHARQLRGVLSGYTSPRMAAHVRAVRVITYFLVTYLVNFVALMLLLTDVFSEDSALDFLCTIVLNACPSVHSITLILTNPKFKKTFLQMLRHGGCKW

>Japanese_Gecko_Tas2r34

MSSLLSIGLALLVLETLIGMVANGFIVLFICTDWFRSRKVSPTDLILCCLGLLRFIWQVTVFLIVIMTSFFKCTFVQVNVWLVFEIMWVFMNTVHLWFAAWLSVLYFVKITMFSHPVFLQVKQRFSGLVPWLLLGSVVFSAAVTILITASTYIFPICHHYKSLSRNSSDSECKAPHSCSDFAMLSIAPSLIPFLLFLSSSILLIISLSKHLRHLQHNGVGVMDLNTQAHLSAIKALASFAVLYLSSFVAANSLIIMPWIAYERHWTSTLVENASAIYPAGHAVILILMNPKLKQAVIQMLHHLRCRL

>Japanese_Gecko_Tas2r35

MAMIDRHLFSPLGVFFLVVFGIESIVSLLGNGFILVVNGHSWLHNKKMLPSDFLLTTLSLSRFLSQWVSMTAQFMYFSSPDTYIYSKEQPALKFSWLYLNTASLWCATWLNVFYCVKVTNFAHPLFSWLKLRIGVLVPRFLGISLLAFILCSIHPVMMYFEDEKCHYLTGNLPENTSKSEAHGSECVMFLSPLLISSTAISFSICLTASVVLLLSLWRHKRNLKKGGLSPKDFSTQAHLRVINPLLLSLFFYVLHFAAIILIFTGVFKYGKLQRLICEIFLFLYPSAHSIILIFTNPKLRKLCTRVLNL

>Japanese_Gecko_Tas2r36

MAAMDRNMFSPLGIFFLIIFGIESLVSLLGNGFIVVVNGQSWLHSRKMLPCDFLLTTLSLSRFLLQWVIVSSQFVYFSYLETYIDSRKHQAFSLTWMYLNTASLWCATWLSVFYCVKVTNFAHPLFVWLKPRIAVLVPRFLGISLLAFIICSVHPVMRSLENEKCCNLTGNLVGNTSQSKTCGSHFLMLLSPLQFSFTAISFSICVSASILLLLSLWRHTRNLKMGGLSTKDFSTQAHLSVMKPLLLLLFFYIVHFVAMIITMGDILHYGKLERLISDIVLTSYPSAHSVILIFTNPKLKKTCIHMLNLRRGAS

>Japanese_Gecko_Tas2r37

MVQSSMGHPQGQKKDSTAMESAMPRDRALVPSSLVTFYLAVTGTAYLLVIMTNGFIIVVNLSDWSKGRGLMPNDKILSSLALSNLCYSTSFITDYFFSLIWDEFYSVFYNLQRVFMTLDIATSFSSFWFTAWLSVFYCVKIVSFKRLLLLKLKLQFPGLVRWLLLGSALVSLGAALLFQLAFMVVSHRKPATNQTTSLSPASNCTRNHVNAGIILVHMSPVYRVLIIIIGCSIPLMVVVFSSVPVLWSLFRHTQKLEQTLSPSHLEAHVKAAKAVLTLLLCYIISFVCQTLVRAEIYRNWYYPYFLCLMVQLATLLAQSTILIRSNSRLMQTATHLLPCPLGRQRKGNSRKPTELQEV

>Japanese_Gecko_Tas2r38

MATLLSITGFAILVMETLIGMAANGFIVLVSYIEWIRSRKLSPTDRILIWLGLARLMWQAIVMLHVTMLSFSPHTYILKQIHFGVAMMWFFADTVNVCFAACLGVWYLTKIAVFSNPVFLQVKQRISGLLPWLLLGSVAFSAFMTMTMTVFAVSSSDITIRDPYKLLLNNSFGSEIQKPHSCMDLVFLRIVPTVIPSVIFLSSTILLSVSLWKHARHLQQNGIGGRDLNTNVHLSAIKALASFAILYLSSLVATNLQTVLVCRSNDGSWASVFFHNVSAAYPSGHAVVLILINPKLKQAWVRMIHHLKCLVSEATS

>Japanese_Gecko_Tas2r39

MVMMDRDLFSPLEVFFLIIFAIESMVSLLGNGFILAVCGHSWLRNKKMLPCDFLLTTLSLSRFLSQWVSTSSQFVHFSSPEMYIYSTAEQASAISWNYFNTASLWCATWLNVFYCVKVTNFPHPLFAWLKLRIGALVPRFLGISLLAFIILSIHPLMRYFEDEKCHNLTGYLPENTSQKEAHDINCVRFLIALQISSIGISFSICLTASVVLLLSLWRHKQNLKKGGLSTKDFSTQAHLSIMKPLLLSLFFYILHFAATILALTNSFKYGKIEQLICEIFQFSYPSAHSVILIVTNPKLRKACTHVLNLRRHTS

>Japanese_Gecko_Tas2r4

MEVSVFMIISYILLVIETLVGLVANGFIVLMNYFDWSRSRKLSPYDQILTCLGFSRLAWLAVVILGTTMDCFSMGQHTCHYAYLMPPILWMFTNSATIWFATWLSVFYLAKIATFSHPVFLQVKLRFSGLVPRLLLGSVVFSAIMAITSLSNGFSMCDFDKSLLNISDSEINFTDSYAYIDFLAAAPTLIPLIIFLPSSILLMASLWMHRRRMQCNGIGIQDLSTQAHLTAIKALASFALLYLSCFSALIAQAVLISNNMSDTWLFMLLNNVAVSSPSGHAVILILINPKLKQRWVRMLLHLKRCSSEVPS

>Japanese_Gecko_Tas2r40

MMVMMERDLFPPLEVFFLVVFGIESIVSLLGNGFILAVNSHSWLRSKKMLPCDFLLTTLSLSRFLLQWVSTSSQFVYFSSPETYIHSEKRQAFLLPWVYLNTASLWCATWLNVFYCVKVTNFPHPLFSWLKLRIGALVPRFLGISLLAFIICSIPPVLRSFENEKCCNLTGYLPENTSQKEAHDSLRFLIMLQISSTAISFSICLTASVVLLLSLWRHKRNLKKGGFSTKDLSTQTHLSVMKPLLLSLFFYILHFAATILAFNDILRYGKLQHLISDIFLSSYPSAHSVILIFTNPKLRKVCTYVLNLRRSTS

>Japanese_Gecko_Tas2r41

MVVMDRNLFSPLGVFFLIVFGIESMVSLLGNGFILAVCGHSCLCSKKMLPCDFLLTTLSLSRFLFQLVSMSSQFWFFSSPETYIDSETEHAFALFWHYLNIAILWCATWLNVFYCVKVTNFPHPLFAWLKLRIGALVPRFLGISLLAFIICSIPPVLRSFENEKCCNLTGNLPENTSQSEAHPRYSRMFLKTIQIYFTAMNFSICFTASVVLLLSLWRHTRNLKKGGLSTKDLSTQAHLRVMNPLLLSLFFYILHFAAMILGLANVFEFGKLERLISDIFLSSYPTAHSVILIFINPKLRKACTHVLNLRRSAS

>Japanese_Gecko_Tas2r42

MGSNSDCGSTTGDLFDILFGTILLFGRTCVDVFILTVNGRDWMEEKCLSTTDRILTLQGSIRICLGYMNLIWHILEKFCPWITRIGYVFKAFMFIFWYLMSCNIWLTTSLCTYYCVKIADFSHPFFVHLKLRLSGLGSTWLLGSAILSLASTLPFAYIDLEIRDNSNSSNFSQNKNETDSHTGMAFRMYVLLGLGTFAAFSICAISAVLLLFSLWRHRQRMLGSWGGHRSPQTDAHFQAVITIFSLLVNNVAVFIAILVFLSRVFEGNSISQLGPSVVLYCCFLVESLVSVWGNKKLKNELRRSLHFAKCRGCVS

>Japanese_Gecko_Tas2r43

MATLLSITGFALLLMETLVGMVANGFIVLISCTEWIRSRKLSPTDLILTCLGLARFAWQAIVILDATMYSFFLRIYLLNYVRLVLTIVWLFTYNVSLWFAAYLSILYFVKITTFSHPLFLRVKQRFAGMLPWLLLSSVAFSAFMTMTIAVTTVFSSDLTICDPYQLFLNNSYNSGMQRPYLCRKLVIAVTAPTFILSVIFLSSTILLIISLWKHTRHLQHNGISTNDLNTGVHLSAIKVLASFVVLYLFSLVAINLQVLLVWRRINRPWTDVLFQTMSAVYPSGHAVALILMNPKLKQAWVRMIHHLKCHVSEAPS

>Japanese_Gecko_Tas2r44

MASLFFILALTLLVMETLVGVVANGFILLINGIDWFRSRKLSPTDLTLCCLGLSRLAWQVVGFLDAIMFFFFLSTYLSNSIQLMFLVLSIFTHAADIWFATWLGVLYFVKITMFSHPVFLRVKQRFSGLVPWLLLGSVVFSAVLTMSIITALNYDASICNPYKSRLSNSNDSGIKMPHFCRNVTILATAPHFVPIVIFLSSTTLLIASLWKHTRRVQHNGTGTKDLSTQAHLTAIKALASFLILYLSSFVAVTLQSLVTWRNDNGSWISVLFHNVIAAYPSGHAVILILINPRLKQAWVGMLHHLKCRLREVPS

>Japanese_Gecko_Tas2r45

MEVSVFMVISSILLVIETLVGMVANGFIVLMNYIDWFRRRKLSPNDQILTCLGLSRFSWLAVVFLDITKGFFSKDKHNCHYVYLMLHIGLIFAKTATIWFATWLSVFYLAKIATFSHAVFLQVKLRFSGLVPWLLLGSVVFSAIMAISLVTSLSNGFSMCDSNKSLLNVSDSGIKLPDLYMYIDFLATAPNLIPLMIFLASSILLMTSLWMHKRRMQSYGSGIQDLNTQVHLTAIKALVPFTVLYLSCFLAVIAQGVLIWNDMSDTWLFMLLSNVAVFSPSGHAVILILINPKLKQAWVRMLLHLKCCSSEVPS

>Japanese_Gecko_Tas2r46

MYNFLRRITFTLLVIETLVGMAANGFIVLINCIDWFRTRKLSPNDLILSCLGLSRLAWLVVMTLDRTKVFFSLGNHIWDTHVMPPIVWIFTNSANIWFATWLSVFYLAKIAIFSHPIFLQVKQRISALVPWLLLGSVIFSAMTAVILMTSLNNGFAMCNPSNDSEIKEPDSWKYLDILGMAPNLIPFLIFLSSTILLISSLWKHMRRVQSNGTGTGDLNTQAHLSAIKALASFAVLYLSSFLAITSQAVLILNNMDHSLPVRLLDLVVAAYPSAHTIILILINPKLKQAWVRMLHPLKCCLREVPT

>Japanese_Gecko_Tas2r47

MATIFMRITFTLRVTEILVGMVANGFIVLINCIDWFRSRKLSSNDLILTCLGLSRLAWLVIMILEGMENFFSLGNRVWNDAHPMPPIMWIFTNSANIWFATWLSVFYLAKIAIFSHPIFLQVKQRISALVPWLLLGSVVFSAMTAVILMTSLNNGFSIRSPANDSESKKPGCSNYVGILTVATNLIPFLIFLSSTILLITSLWKHIRHVQCNGIGVRDLNIQVHLTAIKALASFAVLYLSSFLAIISQEVLIKNNMDHSWSVRLLDLVVAAYPAGHTIILILINPKLKQTWVRMLHHVKCCEGPS

>Japanese_Gecko_Tas2r48

MATLLLITGFALLIMETLIGMVANGFIVLFSCIEWTRSRKLSSTDLILTCLALARLAWQAIVILDVTMYSFFLRTYLLNHVFLMISMVWFFTYNTNLSFAACLSILYFVKITTFSHPLFLQVKQRFSGLLPWLLLGSLAFSAFMTMTITVITVSSRGLTFCDPYKLLFNNTFDLEIKNDRPCTDFAISVVVPNVIPSVIFLSSTILLIISLWKHTRHMQHNGISTTDLNTSVHLTAIKTLTSFAILYLSSSVAINLQILPAWRIIDRSSTNVLLEAMSAVYPSGHAVVLILINPKLKQAWVGMMHHLKCHESEAPS

>Japanese_Gecko_Tas2r49

MATLLSITGFALLIMEILIGMVANGFIVLISCTEWIRSRKLSLTDLILTCLGLSRLMWQATVTLQVTMLSFFLRTYLLTHVYLVINMVWFFTYIINLSFAAWLSIMYFVKITTFSHPIFLQVKQRFSGLLPRLLLSSVAVSAFMTVTVAVITVSSGGLTICDPYKLFLNNSYNSDIKRPHLCRQLFITVIAPNFILSIIFLSSTILLIISLWKHTRHLQHNGIITKDLNASVHLTAMKSLASSAILYLFSFVAINLQSILVLRSINHPWTDVLFQTMSAVYPSGHAVVLILINPKLKKAWVGMMHHLKCHESEAPS

>Japanese_Gecko_Tas2r5

MEVLAFMIIGSILVIEALVGLVANGFIVLMNYIVWFRSRKLSSNDQIMTCLALSRFSWLVVVILNIMKHFFSMGQHTCIYAYLMLPILWIFTNTASIWFATWLSVFYLVKIATFSHPVFLQVKLRFSGLVPRLLLGSVVFSAIMAITSLSSSFSMCDSDKSLLNITDSGIKLSDLNTDFLATAPTLIPLMIFSSSTILLLASLWMHRRRMQRNGTGIQDLNTQVHLTAIKALASFALLGLSSCLVVTAQAVLIWNNMSDTWLFMLLSNVTVSSPSVHTVILILLNPKLKQAWVRMLLHLKHCSSEVPS

>Japanese_Gecko_Tas2r50

MMVAMDRYLLSPLGVFFLIIFAIESIVSLLGNGFILAVSVHSWLHSKKMLPCDFLLTTLSLSRFLLQWVSLSSRSMYFRSLLETYIYSKEQQAFGFLWVYLNTASLWCATWLNVFYCVKVTNFAHPLFSWLKLRIGVLVPRFLGISLLSFIICSIHPVVGYFEDEKCHNLTGYLPENISQREADDTNCAMLLNTLQMSFTAISFSICLTASLVLLLSLWRHTRNLKKGGLSTKDLSTQAHLRVMKPLLLSLFFYILHFAAMILALTNIFRYGKLEQLICEIFMALYPSAHSVILIVTNPKLRKVCSHVLNLRRSAS

>Japanese_Gecko_Tas2r6

MLCCVLLMGSVSIAAEERLLFSFVLRKMATLLSITGFVLLIMETLVGLVANGFIVFINCIDWYRSRKLSPTDLILICLASSRLMWQALVMLHVTMLSFFLHTYVLKRVHLIVMIMWFFTDTVNLWFAACLGVWYLTKIAIFSHPIFLQVKQRFAGLLPWLLFGSVVFSSFMTIINFTESFSGLATCDPYKLVLSNSSDSDIQKPHSCMGLVFLRIVSNFVPSVIFLSSTVLLIISLWKHTRHLQHNGTGVKDINTRVHLTAIKALASFAILYLFSLVAINLQSMLVWGSNDLSWTSVLFHNVSDVYPSGHAVILILINPKLKQAWIRMIHHLKCHVSEASS

>Japanese_Gecko_Tas2r7

MMVAMDRDQLSPLGVFFLIVFGIESIVSLLGNGFILAVNGHSCLCSKKMLPCDFLLTTLSLSRFLLQWVSMSSRYMYFSSPETFIYSKKHQALRFPWVYLNTASLWCATWLNVFYCVKVTNFAHPLFIWLKLRIGVLVPRFLGITLLAFIICSIHPVVGYFEDEKCHNLTGYLPENTSQRAAHDIKCFMLLNTLQMSSTGISFSICVTASIVLLLSLWRHTRNLKKGGLSTKDFSTQAHLRVMKPLLLSLFIYILYFAAMILIHTNMFRYGKLEQLICEIFMSLYPSAHSIILIFTNPKLRNVCTHVLKLRRSAS

>Japanese_Gecko_Tas2r8

MFSLRNITQEPGEPQFNSSCSHFRYFSPPMVVMDRDLFSPLGVFFLIVFGIESIVSLLGNGFILAVNGHSWLCSKKVLLCDFLLITLSLSRFLWQSVITSSQFLYFRSPETYIYSKEEQAFGYLWVYLNTASLWCATWLNVFYCVKVTNFTHPLFSWLKPRIGVLVPRFLGISLLTFIISSILPVLRSFEDEKCHNLTGNLPENTSQSEVHGTNCVMFLNTLHIYFTAISFSICLTASLVLLLSLWRHKRNLKKSGLNTKDFSTQAHLSVMKPLLLSLFFYILHFATMILVFNNIFRYGKLEQLIGEIFLCSYPSAHSVILIFTNPKLRKASTHVLNLRRSAP

>Japanese_Gecko_Tas2r9

MFSWQNIVQELEEPQFNSTCSHFLIFPMMLEMDRDLFSSLGVFLLGIFGIESIVSLLGNGFILAVNGHSWLRSKKMPPCDFLLTTLSLSRFLSQWVSLSSRCMYFSYPETYLYSKEEQAFNFSWFYLNTASLWCATWLNVFYCVKVTTFTHPLFSWLKLRIGVMVLRFLGISLLLFIICSIHPVVEYFEDEKCHNLTENLRENSSQREARGTNSLRFLNTLEIFFTAISFSICLTASVVLLLSLWRHMRNLKKSGLSTKDFSTQAHLRVMKPLLLSLFFYILHFAAMILLLTKTFRYGQLEQLICEIFVYSYPSAHSVILIFTNPKLRKVCSHVLNLRRSAS

>Speckled_Rattlesnake__Tas2r1

MSVSGVHNWLCLIIITAVTLVGMTGNGFIFLSDCHDWIRSKAPSGPGLLLMTLSLTRFIFLGIMLGFHCFSFLDINRPKYAGSVISFFWTFFNATTLWITTCLGVFYCVKIVNFSQPFLVKMKLRISSMVPHLLVAVVLVSLISALPFLWIDDHNQSDNAEGVRELRVQTFLFSILYTLGTFPSFVIFLISSGFLIYSLVHHMKRMQNSSVGFRDQRMDVHLKTTKILTSFLILYAATFAAEISMIFSPSPWTTVISNIVVSSYNSGHTVALIVMNSKLRGRLSKMFWCFRKQT

>Spiny_Softshell_Turtle_Tas2r1

MFSIIIYFIILGMELIVGMIANGLMAVVNCLEWIRSRNVTCCDMILTSLGISRFFFQCMIIINSAIYNISLEDNAHLALMRTLAFISSFLNTLSLWLATWLSVFYCAKIANFSQPLFFWLKWRILGLMPQLLTGTFLVSFVTSLPSINSVNRKYVNNSVNNLLGNTTEEWTYYTNFFSGLSILYMLGHSFPFVIFIVSSALLLTSLWRHTKRMAKITSSCRDTVTQAHVKAIQGQLSFIFFHSSYFVAQVILFSGFFTNSISNSLWCIVIMAAYPSGHSVILVLGNPKLKKVAVRALHYARCRLRDEVS

>Spiny_Softshell_Turtle_Tas2r2

MLTPVALIFLILLGLESLVSNFGNGFIIVVIFSNWIKSRKLASCEHILICLSISRFLLQWLVMLSNFIYISFPKTSALGCKHKAFGILWAYLNLVSLWCATCLSFFYSVKIANFTQPLFRWLKLRIAWLVPRLLLGSLIISLVSTIPLVWSDVGFDLCNSTKSLERNTTWNDAKDIPYIIFVPVQILVLIIPFIIFFVSSTLLLISLWKHTKKMKNNVTRFKDLSVEAHIGAMKSLLSFFILYIIYFVTVIVILTSSITSQNSVHLPYEVLLSAYPSGHPIVLILTNPKLKQVAVKILHQIKCQLREGTL

>Spiny_Softshell_Turtle_Tas2r3

MMKNSLALSDIFYLIITAVELSAGVVANGFIVGLNCIDWAKSRTMTSYDMIITSLAFSRFCLQFLVSSDNFLHILYPDVCDMVEIIMMMLVIWMFINHVSLCFASCLSVFYCVKIATFNQSFFTWLKLKLSRLVPWLLLGSLLYCLVTTVTFTFFSYFFMITSHICPYRPSRNITIPEKEKNLTTFVFLIHGVGSIFPLILFIASSLLLIISLCRHIRKMDLNSDLNPSFRNSRTDAHVSALKSVLSFFIIYNIFYVASTLSIGTGSYFSAQWKIMLCTLVVAAYPSVHSFVLILVNPKLKLASARILHSANCCFREVTS

>Spiny_Softshell_Turtle_Tas2r4

MEDIDYNIEQEDDITIPRIIMLVILVAEAFVGMWINSFIVATNCFQCVKHRGLSSSDNILTVVAFSRFCILLETTLQTFCSTFYPEIYYMDSVFQAFRAVTWFLNSSNQWFAACLGVFYCVKIANFSHPLFISLKFKISRLVPWLLLVSVLFSLFSSLPFLNTLYKIRYNDFNSSLKRTYPMKNVTVETSVSHVLFICGTGFSTAFTIFIISAFLLWFSLWRHTRRMQNNSRCFRSPCVEAHIQAMKAIMSFLLINVVNFIALLILLTNTLEETSVMGIACTIIIDACPSVHSIVLVLSNSKLKTTLIKVLHYAKCKG

>Spiny_Softshell_Turtle_Tas2r5

MLSAIIIALIVLGIELIIGSIANGPMIVVNCLEWIRSRKLTRCDMILTSLGISRFLLQCMIFVNSIVLQLLQDINRSCNTSSHFFVVWMYLSTLSLWFATWLSVFHCVKIATFSQPLFLWLRQKIPGLLPQLLLSSLLISLLTCFPSVNTVYRNSSMNNLSGNTTVECKCVIDLFSGLSMFSIVGFYSPFIIFIVSSALLITSLWKHSKRMRKAMSSSKDTITEAHVRAIKGLISFIFFLQFIFCSTSHIFDRIIQQRPLFLVVVGSDNGCLSLWALCYPCAGESQTEKGSSEGFALCPVQAER

>Timber_Rattlesnake_Tas2r1

MSVSGVHNWLCLIIITAVTLVGMTGNGFIFLSDCHEWIRSKAPSGPGLLLMTLSLTRFIFLGIMLGFHCFSFLDINRPKYAGSVISFLWTFFNATTLWITTCLGVFYCVKIVNFSQPFLVKMKLRISSMVPHLLVAVVLVSLISALPFLWIDDHSQSDNAEGVRELRVQTFLFSILYILGTFPSFVIFLISSGFLIYSLVHHMKRMQNSSVGFRDQRMDVHLKTTKILTSFLILYAATFAAEISMIFSPSPWTTVISNIVVSSYNSGHTVALIVMNSKLRGRLSKMFWCFRKQT
