# Supplementary material for: Genomic evidence of bitter taste in snakes and phylogenetic analysis of bitter taste receptor genes in reptiles
Source: PeerJ. 2017 Aug 18;5:e3708. doi: 10.7717/peerj.3708 (PMC5564386; doi:10.7717/peerj.3708)
Supplement: Data S3 [file peerj-05-3708-s003.docx]

>mouse Calhm1

ATGGATAAGTTTCGGATGATCTTCCAGTTCTTGCAATCCAACCAAGAGTCCTTCATGAATGGCATCTGTGGCATCATGGCGCTGGCCAGTGCGCAGATGTATTCTGCCTTTGACTTCAACTGCCCCTGCTTACCCGGCTACAACGTGGTCTACAGCCTGGGCATACTGCTGACGCCTCCCCTGGTGCTCTTCCTGCTTGGTCTGGTCATGAACAACAACATATCCATGCTAGCTGAAGAGTGGAAGCGCCCCGCAGGTCGCCGGGCCAAGGACCCAGCTGTTCTACGCTACATGTTCTGTTCCATGGCCCAGAGAGCTCTCATCGCCCCTGTCGTCTGGGTGGCTGTCACACTGCTGGATGGCAAGTGCTTTCTCTGTGCCTTCTGCACAGCTGTGCCCGTGGCCACACTAGGCAATGGCAGCCTGGTGCCGGGCCTGCCTGCTCCAGAACTTGCTCGCCTACTGGCTCGGGTACCCTGCCCTGAGATCTATGATGGGAACTGGCTGCTAGCCCGAGAGGTGGCCGTGCGGTATTTGCGCTGCATCTCTCAGGCACTGGGTTGGTCCTTCGTGCTGCTGACCACATTACTAGCGTTCGTGGTACGCTCTGTGCGTCCCTGCTTCACGCAGGTCGCCTTTCTCAAGAGCAAGTACTGGTCCCACTACATTGACATTGAGCGCAAGCTCTTCGATGAGACATGCACAGAGCATGCCAAAGCCTTTGCTAAGGTATGTATCCAGCAGTTCTTTGAAGCCATGAACCATGACCTGGAACTGGGTCATACCCACGGAGTACTGGCCACGGCCACAGCCACAGCCACAGCCACAGAGGCTGTCCAAAGTCCCTCGGACAGGACAGAAGAAGAGAGGGAGAAGTTGCGTGGCATCACTGACCAAGGCACCATGAATAGGCTACTCACAAGCTGGCACAAATGCAAACCACCACTGAGGCTGGGCCAGGAGGCACCACTGATGAGCAACGGCTGGGCTGGGGGCGAGCCCCGGCCTCCACGCAAGGAAGTGGCCACCTACTTCAGCAAAGTGTGA

>Anolis_carolinensis

ATGGATAAATTTCGAATGATCTTCCAGTTCCTCCAATCCAACCAGGAATCATTCATGAATGGCATATGTGGCATTATGGCTCTTGCCAGCGCCCAAATGTATGTGGCTTTTGATTTCACCTGTCCTTGCCTACCAGGTTATAATCTAGCCTATGGGATGGGTATCCTGGTTGTGCCACCCCTAGTGTTGTTTTTACTGGGCTTTGTGATGAATAACAATGTCTCCATGTTGGCCGAAGAATGGAGAAGGCCCATAGGGAAGCGGCAGAAAGATCCATCCGTTTTGCGTTACATGTTCTGCTCCATGGCACAGCGGGCTATGATTGCCCCCGCTGTCTGGATTTCAGTCACTCTGTTGCATGGGGAATGCTTTATATGTGCCTTTAGCACCACTGTGCCCATAGAGAAGTTGGGAAACGACAGCTATATGCTCCTGCCTGAGAAAGAGATAAGGAAAATTCTGGCTCGGATACCCTGCAAAGACATTTACAATGGACAAGAACTTATTGCCAAAGAAGTCGCAACCAGGTATCTACGCTGTATCTCCCAGGCAATGGGCTGGTCTTTCGTGTTGCTGATGACCTTGTTGGCGTTCCTTGTCAGATCCTTACGCCCCTGCTTTACACAAGCAGCCTTCCTGAAGAGCAAGTATTGGTCCCACTATATTGACATTGAGCGCAAGCTCTTTGATGAGACCTGCACAGAGCATGCTAAAAGTTTTGCCAAGGTTTGTATCCAGCAGTTCTTTGAGGGCATGAACAAGGACCTGATCATGGGCCATACACACGTTCCTGAGAAGACACCTTCAGAAGCTGACGACGAGAAGGAAAAATTGCGGGGTATCATGGATCAGGGGACTATGAACAAACTTCTGAAGAACTGGCACAAGTGTAAGCCACCTTTGTGCCTCAACCAAGAGGTGGTCCAGAATGGGAATTGCTGGACTGGAGAAATCACACGCCCTCACCTGCCTAGGAGAGAGTATGTCACCTATTATAGCAAAGTCTAG

>Gekko japonicus

ATGGATAAATTCCGAATGATCTTCCAGTTCCTTCAGTCCAACCAGGAGTCATTCATGAATGGCATCTGTGGCATCATGGCTCTTGCCAGTGCACAAATATATGTGGCTTTTGATTTCAAGTGTCCATGTTTACCAAGCTATAATCTGGCCTATGGGATGGGTATCCTGTTTGTACCACCTCTCGTCTTGTTCTTACTGGGCTTCGTAATGAACAACAACGTCTCCATGTTAGCTGAAGAATGGAAAAGACCCACAGGGAAACGGCAGAAAGACCCAGCTGTCTTGCGTTACATGTTCTGTTCCATGGCACAGCGGGCCATGATTGCTCCCGCTGTCTGGGTTTCCGTCACACTGCTTGATGGAGAGTGCTTTGTGTGTGCCTTCTGCACTTCTGTGCCCATAGAGAAGCTGGGGAATGACAGTTACACGGGCCTATCTGAGAAGGCAATGAGGAGGATTCTGGCCCAGATTCCCTGCATAGAGATTTACAGTGGACAGGAACTTATAGCCAGAGAAGTGGCAATCAGGTACCTGCGCTGTATTTCCCAGGCAATAGGCTGGACCTTTGTGCTTCTGATGACCTTGCTGGCATTCCTTGTTCGATCCTTGCGACCTTGCTTCACTCAAGCTGCCTTCCTGAAGAGCAAGTACTGGTCCCACTACATTGACATTGAACGCAAGCTCTTTGATGAGACTTGTACAGAGCATGCAAAAAGCTTTGCCAAAGTTTGCATCCAGCAGTTTTTCGAAGGCATGAACAAGGACCTGAGCATGGGCCACTCACACTTTCCTGAGAAGGCACCTTCAGAAGCTGGGGAAGAGAAGGAAAAACTACTGGGCATTATGGATCAAAGGACTATGAACAAACTTCTGAAGAACTGGCATAAATGTAAGCCCCCGCTATGCCTCAACCAAGAGGTGCTACTGAATGGCTGGGCGGGAGATATCATACACTCTCATGTGCCTAAGAAAGAGTATGCTGCATACTACAGTAAAGTCTGA

>Crotalus horridus

ATGGATAAATTCCGAATGATATTCCAGTTCCTCCAGTCCAACCAGGAGTCATTTATGAATGGCATATGTGGCATTATGGCTCTCCTGAGTGCACAGATTTATGTAGCTTTTGATTTCAAGTGCCCATGTCTACCTAGTTACAATCTGGCCTATGGGATGGGTATCCTGTTTGTGCCTCCCTTAGTCTTGTTCCTGTTTGGTTTTGTGATGAATAACAATATTTCCATGTTGGCTGAAGAATGGAAAAGGCCCATAGGAAAACGACAGAAAGACCCAGCTGTCTTGCGTTACATGTTCTGCTCTATGGCTCAGCGGGCTATAATTGCTCCTGTAATTTGGATTTCAGTCACACTGTTGCATGGGGAATGTTTTATATGTGCCTTCAGTACCTCTGTTCCCATGCACAAACTTGGAAACAGCAGTTATAGACCTCTTCCTGAGAAGGAGATCAGGAAGATTTTAGCCCAAATTCCCTGCAATGATATTTACAGTGGCCAAGAACTGATTGCCAGAGAAGTGGCAACCAGGTATCTACGCTGCATTTCACAGGCAACAGGCTGGGTCTTTGTGCTGCTAGTAACTCTGCTGGCTTTCCTTGTCCGAGCCATACGTCCCTGCTTTACTCAATCTGCCTTTTTGAAGAGCAAATATTGGTCCCACTACATTGACATTGAGCGGAAGCTCTTTGAAGAGACATGTACAAAACATGCTAAGAGTTTTGCCAAGGTTTGCGTTCAGCAATTCTTTGAGAACATGAACAAAGATCCCTTCATGGGCCACACACATATGCACATTCCAGAGAAGGCACCATTAGAGTCAGAGGAAGAAAAAGAACAACTGCTTGGCATCGTAGATCAAGGAACCATGAACAACCTTCTGAAGAACTGGCACAACTGTAAGCCCCCTTTGTGTCTCAATCAAGAGCTGAGGCAAAATGGAACCAGCTGGACAAGAGAAATTGCCCAGCCTTGTCCTTCAAGAAAAGAATATGCAGCCTATTACAGCAAAGTCTGA

>Crotalus mitchellii

ATGGATAAATTCCGAATGATATTCCAGTTCCTCCAGTCCAACCAGGAGTCATTTATGAATGGTATATGTGGCATTATGGCTCTCCTGAGTGCACAGATTTATGTAGCTTTTGATTTCAAGTGCCCATGTCTACCTAGTTACAATCTGGCCTATGGGATGGGTATCCTGTTTGTGCCTCCCTTAGTCTTGTTCCTGTTTGGTTTTGTGATGAATAACAATGTTTCCATGTTGGCTGAAGAATGGAAAAGGCCCATAGGAAAACGACAGAAAGACCCAGCTGTCTTGCGTTACATGTTCTGCTCCATGGCTCAGCGGGCTATAATTGCTCCTGTAATTTGGATTTCAGTCACACTGTTGCATGGGGAATGTTTTATATGTGCCTTCAGTACCTCTGTTCCCATGCACAAACTTGGAAACAGCAGTTATAGACCTCTTCCTGAGAAGGAGATCAGGAAGATTTTAGCCCAGATTCCCTGCAATGATATTTACAGTGGCCAAGAACTGATTGCCAGAGAAGTGGCAACCAGGTATCTACGCTGCATTTCACAGGTACAGGCAACAGGCTGGGTCTTTGTGCTGCTAGTAACTCTGCTGGCTTTCCTTGTCCGAGCCATACGTCCCTGCTTTACTCAATCTGCCTTTTTGAAGAGCAAATATTGGTCCCACTACATTGACATTGAGCGGAAGCTCTTTGAAGAGACATGTACAAAACATGCTAAGAGTTTTGCCAAGGTTTGCGTTCAGCAATTCTTTGAGAACATGAACAAAGATCCCTTCATGGGCCACACACATATGCACATTCCAGAGAAGGCACCATTAGAGTCAGAGGAAGAAAAAGAACAACTGCTCGGCATCGTAGATCAAGGAACCATGAACAACCTTCTGAAGAACTGGCACAACTGTAAGCCCCCTTTGTGTCTCAATCAAGAGCTGAGGCAAAATGGAACCAGCTGGACAAGAGAAATTGCCCAGCCTTGTCCTTCAAGAAAAGAATATGCAGCCTATTACAGCAAAGTTTGA

>Ophiophagus hannah

ATGGATAAATTCCGAATGATATTCCAGTTCCTCCAGTCCAACCAGGAATCATTTATGAACGGCATATGTGGCATTATGGCTCTCCTGAGTGCACAGATCTATGTGGCCTTTGATTTCAAGTGCCCATGTCTACCTGGTTACAATCTGGCTTATGGGATGGGTATCCTGTTTATGCCTCCCTTAGTCTTGTTCCTGTTTGGTTTTGTGATGAATAACAATGTTTCCATGTTGGCTGAAGAATGGAAAAGGCCCATAGGGAAACGACAGAAAGACCCAGCTGTCTTGCGTTACATGTTCTGCTCCATGGCTCAGCGGGCTATAATTGCTCCTGTAATTTGGATCTCAGTCACACTGTTGCATGGGGAATGTTTTATCTGTGCATTCAGTACCTCTGTTCCCATTCACAAACTTGGAAACAGCAGTTATAGACATCTTCCTGAGAAGGAGATCAGGAAGATTTTAGCCCAGATTCCCTGCGATGATATTTACAGTAGCCAAGAACTGATTGCCAGAGAGGTGGCAACCAGGTATCTACGCTGCATTTCACAGGCAACAGGCTGGGTCTTCGTGCTGCTAGTAACTCTGCTGGCTTTCCTTGCCCGAGCCATACGTCCCTGCTTTACTCAATCTGCCTTTTTGAAGAGCAAATATTGGTCCCATTACATTGACATTGAGCGGAAGCTCTTTGAAGAGACATGTACAAAACATGCTAAGAGTTTTGCCAAGGTCTGTGTCCAGCAATTCTTTGAGAACATGAACAACGATCCCTTCATGGGCCACACACATATGCACATTCCTGAGAAGGCACCCTCAGATTCAGACGAAGAAAAGGAGCAACTGCTTGGCATCGTAGATCAAGGAACCATGAACAAGCTCCTGAAGAACTGGCACAACTGTAAGCCCCCTTTGTGTCTCAATCCAGAGGCAATGCAAAATGGAACCAGTTGGACAAGAGAAATTGCACAGCCTTGTCCATCAAGGAAAGAGTATGTAGCCTATTACAGCAAAGTGTGA

>Pantherophis guttatus

ATGGATAAATTCCGAATGATATTCCAGTTCCTCCAGTCCAACCAGGAGTCATTTATGAATGGCATATGTGGCATTATGGCTCTCCTGAGTGCACAGATCTATGTGGCTTTTGATTTCAAGTGCCCATGTCTACCTGGTTACAATCTGGCTTATGGGATGGGTATCCTGTTTATGCCTCCCTTAGTCTTGTTCCTGTTTGGTTTTGTGATGAATAATAATGTTTCCATGTTGGCTGAAGAATGGAAAAGGCCCATAGGGAAACGACAGAAAGACCCAGCTGTCTTGCGTTACATGTTCTGCTCCATGGCTCAGCGAGCTATAATTGCTCCTGTAATTTGGATCTCAGTCACACTGTTGCATGGGGAATGTTTTATCTGTGCATTCAGTACTTCTGTTCCCATACACAAACTTGGAAACAGCAGTTATAGACATCTTCCTGAGAAGGAGATCAGGAAGATTTTAGCCCAGATTCCCTGCGATGATATTTACAGTGGCCAAGAACTGATTGCCAGAGAGGTGGCAACCAGGTATCTACGCTGCATTTCACAGGCAACAGGCTGGGTCTTTGTGCTGCTAGTAACTCTGCTGGCTTTCCTTGCCCGAGCCATACGTCCCTGCTTTACTCAATCTGCCTTTTTGAAGAGCAAATATTGGTCCCACTACATTGACATTGAGCGGAAACTCTTTGAAGAGACATGCACAAAACATGCTAAGAGTTTTGCCAAGGTTTGCGTCCAGCAATTCTTTGAGAACATGAACAAAGACCCCTTCATGGGCCACACACATATGCAGATTCCTGAGAAGGCACCATCAGACTCAGAGGAAGAAAAGGAACAACTGCTGGGCATCGTGGATCAAGGAACCATGAACGAGCTTCTGAAGAACTGGCATAACTGTAAGCCCCCTTTGTGTCTCAATCAAGAGCTGATACAAAATGGGACCAGTTGGACAAGAGAGATTCCACAGCCTTGTCCACCAAGGAAAGAGTATGCAGCCTATTACAGCAAAGTTTAA

>Python bivittatus

ATGGATAAATTCCGAATGATATTCCAGTTCCTTCAATCCAACCAGGAATCATTTATGAATGGCATATGTGGCATTATGGCTCTCTTGAGTGCACAGATCTATGTGGCTTTTGATTTCAAGTGTCCATGTCTACCAGGTTACAATCTGGCCTATGGGATGGGTATCCTGTTTGTGCCCCCCTTAGTCTTTTTCCTGTTTGGTTTTGTGATGAATAACAATGTTTCCATGTTGGCTGAAGAATGGAAACGGCCCATAGGGAAGCGACAGAAAGACCCAGCTGTCTTGCGTTACATGTTCTGCTCCATGGCTCAGCGGGCTATGATTGCTCCTGCAATTTGGATCTCAGTCACACTGTTGCATGGGGAATGTTTTATATGCGGATTCAGTACCTCTGTTCCCATGCAGAAACTTGGAAACAACAGTTATAGACAACTCCCTGAGAAGGAGATCAGGAAGATTTTAGCCCAGATTCCCTGCAGTGAAATTTACAGTGGCCAAGGACTTATTGCCAGAGAAGTGGCAATCAGGTATCTGCGCTGCATTTCACAGGCAATGGGCTGGTCCTTTGTGCTGCTAGTAACTCTGCTAGCTTTCCTTATCCGATCCATACGTCCCTGCTTTACTCAGTCTGCCTTCTTGAAGAGCAAGTATTGGTCCCACTACATTGACATTGAGCGGAAGATCTTTGATGAGACCTGTACAAAACACGCTAAGAGCTTTGCCAAGGTTTGCATCCAGCAATTCTTTGAGAACATGAACAAAGACCTCTTCGTGGGCCACACACACATGCACATTTCTGAGAAGGCACCATCAGACTCAGAGGAAGAAAAGGAACAATTGCTGGGCATCATAGATCAAGGAACCATGAACAAGCTTCTAAAGAATTGGCACAAGTGTAAGCCCCCTTTGTACCTCAATCAAGAGCTGATGCAAAATGGGACCAGCTGGGCAAGAGAAATTGCACAGCCGTATCCACCAAAGAAAGAGTTTGCAGCCTATTACAGTAAAGTTTGA

>Thamnophis sirtalis

ATGGATAAATTCCGAATGATATTCCAGTTCCTCCAGTCCAACCAGGAATCCTTTATGAATGGCATATGTGGCATTATGGCTCTCCTGAGTGCACAGATCTATGTGGCTTTTGATTTCAAGTGCCCATGTCTACCTGGTTACAATATGGCTTATGGGATGGGTATCCTGTTTATGCCTCCCTTAGTTTTGTTCTTGTTTGGTTTTGTGATGAATAACAATGTTTCCATGTTGGCTGAAGAATGGAAAAGACCCATAGGGAAACGGCAGAAAGACCCAGCTGTCTTGCGTTACATGTTCTGCTCCATGGCTCAGCGGGCTATAATTGCTCCTGTAATTTGGATCTCAGTCACACTGTTGCATGGGGAATGTTTTATCTGTGCATTCAGTACCTCCGTTCCCATGCACAAACTTGGAAACAGCAGTTATAGACACCTTCCTGAGAAGGAGATCAGGAAGATTTTAGCCCAGATACCCTGCAATGATATTTACACTGGTCAAGAACTGATTGCCAGAGAAGTGGCAATCAGGTATCTACGTTGCATTTCACAGGCAACAGGCTGGGTCTTTGTGCTGCTAGTAACTCTGCTGGCTTTCCTTGCCCGAGCCATACGTCCCTGCTTTACTCAATCTGCCTTTTTGAAGAGCAAATATTGGTCCCACTACATTGACATTGAGCAGAAACTCTTTGAAGAGACGTGTACAAAACATGCCAAGAGTTTTGCCAAAGTTTGTGTCCAGCAATTCTTTGAGAACATGAACAAAGACCCCTTCGTGGGCCACACACATATGCACATTCCTGAGAAGGCACCATCAGACTCAGAGGAAGAAAAAGAACAACTGCTGGGCATCGTAGATCAAGGAACCATGAACAAGCTCCTGAAGAACTGGCACAACTGTAAGCCCCCTTTGTGTCTCAATCAAGAGCTGATGCAAAATGGAACCAGTTGGACAAGAGAAATTGCACAGCCTTGCCCCTCAAAGAAAGAGTATGCAGTCTATTACAGCAAAGTTTGA

>VIPERA

ATGGATAAATTTCGAATGATATTCCAGTTCCTCCAGTCCAACCAGGAATCATTTATGAATGGCATATGTGGCATTATGGCTCTCCTGAGTGCACAGATCTACGTGGCTTTTGATTTCAAGTGCCCATGTCTACCTAGTTACAATCTGGCCTATGGGATGGGTATCCTGTTTGTGCCTCCCTTAGTCTTGTTCCTGTTTGGTTTTGTGATGAATAACAATGTTTCAATGTTGGCTGAAGAATGGAAAAGGCCCATAGGGAAACGACAGAAAGACCCAGCTGTTTTGCGTTACATGTTCTGCTCCATGGCTCAGCGGGCTATAATTGCTCCTGTAATTTGGATTTCAGTCACACTGTTGCACGGGGAATGTTTTATATGTGCCTTCAGTACCTCTGTTCCCATGCACAAACTTGGAAACAGCAGTTATAGACCTCTTCCTGAGAAGGAGATCAGGAAGATTTTAGCCCAGATTCCCTGTGATGACATTTATAGTGGCCAAGAACTGATTGCCAGAGAAGTGGCAACCAGGTATCTACGCTGCATTTCACAGGCAACAGGCTGGGTCTTTGTGCTGCTAGTAACTCTGCTGGCTTTCCTTGTCCGAGCCATACGTCCCTGCTTTACTCAATCTGCCTTTTTGAAGAGCAAATATTGGTCCCACTACATTGACATTGAGCGGAAGCTCTTTGAAGAGACATGTACAAAACATGCTAAGAGTTTTGCCAAGATTTGCGTTCAGCAATTCTTTGAGAACATAAACAAAGATCCCTTCATGGGCCACACACATATGCACATTCCAGAGAAGGCACCATTAGAGTCAGAGGAAGAAAAAGAACAACTGCTGGGCATCGTAGATCAAGGAACCATGAACAACCTTCTGAAGAACTGGCACAACTGTAAGCCTCCTTTGTGTCTCAATCAAGAGCTGATGCAAAATGGAACCAGCTGGACAAGAGAAATTGCCCAGCCTTGTCCATCAAGAAAAGAATATGCATTCTATTACAGCAAAGTTTGA
