# Supplementary material for: Genomic evidence of bitter taste in snakes and phylogenetic analysis of bitter taste receptor genes in reptiles
Source: PeerJ. 2017 Aug 18;5:e3708. doi: 10.7717/peerj.3708 (PMC5564386; doi:10.7717/peerj.3708)
Supplement: Data S4 [file peerj-05-3708-s004.docx]

>mouse Calhm1 protein

MDKFRMIFQFLQSNQESFMNGICGIMALASAQMYSAFDFNCPCLPGYNVVYSLGILLTPPLVLFLLGLVMNNNISMLAEEWKRPAGRRAKDPAVLRYMFCSMAQRALIAPVVWVAVTLLDGKCFLCAFCTAVPVATLGNGSLVPGLPAPELARLLARVPCPEIYDGNWLLAREVAVRYLRCISQALGWSFVLLTTLLAFVVRSVRPCFTQVAFLKSKYWSHYIDIERKLFDETCTEHAKAFAKVCIQQFFEAMNHDLELGHTHGVLATATATATATEAVQSPSDRTEEEREKLRGITDQGTMNRLLTSWHKCKPPLRLGQEAPLMSNGWAGGEPRPPRKEVATYFSKV

>Anolis_carolinensis

MDKFRMIFQFLQSNQESFMNGICGIMALASAQMYVAFDFTCPCLPGYNLAYGMGILVVPPLVLFLLGFVMNNNVSMLAEEWRRPIGKRQKDPSVLRYMFCSMAQRAMIAPAVWISVTLLHGECFICAFSTTVPIEKLGNDSYMLLPEKEIRKILARIPCKDIYNGQELIAKEVATRYLRCISQAMGWSFVLLMTLLAFLVRSLRPCFTQAAFLKSKYWSHYIDIERKLFDETCTEHAKSFAKVCIQQFFEGMNKDLIMGHTHVPEKTPSEADDEKEKLRGIMDQGTMNKLLKNWHKCKPPLCLNQEVVQNGNCWTGEITRPHLPRREYVTYYSKV

>Gekko_japonicus

MDKFRMIFQFLQSNQESFMNGICGIMALASAQIYVAFDFKCPCLPSYNLAYGMGILFVPPLVLFLLGFVMNNNVSMLAEEWKRPTGKRQKDPAVLRYMFCSMAQRAMIAPAVWVSVTLLDGECFVCAFCTSVPIEKLGNDSYTGLSEKAMRRILAQIPCIEIYSGQELIAREVAIRYLRCISQAIGWTFVLLMTLLAFLVRSLRPCFTQAAFLKSKYWSHYIDIERKLFDETCTEHAKSFAKVCIQQFFEGMNKDLSMGHSHFPEKAPSEAGEEKEKLLGIMDQRTMNKLLKNWHKCKPPLCLNQEVLLNGWAGDIIHSHVPKKEYAAYYSKV

>Crotalus_horridus

MDKFRMIFQFLQSNQESFMNGICGIMALLSAQIYVAFDFKCPCLPSYNLAYGMGILFVPPLVLFLFGFVMNNNISMLAEEWKRPIGKRQKDPAVLRYMFCSMAQRAIIAPVIWISVTLLHGECFICAFSTSVPMHKLGNSSYRPLPEKEIRKILAQIPCNDIYSGQELIAREVATRYLRCISQATGWVFVLLVTLLAFLVRAIRPCFTQSAFLKSKYWSHYIDIERKLFEETCTKHAKSFAKVCVQQFFENMNKDPFMGHTHMHIPEKAPLESEEEKEQLLGIVDQGTMNNLLKNWHNCKPPLCLNQELRQNGTSWTREIAQPCPSRKEYAAYYSKV

>Crotalus_mitchellii

MDKFRMIFQFLQSNQESFMNGICGIMALLSAQIYVAFDFKCPCLPSYNLAYGMGILFVPPLVLFLFGFVMNNNVSMLAEEWKRPIGKRQKDPAVLRYMFCSMAQRAIIAPVIWISVTLLHGECFICAFSTSVPMHKLGNSSYRPLPEKEIRKILAQIPCNDIYSGQELIAREVATRYLRCISQVQATGWVFVLLVTLLAFLVRAIRPCFTQSAFLKSKYWSHYIDIERKLFEETCTKHAKSFAKVCVQQFFENMNKDPFMGHTHMHIPEKAPLESEEEKEQLLGIVDQGTMNNLLKNWHNCKPPLCLNQELRQNGTSWTREIAQPCPSRKEYAAYYSKV

>Ophiophagus_hannah

MDKFRMIFQFLQSNQESFMNGICGIMALLSAQIYVAFDFKCPCLPGYNLAYGMGILFMPPLVLFLFGFVMNNNVSMLAEEWKRPIGKRQKDPAVLRYMFCSMAQRAIIAPVIWISVTLLHGECFICAFSTSVPIHKLGNSSYRHLPEKEIRKILAQIPCDDIYSSQELIAREVATRYLRCISQATGWVFVLLVTLLAFLARAIRPCFTQSAFLKSKYWSHYIDIERKLFEETCTKHAKSFAKVCVQQFFENMNNDPFMGHTHMHIPEKAPSDSDEEKEQLLGIVDQGTMNKLLKNWHNCKPPLCLNPEAMQNGTSWTREIAQPCPSRKEYVAYYSKV

>Pantherophis_guttatus

MDKFRMIFQFLQSNQESFMNGICGIMALLSAQIYVAFDFKCPCLPGYNLAYGMGILFMPPLVLFLFGFVMNNNVSMLAEEWKRPIGKRQKDPAVLRYMFCSMAQRAIIAPVIWISVTLLHGECFICAFSTSVPIHKLGNSSYRHLPEKEIRKILAQIPCDDIYSGQELIAREVATRYLRCISQATGWVFVLLVTLLAFLARAIRPCFTQSAFLKSKYWSHYIDIERKLFEETCTKHAKSFAKVCVQQFFENMNKDPFMGHTHMQIPEKAPSDSEEEKEQLLGIVDQGTMNELLKNWHNCKPPLCLNQELIQNGTSWTREIPQPCPPRKEYAAYYSKV

>Python_bivittatus

MDKFRMIFQFLQSNQESFMNGICGIMALLSAQIYVAFDFKCPCLPGYNLAYGMGILFVPPLVFFLFGFVMNNNVSMLAEEWKRPIGKRQKDPAVLRYMFCSMAQRAMIAPAIWISVTLLHGECFICGFSTSVPMQKLGNNSYRQLPEKEIRKILAQIPCSEIYSGQGLIAREVAIRYLRCISQAMGWSFVLLVTLLAFLIRSIRPCFTQSAFLKSKYWSHYIDIERKIFDETCTKHAKSFAKVCIQQFFENMNKDLFVGHTHMHISEKAPSDSEEEKEQLLGIIDQGTMNKLLKNWHKCKPPLYLNQELMQNGTSWAREIAQPYPPKKEFAAYYSKV

>Thamnophis_sirtalis

MDKFRMIFQFLQSNQESFMNGICGIMALLSAQIYVAFDFKCPCLPGYNMAYGMGILFMPPLVLFLFGFVMNNNVSMLAEEWKRPIGKRQKDPAVLRYMFCSMAQRAIIAPVIWISVTLLHGECFICAFSTSVPMHKLGNSSYRHLPEKEIRKILAQIPCNDIYTGQELIAREVAIRYLRCISQATGWVFVLLVTLLAFLARAIRPCFTQSAFLKSKYWSHYIDIEQKLFEETCTKHAKSFAKVCVQQFFENMNKDPFVGHTHMHIPEKAPSDSEEEKEQLLGIVDQGTMNKLLKNWHNCKPPLCLNQELMQNGTSWTREIAQPCPSKKEYAVYYSKV

>VIPERA

MDKFRMIFQFLQSNQESFMNGICGIMALLSAQIYVAFDFKCPCLPSYNLAYGMGILFVPPLVLFLFGFVMNNNVSMLAEEWKRPIGKRQKDPAVLRYMFCSMAQRAIIAPVIWISVTLLHGECFICAFSTSVPMHKLGNSSYRPLPEKEIRKILAQIPCDDIYSGQELIAREVATRYLRCISQATGWVFVLLVTLLAFLVRAIRPCFTQSAFLKSKYWSHYIDIERKLFEETCTKHAKSFAKICVQQFFENINKDPFMGHTHMHIPEKAPLESEEEKEQLLGIVDQGTMNNLLKNWHNCKPPLCLNQELMQNGTSWTREIAQPCPSRKEYAFYYSKV
