# Supplementary figures and images for: Genomic evidence of bitter taste in snakes and phylogenetic analysis of bitter taste receptor genes in reptiles
Source: PeerJ. 2017 Aug 18;5:e3708. doi: 10.7717/peerj.3708 (PMC5564386; doi:10.7717/peerj.3708)

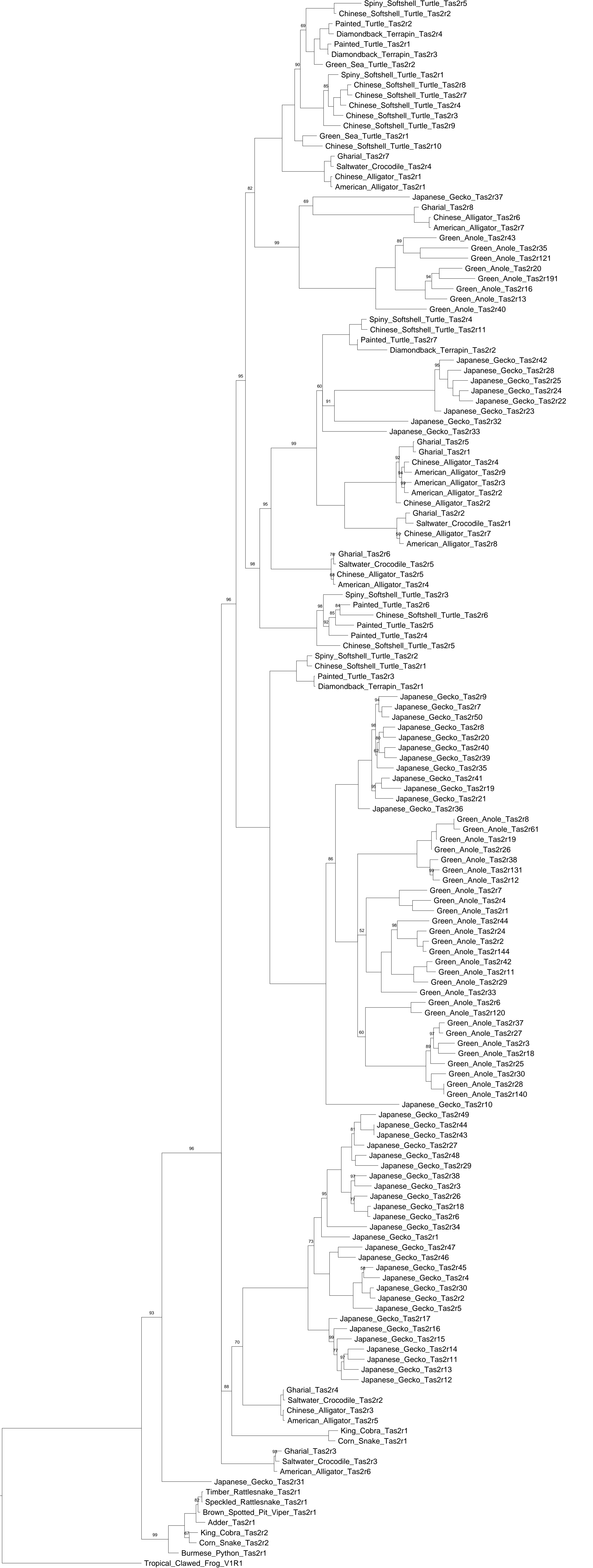

Supplement: Figure S1 [file peerj-05-3708-s005.pdf]

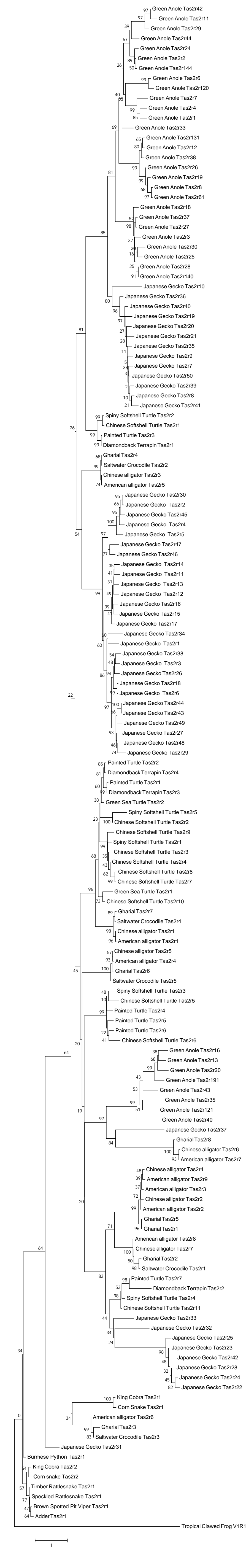

Supplement: Figure S2 [file peerj-05-3708-s006.pdf]
