# Supplementary material for: Genomic evidence of bitter taste in snakes and phylogenetic analysis of bitter taste receptor genes in reptiles
Source: PeerJ. 2017 Aug 18;5:e3708. doi: 10.7717/peerj.3708 (PMC5564386; doi:10.7717/peerj.3708)
Supplement: Table S1 [file peerj-05-3708-s007.docx]

Table S1 References of the dietary preferences and genomic contig N50 statistics for the 19 reptiles studied.

| **Order** | **Species** | **Genome Assembly Number** | **Contig N50 (kbp)** | **Depth(×)** | **Diet** | **References** |
| --- | --- | --- | --- | --- | --- | --- |
| *Squamata* | Corn Snake  (*Pantherophis guttatus*) | GCA_001185365.1 | 2.4 | 13 | carnivore | Sievert et al. 2005  Stake et al. 2005 |
|  | Speckled Rattlesnake  (*Crotalus mitchellii*) | GCA_000737285.1 | 4.1 | 40 | carnivore | Klauber LM. 1997 |
|  | Timber Rattlesnake  (*Crotalus horridus*) | GCA_001625485.1 | 5.8 | 135 | carnivore | Ernst, C., E. Ernst. 2003 |
|  | Adder  (*Vipera berus*) | GCA_000800605.1 | 11.7 | 121 | carnivore | <http://animaldiversity.org> |
|  | Brown Spotted Pit Viper  (*Protobothrops mucrosquamatus*) | GCA_001527695.3 | 21.9 | 85.9 | carnivore | <http://www.iucnredlist.org/details/64323/0> |
|  | King Cobra  (*Ophiophagus Hannah*) | GCA_000516915.1 | 5.2 | 28 | carnivore | <http://animaldiversity.org> |
|  | Burmese Python  (*Python bivittatus*) | GCA_000186305.2 | 10.6 | 20 | carnivore | <http://www.iucnredlist.org/details/193451/0>  7 |
|  | Common Garter Snake  (*Thamnophis sirtalis*) | GCA_001077635.2 | 10.4 | 72 | carnivore | Bartlett, R., P. Bartlett. 2001 |
|  | Green Anole  （*Anolis carolinensis*） | GCA_000090745.2 | 79.9 | 7.1 | insectivore | <http://animaldiversity.org> |
|  | Japanese Gecko  （*Gekko japonicus*） | GCA_001447785.1 | 29.6 | 95 | insectivore | <http://animaldiversity.org> |
| *Testudines* | Spiny Softshell Turtle  （*Apalone spinifera*） | GCA_000385615.1 | 4.7 | 33.4 | carnivore | Harding 1997 |
|  | Green Sea Turtle  （*Chelonia mydas*） | GCA_000344595.1 | 29.2 | 110 | herbivore | Russell et al. 2011 |
|  | Painted Turtle  （*Chrysemys picta*） | GCA_000241765.2 | 21.3 | 15 | omnivore | <http://animaldiversity.org>  <https://en.wikipedia.org/wiki/Painted_turtle#Diet> |
|  | Diamondback Terrapin  (*Malaclemys terrapin*) | GCA_001728815.2 | 437.3 | 16 | carnivore | Tucker, A. et al.. 1997 |
|  | Chinese Softshell Turtle  （*Pelodiscus sinensis*） | GCA_000230535.1 | 22.0 | 105 | carnivore | <https://en.wikipedia.org/wiki/Chinese_softshell_turtle>  C.H. Ernst et al. |
| *Crocodylia* | Saltwater Crocodile  （*Crocodylus porosus*） | GCA_001723895.1 | 34.1 | 74 | carnivore | <http://animaldiversity.org> |
|  | American Alligator  (*Alligator mississippiensis*) | GCA_001541185.1 | 16.7 | 156 | carnivore | <http://animaldiversity.org>  Britton, A. 1999. |
|  | Chinese Alligator  (*Alligator sinensis*) | GCA_000455745.1 | 23.4 | 109 | carnivore | Hutchins et al. |
|  | Gharial  （*Gavialis gangeticus*） | GCA_001723915.1 | 14.8 | 81 | carnivore | <http://animaldiversity.org> |

Bartlett, R., P. Bartlett. 2001. Reptiles Keeper's Guide: Garter and Ribbon Snakes. New York: Barron's Educational Series, Inc.

Ernst, C., E. Ernst. 2003. Snakes of the United States and Canada. Washington, D.C.: Smithsonian Books.

Harding JH. 1997. Amphibians and Reptiles of the Great Lakes Region.

Hutchins, J Murphy, N Schlager, eds. Grizmek's Animal Life Encyclopedia, Vol. 7, 2 Edition.

Klauber LM. 1997. Rattlesnakes: Their Habitats, Life Histories, and Influence on Mankind. Second Edition. First published in 1956, 1972. Berkeley: University of California Press. ISBN 0-520-21056-5.

Russell DJ, Hargrove S, and Balazs GH. 2011. Marine Sponges, Other Animal Food, and Nonfood Items Found in Digestive Tracts of the Herbivorous Marine Turtle Chelonia mydas in Hawai'i. Pacific Science 65:253-260.

Sievert LM, Jones DM, and Puckett MW. 2005. Postprandial thermophily, transit rate, and digestive efficiency of juvenile cornsnakes, Pantherophis guttatus. Journal of Thermal Biology 30:354-359.

Stake MM, Iii FRT, Faaborg J, and Burhans DE. 2005. Patterns of Snake Predation at Songbird Nests in Missouri and Texas. Journal of Herpetology 39:215-222.

Tucker, A., S. Yeomans, J. Gibbons. 1997. Shell strength of mud snails (Ilyanassa obsoleta) may deter foraging by Diamondback Terrapins (Malaclemys terrapin). The American Midland Naturalist, 138: 224-229.
