# Supplementary material for: Genomic evidence of bitter taste in snakes and phylogenetic analysis of bitter taste receptor genes in reptiles
Source: PeerJ. 2017 Aug 18;5:e3708. doi: 10.7717/peerj.3708 (PMC5564386; doi:10.7717/peerj.3708)
Supplement: Table S2 [file peerj-05-3708-s008.docx]

Table S2. Classification of partial *Tas2rs* in the genome assemblies of Reptiles. Overlapping partial *Tas2rs* with similar orthologies in the multiple alignments were regarded as being derived from different loci. In contrast, non-overlapping *Tas2rs* were regarded as being derived from the same loci with gap(s).

| Species | Different orthologs | Overlapping TAS2Rs  (potentially intact paralogs) | Non-overlapping TAS2Rs  (potentially intact gene from  same locus) | **Intact**  **TAS2Rs** | **Partial *TAS2R*s** | **Partial *TAS2R* loci** | **Potentially intact**  **TAS2Rs** |
| --- | --- | --- | --- | --- | --- | --- | --- |
| Japanese Gecko  (*Gekko japonicas*) | - | Tas2r41_P, Tas2r40_P | - | 50 | 2 | 2 | 52 |
| Gharial  (*Gavialis gangeticus*) | Tas2r9_P | - | - | 8 | 1 | 1 | 9 |
