# Supplementary material for: Genomic evidence of bitter taste in snakes and phylogenetic analysis of bitter taste receptor genes in reptiles
Source: PeerJ. 2017 Aug 18;5:e3708. doi: 10.7717/peerj.3708 (PMC5564386; doi:10.7717/peerj.3708)
Supplement: Table S3 [file peerj-05-3708-s009.docx]

Table S3. Syntenic analysis for partial genes.

| Species | Gene name | Scaffold | Upstream gene | Downstream gene |
| --- | --- | --- | --- | --- |
| Japanese Gecko  (*Gekko japonicas*) | Tas2r40_P | 98533 | No blast hit | NA |
|  | Tas2r41_P | 20406 | No blast hit | NA |
| Gharial  (*Gavialis gangeticus*) | Tas2r9_P | C15964618_1 | No blast hit | NA |

NA: No available data due to short contig
