# Supplementary material for: Genomic evidence of bitter taste in snakes and phylogenetic analysis of bitter taste receptor genes in reptiles
Source: PeerJ. 2017 Aug 18;5:e3708. doi: 10.7717/peerj.3708 (PMC5564386; doi:10.7717/peerj.3708)
Supplement: Table S4 [file peerj-05-3708-s010.docx]

Table S4 References of divergence time tree taken from http://www.timetree.org/

|  | **Title** | **Authors** | **Year** |
| --- | --- | --- | --- |
| 1 | A divergence dating analysis of turtles using fossil calibrations: and example of best practices | Joyce | 2013 |
| 2 | A likelihood method for assessing molecular divergence time estimates and the placement of fossil calibrations. | Pyron | 2010 |
| 3 | A mitogenomic timescale for birds detects variable phylogenetic rates of molecular evolution and refutes the standard molecular clock. | Pereira | 2006 |
| 4 | A molecular phylogeny of reptiles. | Hedges | 1999 |
| 5 | A molecular timescale for vertebrate evolution. | Kumar | 1998 |
| 6 | A multilocus timescale for the origin of extant amphibians. | San Mauro | 2010 |
| 7 | A nesting of vipers: Phylogeny and historical biogeography of the Viperidae (Squamata: Serpentes). | Wüster | 2008 |
| 8 | A phylogeny of softshelled turtles (Testudines: Trionychidae) with reference to the taxonomic status of the critically endangered, giant softshell turtle, Rafetus swinhoei | Le | 2014 |
| 9 | A time-calibrated phylogenetic approach to assessing the phylogeography+ colonization history and phenotypic evolution of snakes in the Japanese Izu Islands | Kuriyama | 2011 |
| 10 | A time-calibrated species tree of Crocodylia reveals a recent radiation of the true crocodiles. | Oaks | 2011 |
| 11 | Assessing concordance of fossil calibration points in molecular clock studies: an example using turtles. | Near | 2005 |
| 12 | Basal jawed vertebrate phylogenomics using transcriptomic data from Solexa sequencing. | Chen | 2012 |
| 13 | Blindsnake evolutionary tree reveals long history on Gondwana. | Vidal | 2010 |
| 14 | Calibration age and quartet divergence date estimation. | Brochu | 2004 |
| 15 | Calibration choice, rate smoothing, and the pattern of tetrapod diversification according to the long nuclear gene RAG-1. | Hugall | 2007 |
| 16 | Combining phylogenomic and supermatrix approaches, and a time-calibrated phylogeny for squamate reptiles (lizards and snakes) based on 52 genes and 4162 species. | Zheng | 2016 |
| 17 | Coming to America: multiple origins of New World geckos. | Gamble | 2011 |
| 18 | Comparative phylogeography of pitvipers suggests a consensus of ancient Middle American highland biogeography | Castoe | 2009 |
| 19 | Comparative phylogeography reveals distinct colonization patterns of Cretan snakes | Kyriazi | 2013 |
| 20 | Complete mitochondrial DNA genome sequences show that modern birds are not descended from transitional shorebirds. | Paton | 2002 |
| 21 | Complex evolution in the Neotropics: the origin and diversification of the widespread genus Leptodeira (Serpentes: Colubridae). | Daza | 2009 |
| 22 | Dating cryptodiran nodes: origin and diversification of the turtle superfamily Testudinoidea. | Lourenço | 2012 |
| 23 | Dispersal and vicariance: the complex evolutionary history of boid snakes. | Noonan | 2006 |
| 24 | Diversity-dependent cladogenesis throughout western Mexico: Evolutionary biogeography of rattlesnakes (Viperidae: Crotalinae: Crotalus and Sistrurus). | Blair | 2016 |
| 25 | Do missing data influence the accuracy of divergence-time estimation with BEAST? | Zheng | 2015 |
| 26 | Dynamic nucleotide mutation gradients and control region usage in squamate reptile mitochondrial genomes. | Castoe | 2009 |
| 27 | Eastward from Africa: palaeocurrent-mediated chameleon dispersal to the Seychelles islands. | Townsend | 2011 |
| 28 | Effect of taxon sampling on recovering the phylogeny of squamate reptiles based on complete mitochondrial genome and nuclear gene sequence data. | Albert | 2009 |
| 29 | Estimating divergence dates and evaluating dating methods using phylogenomic and mitochondrial data in squamate reptiles. | Mulcahy | 2012 |
| 30 | Evolution of rattlesnakes (Viperidae; Crotalus) in the warm deserts of western North America shaped by Neogene vicariance and Quaternary climate change. | Douglas | 2006 |
| 31 | Evolutionary origin and phylogeny of the modern holocephalans (Chondrichthyes: Chimaeriformes): a mitogenomic perspective. | Inoue | 2010 |
| 32 | Evolutionary relationships of marine turtles: A molecular phylogeny based on nuclear and mitochondrial genes. | Naro-Maciel | 2008 |
| 33 | Extended mitogenomic phylogenetic analyses yield new insight into crocodylian evolution and their survival of the Cretaceous-Tertiary boundary. | Roos | 2007 |
| 34 | Extinction, ecological opportunity, and the origins of global snake diversity. | Pyron | 2012 |
| 35 | Fossil-based comparative analyses reveal ancient marine ancestry erased by extinction in ray-finned fishes. | Betancur-R | 2015 |
| 36 | Four new avian mitochondrial genomes help get to basic evolutionary questions in the late cretaceous. | Harrison | 2004 |
| 37 | Higher-level salamander relationships and divergence dates inferred from complete mitochondrial genomes. | Zhang | 2009 |
| 38 | Identical skin toxins by convergent molecular adaptation in frogs. | Roelants | 2010 |
| 39 | Impacts of the Cretaceous Terrestrial Revolution and KPg extinction on mammal diversification. | Meredith | 2011 |
| 40 | Integrating fossil preservation biases in the selection of calibrations for molecular divergence time estimation. | Dornburg | 2011 |
| 41 | Integration of molecules and new fossils supports a Triassic origin for Lepidosauria (lizards, snakes, and tuatara). | Jones | 2013 |
| 42 | Is there convergence in the molecular pathways underlying the repeated evolution of sociality in African cichlids? | O'Connor | 2015 |
| 43 | Large-scale phylogeny of chameleons suggests African origins and Eocene diversification. | Tolley | 2013 |
| 44 | Mitochondrial genomes from major lizard families suggest their phylogenetic relationships and ancient radiations. | Kumazawa | 2007 |
| 45 | Mitogenomic analyses place the gharial (Gavialis gangeticus) on the crocodile tree and provide pre-K/T divergence times for most crocodilians. | Janke | 2005 |
| 46 | Mitogenomic perspectives into iguanid phylogeny and biogeography: Gondwanan vicariance for the origin of Madagascan oplurines. | Okajima | 2009 |
| 47 | Mitogenomic perspectives on the origin and phylogeny of living amphibians. | Zhang | 2005 |
| 48 | Molecular evidence for a clade of turtles. | Mannen | 1999 |
| 49 | Molecular evidence for a rapid late-Miocene radiation of Australasian venomous snakes (Elapidae, Colubroidea). | Sanders | 2008 |
| 50 | Molecular phylogenetic and dating analyses using mitochondrial DNA sequences of eyelid geckos (Squamata: Eublepharidae). | Jonniaux | 2008 |
| 51 | Molecular phylogeny, classification, and biogeography of snakes of the Family Leptotyphlopidae (Reptilia, Squamata) | Adalsteinsson | 2009 |
| 52 | Molecular systematics of primary reptilian lineages and the tuatara mitochondrial genome. | Rest | 2003 |
| 53 | Molecular systematics of racers\, whipsnakes and relatives (Reptilia : Colubridae) using mitochondrial and nuclear markers | Nagy | 2004 |
| 54 | Monitors cross the Red Sea: the biogeographic history of Varanus yemenensis. | Portik | 2012 |
| 55 | Multiple colonization of Madagascar and Socotra by colubrid snakes: evidence from nuclear and mitochondrial gene phylogenies. | Nagy | 2003 |
| 56 | Nine exceptional radiations plus high turnover explain species diversity in jawed vertebrates. | Alfaro | 2009 |
| 57 | One tree to link them all: a phylogenetic dataset for the European tetrapoda. | Roquet | 2014 |
| 58 | P66shc and its downstream Eps8 and Rac1 proteins are upregulated in esophageal cancers. | Bashir | 2010 |
| 59 | Phylogenetic analysis of reptilian hemoglobins: trees, rates, and divergences. | Gorr | 1998 |
| 60 | Phylogenetic evidence of historic mitochondrial introgression and cryptic diversity in the genus Pseudemoia (Squamata: Scincidae). | Haines | 2014 |
| 61 | Phylogenetic relationships of the enigmatic longtailed rattlesnakes (Crotalus ericsmithi, C. lannomi, and C. stejnegeri). | Reyes-Velasco | 2013 |
| 62 | Phylogenetic relationships within the lizard clade Xantusiidae: using trees and divergence times to address evolutionary questions at multiple levels. | Noonan | 2013 |
| 63 | Phylogenomic analyses support the position of turtles as the sister group of birds and crocodiles (Archosauria). | Chiari | 2012 |
| 64 | Phylogeny and divergence times of filesnakes (Acrochordus): inferences from morphology, fossils and three molecular loci. | Sanders | 2010 |
| 65 | Phylogeny of a trans-Wallacean radiation (Squamata\, Gekkonidae\, Gehyra) supports a single early colonization of Australia | Heinicke | 2011 |
| 66 | Phylogeny of iguanian lizards inferred from 29 nuclear loci, and a comparison of concatenated and species-tree approaches for an ancient, rapid radiation. | Townsend | 2011 |
| 67 | Phylogeography of Northern Populations of the Black-Tailed Rattlesnake (Crotalus molossus Baird And Girard, 1853), With the Revalidation of C. ornatus Hallowell, 1854 | Anderson | 2012 |
| 68 | Rapid diversification and dispersal during periods of global warming by plethodontid salamanders. | Vieites | 2007 |
| 69 | Repeated evolution of sympatric, palaeoendemic species in closely related, co鈥恉istributed lineages of Hemiphyllodactylus Bleeker, 1860 (Squamata: Gekkonidae) across a sky鈥恑sland archipelago in Peninsular Malaysia | Grismer | 2015 |
| 70 | Rigorous approaches to species delimitation have significant implications for African crocodilian systematics and conservation. | Shirley | 2014 |
| 71 | Role of Chromosome Changes in Crocodylus Evolution and Diversity. | Srikulnath | 2015 |
| 72 | Testing species-level diversification hypotheses in Madagascar: the case of microendemic Brookesia leaf chameleons. | Townsend | 2009 |
| 73 | The complete mitochondrial DNA sequence and the phylogenetic position of Achalinus meiguensis (Reptilia: Squamata) | Wang | 2009 |
| 74 | The complete mitochondrial genome of a tree frog, Polypedates megacephalus (Amphibia: Anura: Rhacophoridae), and a novel gene organization in living amphibians. | Zhang | 2005 |
| 75 | The complete mitochondrial genome sequences of Chelodina rugosa and Chelus fimbriata (Pleurodira: Chelidae): implications of a common absence of initiation sites (O(L)) in pleurodiran turtles. | Wang | 2012 |
| 76 | The determinants of the molecular substitution process in turtles. | Lourenço | 2013 |
| 77 | The development of three long universal nuclear protein-coding locus markers and their application to osteichthyan phylogenetics with nested PCR. | Shen | 2012 |
| 78 | The draft genomes of soft-shell turtle and green sea turtle yield insights into the development and evolution of the turtle-specific body plan. | Wang | 2013 |
| 79 | The mitochondrial genomes of the iguana (Iguana iguana) and the caiman (Caiman crocodylus): implications for amniote phylogeny. | Janke | 2001 |
| 80 | The origin of snakes: revealing the ecology, behavior, and evolutionary history of early snakes using genomics, phenomics, and the fossil record. | Hsiang | 2015 |
| 81 | The phylogenetic position and taxonomic status of the Rainbow Tree Snake Gonyophis margaritatus (Peters, 1871) (Squamata: Colubridae)聽. | Chen | 2014 |
| 82 | The phylogeny of squamate reptiles (lizards, snakes, and amphisbaenians) inferred from nine nuclear protein-coding genes. | Vidal | 2005 |
| 83 | The taming of the skew: estimating proper confidence intervals for divergence dates. | Burbrink | 2008 |
| 84 | The tree of life and a new classification of bony fishes. | Betancur-R | 2013 |
| 85 | Timing of a mtDNA gene rearrangement and intercontinental dispersal of varanid lizards. | Amer | 2008 |
| 86 | Uncertainty in the Timing of Origin of Animals and the Limits of Precision in Molecular Timescales. | dos Reis | 2015 |
| 87 | Understanding the formation of ancient intertropical disjunct distributions using Asian and Neotropical hinged-teeth snakes (Sibynophis and Scaphiodontophis: Serpentes: Colubridae). | Chen | 2013 |
| 88 | Using regional comparative phylogeographic data from snake lineages to infer historical processes in Middle America | Daza | 2010 |
| 89 | Vertebrate time-tree elucidates the biogeographic pattern of a major biotic change around the K-T boundary in Madagascar. | Crottini | 2012 |
| 90 | When continents collide: phylogeny, historical biogeography and systematics of the medically important viper genus Echis (Squamata: Serpentes: Viperidae). | Pook | 2009 |
| 91 | Why does a trait evolve multiple times within a clade? Repeated evolution of snakelike body form in squamate reptiles. | Wiens | 2006 |
