# Supplementary material for: Genomic evidence of bitter taste in snakes and phylogenetic analysis of bitter taste receptor genes in reptiles
Source: PeerJ. 2017 Aug 18;5:e3708. doi: 10.7717/peerj.3708 (PMC5564386; doi:10.7717/peerj.3708)
Supplement: Table S5 [file peerj-05-3708-s011.docx]

Table S5. Genomic locations for all reptile Tas2r genes studied. Potential tandem duplicated genes were highlighted in shadow.

| **Species** | **Gene Name** | **Gene Type** | **Genomic Location** | |
| --- | --- | --- | --- | --- |
|  |  |  | **Scaffold/** **chromosome** | **Reference Sequence** |
| Corn Snake  (*Pantherophis guttatus*) | Corn_Snake_Tas2r1 | Intact | 243989 | JTLQ01235169.1 |
|  | Corn_Snake_Tas2r2 | Intact | 380024 | JTLQ01370171.1 |
| Speckled Rattlesnake  (*Crotalus mitchellii*) | Speckled_Rattlesnake_Tas2r1 | Intact | 117812 | JPMF01117140.1 |
|  | Speckled_Rattlesnake_Tas2r2_Ps | Pseudogene | 32064 | JPMF01031890.1 |
| Timber Rattlesnake  (*Crotalus horridus*) | Timber_Rattlesnake_Tas2r1 | Intact | Sequence_63596_10946 | LVCR01063596.1 |
|  | Timber_Rattlesnake_Tas2r2_Ps | Pseudogene | Sequence_67565_3330 | LVCR01067565.1 |
|  | Timber_Rattlesnake_Tas2r3_Ps | Pseudogene | Sequence_67565_3330 | LVCR01067565.1 |
| Adder  (*Vipera berus*) | Adder_Tas2r1 | Intact | 5319 | KN633984.1 |
|  | Adder_Tas2r2_Ps | Pseudogene | 5319 | KN633984.1 |
|  | Adder_Tas2r3_Ps | Pseudogene | 5319 | KN633984.1 |
| Brown spot pit viper  (*Protobothrops mucrosquamatus*) | Brown_spot_pit_viper_Tas2r1 | Intact | 3244 | NW_015389238.1 |
|  | Brown_spot_pit_viper_Tas2r2_Ps | Pseudogene | 3244 | NW_015389238.1 |
| King Cobra  (*Ophiophagus Hannah*) | King_Cobra_Tas2r1 | Intact | 3907.1 | AZIM01003904.1 |
|  | King_Cobra_Tas2r2 | Intact | 3907.1 | AZIM01003904.1 |
| *Python bivittatus* | Burmese_Python_Tas2r1 | Intact | 6811 | NW_006538825.1 |
|  | Burmese_Python_Tas2r2_Ps | Pseudogene | 6811 | NW_006538825.1 |
|  | Burmese_Python_Tas2r3_Ps | Pseudogene | 6811 | NW_006538825.1 |
| *Anolis carolinensis* | Green_Anole_Tas2r6 | Intact | chromosome 2 | NC_014777.1 |
|  | Green_Anole_Tas2r13 | Intact | chromosome 2 | NC_014777.1 |
|  | Green_Anole_Tas2r18 | Intact | chromosome 2 | NC_014777.1 |
|  | Green_Anole_Tas2r24 | Intact | chromosome 2 | NC_014777.1 |
|  | Green_Anole_Tas2r27 | Intact | chromosome 2 | NC_014777.1 |
|  | Green_Anole_Tas2r28 | Intact | chromosome 2 | NC_014777.1 |
|  | Green_Anole_Tas2r34 | Intact | chromosome 2 | NC_014777.1 |
|  | Green_Anole_Tas2r36 | Intact | chromosome 2 | NC_014777.1 |
|  | Green_Anole_Tas2r43_Ps | Pseudogene | chromosome 2 | NC_014777.1 |
|  | Green_Anole_Tas2r44_Ps | Pseudogene | chromosome 2 | NC_014777.1 |
|  | Green_Anole_Tas2r47_Ps | Pseudogene | chromosome 2 | NC_014777.1 |
|  | Green_Anole_Tas2r48_Ps | Pseudogene | chromosome 3 | NC_014778.1 |
|  | Green_Anole_Tas2r3 | Intact | chromosome 5 | NC_014780.1 |
|  | Green_Anole_Tas2r5 | Intact | chromosome 5 | NC_014780.1 |
|  | Green_Anole_Tas2r10 | Intact | chromosome 5 | NC_014780.1 |
|  | Green_Anole_Tas2r19 | Intact | chromosome 5 | NC_014780.1 |
|  | Green_Anole_Tas2r21 | Intact | chromosome 5 | NC_014780.1 |
|  | Green_Anole_Tas2r22 | Intact | chromosome 5 | NC_014780.1 |
|  | Green_Anole_Tas2r12 | Intact | Unknown | NW_003338954.1 |
|  | Green_Anole_Tas2r4 | Intact | Unknown | NW_003338928.1 |
|  | Green_Anole_Tas2r7 | Intact | Unknown | NW_003338928.1 |
|  | Green_Anole_Tas2r8 | Intact | Unknown | NW_003338928.1 |
|  | Green_Anole_Tas2r9 | Intact | Unknown | NW_003338928.1 |
|  | Green_Anole_Tas2r11 | Intact | Unknown | NW_003338928.1 |
|  | Green_Anole_Tas2r14 | Intact | Unknown | NW_003338928.1 |
|  | Green_Anole_Tas2r15 | Intact | Unknown | NW_003338928.1 |
|  | Green_Anole_Tas2r16 | Intact | Unknown | NW_003338928.1 |
|  | Green_Anole_Tas2r17 | Intact | Unknown | NW_003338928.1 |
|  | Green_Anole_Tas2r20 | Intact | Unknown | NW_003338928.1 |
|  | Green_Anole_Tas2r23 | Intact | Unknown | NW_003338928.1 |
|  | Green_Anole_Tas2r25 | Intact | Unknown | NW_003338928.1 |
|  | Green_Anole_Tas2r26 | Intact | Unknown | NW_003338928.1 |
|  | Green_Anole_Tas2r29 | Intact | Unknown | NW_003338928.1 |
|  | Green_Anole_Tas2r30 | Intact | Unknown | NW_003338928.1 |
|  | Green_Anole_Tas2r32 | Intact | Unknown | NW_003338928.1 |
|  | Green_Anole_Tas2r33 | Intact | Unknown | NW_003338928.1 |
|  | Green_Anole_Tas2r38_Ps | Pseudogene | Unknown | NW_003338928.1 |
|  | Green_Anole_Tas2r39_Ps | Pseudogene | Unknown | NW_003338928.1 |
|  | Green_Anole_Tas2r40_Ps | Pseudogene | Unknown | NW_003338928.1 |
|  | Green_Anole_Tas2r41_Ps | Pseudogene | Unknown | NW_003338928.1 |
|  | Green_Anole_Tas2r45_Ps | Pseudogene | Unknown | NW_003338928.1 |
|  | Green_Anole_Tas2r46_Ps | Pseudogene | Unknown | NW_003338928.1 |
|  | Green_Anole_Tas2r49_Ps | Pseudogene | Unknown | NW_003338928.1 |
|  | Green_Anole_Tas2r50_Ps | Pseudogene | Unknown | NW_003338928.1 |
|  | Green_Anole_Tas2r37_Ps | Pseudogene | Unknown | NW_003339307.1 |
|  | Green_Anole_Tas2r2 | Intact | Unknown | NW_003338938.1 |
|  | Green_Anole_Tas2r31 | Intact | Unknown | NW_003338938.1 |
|  | Green_Anole_Tas2r35 | Intact | Unknown | NW_003338938.1 |
|  | Green_Anole_Tas2r42_Ps | Pseudogene | Unknown | NW_003338938.1 |
|  | Green_Anole_Tas2r1 | Intact | Unknown | NW_003343612.1 |
| *Gekko japonicas* | Japanese_Gecko_Tas2r32 | Intact | 1516 | NW_015172050.1 |
|  | Japanese_Gecko_Tas2r33 | Intact | 1516 | NW_015172050.1 |
|  | Japanese_Gecko_Tas2r52_Ps | Pseudogene | 1516 | NW_015172050.1 |
|  | Japanese_Gecko_Tas2r36 | Intact | 7560 | NW_015167766.1 |
|  | Japanese_Gecko_Tas2r19 | Intact | 1664 | NW_015169828.1 |
|  | Japanese_Gecko_Tas2r20 | Intact | 1664 | NW_015169828.1 |
|  | Japanese_Gecko_Tas2r21 | Intact | 1664 | NW_015169828.1 |
|  | Japanese_Gecko_Tas2r38 | Intact | 1664 | NW_015169828.1 |
|  | Japanese_Gecko_Tas2r39 | Intact | 1664 | NW_015169828.1 |
|  | Japanese_Gecko_Tas2r40 | Intact | 1664 | NW_015169828.1 |
|  | Japanese_Gecko_Tas2r62_Ps | Pseudogene | 1664 | NW_015169828.1 |
|  | Japanese_Gecko_Tas2r62_Ps | Pseudogene | 9237 | NW_015172278.1 |
|  | Japanese_Gecko_Tas2r1 | Intact | 2520 | NW_015163679.1 |
|  | Japanese_Gecko_Tas2r2 | Intact | 2520 | NW_015163679.1 |
|  | Japanese_Gecko_Tas2r7 | Intact | 2520 | NW_015163679.1 |
|  | Japanese_Gecko_Tas2r8 | Intact | 2520 | NW_015163679.1 |
|  | Japanese_Gecko_Tas2r9 | Intact | 2520 | NW_015163679.1 |
|  | Japanese_Gecko_Tas2r10 | Intact | 2520 | NW_015163679.1 |
|  | Japanese_Gecko_Tas2r11 | Intact | 2520 | NW_015163679.1 |
|  | Japanese_Gecko_Tas2r12 | Intact | 2520 | NW_015163679.1 |
|  | Japanese_Gecko_Tas2r13 | Intact | 2520 | NW_015163679.1 |
|  | Japanese_Gecko_Tas2r14 | Intact | 2520 | NW_015163679.1 |
|  | Japanese_Gecko_Tas2r15 | Intact | 2520 | NW_015163679.1 |
|  | Japanese_Gecko_Tas2r16 | Intact | 2520 | NW_015163679.1 |
|  | Japanese_Gecko_Tas2r17 | Intact | 2520 | NW_015163679.1 |
|  | Japanese_Gecko_Tas2r33 | Intact | 2520 | NW_015163679.1 |
|  | Japanese_Gecko_Tas2r34 | Intact | 2520 | NW_015163679.1 |
|  | Japanese_Gecko_Tas2r35 | Intact | 2520 | NW_015163679.1 |
|  | Japanese_Gecko_Tas2r49 | Intact | 2520 | NW_015163679.1 |
|  | Japanese_Gecko_Tas2r53_Ps | Pseudogene | 2520 | NW_015163679.1 |
|  | Japanese_Gecko_Tas2r54_Ps | Pseudogene | 2520 | NW_015163679.1 |
|  | Japanese_Gecko_Tas2r58_Ps | Pseudogene | 2520 | NW_015163679.1 |
|  | Japanese_Gecko_Tas2r59_Ps | Pseudogene | 2520 | NW_015163679.1 |
|  | Japanese_Gecko_Tas2r60_Ps | Pseudogene | 2520 | NW_015163679.1 |
|  | Japanese_Gecko_Tas2r50_Ps | Pseudogene | 2793 | NW_015164744.1 |
|  | Japanese_Gecko_Tas2r3 | Intact | 4085 | NW_015161533.1 |
|  | Japanese_Gecko_Tas2r4 | Intact | 4085 | NW_015161533.1 |
|  | Japanese_Gecko_Tas2r45 | Intact | 4085 | NW_015161533.1 |
|  | Japanese_Gecko_Tas2r46 | Intact | 4085 | NW_015161533.1 |
|  | Japanese_Gecko_Tas2r55_Ps | Pseudogene | 4085 | NW_015161533.1 |
|  | Japanese_Gecko_Tas2r56_Ps | Pseudogene | 4085 | NW_015161533.1 |
|  | Japanese_Gecko_Tas2r57_Ps | Pseudogene | 4085 | NW_015161533.1 |
|  | Japanese_Gecko_Tas2r56_Ps | Pseudogene | 11585 | NW_015213287.1 |
|  | Japanese_Gecko_Tas2r22 | Intact | 4176 | NW_015172676.1 |
|  | Japanese_Gecko_Tas2r23 | Intact | 4176 | NW_015172676.1 |
|  | Japanese_Gecko_Tas2r24 | Intact | 4176 | NW_015172676.1 |
|  | Japanese_Gecko_Tas2r25 | Intact | 4176 | NW_015172676.1 |
|  | Japanese_Gecko_Tas2r31 | Intact | 4176 | NW_015172676.1 |
|  | Japanese_Gecko_Tas2r32 | Intact | 4176 | NW_015172676.1 |
|  | Japanese_Gecko_Tas2r41 | Intact | 4176 | NW_015172676.1 |
|  | Japanese_Gecko_Tas2r53_Ps | Pseudogene | 15197 | NW_015190047.1 |
|  | Japanese_Gecko_Tas2r69_Ps | Pseudogene | 4265 | NW_015165961.1 |
|  | Japanese_Gecko_Tas2r70_Ps | Pseudogene | 4265 | NW_015165961.1 |
|  | Japanese_Gecko_Tas2r54_Ps | Pseudogene | 18953 | NW_015190143.1 |
|  | Japanese_Gecko_Tas2r26 | Intact | 5381 | NW_015175014.1 |
|  | Japanese_Gecko_Tas2r27 | Intact | 5381 | NW_015175014.1 |
|  | Japanese_Gecko_Tas2r28 | Intact | 5381 | NW_015175014.1 |
|  | Japanese_Gecko_Tas2r41_P | Partial | 20406 | NW_015213480.1 |
|  | Japanese_Gecko_Tas2r6 | Intact | 5983 | NW_015162162.1 |
|  | Japanese_Gecko_Tas2r29 | Intact | 5983 | NW_015207162.1 |
|  | Japanese_Gecko_Tas2r44 | Intact | 5983 | NW_015207162.1 |
|  | Japanese_Gecko_Tas2r47 | Intact | 6799 | NW_015162162.1 |
|  | Japanese_Gecko_Tas2r48 | Intact | 6799 | NW_015162162.1 |
|  | Japanese_Gecko_Tas2r50 | Intact | 6813 | NW_015174458.1 |
|  | Japanese_Gecko_Tas2r18 | Intact | 7370 | NW_015168253.1 |
|  | Japanese_Gecko_Tas2r37 | Intact | 7370 | NW_015168253.1 |
|  | Japanese_Gecko_Tas2r30 | Intact | 30495 | NW_015219447.1 |
|  | Japanese_Gecko_Tas2r55_Ps | Pseudogene | 63497 | NW_015200626.1 |
|  | Japanese_Gecko_Tas2r40_P | Partial | 98533 | NW_015203419.1 |
| *Spiny_Soft-shell_Turtle* | Spiny_Soft-shell_Turtle_Tas2r8_Ps | Pseudogene | 114 | KB928071.1 |
|  | Spiny_Soft-shell_Turtle_Tas2r2 | Intact | 3241 | KB930101.1 |
|  | Spiny_Soft-shell_Turtle_Tas2r9_Ps | Pseudogene | 3241 | KB930101.1 |
|  | Spiny_Soft-shell_Turtle_Tas2r10_Ps | Pseudogene | 3241 | KB930101.1 |
|  | Spiny_Soft-shell_Turtle_Tas2r7_Ps | Pseudogene | 3765 | KB930558.1 |
|  | Spiny_Soft-shell_Turtle_Tas2r5_Ps | Pseudogene | 3300 | KB930154.1 |
|  | Spiny_Soft-shell_Turtle_Tas2r6_Ps | Pseudogene | 3300 | KB930154.1 |
|  | Spiny_Soft-shell_Turtle_Tas2r4_Ps | Pseudogene | 19151.1 | APJP01342576.1 |
|  | Spiny_Soft-shell_Turtle_Tas2r1 | Intact | 50713.1 | APJP01712690.1 |
|  | Spiny_Soft-shell_Turtle_Tas2r3_Ps | Pseudogene | 1384705 | APJP01253257.1 |
| *Chelonia mydas* | Green_Sea_Turtle_Tas2r1 | Intact | 21 | NW_006623410.1 |
|  | Green_Sea_Turtle_Tas2r2 | Intact | 21 | NW_006623410.1 |
|  | Green_Sea_Turtle_Tas2r3_Ps | Pseudogene | 21 | NW_006623410.1 |
|  | Green_Sea_Turtle_Tas2r4_Ps | Pseudogene | 320 | NW_006635626.1 |
|  | Green_Sea_Turtle_Tas2r5_Ps | Pseudogene | 1108 | NW_006583126.1 |
|  | Green_Sea_Turtle_Tas2r6_Ps | Pseudogene | 2488 | NW_006627719.1 |
|  | Green_Sea_Turtle_Tas2r7_Ps | Pseudogene | 5585 | NW_006662117.1 |
| Painted Turtle  (*Chrysemys picta*) | Painted_Turtle_Tas2r1 | Intact | 55 | NW_007281384.1 |
|  | Painted_Turtle_Tas2r2 | Intact | 55 | NW_007281384.1 |
|  | Painted_Turtle_Tas2r8_Ps | Pseudogene | 55 | NW_007281384.1 |
|  | Painted_Turtle_Tas2r9_Ps | Pseudogene | 69 | NW_007281397.1 |
|  | Painted_Turtle_Tas2r3 | Pseudogene | 180 | NW_007281499.1 |
|  | Painted_Turtle_Tas2r10_Ps | Pseudogene | 180 | NW_007281499.1 |
|  | Painted_Turtle_Tas2r11_Ps | Pseudogene | 180 | NW_007281499.1 |
|  | Painted_Turtle_Tas2r12_Ps | Pseudogene | 180 | NW_007281499.1 |
|  | Painted_Turtle_Tas2r7 | Intact | 78574 | NW_007359875.1 |
|  | Painted_Turtle_Tas2r4 | Intact | 78588 | NW_007359885.1 |
|  | Painted_Turtle_Tas2r5 | Pseudogene | 78588 | NW_007359885.1 |
|  | Painted_Turtle_Tas2r6 | Pseudogene | 78588 | NW_007359885.1 |
|  | Painted_Turtle_Tas2r13_Ps | Pseudogene | 78588 | NW_007359885.1 |
|  | Painted_Turtle_Tas2r14_Ps | Pseudogene | 78588 | NW_007359885.1 |
| Diamondback Terrapin  (*Malaclemys terrapin*) | Diamondback_Terrapin_Tas2r1 | Intact | Unknown | MDXI01008514.1 |
|  | Diamondback_Terrapin_Tas2r2 | Intact | Unknown | MDXI01018911.1 |
|  | Diamondback_Terrapin_Tas2r3 | Intact | Unknown | MDXI01019222.1 |
|  | Diamondback_Terrapin_Tas2r4 | Intact | Unknown | MDXI01019226.1 |
|  | Diamondback_Terrapin_Tas2r5Ps | Pseudogene | Unknown | MDXI01004202.1 |
|  | Diamondback_Terrapin_Tas2r6Ps | Pseudogene | Unknown | MDXI01004202.1 |
|  | Diamondback_Terrapin_Tas2r7Ps | Pseudogene | Unknown | MDXI01005383.1 |
|  | Diamondback_Terrapin_Tas2r8Ps | Pseudogene | Unknown | MDXI01005383.1 |
|  | Diamondback_Terrapin_Tas2r9Ps | Pseudogene | Unknown | MDXI01008915.1 |
|  | Diamondback_Terrapin_Tas2r10Ps | Pseudogene | Unknown | MDXI01011651.1 |
|  | Diamondback_Terrapin_Tas2r11Ps | Pseudogene | Unknown | MDXI01014421.1 |
|  | Diamondback_Terrapin_Tas2r12Ps | Pseudogene | Unknown | MDXI01014422.1 |
|  | Diamondback_Terrapin_Tas2r13Ps | Pseudogene | Unknown | MDXI01014422.1 |
|  | Diamondback_Terrapin_Tas2r14Ps | Pseudogene | Unknown | MDXI01018809.1 |
|  | Diamondback_Terrapin_Tas2r15Ps | Pseudogene | Unknown | MDXI01019232.1 |
|  | Diamondback_Terrapin_Tas2r16Ps | Pseudogene | Unknown | MDXI01020247.1 |
|  | Diamondback_Terrapin_Tas2r17Ps | Pseudogene | Unknown | MDXI01020247.1 |
|  | Diamondback_Terrapin_Tas2r18Ps | Pseudogene | Unknown | MDXI01020569.1 |
| *Pelodiscus sinensis* | Chinese_softshell_turtle_Tas2r12_Ps | Pseudogene | 1010 | NW_005857732.1 |
|  | Chinese_softshell_turtle_Tas2r2 | Intact | 109 | NW_005853641.1 |
|  | Chinese_softshell_turtle_Tas2r3 | Intact | 109 | NW_005853641.1 |
|  | Chinese_softshell_turtle_Tas2r4 | Intact | 109 | NW_005853641.1 |
|  | Chinese_softshell_turtle_Tas2r1 | Intact | 293 | NW_005851757.1 |
|  | Chinese_softshell_turtle_Tas2r5 | Intact | 588 | NW_005857331.1 |
|  | Chinese_softshell_turtle_Tas2r6 | Intact | 588 | NW_005857331.1 |
|  | Chinese_softshell_turtle_Tas2r11 | Intact | 747 | NW_005854414.1 |
|  | Chinese_softshell_turtle_Tas2r7 | Intact | 604 | NW_005858870.1 |
|  | Chinese_softshell_turtle_Tas2r8 | Intact | 604 | NW_005858870.1 |
|  | Chinese_softshell_turtle_Tas2r9 | Intact | 604 | NW_005858870.1 |
|  | Chinese_softshell_turtle_Tas2r10 | Intact | 604 | NW_005858870.1 |
| *Crocodylus porosus* | Saltwater_Crocodile_Tas2r1 | Intact | 572 | KN278197.1 |
|  | Saltwater_Crocodile_Tas2r2 | Intact | 7022 | KN282756.1 |
|  | Saltwater_Crocodile_Tas2r5 | Intact | 7022 | KN282756.1 |
|  | Saltwater_Crocodile_Tas2r3 | Intact | 10678 | KN285308.1 |
|  | Saltwater_Crocodile_Tas2r4 | Intact | 16288 | KN289208.1 |
| *Alligator mississippiensis* | American_Alligator_Tas2r1 | Intact | - | NW_014548347.1 |
|  | American_Alligator_Tas2r4 | Intact | - | NW_014546497.1 |
|  | American_Alligator_Tas2r6 | Intact | - | NW_014551907.1 |
|  | American_Alligator_Tas2r7 | Intact | - | NW_014547796.1 |
|  | American_Alligator_Tas2r8 | Intact | - | NW_014549531.1 |
|  | American_Alligator_Tas2r2 | Intact | - | NW_014552701.1 |
|  | American_Alligator_Tas2r3 | Intact | - | NW_014542701.1 |
|  | American_Alligator_Tas2r9 | Intact | - | NW_014547779.1 |
|  | American_Alligator_Tas2r10_Ps | Pseudogene | - | NW_014552701.1 |
|  | American_Alligator_Tas2r5 | Intact | - | NW_014546497.1 |
| *Alligator sinensis* | Chinese_Alligator_Tas2r1 | Intact | 534_1 | NW_005842050.1 |
|  | Chinese_Alligator_Tas2r11_Ps | Pseudogene | 1089_1 | NW_005842030.1 |
|  | Chinese_Alligator_Tas2r12_Ps | Pseudogene | 1089_1 | NW_005842030.1 |
|  | Chinese_Alligator_Tas2r6 | Intact | 537_1 | NW_005842734.1 |
|  | Chinese_Alligator_Tas2r2 | Intact | 1156_1 | NW_005842296.1 |
|  | Chinese_Alligator_Tas2r8_Ps | Pseudogene | 1156_1 | NW_005842296.1 |
|  | Chinese_Alligator_Tas2r9_Ps | Pseudogene | 1156_1 | NW_005842296.1 |
|  | Chinese_Alligator_Tas2r4 | Intact | 1666_1 | NW_005842817.1 |
|  | Chinese_Alligator_Tas2r7 | Intact | 1666_1 | NW_005842817.1 |
|  | Chinese_Alligator_Tas2r10_Ps | Pseudogene | 1666_1 | NW_005842817.1 |
|  | Chinese_Alligator_Tas2r3 | Intact | 653_1 | NW_005842498.1 |
|  | Chinese_Alligator_Tas2r5 | Intact | 653_1 | NW_005842498.1 |
| *Gavialis gangeticus* | Gharial_Tas2r10_Ps | Pseudogene | 6078 | KN329070.1 |
|  | Gharial_Tas2r1 | Intact | 789 | KN323950.1 |
|  | Gharial_Tas2r2 | Intact | 789 | KN323950.1 |
|  | Gharial_Tas2r3 | Intact | 3395 | KN326545.1 |
|  | Gharial_Tas2r4 | Intact | 4682 | KN327704.1 |
|  | Gharial_Tas2r6 | Intact | 4682 | KN327704.1 |
|  | Gharial_Tas2r11_Ps | Pseudogene | 14140 | KN336701.1 |
|  | Gharial_Tas2r5 | Intact | 17078 | KN345040.1 |
|  | Gharial_Tas2r7 | Intact | 17078 | KN339459.1 |
|  | Gharial_Tas2r8 | Intact | 19960 | KN342124.1 |
|  | Gharial_Tas2r9_P | Partial | C15964618_1 | JRWT01292050.1 |
