# Supplementary material for: Genomic evidence of bitter taste in snakes and phylogenetic analysis of bitter taste receptor genes in reptiles
Source: PeerJ. 2017 Aug 18;5:e3708. doi: 10.7717/peerj.3708 (PMC5564386; doi:10.7717/peerj.3708)
Supplement: Table S6 — Genome size (pg) is the amount of DNA in one copy of a species’ chromosomes or the haploid nuclear DNA content. [file peerj-05-3708-s012.docx]

Table S6 Genome Size in reptiles. Genome size (pg) is the amount of DNA in one copy of a species' chromosomes or the haploid nuclear DNA content.

|  | Number of Species | Average Genome size (pg) |
| --- | --- | --- |
| Crocodilians | 10 | 2.86 |
| Turtles | 64 | 2.8 |
| Lizards | 229 | 2.15 |
| Snakes | 115 | 2.14 |
| Birds | 75 | 1.43 |
