# Supplementary material for: Genomic evidence of bitter taste in snakes and phylogenetic analysis of bitter taste receptor genes in reptiles
Source: PeerJ. 2017 Aug 18;5:e3708. doi: 10.7717/peerj.3708 (PMC5564386; doi:10.7717/peerj.3708)
Supplement: Table S7 — Branch model in PAML was used to compute the ω value. [file peerj-05-3708-s013.docx]

| Table S7. Likelihood ratio tests of selective pressures on *Calhm1* genes of snakes. **Branch model in PAML was used to compute the** ω value. | | | | | | | | | |
| --- | --- | --- | --- | --- | --- | --- | --- | --- | --- |
| **Model** | **np** | **Ln L** | **Estimates of parameters** | | | **Model compared** | **LRT P-value** | **Omega for Branch** |  |
| **Two ratio Model 2** | 21 | -3795.691099 | ω: | 0.11909 | 0.09704 | Model 0 vs. Two ratio Model 2 | 0.494690583 | 0.11909 |  |
| Model 0 | 20 | -3795.924253 | ω= | 0.11525 | |  |  |  |  |
